# Illuminating a Solvent-Dependent Hierarchy for Aromatic CH/π Complexes with Dynamic Covalent Glyco-Balances

**DOI:** 10.1021/jacsau.3c00592

**Published:** 2024-01-02

**Authors:** Laura Díaz-Casado, Alejandro Villacampa, Francisco Corzana, Jesús Jiménez-Barbero, Ana M. Gómez, Andrés G. Santana, Juan Luis Asensio

**Affiliations:** † Departamento de Química Bio-Orgánica, Instituto de Química Orgánica General (IQOG-CSIC), Consejo Superior de Investigaciones Científicas (CSIC), 28006 Madrid, Spain; ‡ Departamento de Química, Centro de Investigación en Síntesis Química, Universidad de La Rioja, 26006 Logroño, Spain; § Basque Researchand Technology Alliance (BRTA), CIC bioGUNE, 48170 Derio, Spain; ∥ Basque Foundation for Science, Ikerbasque, 48009 Bilbao, Spain; ⊥ Centro de Investigación Biomédica En Red de Enfermedades Respiratorias, 28029 Madrid, Spain; # Department of Chemistry of Natural Products and Bioactive Synthetics, Instituto de Productos Naturales y Agrobiología (IPNA-CSIC), San Cristóbal de La Laguna, Santa Cruz de Tenerife 38206, Spain

**Keywords:** CH/π complexes, molecular glyco-balance, solvent dependent stability, dynamic covalent chemistry, isotope labeling, NMR

## Abstract

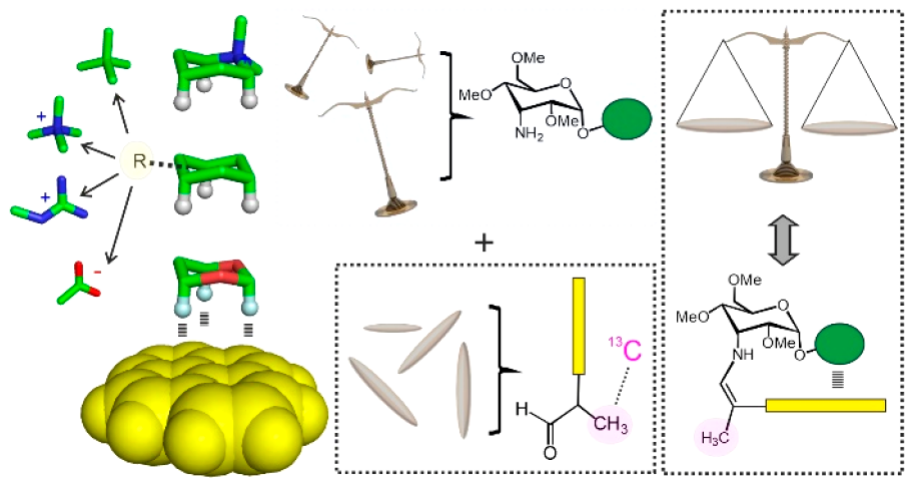

CH/π interactions are prevalent among aromatic complexes and represent invaluable tools for stabilizing well-defined molecular architectures. Their energy contributions are exceptionally sensitive to various structural and environmental factors, resulting in a context-dependent nature that has led to conflicting findings in the scientific literature. Consequently, a universally accepted hierarchy for aromatic CH/π interactions has remained elusive. Herein, we present a comprehensive experimental investigation of aromatic CH/π complexes, employing a novel approach that involves isotopically labeled glyco-balances generated *in situ*. This innovative strategy not only allows us to uncover thermodynamic insights but also delves into the often less-accessible domain of kinetic information. Our analyses have yielded more than 180 new free energy values while considering key factors such as solvent properties, the interaction geometry, and the presence and nature of accompanying counterions. Remarkably, the obtained results challenge conventional wisdom regarding the stability order of common aromatic complexes. While it was believed that cationic CH/π interactions held the highest strength, followed by polarized CH/π, nonpolarized CH/π, and finally anionic CH/π interactions, our study reveals that this hierarchy can be subverted depending on the environment. Indeed, the performance of polarized CH/π interactions can match or even outcompete that of cationic CH/π interactions making them a more reliable stabilization strategy across the entire spectrum of solvent polarity. Overall, our results provide valuable guidelines for the selection of optimal interacting partners in every chemical environment, allowing the design of tailored aromatic complexes with applications in supramolecular chemistry, organocatalysis, and/or material sciences.

## Introduction

**Figure 1:**
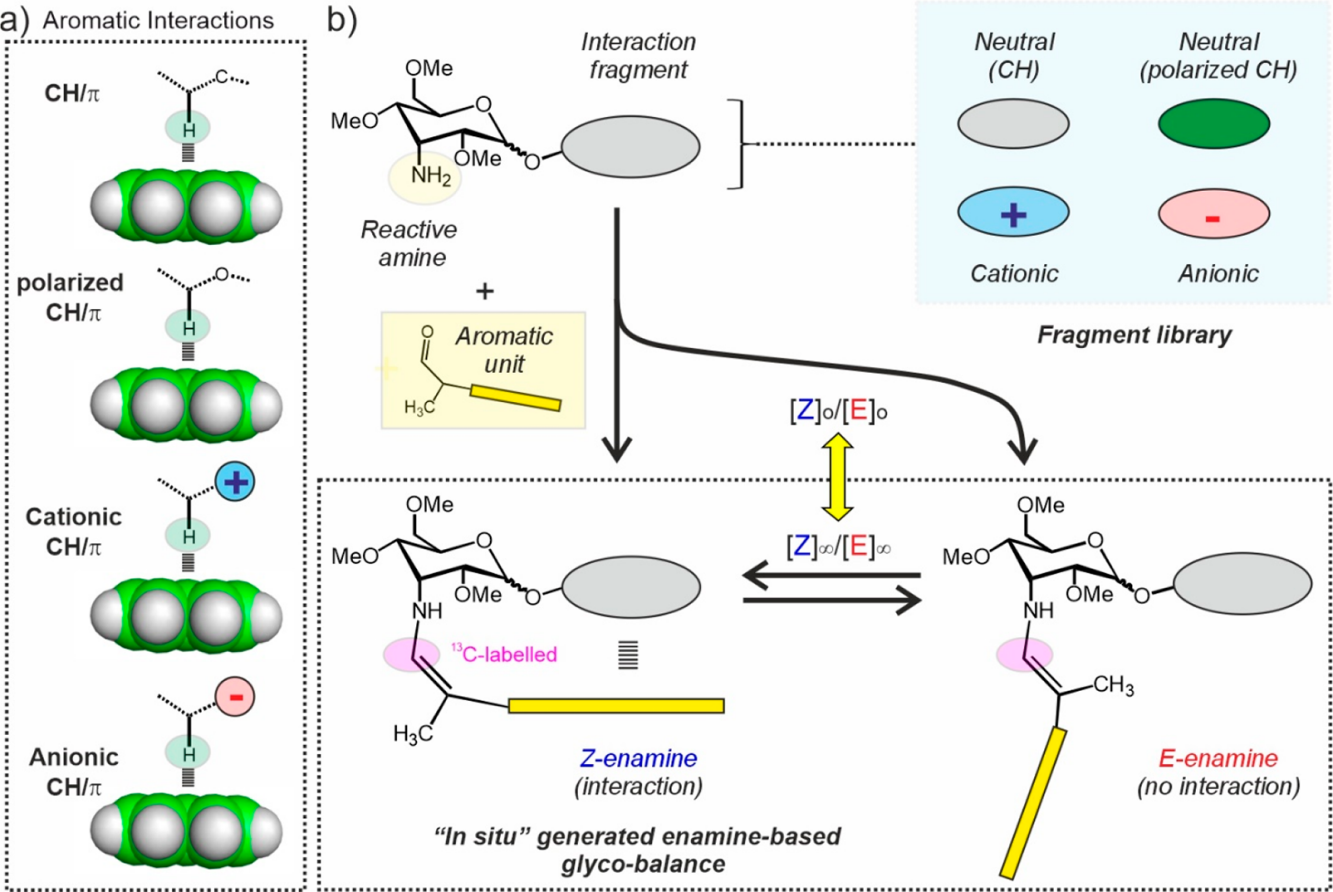
a) Aromatic X-CH/π complexes analyzed in this study. Binding free energies can be modulated by polarizing, cationic, or anionic fragments. b) Schematic representation of the *in situ* generated glyco-balances employed in this work.

Aromatic units present a rich repertoire of noncovalent interaction modes, playing essential roles in the stabilization of biomolecular architectures, binding complexes and transition states.^1−5^ Their versatility as recognition elements stems from their unusual capacity to simultaneously benefit from solvophobic and electrostatic forces while maintaining a significant stability over a wide range of conditions. This dual character renders aromatic contacts key actors in a plethora of research fields ranging from supramolecular chemistry,^6,7^ through material chemistry,^8^ all the way to biocatalysis^9^ and organocatalysis.^10−13^ Many of these noncovalent bonds involve alkyl CH fragments, whose attractive forces with the aromatic domain are strongly modulated by structural and environmental factors. In particular, CH fragments attached to polarizing electronegative atoms, or even cationic or anionic functions, generate a significant diversity of complexes commonly included within CH/π, cation/π, and anion/π categories, respectively (Figure 1a).

Water is the natural environment for biological phenomena to happen, and consequently, it represents the obvious choice for chemical studies on aromatic complexes. Indeed, much of the research revolving around this topic has been focused on naturally occurring examples, such as those provided by carbohydrate binding proteins,^14,15^ glycoproteins,^16^ or water-soluble synthetic models.^17−20^ Sufficient experimental evidence has accrued over recent years to conclude that aromatic interactions stabilized by up to three neutral CH/π contacts can exhibit free energy values in the 1.0–2.0 kcal/mol range.^14,18^ Furthermore, the polarization of these CH moieties by electron-withdrawing substituents seems to have a strengthening influence close to 0.3 kcal/mol per interacting CH function, amounting to up 1 kcal/mol extra.^19^ In contrast, the incorporation of cationic fragments exerts a more diverse influence on the interaction free energy, their final contribution being extremely reliant on both desolvation processes and long-range electrostatic forces.^21−26^ Despite this context-dependent character, studies performed with synthetic neutral receptors^26^ have shown that cationic functions tend to form weaker aromatic complexes than their neutral isosteres, which reflects the stronger preference of charged moieties for water. Finally, while comparatively less analyzed, anionic functions should lead to repulsive interactions with the π cloud of electron-rich aromatics, yet their magnitude is conditioned by the geometry of the complex.^27−29^ In summary, according to current knowledge, the strength of aromatic X-CH/π contacts in water is heavily governed by the chemical nature of the X-substituent and, overall, follows the trend: polarized CH/π > nonpolarized CH/π > cation/π > anion/π.

On this matter, it should be considered that protein binding sites might be better represented by an organic rather than an aqueous interphase,^30^ a statement that is especially true for buried pockets. In addition, it has been shown that cationic CH/π contacts are involved in the membrane lipids interfacial binding by amphitropic proteins.^31^ However, the extent to which the aforementioned hierarchy is maintained or subverted in nonpolar media remains unclear due to a lack of extensive and systematic studies on this topic.^32−34^ In particular, the modulatory influence exerted by CH-polarizing fragments or charged substituents has been hardly analyzed in the past. Overall, reported results point to a prevalence of the cationic complexes with respect to the neutral pairs in nonaqueous media, with free energies for the former being comparable or lower than those derived in water. However, these works still suffer from a limited scope, dealing with a rather reduced set of complex types and geometries, interacting pairs, solvents, and counterions. Indeed, a systematic analysis accounting for variations in the main relevant parameters is still missing.

Herein, we report on an extensive and thorough structure/stability analysis of aromatic complexes of the form X-CH/π, where X accounts for nonpolarizing, polarizing, cationic, or anionic fragments (Figure 1a) and is placed at different sites with respect to the aromatic platform. On the methodological side, this study relies on a new reactivity-based strategy that incorporates features from dynamic combinatorial approaches previously developed by our group to dissect CH/π bonds in water.^17−19^ This procedure is based on the *in situ* generation of molecular glyco-balances in NMR tubes and combines isotopic labeling with the NMR monitoring of chemical reactions, having the potential of providing kinetic as well as thermodynamic information. In total, this analysis has yielded more than 180 new accurate free energy values, delivering a detailed picture of the solvent-dependent structure/stability relationships that govern the formation of aromatic X-CH/π complexes.

## Results

### *In Situ* Generated Glyco-Balances for the Analysis of Aromatic Complexes: Design Principles

The proposed methodology is schematically represented in Figure 1b and makes use of a library of 3-amino-3-deoxy-d-allopyranosides equipped with alternative interacting fragments embedded in the aglycone moiety. Upon reaction with 2-aryl-propionaldehydes, these derivatives evolve to yield a *Z*/*E* mixture of enamines in exchange either through full dissociation or via intramolecular imine/enamine tautomerism. Given that only the *Z* stereoisomer is compatible with the establishment of an aromatic interaction with the aglycone, the final **Z*/*E** equilibrium ratio will be tilted by the strength of such contacts, whose free energy can be derived by employing the appropriate reference compounds. For simplicity, the proposed reactions were performed in NMR tubes, and their evolution followed with simple 2D experiments (see below).

For such an approach to work, designed models must fulfill several critical requirements. As a first consideration, the enamine species should present sufficient stability against chemical evolution, such as aldol-type reactions, thus preventing chemical quenching of the equilibrium mixture. These parasitic reactions were selectively hindered by installing a methyl group at the α-position of the employed aldehydes. In addition, enamine detection should be sensitive enough for an accurate evaluation of the *Z/E* populations during the reaction course. To facilitate this, we resorted to 2-aryl-propionaldehydes with a ^13^C-labeled carbonyl group, which allowed for a convenient monitoring of the reaction kinetics by means of sequential HSQC-type experiments. Finally, product mixtures should be ideally dominated by the enamines, with only marginal concentrations of hemiaminal or imine intermediates remaining. Preliminary assays showed that all these criteria were met only by 3-aminoallose models with α configuration. The observed behavior can be rationalized according to the differential influence exerted by the axially oriented α-anomeric substituent on the interchangeable imine and enamine species, respectively: while the former is electrostatically disfavored, the latter is strongly stabilized by an intramolecular hydrogen bonding interaction (Figure S1).

The proposed strategy presents several key differences with respect to previously described approaches. First, while known molecular balances^35−38^ typically rely on a conformational exchange process consisting of a hindered rotation about a particular covalent bond, our system makes use of a *configurational Z/E* exchange, an alternative hitherto unexplored by the chemical community. Second, the *in situ generation strategy* offers significant flexibility, providing a simple means to assay alternative aromatic platforms with the same 3-aminoallose module. Third, the designed derivatives present a certain degree of conformational flexibility, mainly located at the glycosidic and 3-aminoallose C_3_–N torsions, which permits alleviating potential steric clashes between the interacting fragments, thus implementing some optimization of the aromatic complexes established within the *Z* enamines. Fourth, monitoring of the reaction kinetics allows deriving not just the equilibrium *Z/E* enamine populations but also their relative formation rates. In this way, the impact of the aromatic interactions *at both the kinetic and thermodynamic levels* can be evaluated.

### Description of the Designed Libraries

**Figure 2:**
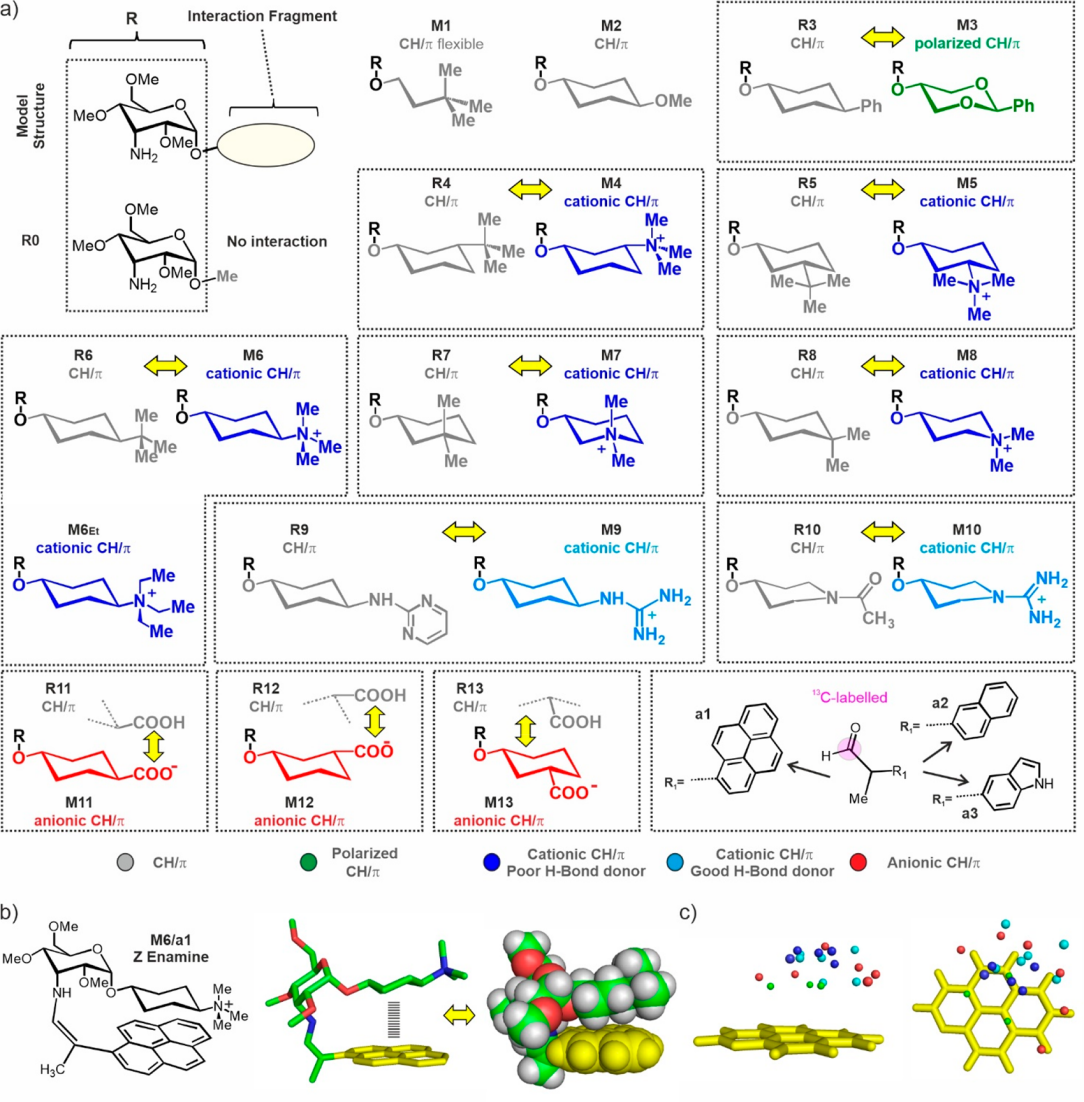
a) Library of 3-aminoallose derivatives and ^13^C-labeled 2-arylpropionaldehydes synthesized for the *in situ* generation of the molecular glyco-balances. Interaction fragments are colored according to the code indicated at the bottom. Isosteric charged/neutral fragment pairs are shown in the same box. b) Minimized model (general Amber force field GAFF, explicit dichloromethane solvent) for the *Z*-enamine generated from pair **M6**/**a1**. c) Relative position for key atoms (polarized H-**M3**, green; N^+^-**M4**–**M8**, blue; N^+^-**M9** and -**M10**, cyan; and O^–^-**M11**–**M13**, red) with respect to the pyrene unit in minimized *Z*-enamines.

Figures 2a, S2, and S3 show the 3-aminoallose-based scaffolds considered in our analysis.

Derivative **R0α**, devoid of any interaction fragment attached to the 3-aminoallose anomeric position was employed as absolute reference to derive free energies of interaction (Δ*G*_int_) for all the analyzed complexes. The rest of our models incorporate linear (**M1**) or cyclic (**M2**) interaction fragments equipped with polarizing electronegative atoms (**M3**), exocyclic (**M4**–**M6** and **M6Et**) or endocyclic (**M7** and **M8**) tetra-alkyl-ammonium functions, exocyclic guanidinium groups (**M9** and **M10**), and carboxylates (**M11**–**M13**) at different positions so that a variety of complex geometries are being sampled (Figure 2b,c). Our selection of cationic fragments covers both poor (**M4**–**M8**) and excellent hydrogen-bond donors (**M9** and **M10**). Derivative **M6Et** represents an **M6** analogue equipped with ethyl fragments attached to the quaternary nitrogen. To facilitate the solubility of all these species in various organic media, a large organic anion such as bistriflylimide was selected as a counterion, unless otherwise stated.

For comparison purposes, we prepared a neutral isostere for every cationic model (compounds **R4**–**R10**). Similarly, an **M3** analogue devoid of polarizing oxygen atoms was included in the library (**R3**). These references allowed determining the precise contribution made by the positive charges or the polarizing heteroatoms on to the free energy of the corresponding aromatic complexes (herein termed Δ*G*_charge_ or Δ*G*_pol_, respectively). Regarding the anionic compounds (**M11**–**M13**), they were generated in the NMR tube upon treatment of the neutral carboxylic acids (**R11**–**R13**), employed as references, with DBU as the organic base.

Inspection of minimized models (Figure 2b) reveals that the aromatic interactions established by the *Z* enamine species are in all cases dominated by CH/π contacts. However, their stabilities are expected to be highly dependent on the presence or absence of polarizing, cationic, or anionic functions in a solvent-dependent manner and, therefore, they were broadly classified as nonpolarized CH/π, polarized CH/π, cationic CH/π (poor H-bond donor substituents), cationic CH/π (good H-bond donor substituents), and anionic CH/π complexes, according to the color code shown at the bottom of Figure 2a and maintained throughout this manuscript.

Finally, 2-arylpropionaldehydes equipped with electron-rich aromatic units of different sizes and electronic properties^39,40^ were prepared. It should be noted that the inclusion of π-acidic aromatics in the balances is not of interest because CH/π interactions would become repulsive. These moieties included a pyrene (**a1**), naphthalene (**a2**), or indole (**a3**) as fused-ring systems, whose π-basic character shall determine attractive and repulsive interactions with cationic and anionic fragments, respectively (Figure 2).

### General Synthetic Strategy

**Scheme 1:**
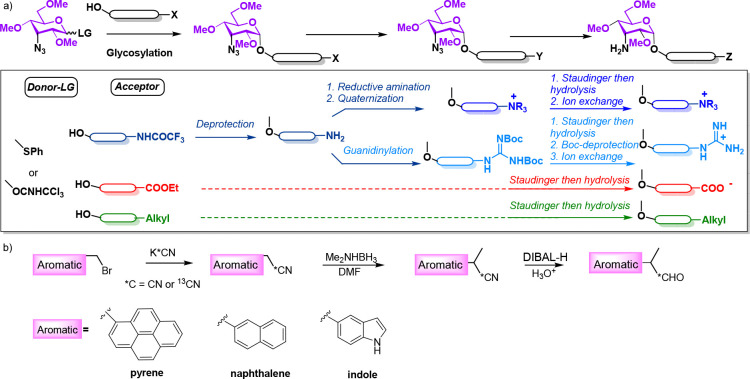
General Synthetic Routes Employed for the preparation of (a) the designed 3-aminoallose derivatives and (b) ^13^C-labeled 2-aryl-propionaldehydes.

To analyze the cationic, anionic, or neutral CH/π complexes in a wide range of organic solvents with varying polarities, we constructed our model systems from a common 3-azido-3-deoxy-d-allopyranosyl donor protected with nonparticipating methoxy groups, which would then undergo a glycosylation reaction with different acceptor alcohols to provide the corresponding α-glycosides. This stereoselectivity was required for a suitable parallel orientation within our hairpinlike scaffold between the α-axial 3-azide, which would be eventually transformed into the desired amine, and the acceptor interacting unit. Further processing of the aglycone moiety entailed deprotection, reductive amination, and alkylation to yield the corresponding tetra-alkyl-ammonium cationic species or acid promoted Boc-deprotection of the guanidines. Likewise, anionic species were generated after basic ester hydrolysis. Once the charged functional group had been installed, we normally proceeded to reduce the azide moiety under Staudinger conditions with PMe_3_, followed by an ion exchange step to replace the accompanying anion (typically iodide) by the noncoordinating bistriflylimide. This last salt metathesis reaction was necessary to ensure better solubility in organic solvents (Scheme 1a).

Regarding the aromatic counterparts required for the assembly of our enamine-based molecular glyco-balances, the synthesis started from the commercially available benzylic bromides, which were treated with ^13^C-labeled potassium cyanide to produce the corresponding nitrile derivatives. This isotopic labeling is meant to improve the sensitivity of our NMR methodology to allow for smooth monitoring of the reaction kinetics. Subsequent elaboration of these substrates involved an α-alkylation reaction^41^ to yield the corresponding racemic 2-aryl-propionitriles, which were later reduced with DIBAL-H under low temperature conditions to provide the desired branched aromatic aldehydes (Scheme 1b).

### Preliminary Assays and Control Experiments

**Figure 3:**
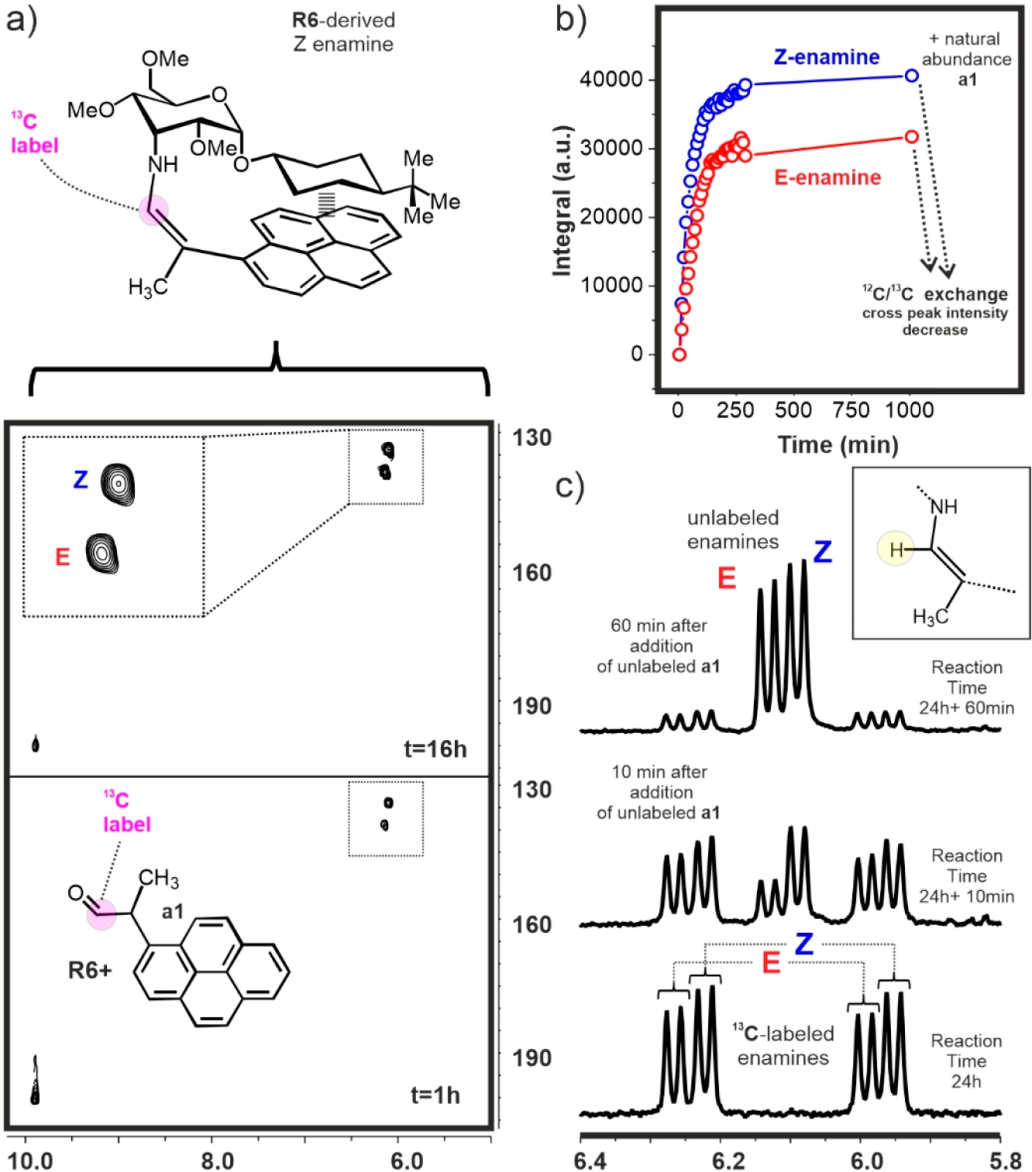
a) HSQC spectra acquired with derivative **R6** after addition of ^13^C-labeled aldehyde **a1** in dichloromethane-*d*_2_ at 20 °C at 1 h (bottom) and 16 h (top). b) Kinetic curves derived upon integration of the *Z*/*E* enamine HSQC cross-peaks. Subsequent addition of unlabeled aldehyde **a1** after reaction completion promotes a ^13^C/^12^C enamine exchange, leading to a sudden decrease in the intensity of the HSQC cross peaks. c) Exchange of ^13^C-labeled by natural abundance enamines promoted by the addition of unlabeled **a1**, as revealed by 1D NMR (see the main text).

Chemical reactions in NMR tubes were initiated by the addition of a substoichiometric amount of ^13^C-labeled aldehyde to a solution containing any of the aminated models. Time evolution of the reaction mixture was followed by means of sequential 2D-HSQC experiments. To illustrate this simple *in situ* generation protocol, Figure 3 shows HSQC experiments acquired 1 and 24 h after the addition of ^13^C-labeled aldehyde **a1** (0.5 equiv) to model **R6** in dichloromethane-*d*_2_. Both data sets are remarkably simple, presenting single cross-peaks for the *Z*- and *E*-enamine species (see Figure S4 for assignment). Integration of these signals over time allowed us to derive kinetic curves for both species, which stabilized in approximately 200 min. It is worth mentioning that *Z* enamine formation is in all cases accompanied by the appearance of strongly upfield shifted signals in the 1D spectrum, which is indicative of shielding resulting from aromatic complex formation (Figure S5). After overnight equilibration of this reaction mixture, an excess of unlabeled aldehyde **a1** was added to the NMR tube to prove that the exchange between the starting 3-aminoallose and the enamine species remained active. This immediately produced a drop in the intensity of the HSQC cross-peaks, together with the appearance of unlabeled enamine signals in the 1D spectrum, implying that the observed stabilization of the enamine concentrations indeed corresponded to a true equilibrium situation (Figure 3).

**Figure 4:**
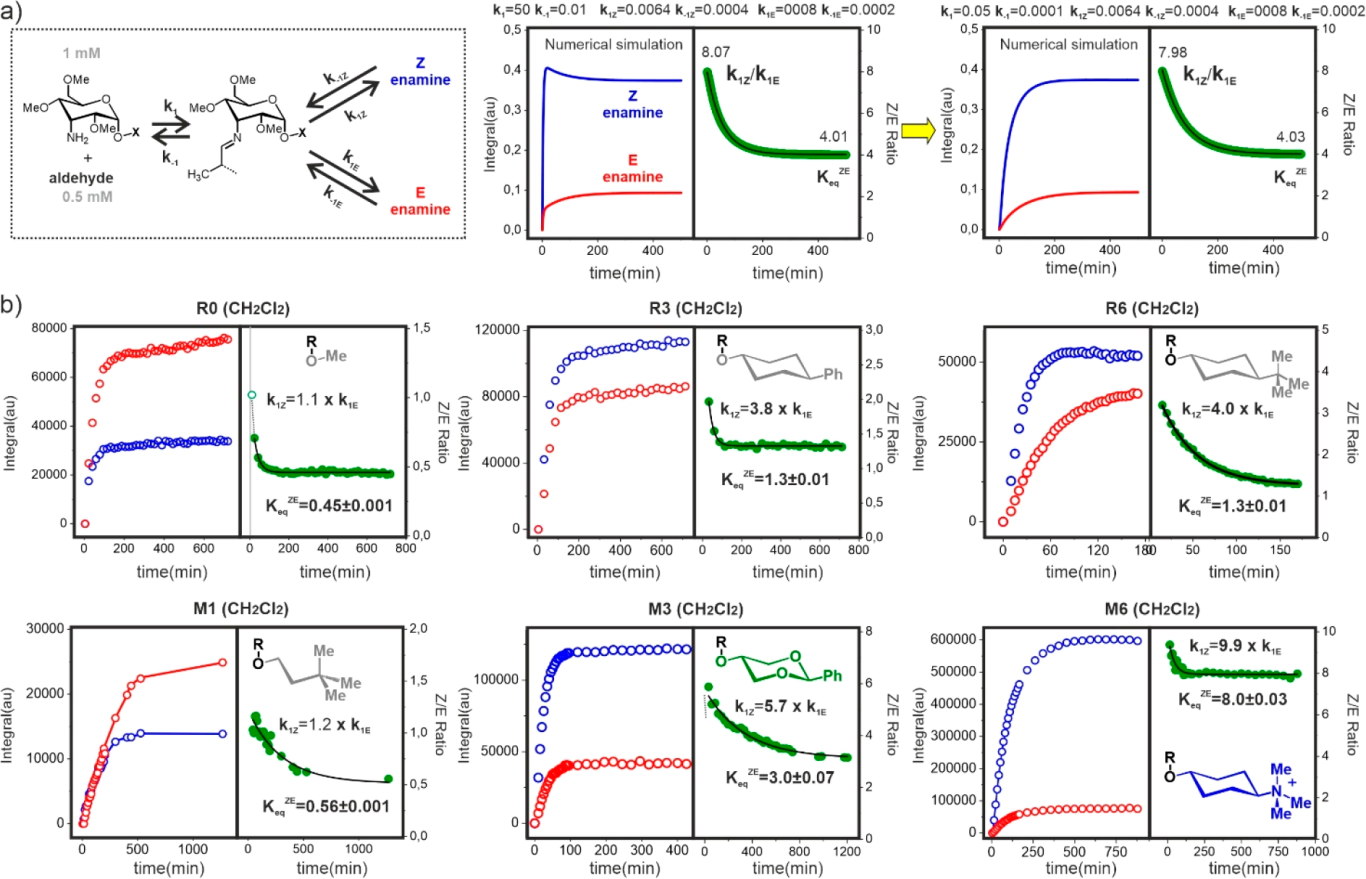
a) Simple kinetic model considered for our Gepasi kinetic simulations. Middle and right panels show theoretical curves calculated assuming fast or slow imine formation on the reaction time-scale along with the employed kinetic constants. Time evolution of the corresponding *Z*/*E* ratios, in green, is represented on the right together with the fitted curves and the derived *K*_eq_^*Z*/*E*^ and *k*_**Z**_/*k*_*E*_ values. b) Experimental curves obtained for models **R0**, **M1**, **R3**, **R6**, **M3**, and **M6** upon treatment with aldehyde **a1**, together with the derived *K*_eq_^*Z*/*E*^ and *k*_*Z*_/*k*_*E*_ values.

In order to get further insights into the enamine formation process, we performed extensive theoretical kinetic simulations with the program Gepasi,^42^ employing for our analysis the kinetic regime represented in Figures 4a and S6 that comprises a network of three interrelated reactions: a bimolecular formation of an imine/hemiaminal adduct, followed by an intramolecular arrangement to give rise to either the *Z* or the *E* enamine. According to this, the system is defined by six rate constants, herein referred to as *k*_1_, *k*_–1_, *k*_1*Z*_, *k*_–1*Z*_, *k*_1*E*_, and *k*_–1*E*_ (Figure 4).

Our initial assumption was that formation of the imine intermediate is fast relative to the subsequent unimolecular reactions, with such an equilibrium constant in the millimolar range (*k*_+1_/*k*_–1_ = 5000 M^–1^). In this scenario, kinetic curves for *Z*- and *E*-enamines can exhibit different evolution pathways, showing a conventional exponential profile or a more complex shape characterized by the presence of a maximum followed by a slow concentration decrease toward the final equilibrium value (Figure 4a, middle panel). Interestingly, the ratio between the two kinetic curves (Figure 4a, green) can be approximated by a simple exponential function (see the experimental section), whose values at *t* = 0 and ∞ provide access to the initial and final *Z*/*E* concentration ratios (equal to *k*_1*Z*_/*k*_1*E*_ and the equilibrium constant *K*_eq_^*Z*/*E*^, respectively). On the other hand, the assumption of a slow bimolecular step followed by faster intramolecular processes leads to a normalization of the enamine kinetic profiles (Figure 4a, left panel). Under these circumstances, the concentration ratio approaches equilibrium faster than the individual *Z* or *E* curves, so much that the equilibrium constant can be derived even from incomplete kinetic experiments (Figure S6). Examples of all these behaviors were later detected in the experimental assays carried out throughout this study.

To illustrate our approach, Figure 4b shows experimental data sets measured in dichloromethane-*d*_2_ with models **R0**, **M1**, **R3**, **M3**, **R6**, and **M6** and aldehyde **a1**, together with the corresponding *Z*/*E* ratios. Fitted curves are shown in black next to the deduced *K*_eq_^*Z*/*E*^ and *k*_1*Z*_/*k*_1*E*_ values. As expected, it can be observed that in the absence of an interaction fragment (model **R0**), the final *Z*/*E* equilibrium is dominated by the *E* stereoisomer (*K*_eq_^*Z*/*E*^ = 0.45), whose formation rate is similar to that of the *Z* derivative (*k*_1*Z*_ = 1.1*k*_1*E*_). An analogous behavior was observed for **M1,** showing that a flexible alkyl chain has a very limited capacity to favor the *Z*-enamine, either kinetically or thermodynamically. In contrast, preorganized cyclohexyl fragments, such as those present in **R3** and **R6**, lead to clear stabilizing CH/π dispersive contacts with the pyrene platform upon formation of the *Z*-enamine, which now dominates the equilibrium (*K*_eq_^*Z*/*E*^ = 1.3 for both **R3** and **R6**). Remarkably, this aromatic interaction has an impact on the relative formation rates of the enamines, too, with the *Z* stereoisomer now forming faster (*k*_1*Z*_/*k*_1*E*_ = 3.8 for **R3** and 4.0 for **R6**). In the same way, the replacement of two cyclohexyl methylene groups by oxygen atoms (model **M3**) establishes a polarizing influence on the three interacting CH groups, which enables an electrostatically enhanced complex with the pyrene in the *Z*-enamine species. As a consequence, the final equilibrium is more dominated by the *Z*-enamine, now showing a 3-fold increase in concentration with respect to the *E*-stereoisomer. Again, this stabilization of the aromatic complex (from nonpolarized CH/π to polarized CH/π) translates in a larger rate constant ratio in favor of the more stable species (*k*_1*Z*_/*k*_1*E*_ = 5.7). Finally, a trimethylammonium moiety attached to the cyclohexyl anomeric substituent (**M6**) further stabilizes the *Z*-enamine via cation/π forces. Consequently, this now forms 9.9 times faster than the *E*-stereoisomer, yielding an equilibrium constant of *K*_eq_^*Z*/*E*^ = 8.0.

A common trend along this series is that more stable *Z* stereoisomers tend to form faster in relation to the corresponding *E*-derivatives. Indeed, the relative *Z*/*E* formation rates and stabilities show a certain correlation. This observation suggests that the aromatic complex established by the pyrene may already be somewhat present in the imine/hemiaminal intermediates, leading to a conformational preorganization that facilitates a faster *Z*-enamine formation. In summary, both the equilibrium constants and the relative formation rates can provide independent evidence for the complex stabilities.

Regarding the equilibrium constants (*K*_eq_^*Z*/*E*^) derived by this procedure, they were translated into free energies Δ*G*_*Z*/*E*_ (according to Δ*G*_*Z*/*E*_ = −*RT* ln *K*_eq_^*Z*/*E*^) that, once subtracted from that of reference compound **R0**, provided the stabilities of the alternative aromatic complexes, herein referred to as free energies of interaction, Δ*G*_int_ (Δ*G*_int_^model^ = Δ*G*_*Z*/*E*_^model^ – Δ*G*_*Z*/*E*_^R0^). Likewise, a comparison between free energy values for CH-polarized or charged complexes (such as those established by **M3** and **M6**, respectively) with those measured for the neutral isosteres (**R3** and **R6**) allows determining the net contribution provided by the CH-polarization (Δ*G*_pol_) or charge (Δ*G*_charge_) to the stability of the complexes (i.e., Δ*G*_pol_^M3^ = Δ*G*_*Z*/*E*_^M3^ – Δ*G*_*Z*/*E*_^R3^ and Δ*G*_charge_^M6^ = Δ*G*_*Z*/*E*_^M6^ – Δ*G*_*Z*/*E*_^R6^).

### Proof of Concept: Assays in Dichloromethane

**Figure 5:**
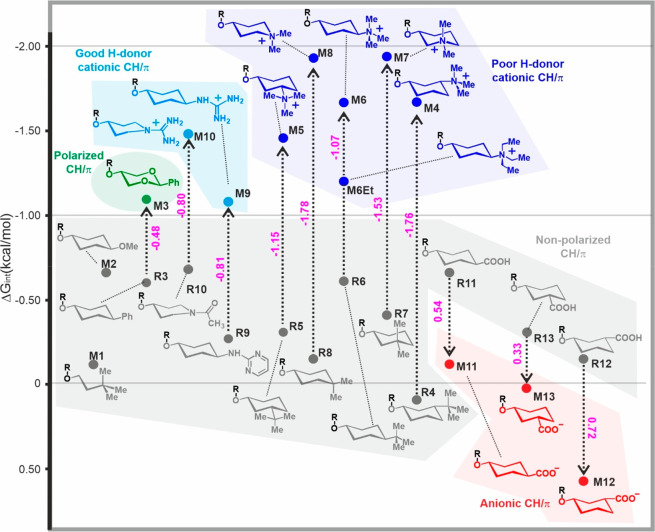
Interaction free energy values Δ*G*_int_ (kcal/mol) measured in dichloromethane-*d*_2_ at 20 °C. Dotted arrows connect models with their references, where Δ*G*_charge_ or Δ*G*_pol_ (kcal/mol) values are shown in magenta.

As a proof of concept, the *in situ* glyco-balance generation protocol was first assayed in dichloromethane-*d*_2_ employing aldehyde **a1**. Figure 4 shows examples of obtained kinetic curves together with the derived *K*_eq_^*Z*/*E*^ values (see also Figure S7 and Tables S1 and S2). Interaction free energies for the alternative complexes are listed in Figure 5. In this representation, the different complex categories are grouped by colors according to the code stated in Figure 2. In addition, ionic or CH-polarized complexes have been linked to their corresponding neutral isosteres with a vertical arrow, and the specific free energy contributions made by charges or CH-polarization (Δ*G*_charge_ or Δ*G*_pol_, respectively), which are proportional to the arrow length, are indicated in magenta.

Several conclusions can be drawn from the obtained experimental data. As a first observation, the analyzed aromatic interactions present a notable dispersion of free energy values, covering a window of approximately 2.5 kcal/mol. Even within the same type of interaction, the observed free energy variations are substantial. As an example, the cationic CH/π contacts involving tetra-alkyl-ammonium salts show a mean stability of −1.64 ± 0.28 kcal/mol, while the corresponding value for nonpolarized CH/π complexes is −0.37 ± 0.25 kcal/mol. This variability reflects the diversity of the conformational and dynamic behaviors of the analyzed complexes. In short, the question of how much an aromatic CH/π complex is worth in terms of free energy does not have a general answer since it is extremely dependent on the chemical context.

Under the employed conditions, the largest stabilities correspond to the cationic complexes, with maximum free energy stabilizations of close to −2 kcal/mol. Overall, tetra-alkyl-ammonium salts promote stronger complexes than guanidinium fragments, probably reflecting the weak hydrogen-bonding character of the former, which limits the competition exerted by counterions and solvent. This view is supported by molecular dynamics simulations that reveal closer cation/counterion contacts for the guanidinium derivatives (Figure S8). The position of the tetra-alkyl-ammonium moiety with respect to the aromatic unit also seems to play an important modulatory role. Indeed, observed free energies are scattered between −1.20 and −1.93 kcal/mol, with more negative values corresponding to fragments **M7** and **M8**, which place the cationic moieties in a more centered spot over the pyrene unit. Regarding models **M6** and **M6Et**, the former generates a stronger complex, probably reflecting a less disperse positive charge, and thus more conveniently located at the interacting cyclohexyl fragment.

Positive charge contributions (accounted for by Δ*G*_charge_, see Table S2) derived from the stabilities of cationic complexes and those formed by the isosteric neutral species are, in all cases, favorable and represent a significant fraction of the corresponding interaction free energies (Δ*G*_int_, for example 64% for **M6** or 54% for **M10**). An interesting remark regarding this parameter is that charge contributions tend to be larger for those complexes characterized by poor shape complementarity. For example, according to molecular models, aromatic interactions established by pairs **R4**/**M4** or **R8**/**M8** are somewhat destabilized by steric clashes with methyl groups of the aglyconic moiety (Figures 5 and S9). However, this energy penalty is more severe for the neutral reference complexes, solely stabilized by dispersive forces, which determines a greater stability gap with respect to the more tolerant cationic species. *According to this, the introduction of positive charges emerges as a particularly valuable strategy to enhance the interaction between poorly complementary surfaces.*

Among all neutral complexes, the one equipped with polarized CH groups (**M3**) exhibits the largest interaction free energy, well above −1.0 kcal/mol. Remarkably, this value is equivalent to those derived for the less stable cationic complexes, *thus representing a practical tool for the construction of complex architectures in nonpolar media*.

Regarding nonpolarized CH/π interactions, these contacts occupy the third position in the stability ranking. Neutral complexes are mainly favored by dispersive forces and consequently display the highest sensitivity to shape-complementarity. In agreement with this view, most cyclohexyl fragments (**M2**, **R3**, etc.), exhibiting three axial CH groups already preorganized for aromatic contacts, form stronger complexes than the linear flexible chain present in **M1**.

Finally, the most unstable complexes among our set of models are those negatively charged, with their strengths being dependent on the distance between the pyrene ring and the carboxylate functions. In particular, complex **M12**, with the closest carboxylate/aromatic proximity, exhibits the largest positive free energy value (Figure 5). The behavior observed for these anionic complexes in comparison with their corresponding neutral carboxylic acids shows that the charge contribution to the free energy (Δ*G*_charge_) is always repulsive, as expected for electron-rich aromatic units. However, the values ultimately measured (Table S2) are lower in absolute value than the corresponding favorable contributions for cations. This observation may reflect the more limited capacity of negative charges to partially delocalize within the cyclohexyl hydrocarbon skeleton through hyperconjugation and inductive effects, as is the case with positive charges. Regardless of the relative position of the carboxylate group in these complexes, attractive interactions with the partially positive edge of the aromatic platform are not evidenced in any case.

### Broad Environmental Sensitivity of the Aromatic Complexes: Stability Studies in Organic Media

**Figure 6:**
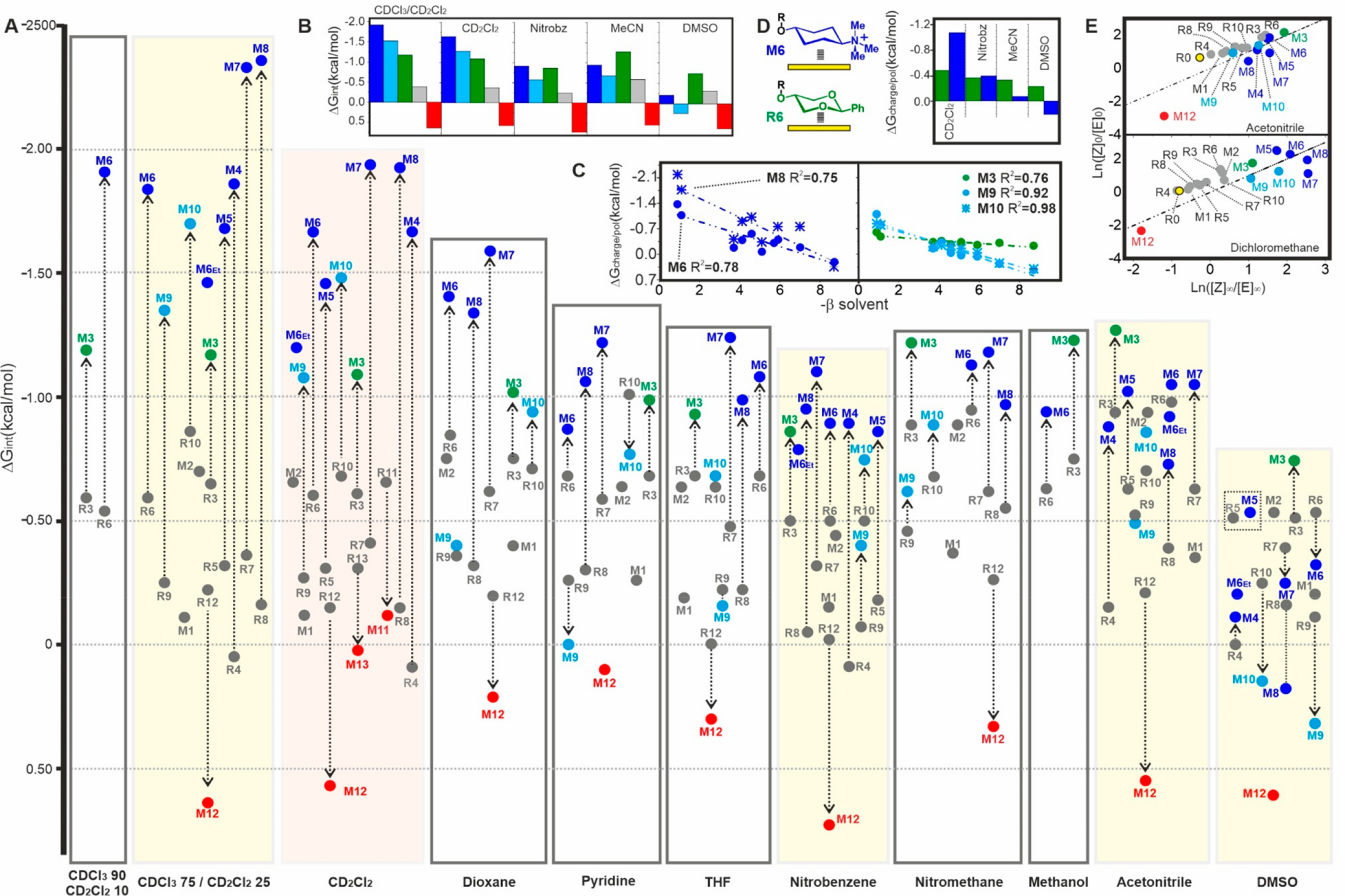
a) Interaction free energies derived for selected CH/π complexes in organic media. Highlighted boxes are further analyzed in panel b. b) Average Δ*G*_int_ values measured for the alternative types of complexes in five solvents. c) Correlations between Δ*G*_charge_ or Δ*G*_pol_ and the solvent H-bond acceptor β parameter observed for **M6**/**M8** (left) and **M3**/**M9**/**M10** (right). d) Δ*G*_pol_ or Δ*G*_charge_ values measured with neutral **M3** and charged **M6**, respectively, in various organic media. e) Representation of initial vs equilibrium *Z*/*E* ratios measured in dichloromethane and acetonitrile with selected derivatives.

We next proceeded to dissect the stability of the complexes in organic solvents other than dichloromethane. Given the comprehensive character of our study, a reduced data set formed by representative models of each type was used for some solvents, being our selection guided both by chemical and experimental criteria (see the Supporting Information). Overall, this work implied an accurate evaluation of more than 180 interaction free energy values that allowed establishing a solvent-dependent hierarchy for the most relevant types of aromatic complexes (Figure 6a–e). For clarity, ionic and CH-polarized complexes have been linked to their corresponding reference isosteres with a vertical arrow, where its length is proportional to Δ*G*_charge_ or Δ*G*_pol_, respectively (see also Tables S1 and S2 and Figures S10–S15).

At first glance, we noticed that the dispersion of experimental free energies (Δ*G*_int_) became gradually narrower and more compact as the solvent polarity increased. This phenomenon mainly reflects the progressive destabilization of the cationic complexes, whose strength significantly diminishes in the most polar environments, even to the point of becoming destabilizing in extreme cases. Unlike contacts involving positively charged species, the stability of neutral CH/π complexes seems relatively insensitive to the polarity of the medium, whether they comprise polarized or nonpolarized CH functions. *This property determines a cutoff point in terms of polarity, above which the neutral complexes outcompete the charged ones.*Figure 6b shows the average interaction free energies deduced for the diverse aromatic contacts in five different solvents: while cationic fragments win in nonpolar media, such as dichloromethane or chloroform, this hierarchy is not sustained for all solvents assayed. Thus, in acetonitrile, the stability ranking overturns as follows: polarized-CH/π > cationic CH/π > nonpolarized CH/π > anionic CH/π. Moreover, in dimethyl sulfoxide the dominance of the neutral complexes becomes more pronounced, with the stability order as follows: polarized-CH/π > nonpolarized CH/π > cationic CH/π > anionic CH/π. In fact, the somewhat constant behavior of the polarized-CH/π type complex exemplified by **M3**, with free energy scores around −1.0 kcal/mol regardless of the medium, confers *CH-polarization a strategic value for the construction of receptors or catalysts with the potential to work in diverse, or even biphasic environments*. Notably, charge contributions to the obtained Δ*G*_int_ values (Δ*G*_charge_, represented in Figures S10 and included in Table S2) also decrease along with the solvent polarity, this being the main reason for the destabilization of the cation/π contacts. The observed reductions are especially pronounced for those interactions involving guanidinium functions, whose outstanding hydrogen bond donor character enables more efficient competition by the solvent. Indeed, Δ*G*_charge_ values derived for guanidinium-containing complexes **M9** and **M10** show an excellent correlation (*R*^2^ > 0.9) with the hydrogen-bonding acceptor properties of the solvent, as evaluated by the β parameter reported by Hunter and col. (Figure 6c).^43−45^ On the contrary, for poor hydrogen-bond donor cations, such as those present in models **M6** or **M8**, the observed correlations are worse (Figures 6c and S16). This divergent behavior exhibited by both cation types is epitomized in pyridine, one of the best hydrogen-bond acceptor solvents among those tested, where the presence of guanidinum groups ultimately determines unfavorable Δ*G*_charge_ contributions, in clear contrast with tetra-alkyl-ammonium fragments (Figure 6a and Table S2).

Regarding the aromatic complex formed by **M3**, stability enhancements promoted by the polarization of the interacting CH groups show a more limited solvent dependency, as revealed by the Δ*G*_pol_ parameter (Figures 6c,d and S16). Interestingly, the obtained values clearly surpass those observed for cationic model **M6** in various environments, such as nitromethane (Table S2) or acetonitrile (Figure 6d). Stability differences with respect to guanidinium-bearing complexes **M9** and **M10** are even larger and extend to most solvents. The better electrostatics of **M3** neutral complexes reflects the poor hydrogen-bonding character of the polarized CH functions toward classic acceptors, which provides an edge for aromatic surfaces and determines a more limited solvent competition.

Despite the few anionic examples contained in our data sets, unfavorable free energy contributions promoted by negatively charged fragments seem barely dependent on the polarity of the environment, as revealed by **M12** destabilizing charge contributions (Δ*G*_charge_), which are tantamount in both dichloromethane and acetonitrile. On the contrary, Δ*G*_charge_ values show a certain correlation (*R*^2^ > 0.7, Figure S16) with the solvent hydrogen-bonding donor properties, given by Hunter’s α parameter.^43^ This observation indicates that as previously observed for the positively charged complexes, stronger anion/solvent interactions also translate to weaker complexes.

Finally, from a methodological perspective, the *in situ* generation of molecular glyco-balances explored in this study has the potential to reveal not only the equilibrium population of the *Z*-enamine with respect to the *E*-enamine but also their relative formation rates. These kinetic parameters contain qualitative information about the influence exerted by the aromatic contacts on the incipient intermediates and/or transition states in the *Z*/*E* selectivity-determining steps (see Figure 4a). Thus, a situation where the aromatic complexes preferentially enrich hemiaminal/imine conformations favoring the *Z*-enamine could be envisaged (Figures 4 and S17). These putative contacts could exhibit interaction geometries related, but not identical, with those existing in the final *Z*-product, implying a multitude of potentially stabilizing scenarios (Figure S17).

Interestingly, both thermodynamic and kinetic parameters are somewhat correlated: stronger equilibrium complexes promote faster formation of the *Z* vs *E* derivatives, while weaker equilibrium interactions translate into more reduced initial (*t* = 0) *Z*/*E* ratios. To illustrate this point, Figures 6e and S18 show a representation of the observed *Z*/*E* ratios at *t* = 0 and ∞, in dichloromethane and acetonitrile. It can be observed that in dichloromethane, complexes involving tetra-alkyl-ammonium fragments are thermodynamically and kinetically favored, whereas in acetonitrile the polarized-CH/π complex formed by **M3** presents the highest *Z*/*E* ratios at both *t* = 0 and ∞. Similarly, the anionic fragment present in **M12** not only decreases the *Z*/*E* ratio at equilibrium but also exerts a very detrimental influence on this parameter at *t* = 0. In summary, the inchoate *Z*_0_/*E*_0_ ratios represent a complementary source of accessible information about the relative strength of the aromatic interactions in any given pair of enamines. Although still qualitative, this analysis becomes especially telling in those environments in which enamine formation and/or equilibration is particularly slow, such as DMSO, where the destabilizing influence exerted by **M6** positive charge on the stability of the aromatic complex is already apparent from the initial HSQC data set (Figure S19).

### Counterions and Aromatic Units: Optimizing the Ternary Complex

**Figure 7:**
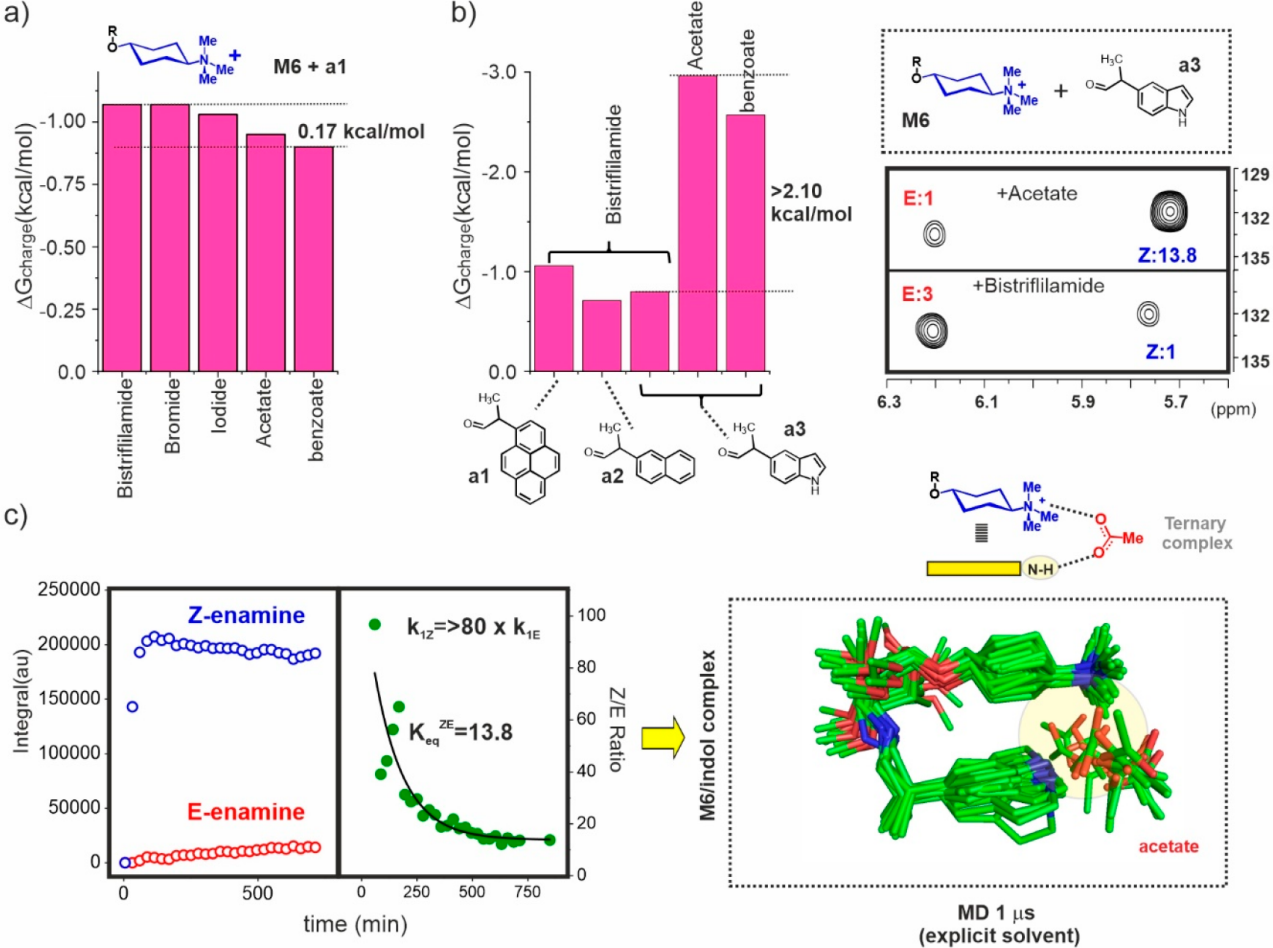
a) Δ*G*_charge_ values measured for **M6** in dichloromethane employing aldehyde **a1** and five counterions. b) Left: Δ*G*_charge_ measured for **M6** with aldehydes **a1**–**a3**. With aldehyde **a3**, the results obtained with three anionic species are shown. Right: Final HSQC data sets measured with pair **M6**/**a3** in the presence of acetate (top) or bistriflylimide (bottom). c) Left: Kinetic curves for *Z*- and *E*-enamine formation (left), together with the corresponding *Z*/*E* ratio (right). Right: Ternary indole/acetate/cation contacts as revealed by MD simulations (1 μs, **M6**, acetate 1:1 in explicit dichloromethane).

As a next step, we examined the influence counterions possess on the stability of the cationic complexes. For these assays, we focused exclusively on **M6** and selected a low polarity solvent such as dichloromethane, where the anion/cation interactions are stronger. Thus, **M6** charge contributions to stability (Δ*G*_charge_) were evaluated in the presence of five different anionic species including bistriflylimide, iodide, bromide, acetate, and benzoate. The obtained results are shown in Figure 7a (see also Figures S20–S22). In general, the effect associated with a change in counterion is minor in all cases. More specifically, the Δ*G*_charge_ value measured with bromide was virtually identical to that previously obtained with bistriflylimide. With iodide, a small reduction was detected, although the observed variation falls within experimental error. Alternatively, charge contributions seem somewhat reduced in the presence of bidentate anions such as acetate or benzoate. The observed variations amount to 0.12 kcal/mol for acetate and 0.17 kcal/mol for benzoate, which represent 11% and 16% of the Δ*G*_charge_ values measured with bistriflylimide, respectively. In short, even in low polarity environments, our experiments revealed a null to modest influence of counterions regardless of their structural or electronic nature. We then hypothesized that aromatic units equipped with hydrogen-bonding donor capacity, such as an indole, might display radically different behavior due to their ability to establish direct polar interactions with the anionic species. Indeed, these systems could participate in stable ternary aromatic/cation/anion complexes, with the anionic species acting as a hinge between the ammonium center and the heterocyclic aromatic platform.

In order to challenge this view, we first carried out reactions with models **M6** (+ bistriflylimide) and **R6**, employing aldehydes **a2** or **a3**, equipped with naphthyl and indole units, respectively. The obtained results are shown in Figure 7b, where it can be observed that **a2** yields a Δ*G*_charge_ value around 30% smaller than that previously derived with **a1**, echoing the reduced surface area of the naphthyl unit. Aromatic dimensions are further decreased in aldehyde **a3**. However, this factor seems partially counterbalanced by the electron-rich character of the indole platform, overall leading to a less dramatic reduction in Δ*G*_charge_. Strikingly, the replacement of bistriflylimide by acetate or benzoate resulted in an substantial strengthening of the aromatic complex formed by **M6**, evidenced by a significant shift in the enamine *Z*/*E* balance toward the *Z* derivative (Figure 7b,c). In addition, this *Z* stereoisomer was also kinetically favored, now forming around 2 orders of magnitude faster than the *E* derivative. On the contrary, the presence of carboxylate counterions influenced neither the *E*/*Z* balance nor the relative formation rates for the neutral reference system **R6**, a result that was replicated when the reactions were performed with the nalphthyl-containing aldehyde **a2**. Overall, the strengthening of the **M6**/indole complex promoted by switching from bistriflylimide to acetate or benzoate as counterions amounted to −2.16 and −1.77 kcal/mol, respectively, an unprecedented stabilization leap that mirrors the establishment of matching ternary contacts between the anion and both the cationic center and the heterocyclic aromatic platform. This view was also supported by molecular dynamics simulations, which correctly reproduced the proposed interaction motif (Figure 7c).

In summary, even in low polarity media, the modulation of the cation/π contacts mediated by counterions is, at best, modest. However, a careful selection of cation, anion, and aromatic partners can synergistically boost the stability through the formation of self-complementary ternary complexes.

## Discussion

Aromatic CH/π complexes in water have been extensively analyzed by the chemical community in recent years.^1−3^ In contrast, the structure/stability relationships that govern their formation in organic media have received much less attention. In particular, the relative energy strengths of these interactions and how they are modulated by the organic solvent remain elusive, despite a solvent-dependent hierarchy would be extremely helpful in selecting the best interacting partners to stabilize any given molecular architecture.

In principle, the change from water to an organic medium would present itself with three main consequences for the magnitude of the aromatic contacts: first, the solvophobic contributions will be greatly attenuated in a less cohesive environment. Second, electrostatic forces will be amplified as the dielectric constant of the new solvent decreases. Finally, charged complexes will exist either as close ion pairs or solvent-separated ion pairs but never as solvent-equilibrated ion pairs, since a complete solvolysis of the salt is unfeasible. Under these circumstances, counterions can also influence the stability of the aromatic contacts, from both a steric and electrostatic perspective. Accordingly, organic media can have a profound influence on the relative stabilities of both neutral and ionic aromatic interactions.

To tackle this issue, we have performed an extensive analysis of different CH/π contacts in which the interacting alkyl fragments are equipped with nonpolarizing, polarizing, cationic, or anionic functions. Our analysis covers the most relevant complex categories and involves more than 180 accurate free energy measurements. According to our results, the strongest interactions are those exhibiting cationic contacts in low polarity media. However, their free energy values are somewhat scattered, indicating that other significant factors, such as the geometry of the complex, might be at play as well. Remarkably, poor shape complementarity can be counterbalanced by the introduction of positive charges, this being a particularly resourceful stabilization strategy for less optimized interacting surfaces. However, as the solvent polarity increases, a progressive destabilization of the cationic interactions becomes apparent, a trend that is more pronounced for better hydrogen-bond donors due to their preference for establishing polar interactions with the solvent.

Conversely, neutral CH/π contacts showed a more constant stabilization across a whole range of environments. Among them, polarized CH functions established the strongest interactions, well above 1.0 kcal/mol in terms of free energy in most cases. Even in low polarity media, the stability provided by these contacts falls within the same range observed for cationic complexes and is maintained in more polar solvents such as acetonitrile. Therefore, CH-polarization strengthens aromatic complexes in a wide variety of environments, to the point of outperforming the stabilization caused by positive charges.

The relative strength of cationic and neutral aromatic complexes in water has been the subject of intense debate within the scientific community for years. In this regard, it is now commonly accepted that, regardless of their chemical nature, cationic functions tend to destabilize CH/π contacts, reflecting their preference for water.^25,26^ Interestingly, the results presented herein show that this behavior can also extend to organic solvents. For example, cationic fragments with good hydrogen bonding properties, (i.e., **M9** and **M10**) are destabilizing in organic environments with excellent hydrogen bond acceptor properties, such as pyridine or DMSO. Indeed, in this latter medium, even trimethylammonium moieties can be unfavorable (see, for example, the interaction free energies derived for pair **M6**/**R6**). As previously mentioned, this behavior resembles that of cation/π complexes in water and shows that in organic media positive charges can also present strong interactions with the solvent, and consequently be detrimental for aromatic complex formation.

Regarding the anionic fragments, they always lead to geometry-dependent repulsive forces with the electron-rich aromatic platforms employed in our study; however, their absolute value is moderate in comparison to the attractive interactions promoted by cationic centers.

Finally, even in apolar environments, counterions seem to exert mild influences, at best, on the free energy of association for cation/π and anion/π contacts. Strikingly though, particular combinations of aromatic units, cationic fragments, and anionic counterions may act synergistically to promote large stabilizations of the full aromatic complex. This observation can be explained by the establishment of matching ternary contacts with the counterion cross-linking both the cationic center and the aromatic unit, a combination responsible for free energy changes above 2.0 kcal/mol.

On the methodological side, a novel dynamic covalent strategy has been developed to quantitatively measure relative free energy values. Our approach employs a simple chemical reaction to generate the reporter molecular balance *in situ*. A careful design of the model amines and ^13^C-labeled aldehydes, favoring enamine formation over undesired side-reactions, enabled a quantitative NMR-monitoring of this process. By analysis of the resulting kinetic curves, the effect of the aromatic interactions on the relative enamine formation rates and equilibrium populations can be measured. Both data sets show some correlation, signaling that aromatic contacts also influence key steps of the reaction pathway leading to the Z stereoisomer. Contrary to equilibrium constants, initial *Z*/*E* ratios can be derived with relatively short experiments, and although qualitative in nature, they have diagnostic value, too. Overall, the initial (*t* = 0) and equilibrium (*t* = ∞) *Z*/*E* ratios afford common chemical conclusions, providing complementary information about the relative stability of the analyzed CH/π contacts.

## Conclusions

For the first time, a general solvent-dependent hierarchy for aromatic interactions was constructed and presented to the scientific community. This accomplishment has been achieved by carrying out a painstaking analysis of aromatic complexes in organic media using a novel strategy based on the *in situ* generation of isotopically labeled molecular glyco-balances with a dual kinetic/thermodynamic character. The work presented herein is of general applicability, since it encompasses a wide variety of organic environments and covers most of the conceivable aromatic complex categories. Moreover, alternative interaction geometries and counterions have been studied. Thus, by revealing the optimal interacting partners in every chemical environment, the design of strong *ad hoc* aromatic complexes can be achieved with potential applications in diverse fields related to supramolecular chemistry, from organocatalysis to material chemistry.

## Materials and Methods

A detailed description of the experimental and synthetic protocols and the characterization of products and intermediates are included in the Supporting Information.

### *In Situ* Generation of Molecular Balances: NMR Assays and Data Analysis

Reactions were performed in 5 mm NMR tubes. Aliquots of the different model or reference derivatives (5–10 μL) were added from stock solutions to NMR tubes previously loaded with 550 μL of deuterated solvent to give a final concentration in the 500–1000 μM range. In order to accelerate the formation of the enamine species, various Lewis or protic acids such as Bi(OTf)_3_, Sc(OTf)_3_ and trifluoroacetic acid (TFA), were tested. Best results were obtained by adding 0.05 equiv of TFA, conditions that were selected for the rest of our studies, unless otherwise stated. Enamine formation was initiated by the addition of a substoichiometric amount (0.3–0.5 equiv) of ^13^C-labeled-aldehydes **a0**–**a3** and monitored by sequential acquisition of HSQC experiments. These experiments were carefully optimized to allow for precise quantification of the relative *Z*/*E* enamine populations. Accordingly, the ^13^C offset was fixed at the midpoint of the *Z*/*E* enamine signals to ensure proper excitation of both species. Additionally, HSQC INEP modules were optimized for a ^1^*J*_CH_ heteronuclear coupling of 163 Hz, coincident with that previously determined for the enamine −N–^13^CH=C fragments. Finally, the effect of relaxation delay, *d*_1_, on the evaluation of the enamine populations was analyzed. These assays allowed us to confirm that this parameter does not have a relevant effect and thus was set to 1 s. All spectra were recorded on a Bruker AVIII 600 MHz spectrometer at 293 K. HSQC data sets were acquired on a 1024 × 256-point matrix, zero filled to 2000 × 2000, with 2–32 scans per increment, and the interpulse delay, corresponding to 1/4 *J*_CH_, set to 1.53 ms. Spectral widths in the proton and carbon dimensions were 10 and 200 ppm respectively.

Preliminary reactions with aldehyde **a0** (lacking an α-methyl group) in dichoromethane-*d*_2_ showed that the process is relatively fast, reaching an apparent equilibrium in less than 60 min. However, on longer time scales, the reaction mixture continues to evolve, leading to a progressive decrease in the intensity of the enamine cross-peaks, accompanied by the appearance of additional signals. Such evidence forced us to reconsider the chemical structure of the employed aldehyde. Since aldolic-type condensations involve the α-carbon, we hypothesized that these side-reactions could be selectively hindered by the incorporation of an α-methyl group. Indeed, reaction mixtures derived from **a1**–**a3** showed a much greater chemical stability, allowing for a complete monitorization of the enamine formation and equilibration process, albeit with slower reaction rates.

Enamine *Z*/*E* cross-peaks were integrated with Topspin Bruker software, and the obtained integrals, together with their corresponding ratios, were represented against the evolution time to generate kinetic curves, employing the program Origin pro 2018. *Z*/*E* population ratios displayed an exponential-like behavior, converging asymptotically to the *K*_eq_^**Z**/**E**^ equilibrium constant. These curves were approximated by the simple exponential function [*Z*]/[*E*] = *K*_eq_ + *A* e^–*t*/*c*^ to derive the enamine *Z*/*E* concentration ratios at *t* = ∞ (the *K*_eq_ equilibrium constant) and at *t* = 0 (equivalent to the ratio between kinetic constants ratios *k*_1*Z*_ and *k*_1*E*_ represented in Figures 4 and S6). In this way, *K*_eq_ equilibrium constants could be derived in a straightforward manner, even recurring, in particular cases, to a relatively reduced number of data points. Regarding the initial enamine concentration ratios, their measurement required initial HSQC spectra with excellent signal-to-noise ratio which sometimes forced us to reduce the fraction of TFA in the reaction media or even to carry out the reaction in the absence of catalyst, to permit a slower reaction progress and a more adequate monitoring of the initial stages. It should be mentioned that both final and initial *Z*/*E* concentration ratios were found relatively insensitive to the employed catalytic TFA (Figure S12). Examples of experimental curves measured with different model systems are represented in Figures S6 and S12–S15.

From the *K*_eq_^**Z**/**E**^ equilibrium constants the free energy differences between both enamine stereoisomers were derived using the expression: Δ*G*_*Z*/*E*_ = −*RT* ln *K*_eq_*.* Interaction free energies for the aromatic complexes (herein referred to as Δ*G*_int_) were obtained by subtracting the Δ*G*_*Z*/*E*_ values measured with the different models from that obtained for the reference derivative **R0**. Similarly, direct subtraction of the Δ*G*_*Z*/*E*_ values determined for the ionic derivatives and the corresponding neutral isosteric compounds (such as **M6** and **R6**) provided access to the contribution of the charges to the stability of the aromatic complexes (Δ*G*_charge_). Finally, subtraction of the Δ*G*_*Z*/*E*_ values determined for **M3** and **R3** provided information about the contribution of CH polarization to the strength of the CH/π contacts (herein referred to as Δ*G*_pol_). The obtained *K*_eq_^**Z**/**E**^*,* Δ*G*_*Z*/*E*_, Δ*G*_int_, and Δ*G*_charge_ or Δ*G*_pol_ values are represented in Tables S1 and S2.

Our studies were extended to a wide variety of solvents, selected to cover the broadest range of polarities possible. This selection was conditioned by two factors: the solubility of our systems (especially those charged) and their reactivity. Regarding the first factor, it imposes clear limitations in the low polarity range. Thus, initial assays indicated that charged models displayed insufficient solubility in carbon tetrachloride, benzene, or pure chloroform. Bearing these considerations in mind, we selected chloroform/dichloromethane mixtures as the lowest polarity environments being able to reduce the proportion of dichloromethane in the mixture to 10% in specific cases. Regarding the reactivity of the systems, our preliminary assays indicate that the formation/equilibration kinetics of the molecular balances are tremendously sensitive to the polarity of the medium. Additionally, we verified that in polar solvents, the efficiency of acid catalysis by TFA is significantly lower, sometimes forcing us to monitor the evolution of the reaction mixtures discontinuously, on a time scale of several days. Fortunately, the remarkable stability of the enamines generated with aldehyde **a2** allowed us to derive equilibrium constants without chemical quench being an issue (see Figure S11).

Reaction media included in our analysis are CDCl_3_/CD_2_Cl_2_ 90:10, CDCl_3_/CD_2_Cl_2_ 75:25, CD_2_Cl_2_, 1,4-dioxane-*d*_8_, nitrobenzene-*d*_5_, pyridine-*d*_5_, THF-*d*_8_, nitromethane-*d*_3_, acetonitrile-*d*_3_, DMSO-*d*_6_, and CD_3_OD (Figure 6). Given the extension of the proposed study, we employed the complete library of 3-amine-3-deoxy-allose derivatives only in dichloromethane-*d*_2_, restricting our analysis to representative sets of compounds in the rest of the environments. This selection was guided by reactivity criteria. More specifically, uncatalyzed reactions with anionic fragments **M11** and **M13** displayed a very slow kinetics in most media and consequently these derivatives, together with the corresponding neutral references (**R11**/**R13**) were tested only in dichloromethane-*d*_2_. Similarly, **R12** was excluded from our studies in pyridine-*d*_5_ and DMSO-*d*_6_ due to the impossibility of guaranteeing full protonation of the carboxylic function. Moreover, models **M4** and **M5** were found to be basically unreactive in THF-*d*_8_. Finally, in both CDCl_3_/CD_2_Cl_2_ 90:10 and CD_3_OD, our analysis was restricted to five representative derivatives, namely, **R0**, **R3**, **M3**, **R6**, and **M6**, attending to solubility reasons and slow reaction kinetics, respectively. Overall, our study implied accurate measurement of more than 180 free energy values covering more than 30 glyco-balances and 11 alternative environments.

Duplicate experiments allowed us to establish conservative error estimations for *K*_eq_*.* Thus, for *K*_eq_ values in the 1–5 range, errors were proven below 5%, leading maximum free energy variations inferior to ±0.03 kcal/mol, which were assumed for all values within this stability range. Similarly, for *K*_eq_ values in the 5–10 range, errors were estimated below 7.5%, leading to maximum free energy variations below ±0.05 kcal/mol. Equilibrium constants in the 10–15 comprised errors in the 10% range and maximum free energy changes below ±0.08 kcal/mol. Finally, for *K*_eq_ values larger than 15, a maximum 15% variation was estimated leading to uncertainties in Δ*G*_*Z*/*E*_ inferior to ±0.10 kcal/mol. This protocol was validated by performing three independent experiments for a reduced set of representative models (Table S7). Regarding the interaction free energies (Δ*G*_int_) and the charge or polarization contributions to stability (Δ*G*_charge_ or Δ*G*_pol_), errors were calculated from the corresponding Δ*G*_*Z*/*E*_ values according to the equation Error(Δ*G*) = (Error(Δ*G*_*Z*/*E*_(model)^2^+ Error(Δ*G*_*Z*/*E*_(reference)^2^)^1/2^.

### Numerical Simulations with the Program GEPASI

The enamine formation and equilibration process was modeled employing the biochemical kinetic simulator GEPASI.^42^ A simplified kinetic model for the reaction was assumed, comprising bimolecular formation of an hemiaminal/imine adduct followed by the subsequent unimolecular rearrangements to produce either the *Z*- or *E*-enamine products (Figures 4 and S6). Calculations were performed assuming 1 mM concentration for the 3-amine-3-deoxy-allose derivative and 0.5 mM for the aldehyde, in agreement with our experimental set up. Several equilibrium constants for the bimolecular (in the 1000–10000 M^–1^) and unimolecular steps (in the 1–20 range) were sampled so that simulations reproduce basic empirical observations such as the consumption of the aldehyde and marginal accumulation of the intermediate hemiaminal/imine adducts. Regarding the kinetic constants, these were adjusted to roughly reproduce reaction times observed in dichloromethane (in the 300–600 min range). These assays allowed us to differentiate between two regimes, depending on the relative rates of the bimolecular and unimolecular reactions. Thus, when adduct formation is fast relative to the following rearrangements, kinetic curves for *Z*- and *E*-enamines can exhibit different features, showing either a conventional exponential profile or a more complex shape characterized by the presence of a maximum followed by a slow concentration decrease toward the final equilibrium value (as illustrated in Figure S12). On the other hand, a gradual decrease in the first association rate leads to a normalization of the enamine kinetic profiles, which gradually approach an exponential behavior. Theoretical *Z*/*E* concentration ratios were represented vs time, and the resulting curves approximated by the simple exponential [*Z*]/[*E*] = *K*_eq_ + *A* e^–*t*/*c*^. While this provided satisfactory results in most cases, extensive simulations showed that for particular combinations of kinetic constants theoretical curves could be better fitted by a sum of exponentials such as [*Z*]/[*E*] = *K*_eq_ + *A*_1_ e^–*t*/*c*1^+ *A*_2_ e^–*t*/*c*2^ (see Figure S6), a situation that was not encountered in the analysis of the experimental curves. Fitted values for *K*_eq_ and *k*_1**Z**_/*k*_1**E**_ were compared to the actual theoretical values employed in the simulations to assess the uncertainty of this approximation.

### Molecular Dynamics (MD) Simulations

Parameters for carbohydrate derivatives were generated with the antechamber module of AMBER 18,^46^ using the general Amber force field (GAFF),^47^ with partial charges set to fit the electrostatic potential generated with HF/6-31G(d) by RESP.^48^ The charges are calculated according to the Merz–Singh–Kollman scheme using Gaussian 09.^49^ Each derivative was immersed in a water box with a 10 Å buffer of solvent molecules. For the cationic models, the system was neutralized by adding explicit bistriflylimide counterions. A two-stage geometry optimization approach was performed. The first stage minimizes only the positions of solvent molecules and ions, and the second stage is an unrestrained minimization of all of the atoms in the simulation cell. The systems were then gently heated by incrementing the temperature from 0 to 300 K under a constant pressure of 1 atm and under periodic boundary conditions. Harmonic restraints of 30 kcal·mol^–1^ were applied to the solute, and the Andersen temperature coupling scheme^18^ was used to control and equalize the temperature. The time step was kept at 1 fs during the heating stages, allowing potential inhomogeneities to self-adjust. Water molecules are treated with the SHAKE algorithm such that the angle between the hydrogen atoms is kept fixed. Long-range electrostatic effects are modeled using the Particle–Mesh–Ewald method.^50^ An 8 Å cutoff was applied to Lennard-Jones and electrostatic interactions. Each system was equilibrated for 2 ns with a 2 fs time step at a constant volume and temperature of 300 K. Production trajectories were then run for an additional 1.0 μs under the same simulation conditions.

## Supporting Information Available

Detailed description of the experimental methods and synthetic protocols together with the characterization of products and intermediates and copies of ^1^H and ^13^C NMR spectra; Figures S1–S22 with details of the NMR reactivity experiments and MD calculations (PDF)
