# Supplementary material for: Illuminating a Solvent-Dependent Hierarchy for Aromatic CH/π Complexes with Dynamic Covalent Glyco-Balances
Source: JACS Au. 2024 Jan 2;4(2):476–90. doi: 10.1021/jacsau.3c00592 (PMC10900200; doi:10.1021/jacsau.3c00592)

Supporting Information

For

Illuminating a Solvent-dependent Hierarchy for Aromatic CH/π Complexes with Dynamic Covalent Glyco-Balances

Laura Díaz-Casado,[a] Alejandro Villacampa,[a] Francisco Corzana,[b] Jesús Jiménez-Barbero,[c,d,e] Ana M. Gómez,[a] Andrés G. Santana,[f] Juan Luis Asensio[a]*

[a]Departamento de Química Bio-Orgánica, Instituto de Química Orgánica General (IQOG-CSIC), Consejo Superior de Investigaciones Científicas (CSIC), 28006 Madrid, Spain.

[b]Departamento de Química, Centro de Investigación en Síntesis Química, Universidad de La Rioja, 26006 Logroño, Spain

[c]CIC bioGUNE, Basque Researchand Technology Alliance (BRTA), 48170 Derio, Spain;

[d]Ikerbasque, Basque Foundation for Science,48009 Bilbao, Spain

[e]Centro de Investigación Biomédica En Red de Enfermedades Respiratorias, 28029 Madrid, Spain

[f]Department of Chemistry of Natural Products and Bioactive Synthetics. Instituto de Productos Naturales y Agrobiología (IPNA-CSIC), San Cristóbal de La Laguna, Santa Cruz de Tenerife, 38206, Spain.

Correspondence should be addressed to J.L.A.: juanluis.asensio@csic.es

CONTENTS

1.-Synthesis of 3-amine-3-deoxy-allose derivatives: experimental protocols and characterization of products and intermediates ---S3-S45

2.- Copies of the ^{1}H- and ^{13}C{1H}- NMR spectra of purified synthetic intermediates------------S46-S113

3.-References---S114-S115

4.-Tables S1-S7--S116-S124

5.-Supplementary Figures S1-S22--S125-S147

1. Synthesis of 3-amine-3-deoxy-allose derivatives: experimental protocols and characterization of products and intermediates.

1.1 General methods

All necessary solvents and reagents were purchased and used as received, unless otherwise indicated. To remove water from the starting compounds, an azeotropic distillation with toluene was employed. All moisture sensitive reactions were performed in dry flasks fitted with glass stoppers or rubber septa under a positive pressure of argon. Liquids and solutions sensitive to air and/or moisture were transferred using syringes or stainless-steel cannulas. To dry organic solutions, anhydrous $MgSO_4$ or Na_2SO_4 were used, and evaporation of solvents was performed under reduced pressure using a rotary evaporator. In general, reactions were carried out at room temperature unless otherwise indicated. Purification by flash column chromatography was performed on silica gel 230-400 (Merck). Monitoring of the reactions by thin layer chromatography (TLC) was performed using Kieselgel 60 F254 chromatographs. The spots were first observed under UV irradiation (254 nm) and then charred with a 5% H_2SO_4 solution in ethanol. ^{1}H- and ^{13}C-NMR spectra were recorded primarily in $CDCl_3$ at 300, 400, 500, 600, or 800 MHz and 75, 101, 126, 150, or 200 MHz, respectively. Chemical shifts were expressed in parts per million (δ scale) and referenced to the residual H signal from the solvent ($CHCl_3$: δ 7.26 ppm). Coupling constants (*J*) are given in Hz. All ^{13}C-NMR spectra presented are decoupled from protons. Mass spectra were recorded by direct injection with a precise mass Q-TOF LC/MS spectrometer equipped with an electrospray ion source in positive mode, and when specified in negative mode.

1.2 General Procedures

Procedure I: Methylation of Hydroxyl groups

To a solution of the alcohol (1.0 mmol) in dry DMF or dry THF, NaH (60%) (1.5 mmol per each hydroxyl) and MeI (2.0 mmol for each hydroxyl) were added at 0 ºC under inert atmosphere. The reaction mixture was stirred at room temperature until complete disappearance of the starting product, as observed by TLC. The reaction was then quenched by adding water and extracted with AcOEt. Finally, the organic phase was dried over anhydrous $MgSO_4$, the residue was concentrated in vacuo and purified by column chromatography on silica gel.

Procedure II: Benzylidenation of diols

To a solution of the alcohol (1.0 mmol) in dry ACN (10 mL/mmol) was added, under an argon atmosphere, anhydrous *p*-TsOH (0.1 mmol) and then the corresponding arylaldehyde dimethylacetal (1.2 mmol) also dissolved in anhydrous ACN (5 mL/mmol). The reaction was stirred at room temperature until complete conversion of the starting material (12 h), then the reaction mixture was neutralized with Et_3N, evaporated in vacuo and the resulting crude was purified by column chromatography on silica gel.

Procedure III: Ketone reduction

A) To a solution of the ketone (1.0 mmol) in dry Et_2O (12 mL/mmol), $LiAlH_4$ (1.0 mmol) also dissolved in Et_2O (1 mL/mmol) was added dropwise at 0 ºC. After observing the disappearance of the starting material by TLC, successive washings were carried out: first with water, then with an aqueous solution of NaOH (0.1M) and finally with water again. The organic phase was dried over anhydrous $MgSO_4$, filtered and evaporated, and the resulting crude was purified by column chromatography on silica gel.

B) To a solution of the ketone (1.0 mmol) in MeOH (50 mL/mmol), $NaBH_4$ (1.5 mmol) was added at 0 ºC (ice bath). The reaction mixture was stirred at room temperature until the starting product disappeared on TLC (ca. 1 h), at which time it was poured into aqueous HCl (1M) and extracted with AcOEt twice. The organic phase was washed with brine, dried over anhydrous $MgSO_4$, filtered and evaporated; the resulting crude was purified by silica gel column chromatography.

Procedure IV: O-benzoylation of alcohols

To a solution of the alcohol (1.0 mmol) in CH_2Cl_2 (20 mL/mmol) at 0 °C, dry pyridine (5 mL/mmol) was added, followed by benzoyl chloride (1.1 mmol/hydroxyl), and the resulting reaction mixture was stirred at room temperature overnight. The reaction was then cooled to 0 °C and the excess benzoyl chloride was consumed by adding MeOH, then it was concentrated under reduced pressure, re-dissolved in CH_2Cl_2 and consecutively washed with HCl (1M), $NaHCO_3$ (sat) and brine. The organic phase was dried over anhydrous $MgSO_4$, filtered and the solvent was removed under reduced pressure. The resulting crude was purified by column chromatography on silica gel.

Procedure V: Amine protection as trifluoroacetamides

To a solution of the amine (1.0 mmol) in MeOH (30 mL/mmol) ethyl trifluoroacetate (3 mmol/amine) was added, followed by Et_3N (3 mmol/amine). The reaction mixture was stirred at room temperature for

approximately one hour, or until complete disappearance of the starting material was observed by TLC. The solvent was then concentrated in vacuo, the crude re-dissolved in AcOEt and washed three times with HCl (1M). The organic phase was dried over anhydrous $MgSO_4$, filtered and evaporated to dryness, and the resulting crude was purified by column chromatography on silica gel.

Procedure VI. Zemplén deprotection of alcohols

To a solution of the ester (1.0 mmol) in MeOH (20 mL/mmol), a catalytic amount (10% w/w) of fresh NaOMe was added. The reaction mixture was stirred at room temperature until the complete disappearance of the starting material was observed by TLC. Subsequently, it was neutralized with amberlite IR 120, filtered and concentrated under reduced pressure.

Procedure VII: Thioglycoside glycosylation

The thiophenyl glycoside donor (1.0 mmol) and the corresponding acceptor alcohol (1.3 mmol) were combined in a flask and dried by azeotropic distillation with toluene under reduced pressure; then, activated 4 Å molecular sieves and NIS (1.2 mmol) were added. After purging with Ar several times, the solids were dissolved in a 3:1 mixture of anhydrous Et_2O and CH_2Cl_2 (30 mL/mmol). The mixture was stirred at room temperature under an argon atmosphere for 15 min, after which time it was cooled to -60 ºC. TMSOTf (0.7 mmol) was then added and the resulting mixture was stirred while allowing it to warm to -40 °C over a 60-120 min period, or until no remaining starting material was detected by TLC. The reaction mixture was filtered, diluted with more CH_2Cl_2 and washed with $NaHCO_3/Na_2S_2O_3$ solution. The organic phase was dried over anhydrous $MgSO_4$, concentrated in vacuo and purified by silica gel column chromatography.

Procedure VIII: Trichloroacetimidate glycosylation

To a solution of the trichloroacetimidate donor (1.2 mmol) and the acceptor alcohol (1.0 mmol) in a 3:1 mixture of anhydrous Et_2O and CH_2Cl_2 (50 mL/mmol), activated 4 Å molecular sieves were added. The mixture was stirred under an argon atmosphere at room temperature for 15 min, after which time it was cooled to -78 °C. TMSOTf (0.3 mmol) was then added and the resulting mixture was stirred while allowing it to warm to -65 °C for a 30-120 min period, or until no remaining starting material was detected by TLC. The reaction was quenched by the addition of Et_3N, diluted with CH_2Cl_2 (20 mL) and washed twice with water. The organic phase was dried over anhydrous $MgSO_4$, filtered, concentrated in vacuo and purified by silica gel column chromatography.

Procedure IX: Deprotection of trifluoroacetamides

To a solution of the corresponding trifluoroacetamide (1.0 mmol) in methanol (10 mL/mmol), K_2CO_3 (5 mmol) dissolved in H_2O (1.5 mL/mmol) was added, and the resulting reaction mixture was stirred at reflux until the complete disappearance of the product was observed by TLC. Then, it was concentrated under reduced pressure and the resulting crude was dry-loaded and purified by column chromatography on silica gel.

Procedure X: Reductive amination

To a solution of a primary amine (1.0 mmol) in MeOH (10 mL/mmol) 37% aqueous formaldehyde (3.0 mmol) was added, followed by $NaBH_3CN$ (3.0 mmol) and then AcOH (1.5 mmol). The reaction mixture was stirred at room temperature for 2 to 6 hours or until complete disappearance of the starting material was observed by TLC. In the case of a secondary amine, half of all the reagents were added: 37% aqueous formaldehyde (1.5 mmol), $NaBH_3CN$ (1.5 mmol) and AcOH (0.75 mmol). It was then concentrated under reduced pressure and the resulting crude was dry-loaded and purified by column chromatography on silica gel.

Procedure XI: Methylation of tertiary amines to form the corresponding ammonium salts

To a solution of the tertiary amine (1.0 mmol) in MeOH (5 mL/mmol) solid $NaHCO_3$ (4 mmol) was added, followed by MeI (3 mmol); the resulting reaction mixture was stirred at room temperature for 16-24 hours until the complete disappearance of the starting material was observed by TLC. The mixture was concentrated in vacuo and the crude was purified by column chromatography on silica gel (AcOEt/MeOH/NH_4OH 17:2:1).

Procedure XII: Staudinger reduction of azides

To a solution of the corresponding azide (1.0 mmol) in THF (10 mL/mmol), an aqueous NaOH solution (0.1 M) (2 mL/mmol) or just water (2 mL/mmol) was added, as indicated in each case, followed by PMe_3 (1.5 mmol/per azide, 1M in THF). The reaction mixture was stirred at the appropriate temperature for each derivative until no starting product was detected by TLC. The reaction mixture was evaporated and the dry crude purified by silica gel column chromatography.

For compounds incorporating an ammonium salt, the reduction conditions were more intense: these compounds (1.0 mmol) were dissolved in a 3:1 mixture of MeOH/NaOH (1M) (5 mL/mmol), followed by PMe_3 (1M, THF) (3 mmol). The reaction mixture was stirred at 65 ºC in a microwave reactor until

the disappearance of the starting product was observed by TLC, tipically 4-6 hours, at which point it was evaporated. Given the high polarity of these products, the crude was first washed with AcOEt (3 times) to remove the excess reagent and trimethylphosphine oxide. Finally, to take the desired compound out of the residue, the crude was extracted 4 times with a 9:1 $CHCl_3$/MeOH mixture, and this organic phase was evaporated under reduced pressure.

Procedure XIII: General anion exchange method

To a solution of the corresponding ammonium salt (1.0 mmol) in dH_2O (20 mL/mmol) lithium bistriflylimide (LiNTf2) (2.2 mmol) was added, and the reaction mixture was vigorously stirred at 90 °C for 1-16 hours. Then, the reaction mixture was evaporated, and the resulting crude was first washed with chloroform (3 times) and then with acetone (5 times). Each fraction was evaporated to dryness separately and its content confirmed by ^{1}H-NMR.

Procedure XIV: Guanidinylation

To a solution of the corresponding amine (1.0 mmol) dissolved in MeOH (5 mL/mmol), DIPEA (1.5 mL/mmol) was added, followed by the guanidinylating reagent 1,3-diBoc-2-(trifluoromethylsulfonyl)guanidine. The reaction mixture was stirred at room temperature overnight, or until the disappearance of the starting material was observed by TLC, evaporated to dryness and purified by column chromatography on silica gel.

Procedure XV: 2-Amino-Pyrimidine formation

To a solution of the corresponding amine (1.0 mmol) in dH_2O (5 mL/mmol) 2-chloropyrimidine (1.1 mmol) was added, followed by Et_3N (1.0 mmol). The reaction mixture was heated at 100 ºC in a microwave reactor (30 min-1 h) until the disappearance of the starting product was observed by TLC. Then the solvent was evaporated to dryness and the resulting crude was purified by silica gel column chromatography.

Procedure XVI: N- and O-acetylation

Acetic anhydride (2 mL) was added to a solution of an amine or an alcohol (1.0 mmol) in pyridine (3 mL) at 0 °C and the mixture was stirred at room temperature (approximately 3-5 h). Excess acetic anhydride was quenched by adding EtOH at 0 °C. The reaction mixture was then evaporated to dryness and the residue was subsequently dissolved in CH_2Cl_2 and sequentially washed with HCl (1M), sat.

$NaHCO_3$ and brine. The organic phase was dried over anhydrous $MgSO_4$, filtered and the solvent removed under reduced pressure. The resulting crude was purified by column chromatography on silica gel.

Procedure XVII: Potassium cyanide nucleophilic substitution

A solution of an aryl-methyl bromide (1.0 mmol) in anhydrous DMF (10 mL) was treated with KCN* (4.0 mmol) under an argon atmosphere. The reaction mixture was stirred at 45 °C until complete conversion of the starting material was observed by TLC (approximately 5 to 12 hours). The mixture was diluted with CH_2Cl_2, washed with sat. NH_4Cl solution and then with brine. The organic phase was dried over anhydrous Na_2SO_4, evaporated under reduced pressure and the resulting crude was purified by column chromatography on silica gel.

*In those cases where $K^{13}CN$ was used, 1.2 equivalents were added, which leads to increased reaction times.

Procedure XVIII: DIBAL-H nitrile reduction

A solution of the corresponding nitrile (1.0 mmol) in anhydrous CH_2Cl_2 (10 mL) was cooled to -78 °C under argon. Then, DIBAL-H (1M, THF) (2.5 mmol) was added dropwise. The reaction mixture was allowed to warm up to -50 °C for approximately 2-3 h. Once all the starting material has been transformed, AcOEt was added to destroy the excess of DIBAL-H. The reaction is then warmed to room temperature, diluted with CH_2Cl_2 and treated with a 1:1 mixture of $AcOH/H_2O$, then it was washed with sat. $NaHCO_3$ solution until neutrality and finally with brine. The organic phase was dried over anhydrous $MgSO_4$ and concentrated in vacuo. The obtained residue was purified by column on silica gel.

Procedure XIX: α-Methylation of benzylic nitriles

A solution of the corresponding nitrile (1.0 mmol) dissolved in anhydrous DMF (5 mL/mmol) under a strict argon atmosphere was cannulated onto another solution previously prepared of $Me_2NH{\cdot}BH_3$ (2.0 mmol) and ^tBuOK (3.0 mmol) in anhydrous DMF (5 mL/mmol), also under an inert atmosphere. The resulting reaction mixture was stirred at 80 ºC until a complete transformation of the starting material was observed (approximately 30 min-1h). Then, the reaction was cooled to room temperature, diluted with AcOEt and sequentially washed with water and brine. The organic phase was dried over $MgSO_4$

and the solvent was evaporated under reduced pressure. The resulting crude was purified by column chromatography on silica gel.

1.3 Synthesis of glycosyl donors

1.3.1 Synthesis of glycosyl donor D1

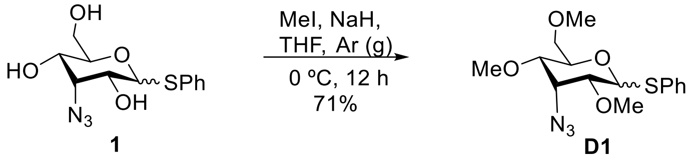

Scheme 1.1.*- Synthetic route for the preparation of 3-azido-3-deoxy* D*-allose derivative* ***D1****.*

Compound D1.

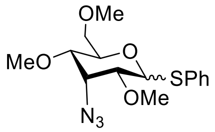

Compound **D1** was obtained following *general procedure I*, by reacting triol **1**[1] (2.24 g, 7.54 mmol) with NaH (60 %) (904 mg, 37.6 mmol) and MeI (1.9 mL, 30.2 mmol) in dry THF (38 mL). The reaction mixture was stirred at rt overnight. It was subsequently purified by column chromatography (Hex/AcOEt 95:5) to obtain donor **D1** (1.83 g, 71%) as a 1.1:1 α/β anomeric mixture. For anomer α: **^{1}H NMR** (400 MHz, $CDCl_3$) δ 7.53-7.49 (m, 3H), 7.34-7.14 (m, 2H), 5.57 (d, *J* = 5.8 Hz, 1H), 4.43 (t, *J* = 3.2 Hz, 1H), 4.28 (ddd, *J* = 9.8, 3.2, 2.1 Hz, 1H), 3.70 (dd, *J* = 10.7, 3.2 Hz, 1H), 3.69 (dd, *J* = 5.8, 3.2 Hz, 1H), 3.61 (dd, *J* =10.7, 2.1 Hz, 1H), 3.50 (dd, *J* = 9.8, 3.2 Hz, 1H), 3.46 (s, 3H), 3.45 (s, 3H), 3.39 (s, 3H); **^{13}C NMR** (101 MHz, $CDCl_3$) δ 137.4, 130.9 (2xC), 128.9 (2xC), 127.0, 86.7, 77.5, 75.1, 70.8, 67.2, 59.4, 58.9, 57.5, 57.2. **HRMS (ESI+) m/z** calc. for $C_{15}H_{21}N_3NaO_4S$ $[M+Na]^+$: 362.11450, found 362.11409. For anomer β: **^{1}H NMR** (400 MHz, $CDCl_3$) δ 7.55-7.51 (m, 2H), 7.32-7.24 (m, 3H), 4.88 (d, *J* = 9.6 Hz 1H), 4.49 (t, *J* = 3.2 Hz, 1H), 3.71 (ddd, *J* = 9.7, 4.0, 2.0 Hz, 1H), 3.61 (dd, *J* = 2.0, 10.9 Hz, 1H), 3.56 (dd, *J* = 4.0, 10.9 Hz, 1H), 3.48 (s, 3H), 3.44 (s, 3H), 3.38 (dd, *J* = 3.2, 9.7 Hz, 1H), 3.37 (s, 3H), 3.14 (dd, *J* = 9.6, 3.2 Hz, 1H); **^{13}C NMR** (101 MHz, $CDCl_3$) δ 133.2 , 132.5 (2xC), 128.9 (2xC), 127.7, 84.0, 78.7, 75.7, 75.2, 71.3, 59.6, 59.4, 58.1, 57.6. **HRMS (ESI+) m/z** calc. for $C_{15}H_{25}N_4O_4S$ $[M+NH_4]^+$: 357.1591, found 357.1585.

1.3.2 Synthesis of glycosyl donor D2

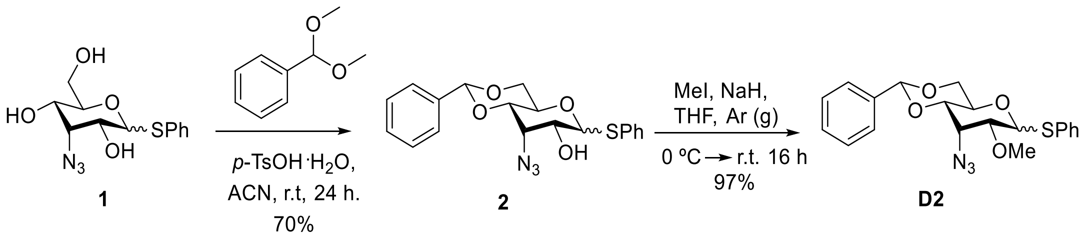

***Scheme 1.2.**- Synthetic route for the preparation of 3-azido-3-deoxy-D-allose derivative **D2**.*

Compound 2.

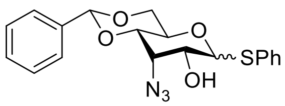

Compound **2** was prepared following *general procedure II*, starting from derivative **1** (1.55 g, 5.22 mmol). The reaction mixture was stirred at rt for 24 h, and the crude was purified by silica gel column chromatography (Hex/AcOEt 85:15) to give the desired compound **6** (α-anomer: 900 mg and β-anomer: 500mg, 70%). For anomer α: **^{1}H NMR** (300 MHz, $CDCl_3$) δ 7.55 – 7.48 (m, 4H), 7.44 – 7.36 (m, 3H), 7.36 – 7.27 (m, 3H), 5.59 (s, 1H), 5.43 (d, *J* = 5.8 Hz, 1H), 4.50 (td, *J* = 9.8, 5.1 Hz, 1H), 4.40 (dd, *J* = 10.3, 5.2 Hz, 1H), 4.31 (t, *J* = 3.3 Hz, 1H), 4.09 (ddd, *J* = 11.9, 5.9, 3.5 Hz, 1H), 3.85 – 3.71 (m, 2H), 2.82 (dd, *J* = 11.9, 1H). **^{13}C NMR** (75 MHz, $CDCl_3$) δ 136.7, 136.0, 131.5 (2xC), 129.2, 129.1 (2xC), 128.3 (2xC), 127.6, 126.1 (2xC), 101.9, 92.0, 77.7, 68.8, 67.6, 62.1, 59.9. **HRMS (ESI+) m/z** calc. for $C_{19}H_{19}N_3NaO_4S$ $[M+Na]^+$: 408.0988, found 408.0983.

Compound D2.

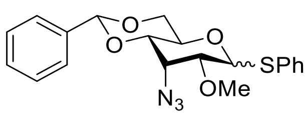

Compound **D2** was prepared following *general procedure I* for methylation of hydroxyl groups, starting with the α-anomer of compound **6** (900 mg, 2.33 mmol). The reaction mixture was stirred at 0 °C for 4 h under argon and the crude was purified by column chromatography on silica gel (Hex/AcOEt 8:2) to give compound **D2** (900 mg α-anomer, 97%). **^{1}H NMR** (300 MHz, $CDCl_3$) δ 7.60 – 7.46 (m, 4H), 7.46 – 7.35 (m, 3H), 7.35 – 7.27 (m, 3H), 5.58 (d, *J* = 4.5 Hz, 1H), 5.57 (s, 1H), 4.60 (td, *J* = 9.9, 5.2 Hz, 1H), 4.41 (t, *J* = 3.2 Hz, 1H), 4.36 (dd, *J* = 10.4, 5.2 Hz, 1H), 3.81 (dd, *J* = 4.5, 3.2 Hz, 1H), 3.75 (t, *J* = 10.3 Hz, 1H), 3.71 (dd, *J* = 8.4, 4.2 Hz, 1H), 3.52 (s, 3H). **^{13}C NMR** (75 MHz, $CDCl_3$) δ 136.7, 136.5, 131.1 (2xC), 129.2, 128.8 (2xC), 128.2 (2xC), 127.1, 126.1 (2xC), 101.9, 87.0, 77.3, 77.1, 68.8, 59.6, 59.0, 56.9. **HRMS (ESI+) m/z** calc. for $C_{20}H_{21}N_3NaO_4S$ $[M+Na]^+$: 422.1145, found 422.1159.

1.3.3 Synthesis of glycosyl donor D3

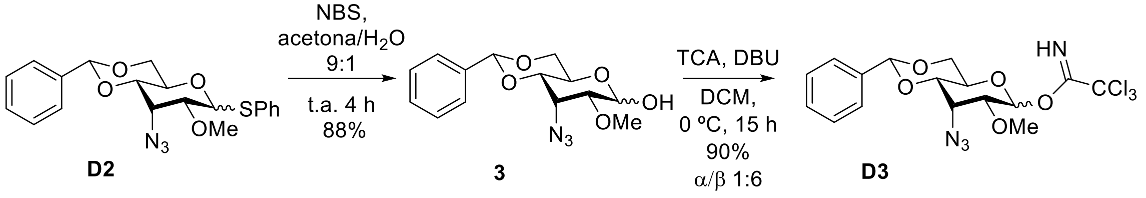

***Scheme 1.3.**- Synthetic route for the preparation of 3-azido-3-deoxy-D-allose derivative **D3**.*

Compound 3.

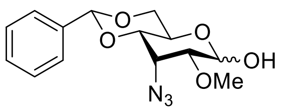

To a solution of derivative **D2** (160 mg, 0.40 mmol) in a (9:1) acetone/water mixture (13 mL) was added NBS (274 mg, 3.8 mmol). The reaction mixture was stirred at 0 °C for 4 h, then the solvent was removed under reduced pressure. The resulting crude was redissolved in AcOEt and washed with a saturated solution of $Na_2S_2O_3$ and subsequently with $NaHCO_3$. The organic phase was dried over anhydrous $MgSO_4$ and then filtered and evaporated. The crude was purified through a silica gel column chromatography (Hex/AcOEt 6:4) to obtain compound **7** (110 mg, 88%) as a 1:6 α/β anomeric mixture. The β anomer, which is the predominant one, is described. **^{1}H NMR** (300 MHz, $CDCl_3$) δ 7.55 – 7.44 (m, 2H), 7.44 – 7.30 (m, 3H), 5.52 (s, 1H), 4.96 (d, J = 7.7 Hz, 1H), 4.79 (br. s, 1H), 4.40 (t, J = 3.2 Hz, 1H), 4.33 (dd, J = 10.4, 5.0 Hz, 1H), 3.94 (td, J = 9.7, 5.0 Hz, 1H), 3.71 (t, J = 10.8, 10.3 Hz, 1H), 3.65 (dd, J = 9.5, 3.0 Hz, 1H), 3.55 (s, 3H), 3.19 (dd, J = 7.6, 3.4 Hz, 1H). **^{13}C NMR** (75 MHz, $CDCl_3$) δ 136.6, 129.2, 128.2 (2xC), 126.0 (2xC), 101.8, 94.4, 80.2, 77.2, 68.7, 63.8, 59.7, 58.3. **HRMS (ESI+) m/z** calc. for $C_{14}H_{17}N_3NaO_5$ $[M+Na]^+$: 330.1060, found 330.1062.

Compound D3.

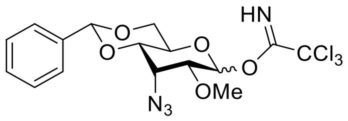

To a solution of hemiacetal **7** (110 mg, 0.36 mmol) in anhydrous CH_2Cl_2 (10 mL/mmol), at 0 ºC under an argon atmosphere, trichloroacetonitrile (0.43 mL, 4.32 mmol) and DBU (14 µL, 0.09 mmol) were sequentially added, and the resulting mixture was left to react for 15 h at 0 °C. Then it was evaporated under reduced pressure and NMR analysis of the reaction crude showed that an α/β = 1/6 mixture was obtained. Purification was carried out by silica gel column chromatography (Hex/AcOEt, 9:1 to 8:2) to obtain compound **D3** (145 mg, 90%) with was used in the next reaction without further purification.

1.4 Synthesis of glycosyl acceptors

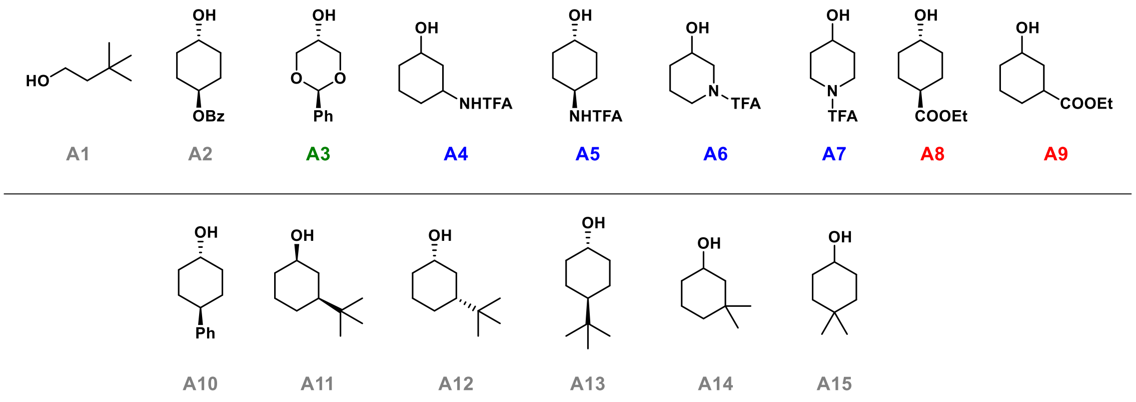

***Table 1.-** Glycosyl acceptors employed in this study.*

Glycosyl acceptors **A1**, **A3**, **A8**, **A9**, **A13**, **A15** are commercially available and were used as such without prior purification. Glycosyl acceptors **A5**,[2] **A6**,[3] **A7**,[4] **A10**,[5] **A11**,[6] **A12**,[6] and **A14**[7] were prepared according to synthetic procedures already published and their spectroscopic characterization was in agreement with reported values.

Compound A2.

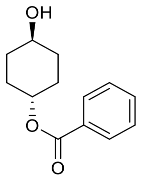

Starting from commercially available trans-1,4-cyclohexanediol (700 mg, 6.03 mmol), *general procedure IV* was followed. The resulting crude was purified by silica gel column chromatography (CH_2Cl_2/MeOH 9:1) to give compound **A8** (920 mg, 69%). **^{1}H NMR** (500 MHz, $CDCl_3$) δ 7.27 (d, J = 4.4 Hz, 3H), 7.19 (s, 2H), 4.47 (s, 1H), 3.64 (tt, J = 9.5, 4.1 Hz, 1H), 3.32 (tt, J = 9.7, 4.0 Hz, 1H), 2.04 – 1.96 (m, 2H), 1.92 (dddt, J = 10.3, 6.1, 2.8, 1.5 Hz, 2H), 1.51 – 1.42 (m, 2H), 1.41 – 1.18 (m, 2H). **^{13}C NMR** (126 MHz, $CDCl_3$) δ 138.9, 128.4 (2xC), 127.5 (2xC), 127.5, 86.2, 70.2, 69.7, 32.7 (2xC), 29.3 (2xC). **HRMS (ESI+) m/z:** calc. for $C_{13}H_{16}O_3NNa$ $[M+Na]^+$: 243.1205, found 243.1214.

Compounds A4a and A4b.

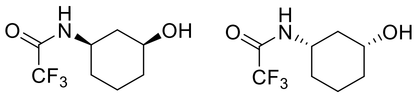

A4a A4b

First, starting from a solution of commercially available (*rac*)-3-aminocyclohexanol (575 mg, 5.0 mmol) in MeOH (50 mL, 10 mL/mmol), *general procedure V* was followed. The crude was purified by column chromatography on silica gel (Hex/AcOEt 7:3) to give the desired

trifluoroacetamides (700 mg, 66%) as a *cis/trans* mixture. The spectroscopic data obtained agreed with those published in the bibliography.[4]

Free alcohol protection was then carried out; for this, the previous compound (700 mg, 3.31 mmol) was dissolved in CH_2Cl_2 (16.5 mL, 5 mL/mmol) and at 0 °C trifluoroacetic anhydride (0.25 mL, 3.31 mmol) was added dropwise. The resulting solution was stirred at room temperature for 5 h and after completion, it was evaporated to dryness. Subsequently, the *cis* mixture (410 mg, 49%) was carefully separated from the *trans* by careful silica gel column chromatography (Hex/AcOEt 9:1), yielding the desired *cis* compounds (410 mg, 49 %).

Finally, from the *cis* mixture (410 mg, 1.3 mmol) *general procedure VI* was followed, whereby the corresponding trifluoroacetates were dissolved in methanol (13 mL, 10 mL/mmol) and a catalytic amount of freshly prepared MeONa is added to the mixture at 0 ºC. The resulting crude material was purified by chromatography on a silica gel column (Hex/AcOEt 7:3) to give compound **A4** as a mixture of *cis* products (230mg, 82%). **HRMS (ESI+) m/z** calc. for $C_8H_{13}F_3N_2O_2$ $[M+H]^+$: 212.0893, found 212.0883.

1.5 Synthesis of model systems M1-M13 (and R11-R13)

Compound 4α.

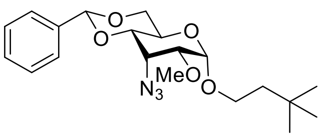

Donor **D3** (145 mg, 0.32 mmol) and commercial acceptor **A1** (36 mg, 0.35 mmol) were reacted following *general procedure VIII*. The crude was purified by silica gel column chromatography (Hex/AcOEt 9:1 to 8:2) to give compound **4α** (α-anomer: 66 mg, 53%; α/β ratio 5:1). **^{1}H NMR** (300 MHz, $CDCl_3$) δ 7.59 – 7.44 (m, 2H), 7.44 – 7.30 (m, 3H), 5.54 (s, 1H), 4.93 (d, *J* = 4.0 Hz, 1H), 4.40 (t, *J* = 3.5 Hz, 1H), 4.32 (dd, *J* = 10.3, 5.2 Hz, 1H), 4.19 (td, *J* = 9.8, 5.2 Hz, 1H), 3.78 (td, *J* = 9.4, 6.4 Hz, 1H), 3.69 (t, *J* = 10.3 Hz, 1H), 3.63 (dd, *J* = 9.4, 3.3 Hz, 1H), 3.54 (td, *J* = 9.5, 6.3 Hz, 1H), 3.48 (s, 4H), 1.73 – 1.56 (m, 2H), 0.94 (s, 9H). **^{13}C NMR** (75 MHz, $CDCl_3$) δ 136.8, 129.2, 128.3 (2xC), 126.1 (2xC), 101.8, 96.7, 77.7, 77.1, 69.1, 66.3, 58.3, 57.6, 57.0, 42.6, 29.6 (3xC), 29.5. **HRMS (ESI+) m/z** calc. for $C_{20}H_{29}N_3NaO_5$ $[M+Na]^+$: 414.1999, found 414.1986.

Compound 5.

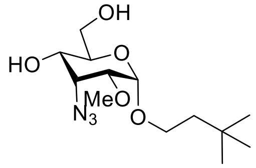

A solution of compound **4α** (66 mg, 0.17 mmol) in MeOH (4 mL) was treated with *p*-TsOH·H_2O, the resulting reaction mixture was stirred at rt for 5 h and then quenched by adding a few drops of conc. aq. NH_4OH. The mixture was evaporated to dryness and the crude was used in the next reaction without purification.

Compound 6.

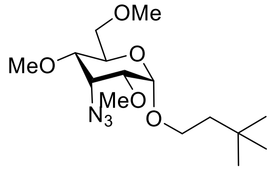

To a solution of crude **5** (51 mg, 0.17 mmol) in THF (1.7 mL, 10 mL/mmol) a methylation reaction was carried out following the *general procedure I*. The crude was purified by silica gel column chromatography (Hex/AcOEt, 3:1) to obtain derivative **6** (42 mg, 75%). **^{1}H NMR** (400 MHz, $CDCl_3$) δ 4.93 (d, *J* = 3.9 Hz, 1H), 4.43 (t, *J* = 3.6 Hz, 1H), 3.93 (ddd, *J* = 9.8, 3.4, 2.3 Hz, 1H), 3.76 (td, *J* = 9.4, 6.3 Hz, 1H), 3.64 (dd, *J* = 10.5, 3.4 Hz, 1H), 3.57 (dd, *J* = 10.5, 2.2 Hz, 1H), 3.50 (td, *J* = 9.4, 6.2 Hz, 1H), 3.45 (s, 3H), 3.43 (s, 3H), 3.41 – 3.36 (m, 2H), 3.39 (s, 3H), 1.69 – 1.54 (m, 2H), 0.91 (s, 9H). **^{13}C NMR** (75 MHz, $CDCl_3$) δ 96.1, 77.2, 75.3, 70.7, 65.9, 65.8, 59.3, 57.1, 56.9, 56.8, 42.6, 29.6 (3xC), 29.5. **HRMS (ESI+) m/z** calc. for $C_{15}H_{29}N_3NaO_5$ $[M+Na]^+$: 354.1999, found 354.1993.

Compound M1.

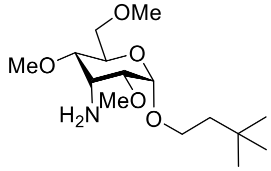

A solution of derivative **6** (25 mg, 0.08 mmol) in THF (1 mL) was treated according to *general procedure XIII*. The crude was purified by silica gel column chromatography (CH_2Cl_2/MeOH, 9:1) to give compound **M1** (21 mg, 91%). **^{1}H NMR** (500 MHz, CD_3OD) δ 4.89 (d, *J* = 3.8 Hz, 1H), 3.86 – 3.76 (m, 2H), 3.73 (t, *J* = 4.0 Hz, 1H), 3.63 (dd, *J* = 10.8, 2.2 Hz, 1H), 3.59 (dd, *J* = 10.8, 5.0 Hz, 1H), 3.49 – 3.45 (m, 1H), 3.43 (s, 3H), 3.39 (s, 3H), 3.37 (s, 3H), 3.33 (t, *J* = 3.9 Hz, 1H), 3.22 (dd, *J* = 10.2, 3.9 Hz, 1H), 1.64 – 1.51 (m, 2H), 0.95 (s, 9H). **^{13}C NMR** (126 MHz, CD_3OD) δ 98.7, 77.7, 77.0, 73.0, 67.1, 66.7, 59.6, 56.9, 56.8, 48.3, 44.0, 30.5, 30.1 (3xC). **HRMS (ESI+) m/z** calc. for $C_{15}H_{32}NO_5$ $[M+H]^+$: 306.2275, found 306.2272.

Compound 7α.

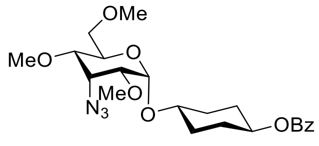

Compound **7α** was obtained following *general procedure VII*, starting from donor **D1** (340 mg, 1.0 mmol) and acceptor **A2** (224 mg, 1.3 mmol). The

crude was purified by column chromatography on silica gel (Hex/AcOEt 7:3) to give compound **7α** (150 mg, 37%; α/β ratio 1.3:1). **^{1}H NMR** (400 MHz, $CDCl_3$) δ 8.01 (dd, *J* = 7.9, 1.2 Hz, 2H), 7.52 (td, *J* = 7.1, 1.5 Hz, 1H), 7.41 (t, *J* = 7.7 Hz, 2H), 5.09 (d, *J* = 3.8 Hz, 1H), 5.08 – 5.03(m, 1H), 4.46 (t, *J* = 3.5 Hz, 1H), 4.00 (dt, *J* = 9.8, 2.9 Hz, 1H), 3.77 (tt, *J* = 7.4, 3.4 Hz, 1H), 3.63 (dd, *J* = 10.6, 3.4 Hz, 1H), 3.56 (dd, *J* = 10.3, 2.0 Hz, 1H), 3.44 (s, 3H), 3.42 (s, 3H), 3.40 (t, *J* = 3.8 Hz, 1H), 3.38 (s, 3H), 3.37 – 3.34 (m, 1H), 2.13 (dp, *J* = 11.8, 3.5 Hz, 2H), 2.00 (tdd, *J* = 12.5, 5.1, 2.8 Hz, 2H), 1.78 – 1.56 (m, 4H).**^{13}C NMR** (101 MHz, $CDCl_3$) δ 166.0, 132.8, 130.9, 130.2, 129.6, 128.6, 128.4, 94.7, 77.8, 75.2, 74.2, 71.9, 70.9, 66.2, 59.4, 57.1, 57.0, 56.5, 29.2, 27.8, 27.5, 27.4. **HRMS (ESI+) m/z** calc. for $C_{22}H_{32}N_3O_7$ $[M+H]^+$: 450.22348, found 450.22337.

Compound 8.

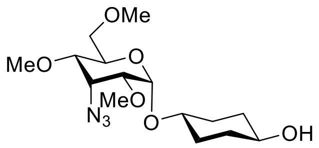

Compound **8** was prepared from a solution of **7α** (220 mg, 0.5 mmol) in MeOH (12.5 mL, 25 mL/mmol) following *general procedure VI*. The crude was purified by silica gel column chromatography (Hex/AcOEt 4:6) to yield derivative **8** (135 mg, 79%). **^{1}H NMR** (500 MHz, $CDCl_3$) δ 5.06 (d, *J* = 3.9 Hz, 1H), 4.42 (t, *J* = 3.6 Hz, 1H), 3.97 (ddd, *J* = 9.8, 3.3, 2.2 Hz, 1H), 3.69 (tt, *J* = 9.4, 3.5 Hz, 1H), 3.64 – 3.57 (m, 2H), 3.55 (dd, *J* = 10.5, 2.1 Hz, 1H), 3.44 (s, 3H), 3.43 – 3.39 (m, 4H) 3.38 (s, 3H), 3.35 (dd, *J* = 9.7, 3.5 Hz, 1H), 2.01 – 1.93 (m, 2H), 1.55 (dt, *J* = 11.7, 9.6 Hz, 1H), 1.49 – 1.41 (m, 3H), 1.39 – 1.27 (m, 2H). **^{13}C NMR** (126 MHz, $CDCl_3$) δ 94.4, 77.4, 75.2, 75.1, 70.7, 69.1, 65.9, 59.3, 57.1, 56.9, 56.6, 32.4, 32.1, 30.1, 28.2. **HRMS (ESI+) m/z** calc. for $C_{15}H_{28}N_3O_6$ $[M+H]^+$: 346.1899, found 346.1896.

Compound 9.

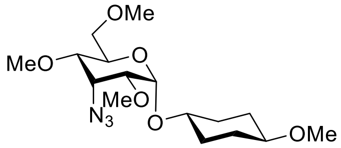

With a solution of crude **8** (135 mg, 0.4 mmol) in THF (6 mL, 15 mL/mmol) a methylation reaction was carried out following the *general procedure I*. The resulting crude material was employed in the next step without further purification.

Compound M2.

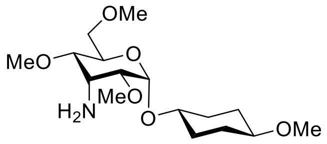

A solution of the previous crude azide (129 mg, 0.36 mmol) in THF (4 mL) was treated according to *general procedure XIII*. The crude was purified by silica gel column chromatography (CH_2Cl_2/MeOH, 9:1) to give compound **M2** (99 mg, 74%, 2 steps). **^{1}H NMR** (400 MHz, $CDCl_3$) δ 5.05 (d, *J* = 3.7 Hz, 1H), 3.84 (dt, *J* = 10.2,

3.1 Hz, 1H), 3.70 (d, *J* = 4.1 Hz, 1H), 3.66 – 3.57 (m, 3H), 3.41 (s, 3H), 3.38 (s, 3H), 3.37 (s, 3H), 3.30 (s, 3H), 3.27 – 3.20 (m, 2H), 3.16 (tq, *J* = 10.7, 3.5 Hz, 1H), 2.13 – 1.89 (m, 4H), 1.52 – 1.20 (m, 4H). **^{13}C NMR** (101 MHz, $CDCl_3$) δ 95.7, 77.6, 76.8, 75.8, 75.1, 71.4, 65.2, 59.3, 56.5, 56.5, 55.9, 47.9, 30.1, 28.7, 28.3, 28.0. **HRMS (ESI+) m/z** calc. for $C_{16}H_{32}NO_6$ $[M+H]^+$: 334.2224, found 334.2222.

Compound 10α.

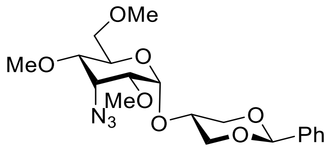

Compound **10α** was obtained from donor **D1** (200 mg, 0.6 mmol) and acceptor **A3** (137 mg, 0.8 mmol) following *general procedure VII*. The crude was purified by column chromatography on silica gel (Hex/AcOEt 7:3) to give compound **10α** (34 mg, 15%; α/β ratio 1.4:1). **^{1}H NMR** (400 MHz, $CDCl_3$) δ 7.45 (dd, *J* = 7.8, 1.9 Hz, 2H), 7.38 – 7.27 (m, 3H), 5.40 (s, 1H), 5.05 (d, *J* = 3.9 Hz, 1H), 4.46 – 4.39 (m, 2H, H-3), 4.36 (ddd, *J* = 11.0, 5.2, 2.3 Hz, 1H), 3.98 – 3.89 (m, 2H, H-5), 3.76 (td, *J* = 10.4, 3.4 Hz, 2H), 3.67 – 3.61 (m, 1H), 3.56 (dd, *J* = 10.6, 2.2 Hz, 1H), 3.44 (s, 3H), 3.43 (s, 3H), 3.43 – 3.39 (m, 1H), 3.39 (s, 3H), 3.38 – 3.35 (m, 1H). **^{13}C NMR** (101 MHz, $CDCl_3$) δ 137.7, 129.1, 128.4, 128.3 (2xC), 126.2 (2xC), 101.3, 96.3, 75.1, 70.8, 70.7, 69.6, 68.1, 66.6, 59.5, 57.3, 57.1, 56.6. **HRMS (ESI+) m/z** calc. for $C_{19}H_{28}N_3O_7$ $[M+H]^+$: 410.1922, found 410.1919.

Compound M3.

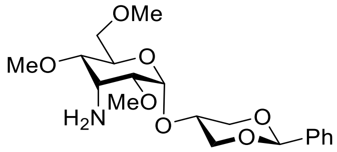

A solution of derivative **10α** (34 mg, 0.08 mmol) in THF (1.6 mL, 20 mL/mmol) was treated according to *general procedure XIII*. The crude was purified by silica gel column chromatography (CH_2Cl_2/MeOH, 9:1) to give compound **M3** (29 mg, 93%). **^{1}H NMR** (400 MHz, $CDCl_3$) δ 7.47 – 7.42 (m, 2H), 7.38 – 7.31 (m, 3H), 5.38 (s, 1H), 5.04 (d, *J* = 3.8 Hz, 1H), 4.44 (ddd, *J* = 10.9, 5.2, 2.2 Hz, 1H), 4.36 (ddd, *J* = 11.2, 5.2, 2.2 Hz, 1H), 3.94 (tt, *J* = 10.2, 5.1 Hz, 1H), 3.83 (dt, *J* = 10.1, 3.0 Hz, 1H), 3.75 – 3.66 (m, 3H), 3.63 – 3.58 (m, 2H), 3.42 (s, 3H), 3.40 (s, 3H), 3.38 (s, 3H), 3.27 (t, *J* = 4.0 Hz, 1H), 3.22 (dd, *J* = 10.1, 3.8 Hz, 1H). **^{13}C NMR** (101 MHz, $CDCl_3$) δ 137.6, 129.1, 128.4 (2xC), 126.2 (2xC), 101.4, 98.0, 76.8, 75.7, 71.3, 70.8, 69.6, 68.4, 65.7, 59.5, 56.6 (2xC), 47.5. **HRMS (ESI+) m/z** calc. for $C_{19}H_{30}NO_7$ $[M+H]^+$: 384.2017, found 384.2017.

Compound 11α (11α-a and 11α-b).

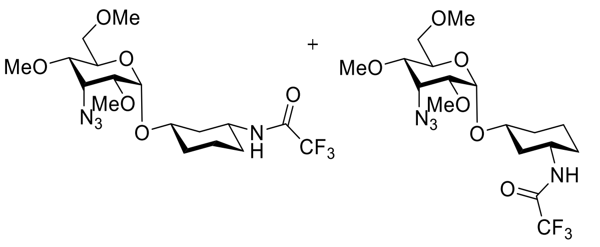

Compound **11α** was obtained by reaction of donor **D1** (650 mg, 1.91 mmol) and acceptor **A4** (485 mg, 2.3 mmol) following *general procedure VII*. The crude was purified by column chromatography on silica gel (Hex/AcOEt 7:3) to give the mixture of **11α** diastereoisomers (α-anomer: 195 mg, 30%; α/β ratio 1:1). The glycosylation products could not be separated in this step. **HRMS (ESI+) m/z** calc. for $C_{17}H_{31}F_3N_5O_6$ $[M+NH_4]^+$: 458.2221, found 458.2210.

Compound 12.

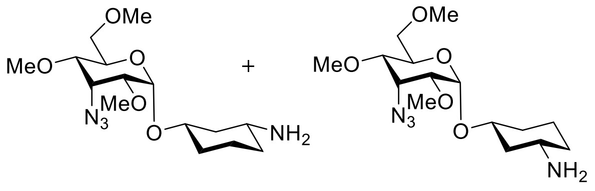

Starting with compound **11α** (320 mg, 0.72 mmol), *general procedure IX* was followed. The crude was purified by silica gel column chromatography (CH_2Cl_2/MeOH, 8:2) to give compound **12** (210 mg, 84%). A mixture of two diastereoisomers (**12a** and **12b**) was obtained, from which a small amount of the less polar one could be isolated and used for characterization. **^{1}H NMR** (500 MHz, CD_3OD) δ 5.02 (dd, *J* = 3.9, 0.7 Hz, 1H), 4.61 (t, *J* = 3.5 Hz, 1H), 4.09 (t, *J* = 3.3 Hz, 1H), 3.92 (ddd, *J* = 9.9, 5.3, 2.0 Hz, 1H), 3.60 (dd, *J* = 10.7, 2.0 Hz, 1H), 3.53 (dd, *J* = 10.7, 5.2 Hz, 1H), 3.49 – 3.47 (m, 2H, H-2), 3.46 (s, 3H), 3.40 (s, 3H), 3.36 (s, 3H), 3.30 – 3.26 (m, 1H), 2.18 (ddt, *J* = 10.0, 3.9, 2.0 Hz, 1H), 2.04 – 1.96 (m, 1H), 1.96 – 1.83 (m, 2H), 1.63 (dt, *J* = 13.9, 3.6 Hz, 1H), 1.54 (ddd, *J* = 12.9, 11.8, 2.6 Hz, 1H), 1.43 – 1.29 (m, 2H). **^{13}C NMR** (101 MHz, CD_3OD) δ 94.4, 77.5, 75.0, 71.8, 71.4, 66.6, 58.2, 56.1, 55.9, 55.5, 46.3, 36.4, 30.6, 27.6, 18.4. **HRMS (ESI+) m/z**: calc. for $C_{15}H_{29}N_4O_5$ $[M+H]^+$: 345.2132, found 345.2119.

Compounds 13 and 14.

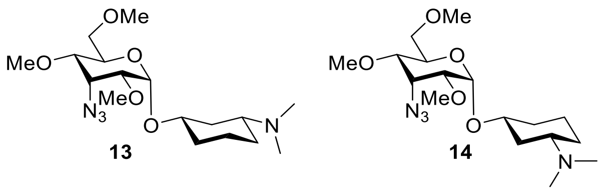

Compounds **13** and **14** were obtained from a solution of compound **12** (60 mg, 0.2 mmol) in MeOH (2 mL), following *general procedure X*. The crude was purified by silica gel column chromatography (CH_2Cl_2/MeOH, 9:1). In this step, both diastereoisomers could be separated, giving rise to compounds **13** (30 mg, 46%) and **14** (31 mg, 48%).

Compound **13**: **^{1}H NMR** (400 MHz, CD_3OD) δ 5.04 (d, *J* = 4.0 Hz, 1H), 4.51 (t, *J* = 3.7 Hz, 1H), 3.93 (ddd, *J* = 10.1, 4.8, 2.3 Hz, 1H), 3.59 – 3.49 (m, 3H), 3.43 (s, 3H), 3.41 (dd, *J* = 3.7, 0.9 Hz, 1H), 3.39 (s, 3H), 3.34 (s, 3H), 3.32 (d, *J* = 3.7 Hz, 1H), 2.35 (td, *J* = 11.4, 5.7 Hz, 1H), 2.28 (s, 6H), 2.25 (d, *J* = 10.4 Hz, 1H) 2.06 – 1.96 (m, 1H), 1.81 (dt, *J* = 10.0, 5.2 Hz, 2H), 1.39 – 1.09 (m, 4H). **^{13}C NMR** (101 MHz, CD_3OD) δ 94.6, 76.8, 76.5, 75.5, 71.4, 66.2, 62.1, 58.3, 56.8, 55.8 (2xC), 40.1 (2xC), 35.2, 31.1, 27.1, 21.7. **HRMS (ESI+) m/z** calc. for $C_{17}H_{33}N_4O_5$ $[M+H]^+$: 373.2445, found 373.2447.

Compound **14**: **^{1}H NMR** (400 MHz, CD_3OD) δ 5.01 (d, *J* = 4.0 Hz, 1H), 4.50 (t, *J* = 3.7 Hz, 1H), 3.91 – 3.81 (m, 1H), 3.60 – 3.46 (m, 3H), 3.41 (s, 3H), 3.38 (t, *J* = 4.0 Hz, 1H), 3.36 (s, 3H), 3.31 (s, 3H), 3.30 – 3.27 (m, 1H), 2.45 – 2.34 (m, 1H), 2.31 (s, 6H), 2.17 (d, *J* = 12.1 Hz, 1H), 1.94 (d, *J* = 10.2 Hz, 1H), 1.82 – 1.70 (m, 2H), 1.39 – 1.13 (m, 4H). **^{13}C NMR** (101 MHz, CD_3OD) δ 94.7, 76.7, 76.4, 75.4, 71.2, 66.2, 62.0, 58.2, 56.7, 55.8 (2xC), 40.0 (2xC), 33.2, 32.8, 26.6, 21.4. **HRMS (ESI+) m/z** calc. for $C_{17}H_{33}N_4O_5$ $[M+H]^+$: 373.2445, found 373.2449.

Compound 15.

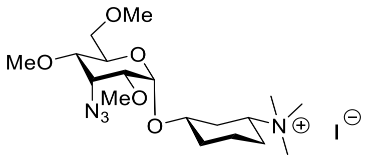

Compound **13** (30 mg, 0.1 mmol) was dissolved in MeOH (3 mL) and treated according to *general procedure XI*. The crude was purified by column chromatography on silica gel (AcOEt/MeOH/NH_4OH 17:2:1) to obtain compound **15** (28 mg, 90%). **^{1}H NMR** (400 MHz, CD_3OD) δ 5.13 (d, *J* = 4.1 Hz, 1H), 4.61 (t, *J* = 3.6 Hz, 1H), 4.03 (ddd, *J* = 10.0, 6.5, 1.8 Hz, 1H), 3.77 – 3.57 (m, 2H), 3.56 – 3.50 (m, 3H), 3.49 (s, 3H), 3.44 (s, 3H), 3.38 (s, 3H), 3.31 (dd, *J* = 3.7, 2.4 Hz, 1H), 3.14 (s, 9H), 2.72 (d, *J* = 11.2 Hz, 1H), 2.29 – 2.17 (m, 1H), 2.15 – 1.99 (m, 2H), 1.63 (q, *J* = 11.4 Hz, 1H), 1.58 – 1.26 (m, 3H). **^{13}C NMR** (101 MHz, CD_3OD) δ 95.4, 76.5, 76.2, 75.8, 72.1, 72.0, 66.4, 58.3, 56.6, 55.9, 55.8, 50.4, 50.4, 50.3, 33.4, 30.3, 24.9, 20.8. **HRMS (ESI+) m/z** calc. for $C_{18}H_{35}N_4O_5^+$ $[M]^+$: 387.2602, found 387.2604.

Compound M4a.

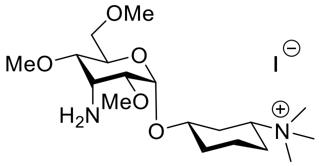

Compound **M4a** was prepared from compound **15** (28 mg, 0.06 mmol) following *general procedure XII*. After washing the crude as explained in the general methods, the desired cationic derivative was extracted using $CHCl_3$/MeOH mixtures (9:1 to 8:2), thus obtaining compound **M4a** (26 mg, 97%), which was not purified further before submitting it to the counterion exchange reaction.

Compound M4.

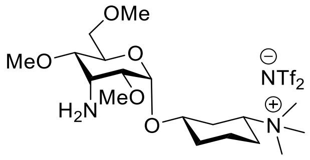

Compound **M4** was prepared from compound **M4a** (26 mg, 0.1 mmol) and $LiNTf_2$ (22 mg, 0.1 mmol) as per *general procedure XIII*, to finally give **M4** (24 mg, 92%). **^{1}H NMR** (400 MHz, CD_3OD) δ 5.15 (d, *J* = 3.8 Hz, 1H), 3.98 (ddd, *J* = 10.5, 6.7, 1.8 Hz, 1H), 3.83 (t, *J* = 4.0 Hz, 1H), 3.70 (tt, *J* = 10.8, 3.0 Hz, 2H), 3.53 (dd, *J* = 10.4, 6.7 Hz, 1H), 3.47 – 3.42 (m, 4H), 3.40 - 3.38 (m 4H), 3.37 (s, 3H), 3.23 (dd, *J* = 10.3, 4.0 Hz, 1H), 3.12 (s, 9H), 2.77 (d, *J* = 11.3 Hz, 1H), 2.24 – 2.16 (m, 1H), 2.12 – 2.04 (m, 1H), 2.00 (d, *J* = 11.2 Hz, 1H), 1.64 (q, *J* = 11.4 Hz, 1H), 1.53 – 1.26 (m, 3H). **^{13}C NMR** (101 MHz, CD_3OD) δ 119.8 (q, *J* = 320.4 Hz, 2x Tf), 96.7, 77.1, 75.4, 75.4, 72.4, 71.9, 65.7, 58.3, 55.8, 55.6, 50.4, 50.4, 50.4, 47.2, 33.4, 30.4, 24.8, 20.7. **HRMS (ESI+) m/z** calc. for $C_{18}H_{37}N_2O_5^+$ $[M]^+$: 361.2697, found 361.2696.

Compound 16.

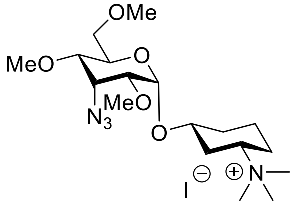

Compound **14** (31 mg, 0.1 mmol) was dissolved in MeOH (3 mL) and treated according to *general procedure XI*. Crude **16** was employed in the next step without further purification and it was characterized only by HRMS. **HRMS (ESI+) m/z**: calc. for $C_{18}H_{35}N_4O_5^+$ $[M]^+$: 387.2602, found 387.2602.

Compound M5a.

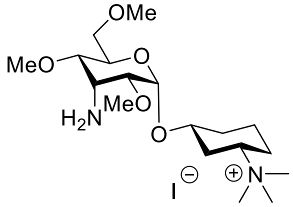

Compound **M5a** was prepared from compound **16** (40 mg, 0.1 mmol) following *general procedure XII*. After washing the crude as explained in the general methods, the desired cationic derivative was extracted using $CHCl_3$/MeOH mixtures (9:1 to 8:2), thus obtaining compound **M5a** (34 mg, 91%), which was not purified further before submitting it to the counterion exchange reaction.

Compound M5.

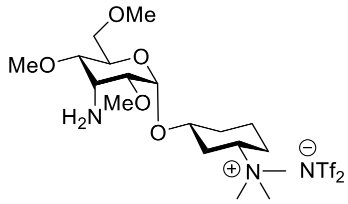

Compound **M5** was prepared from compound **M5a** (34 mg, 0.1 mmol) and $LiNTf_2$ (26 mg, 0.1 mmol), as per *general procedure XIII*, to finally give

M5 (28 mg, 82%). **^{1}H NMR** (400 MHz, CD_3OD) δ 5.17 (d, *J* = 3.6 Hz, 1H), 3.90 (ddt, *J* = 9.9, 5.1, 2.5 Hz, 1H), 3.82 (t, *J* = 4.0 Hz, 1H), 3.77 – 3.68 (m, 1H), 3.63 (dt, *J* = 7.2, 2.5 Hz, 2H), 3.48 (s, 3H), 3.46 – 3.35 (m, 8H, 2xOMe, H-2), 3.32 – 3.27 (m, 1H), 3.14 (s, 9H), 2.63 – 2.53 (m, 1H), 2.21 (td, *J* = 10.2, 5.5 Hz, 1H), 2.14 (d, *J* = 7.7 Hz, 1H), 2.05 – 1.98 (m, 1H), 1.40 (tdd, *J* = 8.7, 4.7, 2.4 Hz, 4H). **^{13}C NMR** (101 MHz, CD_3OD) δ 119.9 (q, *J* = 320.5 Hz, 2x Tf), 96.3, 76.0, 75.9, 75.2, 72.0, 71.6, 65.6, 58.3, 55.8, 55.7, 50.6, 50.52, 50.48, 47.0, 31.9, 31.9, 24.9, 20.9. **HRMS (ESI+) m/z** calc. for $C_{18}H_{37}N_2O_5^+$ $[M]^+$: 361.2697, found 361.2704.

Compound 17α.

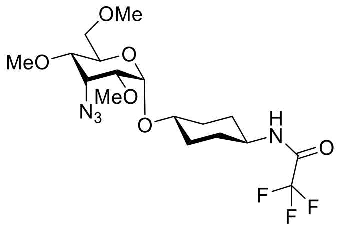

Compound **17α** was obtained by reaction of donor **D1** (670 mg, 1.97 mmol) with acceptor **A5** (542 mg, 2.56 mmol) following *general procedure VII*. The crude was purified by column chromatography on silica gel (Hex/AcOEt 6:4) to give the α-anomer (415 mg, 47%; α/β ratio 1.5:1). Compound **17α**: **^{1}H NMR** (400 MHz, $CDCl_3$) δ 6.36 (d, *J* = 7.9 Hz, 1H), 5.02 (d, *J* = 3.9 Hz, 1H), 4.40 (t, *J* = 3.6 Hz, 1H), 3.94 (dt, *J* = 9.7, 2.8 Hz, 1H), 3.76 (dtt, *J* = 10.9, 7.6, 3.7 Hz, 1H), 3.61 – 3.48 (m, 3H), 3.39 (s, 6H), 3.34 (m, 5H), 2.01 (td, *J* = 14.3, 4.2 Hz, 4H), 1.65 – 1.40 (m, 2H), 1.37 – 1.18 (m, 2H). **^{13}C NMR** (101 MHz, $CDCl_3$) δ 156.6 (q, $^2J_{C-CF3}$ = 36.7 Hz), 115.9 (q, $^1J_{C-F}$ = 288.1) 94.5, 77.2, 75.4, 74.8, 70.8, 66.1, 59.4, 57.2, 57.0, 56.6, 48.5, 31.2, 29.9, 29.7, 29.3. **HRMS (ESI+) m/z** calc. for $C_{17}H_{27}F_3N_4NaO_6$ $[M+Na]^+$: 463.1775, found 463.1777.

Compound 18.

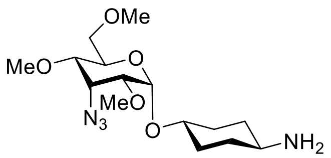

Starting with compound **17α** (150 mg, 0.3 mmol), *general procedure IX* was followed. The crude was purified by silica gel column chromatography (CH_2Cl_2/MeOH, 8:2) to give compound **18** (100 mg, 85%). **^{1}H NMR** (400 MHz, CD_3OD) δ 5.02 (d, *J* = 4.0 Hz, 1H), 4.52 (t, *J* = 3.6 Hz, 1H), 3.89 (dt, *J* = 9.9, 3.5 Hz, 1H), 3.59 – 3.49 (m, 3H), 3.42 (s, 3H), 3.41 – 3.37 (m, 4H), 3.36 – 3.30 (m, 4H), 2.80 (tt, *J* = 10.9, 3.9 Hz, 1H), 2.09 – 1.93 (m, 4H), 1.50 – 1.16 (m, 4H). **^{13}C NMR** (101 MHz, CD_3OD) δ 94.5, 76.9, 75.6, 75.3, 71.3, 66.1, 58.3, 56.7, 55.79, 55.75, 49.2, 31.3, 31.2, 31.1, 29.3. **HRMS (ESI+) m/z**: calc. for $C_{15}H_{29}N_4O_5$ $[M+H]^+$: 345.2132, found 345.2118.

Compound 19.

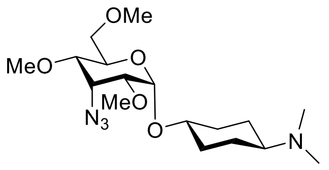

Compound **19** was obtained by reacting a solution of compound **18** (68 mg, 0.2 mmol) in MeOH (2 mL) as per *general procedure X*. The crude was purified by silica gel column chromatography (CH_2Cl_2/MeOH, 9:1) to give compound **19** (88 mg, 86%). **^{1}H NMR** (400 MHz, CD_3OD) δ 5.05 (d, *J* = 4.0 Hz, 1H), 4.54 (t, *J* = 3.6 Hz, 1H), 3.91 (dt, *J* = 9.8, 3.3 Hz, 1H), 3.60 (d, *J* = 0.8 Hz, 1H), 3.58 – 3.55 (m, 2H), 3.55 – 3.47 (m, 1H), 3.46 - 3.43 (m, 4H), 3.42 (s, 3H), 3.36 (s, 3H), 3.36 – 3.32 (m, 1H), 2.30 (s, 6H), 2.10 (td, *J* = 8.2, 3.9 Hz, 2H), 2.02 – 1.88 (m, 2H), 1.56 – 1.24 (m, 4H). **^{13}C NMR** (101 MHz, CD_3OD) δ 94.4, 76.9, 76.3, 75.3, 71.2, 66.1, 62.9, 58.3, 56.8, 55.8 (2xC), 40.5 (2xC), 32.0, 30.0, 26.1, 26.0. **HRMS (ESI+) m/z** calc. for $C_{17}H_{33}N_4O_5$ $[M+H]^+$: 373.2445, found 373.2448.

Compound 20.

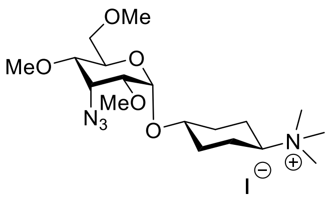

A solution of compound **19** (73 mg, 0.2 mmol) in MeOH (3 mL) was reacted according to *general procedure XI*. The crude was purified by silica gel column chromatography (AcOEt/MeOH/NH_4OH 17:2:1) to obtain compound **20** (30 mg, 40%). **^{1}H NMR** (400 MHz, D_2O) δ 4.97 (d, *J* = 4.1 Hz, 1H), 4.57 (t, *J* = 3.8 Hz, 1H), 3.76 (ddd, *J* = 10.3, 4.6, 2.4 Hz, 1H), 3.55 – 3.40 (m, 4H), 3.37 – 3.27 (m, 7H), 3.22 (s, 4H), 2.90 (s, 9H), 2.20 – 1.93 (m, 4H), 1.57 – 1.32 (m, 3H), 1.32 – 1.19 (m, 1H). **^{13}C NMR** (101 MHz, D_2O) δ 94.2, 75.4, 74.98, 74.96, 72.9, 70.7, 65.2, 58.6, 56.8 (2xC), 56.4, 51.1, 51.02, 50.98, 30.9, 29.1, 23.9, 23.7. **HRMS (ESI+) m/z**: calc. for $C_{18}H_{35}N_4O_5^+$ $[M]^+$: 387.2602, found 387.2614.

Compound M6a.

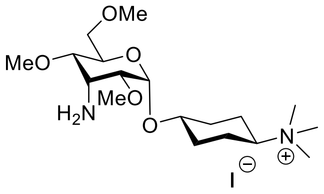

Compound **M6a** was prepared from compound **20** (73 mg, 0.2 mmol) following *general procedure XII*. After washing the crude as explained in the general methods, the desired cationic derivative was extracted using CH_2Cl_2/MeOH mixtures (9:1 to 8:2), thus obtaining compound **M6a** (20 mg, 71%), which was not purified further before submitting it to the counterion exchange reaction.

Compound M6.

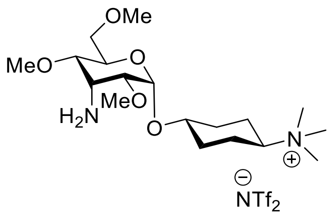

Compound **M6** was prepared from compound **M6a** (20 mg, 0.1 mmol) and $LiNTf_2$ (32 mg, 0.1 mmol), as per *general procedure XIII*, to finally give **M6** (18 mg, 90%). **^{1}H NMR** (400 MHz, CD_3OD) δ 5.00 (d, *J* = 3.6 Hz, 1H), 3.78 (dt, *J* = 10.0, 3.6 Hz, 2H), 3.58 – 3.45 (m, 3H), 3.35 (s, 3H), 3.34 – 3.32 (m, 1H), 3.31 (s, 3H), 3.28 (s, 4H), 3.23 – 3.21(m, 1H), 3.01 (s, 9H), 2.26 – 2.06 (m, 4H), 1.64 – 1.33 (m, 4H). **^{13}C NMR** (101 MHz, CD_3OD) δ 119.8 (q, *J* = 320.4 Hz, 2x Tf), 95.9, 75.8, 75.1, 74.4, 72.9, 71.4, 65.5, 58.2, 56.0, 55.6, 50.52, 50.48, 50.4, 47.3, 31.2, 29.7, 23.9, 23.8. **HRMS (ESI+) m/z** calc. for $C_{18}H_{37}N_2O_5^+$ [M]$^+$: 361.2697, found 361.2690. **HRMS (ESI–) m/z** calc. for $C_2F_6NO_4S_2^-$ [M]$^-$: 279.9178, found 279.9179.

Compound 21.

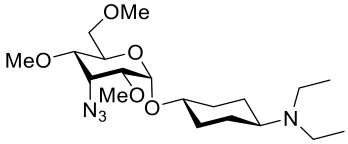

Compound **21** was obtained by reacting a solution of compound **18** (232 mg, 0.7 mmol) in MeOH (7 mL) with acetaldehyde, following *general procedure X*. The crude purified by silica gel column chromatography (CH_2Cl_2/MeOH, 9:1) to give compound **21** (135 mg, 50%). **^{1}H NMR** (400 MHz, CD_3OD) δ 5.05 (d, *J* = 4.0 Hz, 1H), 4.55 (t, *J* = 3.6 Hz, 1H), 3.91 (dt, *J* = 9.9, 3.4 Hz, 1H), 3.57 (d, *J* = 3.4 Hz, 2H), 3.56 – 3.48 (m, 1H), 3.45 (d, *J* = 3.5 Hz, 4H), 3.42 (s, 3H), 3.37 (s, 3H), 3.36 – 3.33 (m, 1H), 2.86 – 2.77 (m, 5H), 2.19 – 2.08 (m, 2H), 2.05 – 1.88 (m, 2H), 1.55 – 1.27 (m, 4H), 1.16 (t, *J* = 7.2 Hz, 6H). **^{13}C NMR** (101 MHz, CD_3OD) δ 94.5, 76.8, 76.0, 75.4, 71.3, 66.2, 59.2, 58.3, 56.8, 55.9, 55.8, 44.0, 43.97, 32.0, 30.1, 26.0, 25.3, 10.9 (2xC). **HRMS (ESI+) m/z** calc. for $C_{19}H_{37}N_4O_5$ [M+H]$^+$: 401.2758, found 401.2756.

Compound 22.

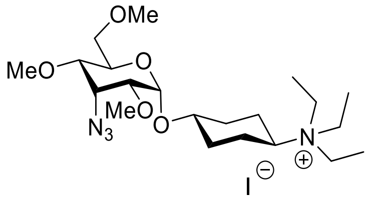

A solution of compound **21** (130 mg, 0.3 mmol) in MeOH (3 mL) was reacted with excess iodoethane (1 mL) according to *general procedure XII*. The crude was purified by silica gel column chromatography (AcOEt/MeOH/NH_4OH 17:2:1) to obtain compound **22** (80 mg, 58%). **^{1}H NMR** (400 MHz, CD_3OD) δ 5.08 (d, *J* = 4.0 Hz, 1H), 4.60 (t, *J* = 3.6 Hz, 1H), 3.91 (ddd, *J* = 10.0, 4.5, 2.3 Hz, 1H), 3.73 – 3.64 (m, 1H), 3.63 – 3.54 (m, 2H), 3.51 – 3.39 (m, 11H), 3.44 (s, 3H), 3.40 (dd, *J* = 10.0, 3.6 Hz, 1H), 3.38 (s, 3H), 2.25 (t, *J* = 7.5 Hz, 4H), 1.89 – 1.74 (m, 2H), 1.67 – 1.44 (m, 2H), 1.37 (tt, *J* = 7.4, 1.6 Hz, 9H). **^{13}C NMR** (101 MHz, CD_3OD) δ 94.6, 76.5,

75.3, 75.0, 71.2, 69.9, 66.2, 58.4, 56.8, 56.1, 56.0, 53.0 (3xC), 31.9, 30.2, 24.6, 24.5, 8.5 (3xC). **HRMS (ESI+) m/z** calc. for $C_{21}H_{41}N_4O_5^+$ $[M]^+$: 429.3071, found 429.3072.

Compound M6Et-a.

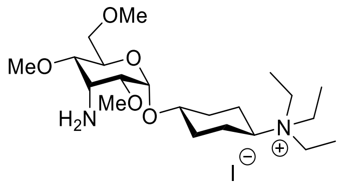

Compound **M6Et-a** was prepared from compound **22** (60 mg, 0.1 mmol) following *general procedure XII*. After washing the crude as explained in the general methods, the desired cationic derivative was extracted using CH_2Cl_2/MeOH mixtures (9:1 to 8:2), thus obtaining compound **M6et-a** (45 mg, 80%), which was not purified further before submitting it to the counterion exchange reaction.

Compound M6Et.

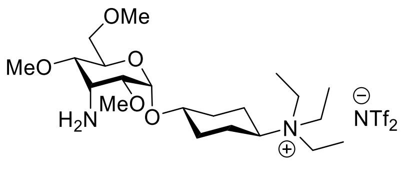

Compound **M6Et** was prepared from compound **M6Et-a** (45 mg, 0.1 mmol) and $LiNTf_2$ (56 mg, 0.2 mmol), as per *general procedure XIII*, to finally give **M6Et** (42 mg, 93%). **^{1}H NMR** (400 MHz, $CDCl_3$) δ 5.02 (d, J = 3.6 Hz, 1H), 3.67 (dt, J = 10.2, 2.9 Hz, 1H), 3.61 – 3.48 (m, 4H), 3.36 (s, 3H), 3.34 – 3.32 (m, 4H), 3.32 (s, 3H), 3.31 – 3.20 (m, 7H), 3.18 (s, 1H), 2.21 – 2.04 (m, 4H), 1.71 – 1.52 (m, 4H), 1.29 (t, J = 7.2 Hz, 9H). **^{13}C NMR** (101 MHz, $CDCl_3$) δ 119.6 (q, J = 320.6 Hz, 2x Tf), 95.5, 75.8, 75.1, 74.7, 70.8, 69.9, 65.2, 59.2, 57.0, 56.9, 53.1 (3xC), 48.1, 31.6, 30.0, 24.7, 24.5, 8.9 (3xC). **HRMS (ESI+) m/z**: calc. for $C_{21}H_{43}N_2O_5^+$ $[M]^+$: 403.3166, found 403.3169.

Compound 23α (23α-a and 23α-b).

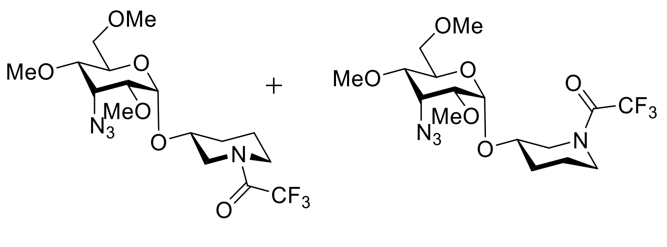

Compound **23α** was obtained by reaction of donor **D1** (520 mg, 1.53 mmol) and acceptor **A6** (393 mg, 1.99 mmol), following *general procedure VII*. The crude was purified by column chromatography on silica gel (Hex/AcOEt 7:3) to give compound **23α** (α-anomer 170 mg, 26%; α/β ratio: 1.1:1, as determined by ^{1}H-NMR of the crude). The glycosylation product could not be purified at this stage. **HRMS (ESI+) m/z** calc. for $C_{16}H_{25}F_3N_4NaO_6$ $[M+Na]^+$: 449.1618, found 449.1611.

Compound 24.

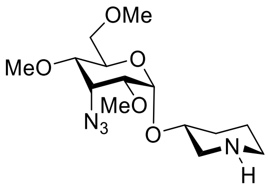

Starting with compound **23α** (140 mg, 0.3 mmol), *general procedure IX* was followed. The crude was purified by silica gel column chromatography (CH_2Cl_2/MeOH, 8:2) to yield one pure diastereomer, compound **24** (52 mg, 40%). **^{1}H NMR** (400 MHz, CD_3OD) δ 5.02 (d, *J* = 4.0 Hz, 1H), 4.64 (t, *J* = 3.6 Hz, 1H), 3.96 (dt, *J* = 10.0, 3.4 Hz, 1H), 3.71 (tt, *J* = 6.0, 2.8 Hz, 1H), 3.61 – 3.54 (m, 2H), 3.49 (s, 3H), 3.49 – 3.45 (m, 1H), 3.43 (s, 3H), 3.36 (s, 3H), 3.35 (dd, *J* = 4.5, 3.3 Hz, 1H), 2.93 (dd, *J* = 13.1, 2.8 Hz, 1H), 2.81 (td, *J* = 12.7, 4.8 Hz, 3H), 1.97 – 1.84 (m, 2H), 1.80 (q, *J* = 8.7 Hz, 1H), 1.57 – 1.47 (m, 1H). **^{13}C NMR** (101 MHz, CD_3OD) δ 95.4, 77.0, 75.2, 72.5, 71.2, 66.4, 58.2, 56.3, 56.0, 55.7, 48.7, 44.9, 30.1, 22.0. **HRMS (ESI+) m/z** calc. for $C_{14}H_{27}N_4O_5$ $[M+H]^+$: 331.1976, found 331.1990.

Compound 25.

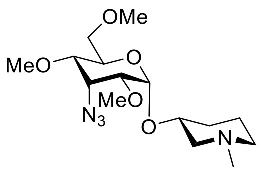

Compound **25** was obtained by reacting a solution of compound **24** (90 mg, 0.3 mmol) in MeOH (3 mL) following *general procedure X*. The crude was purified by silica gel column chromatography (CH_2Cl_2/MeOH, 9:1) to give compound **25** (65 mg, 70%). **^{1}H NMR** (400 MHz, CD_3OD) δ 5.05 (d, *J* = 4.2 Hz, 1H), 4.55 (t, *J* = 3.6 Hz, 1H), 3.97 – 3.88 (m, 1H), 3.74 – 3.62 (m, 1H), 3.59 – 3.53 (m, 2H), 3.46 (s, 3H), 3.45 – 3.42 (m, 1H), 3.41 (s, 3H), 3.37 – 3.35 (m, 4H), 3.07 – 2.97 (m, 1H), 2.74 (d, *J* = 11.5 Hz, 1H), 2.31 (s, 3H), 2.09 – 1.92 (m, 3H), 1.77 (dt, *J* = 13.7, 3.7 Hz, 1H), 1.65 – 1.49 (m, 1H), 1.44 – 1.27 (m, 1H). **^{13}C NMR** (101 MHz, CD_3OD) δ 95.3, 76.9, 75.3, 73.7, 71.2, 66.4, 59.2, 58.3, 56.6, 55.84, 55.77, 54.8, 44.9, 30.7, 22.8. **HRMS (ESI+) m/z** calc. for $C_{15}H_{29}N_4O_5$ $[M+H]^+$: 345.2132, found 345.2131.

Compound 26.

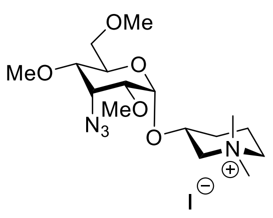

Compound **25** (55 mg, 0.2 mmol) was dissolved in MeOH (3 mL) and treated according to *general procedure XI*. The resulting crude was purified through a silica gel column chromatography (AcOEt/MeOH/NH_4OH 17:2:1) to give compound **26** (50 mg, 87%). **^{1}H NMR** (400 MHz, CD_3OD) δ 5.13 (d, *J* = 4.2 Hz, 1H), 4.70 (q, *J* = 3.0 Hz, 1H), 4.22 - 4.17 (m, 1H), 3.87 (ddt, *J* = 9.6, 4.5, 2.0 Hz, 1H), 3.70 – 3.46 (m, 11H), 3.45 – 3.42, (s, 3H), 3.39 (s, 3H), 3.37 (s, 3H), 3.23 (s, 3H), 2.22 (dt, *J* = 13.3, 7.8 Hz, 1H), 2.03 (d, *J* = 11.8 Hz, 1H), 1.98 – 1.90 (m, 2H). **^{13}C NMR** (101 MHz, CD_3OD) δ 97.5, 78.3, 76.2, 76.1, 72.6, 71.9, 68.2, 64.6, 63.7, 59.7, 57.8, 57.2, 56.9, 52.8, 28.2, 17.9. **HRMS (ESI+) m/z** calc. for $C_{16}H_{31}N_4O_5^+$ $[M]^+$: 359.2289, found 359.2289.

Compound M7a.

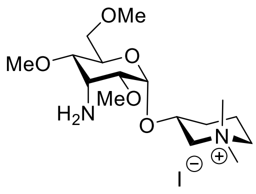

Compound **M7a** was prepared from compound **26** (55 mg, 0.2 mmol) following *general procedure XII*. After washing the crude as explained in the general methods, the desired cationic derivative was extracted using $CHCl_3$/MeOH mixtures (9:1 to 8:2), thus obtaining compound **M7a** (40 mg, 78%), which was not purified further before submitting it to the counterion exchange reaction.

Compound M7.

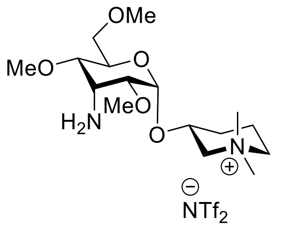

Compound **M7** was prepared from compound **M7a** (40 mg, 0.1 mmol) and $LiNTf_2$ (41 mg, 0.1 mmol), as per *general procedure XIII,* to finally give **M7** (32 mg, 80%). **^{1}H NMR** (400 MHz, CD_3OD) δ 5.11 (d, *J* = 3.7 Hz, 1H), 4.18 (t, *J* = 5.0 Hz, 1H), 3.97 (t, *J* = 4.1 Hz, 1H), 3.89 (ddd, *J* = 10.4, 4.7, 2.4 Hz, 1H), 3.66 – 3.60 (m, 2H), 3.60 – 3.48 (m, 3H), 3.47-3.43 (m, 5H), 3.43 (s, 3H), 3.38 (s, 3H), 3.38 – 3.32 (m, 1H), 3.28 (s, 3H), 3.15 (d, *J* = 1.9 Hz, 3H), 2.20 – 1.88 (m, 4H). **^{13}C NMR** (101 MHz, CD_3OD) δ 119.9 (q, *J* = 320.8 Hz), 96.2, 75.1, 74.3, 71.3, 69.5, 65.9, 62.0 (2xC), 58.3, 56.0, 56.0, 53.1, 52.9, 46.2, 27.3, 17.0. **HRMS (ESI+) m/z** calc. for $C_{16}H_{33}N_2O_5^+$ $[M]^+$: 333.2384, found 333.2389.

Compound 27α.

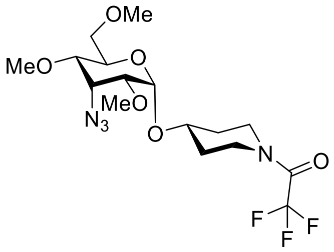

Compound **27α** was obtained by reaction of donor **D1** (520 mg, 1.53 mmol) with acceptor **A7** (393 mg, 1.99 mmol), following *general procedure VII.* The crude was purified by column chromatography on silica gel (Hex/AcOEt 7:3) to give compound **27α** (α-anomer 187 mg, 27%, α/β ratio 1:1). **^{1}H NMR** (400 MHz, $CDCl_3$, 20 ºC, mixture of rotamers) δ 5.07 (dd, *J* = 8.1, 3.9 Hz, 1H), 4.50 (q, *J* = 3.1 Hz, 1H), 3.97 (td, *J* = 7.7, 4.5 Hz, 2H), 3.88 (dd, *J* = 13.0, 6.9 Hz, 1H), 3.75 (ddt, *J* = 14.0, 8.9, 4.8 Hz, 1H), 3.70 – 3.49 (m, 4H), 3.44 (s, 3H), 3.42 (s, 4H), 3.37 (s, 3H), 3.34 (ddd, *J* = 9.8, 3.5, 1.7 Hz, 1H), 1.95 – 1.69 (m, 4H). **^{13}C NMR** (101 MHz, $CDCl_3$) δ 155.7 (q, $^2J_{C\text{-}CF3}$ = 35.9 Hz), 116.7 (q, $^1J_{C\text{-}F}$ = 287.8), 95.3, 95.0, 78.0, 77.9, 77.3, 74.9, 74.9, 71.7, 71.2, 70.8, 70.8, 66.6, 66.6, 59.5, 57.1, 56.0, 42.4, 42.2, 40.2, 39.9, 32.6, 31.7, 31.0, 29.8. **HRMS (ESI+) m/z** calc. for $C_{16}H_{25}F_3N_4NaO_6$ $[M+Na]^+$: 449.1618, found 449.1626.

Compound 28.

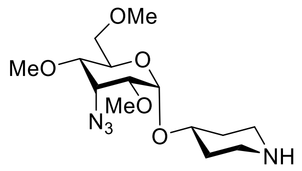

Starting with compound **27α** (170 mg, 0.4 mmol), *general procedure IX* was followed. The crude was purified by silica gel column chromatography (CH_2Cl_2/MeOH, 8:2) to yield compound **28** (95 mg, 72%). **^{1}H NMR** (400 MHz, CD_3OD) δ 5.06 (d, *J* = 4.0 Hz, 1H), 4.64 (t, *J* = 3.5 Hz, 1H), 3.99 (td, *J* = 4.9, 2.4 Hz, 1H), 3.90 (ddd, *J* = 9.9, 5.1, 2.1 Hz, 1H), 3.61 – 3.51 (m, 2H), 3.51 – 3.48 (m, 1H), 3.47 (s, 3H), 3.45 – 3.35 (m, 5H), 3.35 (s, 3H), 3.33 – 3.30 (m, 1H), 3.22 – 3.12 (m, 2H), 2.08 – 1.93 (m, 4H).**^{13}C NMR** (101 MHz, CD_3OD) δ 95.1, 77.2, 75.0, 71.3, 69.1, 66.7, 58.2, 56.1, 56.0, 55.6, 40.3, 40.0, 28.5, 26.8. **HRMS (ESI+) m/z** calc. for $C_{14}H_{27}N_4O_5$ $[M+H]^+$: 331.1976, found 331.1987.

Compound 29.

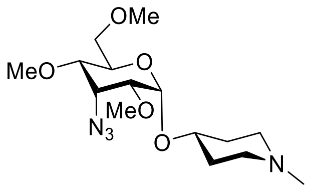

Compound **29** was obtained by reacting a solution of compound **28** (85 mg, 0.3 mmol) in MeOH (3 mL) following *general procedure X*. The crude was purified by silica gel column chromatography (CH_2Cl_2/MeOH, 9:1) to give compound **29** (75 mg, 85%). **^{1}H NMR** (400 MHz, CD_3OD) δ 5.03 (d, *J* = 3.9 Hz, 1H), 4.61 – 4.52 (m, 1H), 3.90 (dt, *J* = 10.0, 3.5 Hz, 1H), 3.71 (s, 1H), 3.53 (dd, *J* = 3.5, 1.4 Hz, 2H), 3.47 – 3.41 (m, 4H), 3.38 (s, 3H), 3.33 (s, 3H), 3.31 – 3.26 (m, 1H), 2.76 (q, *J* = 10.0 Hz, 2H), 2.46 – 2.32 (m, 2H), 2.29 (s, 3H), 1.98 – 1.65 (m, 4H).**^{13}C NMR** (101 MHz, CD_3OD) δ 94.8, 77.4, 75.1, 71.8, 71.3, 66.4, 58.3, 56.2, 55.9, 55.6, 52.0 (2xC), 44.7, 31.5, 29.7. **HRMS (ESI+) m/z** calc. for $C_{15}H_{29}N_4O_5$ $[M+H]^+$: 345.2132, found 345.2145.

Compound 30.

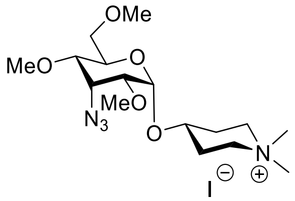

Compound **29** (75 mg, 0.2 mmol) was dissolved in MeOH (3 mL) and treated according to *general procedure XI*. The resulting crude was purified through a silica gel column chromatography (AcOEt/MeOH/NH_4OH 17:2:1) to give compound **30** (35 mg, 45%). **^{1}H NMR** (500 MHz, CD_3OD) δ 5.08 (d, *J* = 4.0 Hz, 1H), 4.71 (t, *J* = 3.5 Hz, 1H), 4.04 (tt, *J* = 5.1, 3.0 Hz, 1H), 3.92 (ddd, *J* = 10.0, 5.3, 2.0 Hz, 1H), 3.70 – 3.59 (m, 3H), 3.57 – 3.52 (m, 2H), 3.51 (s, 3H), 3.49 – 3.44 (m, 2H), 3.43 (s, 3H), 3.37 – 3.34 (m, 4H), 3.21 (t, *J* = 1.9 Hz, 6H), 2.28 (dd, *J* = 15.4, 11.7 Hz, 1H), 2.18 (t, *J* = 13.5 Hz, 1H), 2.06 (d, *J* = 16.0 Hz, 2H). **^{13}C NMR** (126 MHz, CD_3OD) δ 94.8, 77.0, 75.0, 71.3, 67.1, 66.7, 58.3, 58.3 (2xC), 56.2, 56.0, 55.8, 54.1, 48.1, 26.2, 24.4. **HRMS (ESI+) m/z** calc. for $C_{16}H_{31}N_4O_5^+$ $[M]^+$: 359.2289, found 359.2284.

Compound M8a.

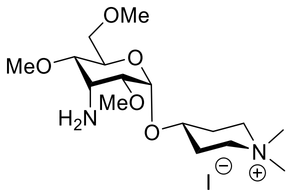

Compound **M8a** was prepared from compound **30** (20 mg, 0.1 mmol) following *general procedure XII*. After washing the crude as explained in the general methods, the desired cationic derivative was extracted using $CHCl_3$/MeOH mixtures (9:1 to 8:2), thus obtaining compound **M8a** (15 mg, 83%), which was not purified further before submitting it to the counterion exchange reaction.

Compound M8.

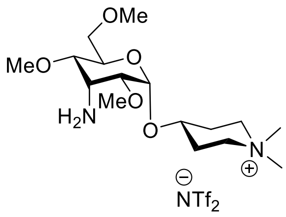

Compound **M8** was prepared from compound **M8a** (15 mg, 0.1 mmol) and $LiNTf_2$ (19 mg, 0.1 mmol), as per *general procedure XIII*, to finally give **M8** (14 mg, 93%). **^{1}H NMR** (400 MHz, CD_3OD) δ 5.03 (d, *J* = 3.9 Hz, 1H), 4.02 – 3.97 (m, 1H), 3.92 (ddd, *J* = 10.3, 5.6, 1.9 Hz, 1H), 3.80 (t, *J* = 4.1 Hz, 1H), 3.67 – 3.52 (m, 4H), 3.45 (s, 3H), 3.39 (s, 3H), 3.39 – 3.34 (m, 6H), 3.23 – 3.18 (m, 4H), 3.15 (s, 3H), 2.30 – 1.98 (m, 4H). **^{13}C NMR** (101 MHz, CD_3OD) δ 119.8 (q, *J* = 320.8 Hz), 96.0, 76.2, 75.8, 71.8 67.8, 65.8, 58.5, 58.2 (2xC), 55.6, 55.5, 53.2 48.3, 46.4, 26.0, 24.2. **HRMS (ESI+) m/z** calc. for $C_{16}H_{33}N_2O_5^+$ $[M]^+$: 333.2388, found 333.2384.

Compound 31.

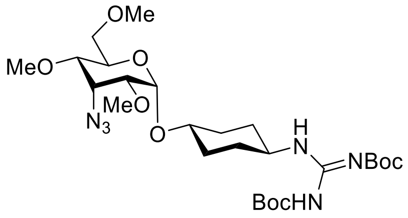

Compound **31** was synthesized from derivative **18** (115 mg, 0.3 mmol), following the guidelines of *general procedure XIV*. The crude was purified by silica gel column chromatography (Hex/AcOEt 7:3) to give compound **31** (140 mg, 72%). **^{1}H NMR** (400 MHz, $CDCl_3$) δ 11.5 (bs, 1H), 8.24 (d, *J* = 8.2 Hz, 1H), 5.28 (s, 1H), 5.06 (d, *J* = 3.9 Hz, 1H), 4.41 (t, *J* = 3.5 Hz, 1H), 4.10 – 3.91 (m, 2H), 3.64 – 3.49 (m, 3H), 3.44 - 3.39 (m, 6H), 3.38 – 3.33 (m, 5H), 2.12 – 2.02 (m, 2H), 2.00 – 1.90 (m, 2H), 1.70 – 1.52 (m, 2H), 1.51 (s, 9H), 1.44 – 1.37 (m, 9H), 1.33 – 1.20 (m, 2H). **^{13}C NMR** (101 MHz, $CDCl_3$) δ 163.8, 155.4, 153.3, 94.5, 83.0, 79.1, 77.3, 75.30, 75.26, 70.8, 66.0, 59.3, 57.1, 56.9, 56.6, 47.8, 30.9, 30.2, 29.9, 29.1, 28.3 (3xC), 28.1 (3xC). **HRMS (ESI+) m/z** calc. for $C_{26}H_{47}N_6O_9$ $[M+H]^+$: 587.3399, found 587.3398.

Compound 32.

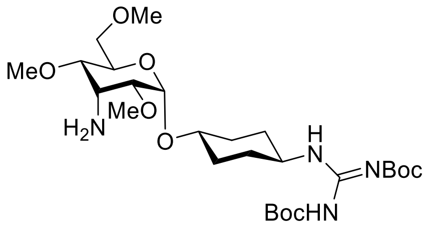

A solution of compound **31** (140 mg, 0.2 mmol) in a 1:1 H_2O/MeOH mixture (1.0 mL, 5 mL/mmol) was treated following *general procedure XII*. The crude was purified by silica gel column chromatography (Hex/AcOEt 7:3) to yield compound **32** (85 mg, 64%). **^{1}H NMR** (400 MHz, CD_3OD) δ 5.09 (d, *J* = 3.7 Hz, 1H), 3.90 – 3.82 (m, 1H), 3.80 (t, *J* = 4.1 Hz, 1H), 3.72 – 3.55 (m, 3H), 3.54 (s, 1H), 3.44 (s, 3H), 3.41 (s, 3H), 3.39 (s, 3H), 3.37 – 3.34 (m, 1H), 3.30 – 3.26 (m, 1H), 2.11 – 1.97 (m, 4H), 1.56 (tdd, *J* = 13.3, 8.4, 3.1 Hz, 1H), 1.48 – 1.39 (m, 20H), 1.42 – 1.23 (m, 1H). **^{13}C NMR** (126 MHz, CD_3OD) δ 162.7, 160.4, 160.3, 95.5, 77.9, 75.8, 75.6, 75.0, 71.5, 65.4, 58.2, 55.7, 55.5, 48.5, 47.3, 47.1, 31.1, 30.1, 30.0, 29.2, 28.3 (3xC), 28.1 (3xC). **HRMS (ESI+) m/z** calc. for $C_{26}H_{49}N_4O_9$ $[M+H]^+$: 561.3399, found 561.3398.

Compound M9a.

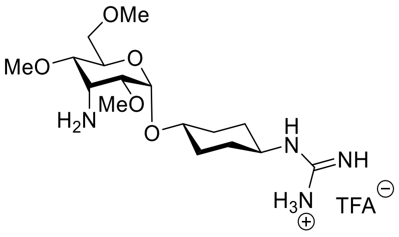

To an ice-cold solution of compound **32** (25 mg, 0.1 mmol) in 3 mL of CH_2Cl_2 an excess of TFA (0.33 mL) was added. The reaction mixture was stirred vigorously until the disappearance of the starting product is observed (approx. 8 h). The mixture was then evaporated under reduced pressure and the resulting crude was used in the next step without further purification.

Compound M9.

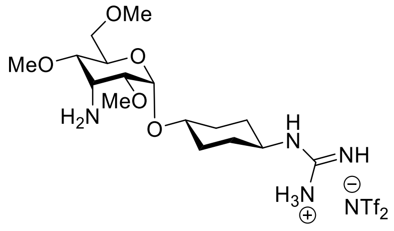

Compound **M9** was prepared from compound **M9a** (16 mg, 0.1 mmol) and $LiNTf_2$ (13 mg, 0.1 mmol), as per *general procedure XIII*, to finally give **M9** (10 mg, 62%). **^{1}H NMR** (400 MHz, CD_3OD) δ 5.19 (d, *J* = 3.5 Hz, 1H), 4.13 (t, *J* = 4.3 Hz, 1H), 3.85 (d, *J* = 4.9 Hz, 1H), 3.71 – 3.60 (m, 3H), 3.59 – 3.54 (m, 1H), 3.52 (t, *J* = 3.9 Hz, 1H), 3.47 (s, 3H), 3.46 (s, 3H), 3.42 – 3.34 (m, 4H), 2.18 – 1.92 (m, 4H), 1.67 – 1.27 (m, 4H). **^{13}C NMR** (101 MHz, CD_3OD) δ 156.5, 119.9 (q, *J* = 320.9 Hz), 94.8, 76.6, 72.8, 72.3, 70.9, 65.6, 58.4, 57.0, 56.0, 49.4, 48.7, 30.8, 30.1, 29.9, 29.8. **HRMS (ESI+) m/z** calc. for $C_{16}H_{33}N_4O_5^+$ $[M]^+$: 361.2445, found 361.2454. **HRMS (ESI-) m/z** calc. for $C_2F_6NO_4S_2^-$ $[M]^{-}$: 279.9178, found 279.9184.

Compound 33.

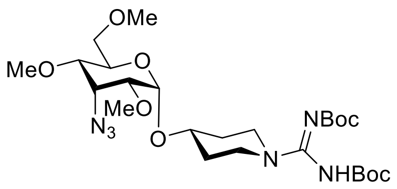

Compound **33** was synthesized from derivative **28** (50 mg, 0.2 mmol) following the guidelines of *general procedure XIV*. The crude was purified by silica gel column chromatography (Hex/AcOEt 4:6) to give compound **33** (38 mg, 44%). **^{1}H NMR** (500 MHz, $CDCl_3$) δ 5.09 (d, *J* = 3.7 Hz, 1H), 4.47 (t, *J* = 3.6 Hz, 1H), 3.98 (dt, *J* = 10.1, 2.5 Hz, 1H), 3.90 (dq, *J* = 6.7, 3.4 Hz, 1H), 3.73 (s, 1H), 3.67 – 3.58 (m, 1H), 3.58 – 3.50 (m, 1H), 3.43 (s, 3H), 3.42 (s, 3H), 3.42 – 3.39 (m, 1H), 3.38 (s, 3H), 3.35 (ddd, *J* = 9.8, 3.2, 1.1 Hz, 1H), 2.03 – 1.74 (m, 4H,), 1.47 (s, 18H), 1.33 – 1.17 (m, 4H). **^{13}C NMR** (126 MHz, $CDCl_3$) δ 155.1, 155.0 (2xC), 94.8, 79.4, 77.8, 77.7, 74.9, 72.5, 70.7, 66.3, 59.3, 57.0, 56.9, 56.2, 31.9, 30.1 (2xC), 28.4, 28.1 (6xC). **HRMS (ESI+) m/z** calc. for $C_{25}H_{45}N_6O_9$ $[M+H]^+$: 573.3243, found 573.3244.

Compound 34.

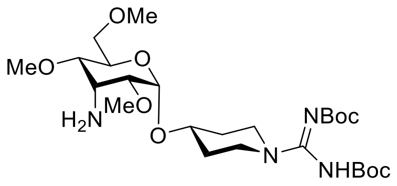

A solution of compound **33** (38 mg, 0.06 mmol) in a 1:1 H_2O/MeOH mixture (5 mL/mmol) was treated according to *general procedure XII.* The crude was purified by silica gel column chromatography (CH_2Cl_2/MeOH 8:2) to yield compound **34** (13 mg, 36%). **^{1}H NMR** (400 MHz, CD_3OD) δ 5.08 (d, *J* = 3.6 Hz, 1H), 3.91 (dt, *J* = 7.1, 3.6 Hz, 1H), 3.86 (ddd, *J* = 10.2, 4.3, 2.4 Hz, 1H), 3.78 (q, *J* = 3.1 Hz, 1H), 3.74 – 3.60 (m, 2H), 3.60 – 3.56 (m, 2H), 3.42 (d, *J* = 1.8 Hz, 5H), 3.37 (s, 3H), 3.35 (s, 3H), 3.33 (d, *J* = 4.0 Hz, 1H), 3.28 – 3.22 (m, 1H), 1.95 (ddt, *J* = 12.6, 8.1, 4.0 Hz, 1H), 1.87 (td, *J* = 8.6, 3.8 Hz, 1H), 1.73 (dddq, *J* = 17.2, 10.5, 6.3, 3.7 Hz, 2H), 1.45 (s, 18H). **^{13}C NMR** (101 MHz, CD_3OD) δ 155.2, 153.0 (2xC), 95.8, 95.6, 75.8, 75.1, 73.0, 71.6, 65.63, 65.59, 58.3, 55.8, 55.6, 47.1, 43.3 43.1, 31.6, 29.7, 27.2 (6xC). **HRMS (ESI+) m/z** calc. for $C_{25}H_{47}N_4O_9$ $[M+H]^+$: 547.3338, found 547.3340.

Compound M10a.

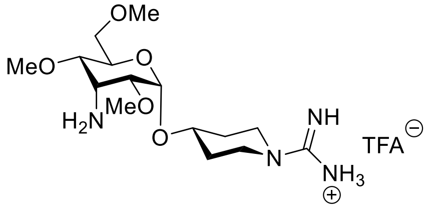

To an ice-cold solution of compound **34** (13 mg, 0.02 mmol) in 3 mL of CH_2Cl_2 an excess of TFA (0.33 mL) was added. The reaction mixture was stirred vigorously until the disappearance of the starting product is observed (approx. 8 h). The mixture was then evaporated under reduced pressure and the resulting crude was used in the next step without further purification.

Compound M10.

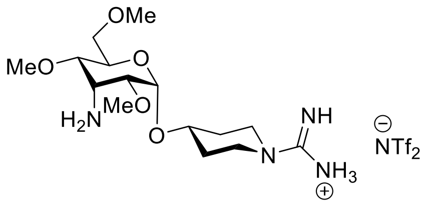

Compound **M10** was prepared from compound **M10a** (13 mg, 0.02 mmol) and $LiNTf_2$ (13 mg, 0.02 mmol), as per *general procedure XIII*, to finally give **M10** (8 mg, 61%). **^{1}H NMR** (400 MHz, CD_3OD) δ 5.11 (d, *J* = 3.6 Hz, 1H, H-1), 4.07 (t, *J* = 4.3 Hz, 1H), 3.91 (tt, *J* = 7.6, 3.7 Hz, 1H), 3.86 – 3.74 (m, 1H), 3.68 – 3.56 (m, 2H), 3.53 (t, *J* = 3.0 Hz, 2H), 3.51 – 3.43 (m, 2H), 3.39 (s, 3H), 3.37 (s, 3H), 3.29 (s, 3H), 3.28 – 3.23 (m, 2H), 2.00 – 1.81 (m, 2H), 1.81 – 1.62 (m, 2H). **^{13}C NMR** (101 MHz, CD_3OD) δ 156.3, 119.8 (q, *J* = 320.9 Hz), 94.7, 73.00, 72.95, 72.3, 70.9, 65.7, 58.3, 57.0, 56.2, 48.3, 42.7, 42.5, 31.0, 29.3. **HRMS (ESI+) m/z** calc. for $C_{15}H_{31}N_4O_5$ $[M+H]^+$: 347.2289, found 347.2289.

Compound 35α.

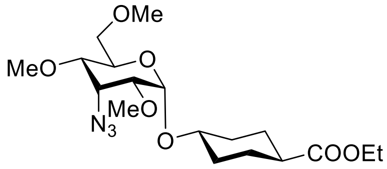

Compound **35α** was obtained from donor **D1** (205 mg, 0.6 mmol) and acceptor **A8** (224 mg, 0.78 mmol) as per *general procedure VII*. The crude was purified by column chromatography on silica gel (Hex/AcOEt 7:3) to give compound **35α** (α-anomer 185 mg, 26%; α/β ratio: 1.3:1). **^{1}H NMR** (400 MHz, $CDCl_3$) δ 5.05 (d, *J* = 3.9 Hz, 1H), 4.39 (t, *J* = 3.6 Hz, 1H), 4.06 (q, *J* = 7.1 Hz, 2H), 3.95 (dt, *J* = 9.7, 2.6 Hz, 1H), 3.63 – 3.48 (m, 3H), 3.39 (s, 6H), 3.37-3.28 (m, 5H), 2.26 – 2.15 (m, 1H), 1.99 (tdd, *J* = 9.6, 4.5, 2.5 Hz, 4H), 1.54 – 1.29 (m, 4H), 1.19 (t, *J* = 7.1 Hz, 3H). **^{13}C NMR** (101 MHz, $CDCl_3$) δ 175.7, 94.3, 77.3, 75.5, 75.4, 70.8, 66.0, 60.3, 59.4, 57.2, 57.0, 56.8, 42.2, 32.2, 30.3, 27.2, 26.9, 14.3. **HRMS (ESI+) m/z** calc. for $C_{18}H_{35}N_4O_7$ $[M+NH_4]^+$: 419.2500, found 419.2499.

Compound M11/R11.

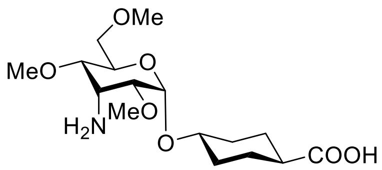

A solution of derivative **35α** (102 mg, 0.25 mmol) in THF (3 mL, 12 mL/mmol) was treated according to *general procedure XII*. The crude was purified by silica gel column chromatography (CH_2Cl_2/MeOH, 8:2) to give compound **R11** (55 mg, 62%). **^{1}H NMR** (500 MHz, CD_3CN) δ 6.99 (s, 3H), 5.12 (d, *J* = 3.6 Hz, 1H), 4.01 (t, *J* = 4.1 Hz, 1H), 3.86 (s, 1H), 3.63 – 3.52 (m, 3H), 3.42 (s, 3H), 3.39 (s, 5H), 3.35 (s, 3H), 2.20 (d, *J* = 11.7 Hz, 1H), 2.15 – 2.07 (m, 1H), 2.07 – 1.97 (m, 3H), 1.59 – 1.31 (m, 4H).**^{13}C NMR** (126 MHz, CD_3CN) δ 174.2, 95.1, 76.8, 74.2, 73.7, 71.2, 65.5, 58.5,

56.7, 56.1, 47.3, 43.3, 32.4, 30.7, 27.7, 27.2. **HRMS (ESI□) m/z** calc. for $C_{16}H_{28}NO_7$ [M-H]$^-$: 346.1871, found 346.1877.

Compound 36α (36α-a and 36α-b).

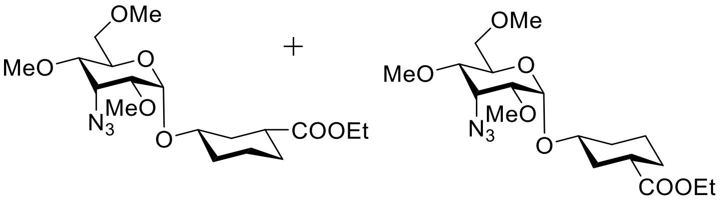

Compound **36α** was obtained from donor **D1** (340 mg, 1.0 mmol) and acceptor **A9** (224 mg, 1.3 mmol), following *general procedure VII*. The crude was purified by column chromatography on silica gel (Hex/AcOEt 7:3) to give the mixture **36α** (mixture of α diastereoisomers 150 mg, 37%; α/β ratio 1.3:1). In this reaction step, the diastereoisomers could not be separated by chromatographic techniques, therefore the spectroscopic characterization is omitted. **HRMS (ESI+) m/z** calc. for $C_{18}H_{32}N_3O_7$ [M+H]$^+$: 402.2162, found 402.2165.

Compounds M12/R12 and M13/R13.

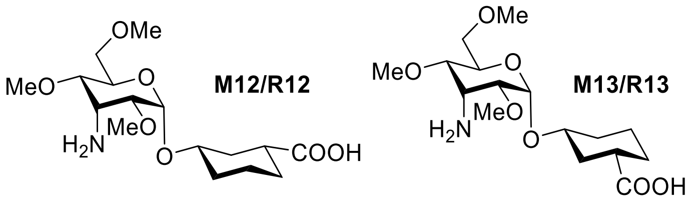

A solution of derivative **36α** (52 mg, 0.13 mmol) in THF (2 mL, 15 mL/mmol) was treated according to *general procedure XII*. The crude was purified by silica gel column chromatography (CH_2Cl_2/MeOH, 8:2) to give compounds **M12/R12** (20 mg, 44%) and **M13/R13** (20 mg, 44%).

Compound **M12/R12**: **^{1}H NMR** (400 MHz, CD_3OD) δ 5.20 (d, *J* = 3.6 Hz, 1H), 4.05 (t, *J* = 4.2 Hz, 1H), 3.93 (ddd, *J* = 10.4, 4.0, 2.0 Hz, 1H), 3.68 (dt, *J* = 10.2, 3.9 Hz, 2H), 3.62 (dd, *J* = 11.0, 2.0 Hz, 1H), 3.57 (dd, *J* = 10.4, 4.3 Hz, 1H), 3.51 – 3.45 (m, 7H), 3.41 (s, 3H), 2.28 – 2.12 (m, 2H), 2.02 – 1.93 (m, 1H), 1.91 – 1.74 (m, 2H), 1.63 (q, *J* = 11.1 Hz, 1H), 1.40 – 1.24 (m, 3H). **^{13}C NMR** (101 MHz, CD_3OD) δ 186.3, 98.3, 80.9, 77.3, 76.5, 74.6, 69.3, 62.2, 60.7, 59.7, 52.6, 48.4, 40.1, 34.8, 32.7, 26.6. **HRMS (ESI□) m/z** calc. for $C_{16}H_{28}NO_7$ [M-H]$^-$: 346.1871, found 346.1872.

Compound **M13/R13**: **^{1}H NMR** (400 MHz, CD_3OD) δ 5.16 (d, *J* = 3.5 Hz, 1H), 3.95 (t, *J* = 4.1 Hz, 1H), 3.87 (ddd, *J* = 10.4, 4.3, 2.1 Hz, 1H), 3.65 – 3.53 (m, 3H), 3.46 – 3.44 (m, 1H), 3.44 (s, 3H), 3.42 - 3.40 (m, 4H), 3.36 (s, 3H), 2.22 – 2.08 (m, 2H), 2.01 (d, *J* = 10.7 Hz, 1H), 1.87 – 1.71 (m, 2H), 1.53 – 1.20 (m, 4H). **^{13}C NMR** (101 MHz, CD_3OD) δ 182.9, 95.4, 78.7, 73.9, 73.2, 71.0, 65.6, 58.3, 56.6, 55.8,

48.5, 45.0, 35.5, 32.9, 28.9, 23.4. **HRMS (ESI⁻) m/z** calc. for $C_{16}H_{28}NO_7$ $[M-H]^-$: 346.1871, found 346.1873.

1.6 Synthesis of reference systems R0, R3-R10

Compound 37.

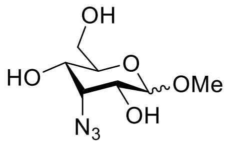

To a solution of 3-azido-3-deoxy-1,2;5,6-bis-*O*-isopropylidene-□-D-allofuranose[8] (1.0 g, 3.5 mmol) in MeOH (18 mL, 5 mL/mmol), acetyl chloride (0.36 mL, 2% vol) was added slowly at 0 °C. The reaction mixture was stirred at rt for 16 h. Next, the solvent was evaporated under vacuum and the resulting residue was purified by silica gel column chromatography (CH_2Cl_2/MeOH 9:1) to give compound **37** (330 mg, 43%) as a 1:10 α/β mixture. The spectroscopic data obtained agree with published characterization in the literature.[9]

Compound 38.

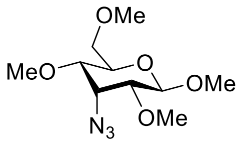

A solution of derivative **37** (320 mg, 1.5 mmol) in THF (7.5 mL, 5 mL/mmol) was per-*O*-methylated following the guidelines of *general procedure I*. The crude was purified by column chromatography on silica gel (Hex/AcOEt, 8:2), obtaining compound **38** (260 mg, 61%) as the β-stereomer. **^{1}H NMR** (400 MHz, $CDCl_3$) δ 4.45 (d, *J* = 7.7 Hz, 1H), 4.35 (t, *J* = 3.3 Hz, 1H), 3.65 (ddt, *J* = 8.6, 4.5, 2.2 Hz, 1H), 3.57 (dd, *J* = 10.7, 2.0 Hz, 1H), 3.50 (dd, *J* = 10.8, 4.2 Hz, 1H), 3.47 (s, 3H), 3.45 (s, 3H), 3.39 (s, 3H), 3.36 – 3.30 (m, 4H), 3.04 (dd, *J* = 7.7, 3.4 Hz, 1H). **^{13}C NMR** (101 MHz, CDCl*3*) δ 101.3, 79.2, 76.0, 72.3, 71.0, 59.7, 59.4, 58.7, 57.4, 56.9. **HRMS (ESI+) m/z** calc. for $C_{10}H_{20}N_3O_5$ $[M+H]^+$: 261.1325, found 261.1327.

Compound R0β.

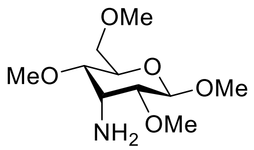

A solution of derivative **38** (160 mg, 0.61 mmol) in THF (9 mL, 15 mL/mmol) was treated according to *general procedure XII*. The crude was purified by silica gel column chromatography (CH_2Cl_2/MeOH, 9:1) to give compound **R0β** (125 mg, 87%). **^{1}H NMR** (500 MHz, $CDCl_3$) δ 4.69 (d, *J* = 7.7 Hz, 1H), 3.91 (ddd, *J* = 9.6, 4.8, 2.1 Hz, 1H), 3.85 (t, *J* = 3.7 Hz, 1H), 3.60 (dd, *J* = 10.8, 2.1 Hz, 1H), 3.50 (dd, *J* = 10.8, 4.8 Hz, 1H), 3.48 (s, 3H), 3.43 (s, 3H), 3.35 (s, 3H), 3.33 (s, 3H), 3.21 (dd, *J* = 9.6, 3.6 Hz, 1H), 2.97 (dd, *J* = 7.7, 3.7 Hz, 1H), 2.29 (s, 2H). **^{13}C NMR** (126 MHz, $CDCl_3$) δ 100.5, 80.1, 76.8, 71.9, 71.3, 59.3, 58.0, 56.9, 56.8, 47.1. **HRMS (ESI+) m/z** calc. for $C_{10}H_{21}NNaO_5$ $[M+Na]^+$: 258.1318, found 258.1321.

Compound 39.

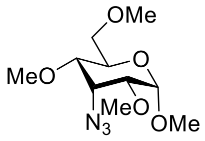

A solution of compound **38** (260 mg, 1 mmol) in MeOH (5 mL, 5 mL/mmol) was treated with concentrated H_2SO_4 (2-3 drops) and the reaction was stirred at reflux (65 °C) for 16 h. Afterwards, it was allowed to cool to rt and a few drops of Et_3N were added to neutralize the acid. Then it was concentrated under vacuum and the resulting crude was partitioned between CH_2Cl_2 and brine. The combined organic phases were dried over anhydrous $MgSO_4$ and the solvent was evaporated. The residue was purified by silica gel column chromatography (Hex/AcOEt 8:2) to give the α-epimer **39** (230 mg, 92%). **^{1}H NMR** (500 MHz, $CDCl_3$) δ 4.84 (d, *J* = 4.0 Hz, 1H), 4.44 (t, *J* = 3.7 Hz, 1H), 3.94 – 3.89 (m, 1H), 3.67 – 3.58 (m, 2H), 3.47 (s, 3H), 3.46 (s, 3H), 3.44 (s, 3H), 3.42 (d, *J* = 3.5 Hz, 1H) 3.41 (s, 3H), 3.39 (t, *J* = 3.9 Hz, 1H). **^{13}C NMR** (126 MHz, $CDCl_3$) δ 97.3, 76.9, 75.5, 70.7, 65.7, 59.3, 57.32, 57.29, 57.1, 56.0. **HRMS (ESI+) m/z** calc. for $C_{10}H_{20}N_3O_5$ $[M+H]^+$: 261.1325, found 261.1321.

Compound R0α.

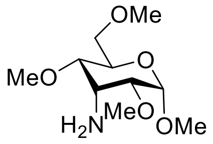

A solution of derivative **39** (29 mg, 0.11 mmol) in THF (2 mL, 20 mL/mmol) was treated according to *general procedure XII*. The crude was purified by silica gel column chromatography (CH_2Cl_2/MeOH, 9:1) to give compound **R0□** (23 mg, 88%). **^{1}H NMR** (300 MHz, $CDCl_3$) δ 4.90 (d, *J* = 3.4 Hz, 1H), 4.30 (t, *J* = 4.0 Hz, 1H), 3.77 – 3.71 (m, 1H), 3.70 – 3.57 (m, 3H), 3.46 (s, 3H), 3.45 – 3.42 (m, 7H), 3.42 (s, 3H). **^{13}C NMR** (101 MHz, $CDCl_3$) δ 97.7, 73.5, 71.7, 70.2, 65.3, 59.5, 58.1, 57.5, 56.0, 48.4. **HRMS (ESI+) m/z** calc. for $C_{10}H_{22}NO_5$ $[M+H]^+$: 236.1493, found 236.14925.

Compound 40α.

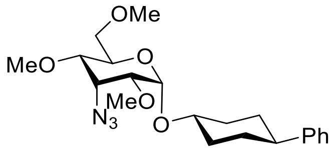

Compound **40α** was obtained by reaction of donor **D1** (145 mg, 0.4 mmol), acceptor **A10** (98 mg, 0.6 mmol), following *general procedure VII.* The crude was purified by column chromatography on silica gel (Hex/AcOEt 8:2) to give compound **40α** (α-anomer: 52 mg, 30%; α/β ratio 1.1:1 as estimated by ^{1}H-NMR of the crude). This product was employed in the next reaction step without further purification. **HRMS (ESI+) m/z** calc. for $C_{21}H_{31}N_3NaO_5$ $[M+Na]^+$: 428.21559, found 428.21708.

Compound R3.

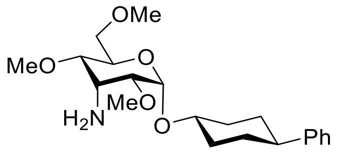

A solution of derivative **40α** (52 mg, 0.13 mmol) in THF (2 mL, 15 mL/mmol) was treated according to *general procedure XII*. The crude was purified by silica gel column chromatography (CH_2Cl_2/MeOH, 9:1) to give compound **R3** (45 mg, 93%). **^{1}H NMR** (400 MHz, CD_3OD) δ 7.32 – 6.95 (m, 5H), 5.11 (d, *J* = 3.7 Hz, 1H), 3.86 (dt, *J* = 10.3, 3.4 Hz, 1H), 3.75 (t, *J* = 4.2 Hz, 1H), 3.72 – 3.49 (m, 3H), 3.42 (s, 3H), 3.41 – 3.37 (m, 6H), 3.35 – 3.29 (m, 1H), 3.27 – 3.22 (m, 1H), 2.48 (ddd, *J* = 11.9, 8.2, 4.0 Hz, 1H), 2.31 – 2.08 (m, 2H), 1.96 – 1.76 (m, 2H), 1.67 – 1.44 (m, 4H). **^{13}C NMR** (101 MHz, CD_3OD) δ 146.6, 128.0 (2xC), 126.5 (2xC), 125.8, 95.5, 77.0, 75.9, 75.3, 71.6, 65.4, 58.3, 55.7, 55.5, 43.6, 33.7, 32.5, 32.3, 32.2, 31.8. **HRMS (ESI+) m/z** calc. for $C_{21}H_{34}NO_5$ $[M+H]^+$: 380.2431, found 380.2428.

Compound 41α.

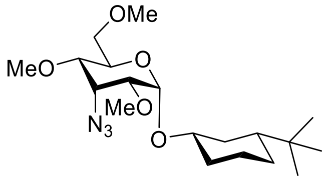

Compound **41α** was obtained by reaction of donor **D1** (182 mg, 0.5 mmol) and acceptor **A11** (120 mg, 0.45 mmol), following *general procedure VII*. The crude was purified by column chromatography on silica gel (Hex/AcOEt 8:2) to give compound **41α** (α-anomer: 42 mg, 30%; α/β ratio 1:1). **^{1}H NMR** (400 MHz, $CDCl_3$) δ 5.12 (d, *J* = 3.9 Hz, 1H), 4.41 (t, *J* = 3.6 Hz, 1H), 4.03 (ddd, *J* = 9.8, 3.5, 2.2 Hz, 1H), 3.65 (dd, *J* = 10.5, 3.5 Hz, 1H), 3.56 (dd, *J* = 10.1, 1.8 Hz, 1H), 3.54 – 3.47 (m, 1H), 3.44 (s, 6H), 3.41 (d, *J* = 3.6 Hz, 1H), 3.39 (s, 3H), 3.36 (t, *J* = 3.9 Hz, 1H), 2.07 – 1.95 (m, 2H), 1.80 (dq, *J* = 9.7, 3.0 Hz, 1H), 1.70 – 1.59 (m, 1H), 1.28 – 1.10 (m, 3H), 1.04 (tt, *J* = 12.3, 2.9 Hz, 1H), 0.88 (dd, *J* = 12.3, 3.5 Hz, 1H), 0.83 (s, 9H). **^{13}C NMR** (101 MHz, $CDCl_3$) δ 93.8, 77.3, 77.1, 75.6, 70.8, 65.8, 59.3, 57.1, 57.1, 56.8, 46.9, 34.8, 32.4, 31.4, 27.6 (3xC), 26.4, 24.2. **HRMS (ESI+) m/z** calc. for $C_{19}H_{35}N_3NaO_5$ $[M+Na]^+$: 408.2469, found 408.2468.

Compound R4.

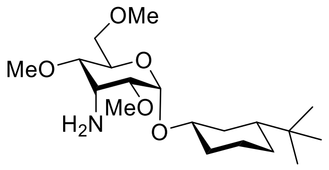

A solution of derivative **41α** (42 mg, 0.1 mmol) in THF (2 mL, 20 mL/mmol) was treated according to *general procedure XII*. The crude was purified by silica gel column chromatography (CH_2Cl_2/MeOH, 9:1) to give compound **R4** (34 mg, 87%). **^{1}H NMR** (400 MHz, CD_3OD) δ 5.05 (d, *J* = 3.7 Hz, 1H), 3.87 (ddd, *J* = 10.2, 5.3, 2.2 Hz, 1H), 3.67 (t, *J* = 4.0 Hz, 1H), 3.62 – 3.54 (m, 2H), 3.54 –

3.44 (m, 1H), 3.40 (s, 3H), 3.35 (s, 3H), 3.34 (s, 3H), 3.26 (d, *J* = 4.0 Hz, 1H), 3.16 (dd, *J* = 10.2, 3.8 Hz, 1H), 2.14 (ddd, *J* = 11.2, 4.3, 2.2 Hz, 1H), 2.05 – 1.97 (m, 1H), 1.81 (dt, *J* = 12.9, 3.3 Hz, 1H), 1.75 – 1.63 (m, 1H), 1.27 – 0.96 (m, 4H), 0.89-0.82 (m, 10H). **^{13}C NMR** (101 MHz, CD_3OD) δ 95.9, 78.6, 76.5, 76.0, 71.9, 65.4, 58.2, 55.4, 55.3, 46.9, 35.2, 31.8, 31.8 (2xC), 26.6 (3xC), 26.3, 24.0. **HRMS (ESI+) m/z** calc. for $C_{19}H_{38}NO_5$ $[M+H]^+$: 360.2744, found 360.2739.

Compound 42α.

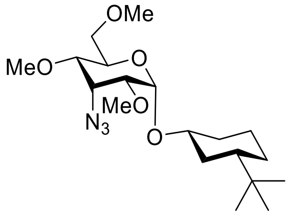

Compound **42α** was obtained by reaction of donor **D1** (286 mg, 0.8 mmol) and acceptor **A12** (170 mg, 1.1 mmol), following *general procedure VII*. The crude was purified by silica gel column chromatography (Hex/AcOEt 8:2) to yield compound **42α** (α-anomer: 55 mg, 19%, α/β ratio: 1:1). **^{1}H NMR** (400 MHz, $CDCl_3$) δ 5.09 (d, *J* = 4.0 Hz, 1H), 4.38 (t, *J* = 3.6 Hz, 1H), 3.99 (dt, *J* = 9.8, 2.7 Hz, 1H), 3.62 (dd, *J* = 10.5, 3.2 Hz, 1H), 3.54 (dd, *J* = 10.4, 2.2 Hz, 1H), 3.51 – 3.44 (m, 1H), 3.42 (s, 6H), 3.41 – 3.37 (m, 1H), 3.36 (s, 3H), 3.33 (t, *J* = 3.9 Hz, 1H), 2.02 (ddt, *J* = 11.2, 4.2, 2.2 Hz, 1H), 1.94 (dddd, *J* = 13.0, 4.7, 3.1, 1.6 Hz, 1H), 1.77 (dp, *J* = 13.4, 3.3 Hz, 1H), 1.66 – 1.59 (m, 1H), 1.31 (dddd, *J* = 14.7, 11.8, 10.8, 3.7 Hz, 1H), 1.23 – 1.12 (m, 1H), 1.10 – 0.93 (m, 2H), 0.86 (dd, *J* = 12.5, 3.6 Hz, 1H), 0.81 (s, 9H). **^{13}C NMR** (101 MHz, $CDCl_3$) δ 94.2, 77.7, 77.0, 75.5, 70.7, 65.7, 57.1, 59.2, 56.91, 56.86, 46.6, 33.5, 32.9, 32.4, 27.5 (3xC), 26.3, 24.5. **HRMS (ESI+) m/z** calc. for $C_{19}H_{35}N_3NaO_5$ $[M+Na]^+$: 408.2469, found 408.2468.

Compound R5.

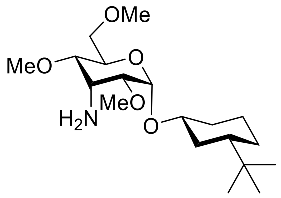

A solution of derivative **42α** (65 mg, 0.16 mmol) in THF (3 mL, 20 mL/mmol) was treated according to *general procedure XII*. The crude was purified by silica gel column chromatography (CH_2Cl_2/MeOH, 9:1) to give compound **R5** (56 mg, 93%). **^{1}H NMR** (400 MHz, CD_3OD) δ 5.05 (d, *J* = 3.7 Hz, 1H), 3.86 – 3.80 (m, 1H), 3.69 (t, *J* = 4.0 Hz, 1H), 3.59 – 3.55 (m, 2H), 3.54 – 3.45 (m, 1H), 3.41 (s, 3H), 3.35 (s, 3H), 3.34 (s, 3H), 3.32 – 3.24 (m, 1H), 3.19 (dd, *J* = 10.3, 3.8 Hz, 1H), 2.09 (ddq, *J* = 11.6, 4.7, 2.3 Hz, 1H), 2.05 – 1.97 (m, 1H), 1.80 (dq, *J* = 9.7, 3.6 Hz, 1H), 1.73 – 1.64 (m, 1H), 1.30 – 1.14 (m, 2H), 1.09 – 0.99 (m, 1H), 0.99 – 0.87 (m, 1H), 0.93 – 0.81 (m, 10H). **^{13}C NMR** (101 MHz, CD_3OD) δ 95.8, 78.3, 76.4, 75.7, 71.7, 65.3, 58.3, 55.4 (2xC), 47.0, 46.6, 33.7, 33.2, 31.9, 26.6 (3xC), 26.3, 24.2. **HRMS (ESI+) m/z** calc. for $C_{19}H_{38}NO_5$ $[M+H]^+$: 360.2744, found 360.2740.

Compound 43α.

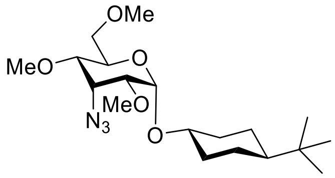

Compound **43α** was obtained by reaction of donor **D1** (200 mg, 0.6 mmol) and **A13** trans-4-*tert*-butylcyclohexanol (120 mg, 0.8 mmol), following *general procedure VII*. The crude was purified by column chromatography on silica gel (Hex/AcOEt 8:2) to give the compound **43α** (α-anomer: 61 mg, 27%; α/β ratio 1.1:1). **^{1}H NMR** (400 MHz, $CDCl_3$) δ 5.09 (d, *J* = 3.9 Hz, 1H), 4.39 (t, *J* = 3.6 Hz, 1H), 3.99 (ddd, *J* = 9.9, 3.3, 2.1 Hz, 1H), 3.63 (dd, *J* = 10.5, 3.3 Hz, 1H), 3.57 – 3.52 (m, 1H), 3.50 – 3.43 (m, 1H), 3.42 (s, 3H), 3.41 (m, 4H), 3.41 – 3.38 (m, 1H), 3.38 (s, 3H), 3.37 – 3.34 (m, 1H), 2.07 – 1.97 (m, 2H), 1.81 – 1.72 (m, 2H), 1.48 – 1.20 (m, 2H), 1.07 – 0.91 (m, 3H), 0.81 (d, *J* = 1.4 Hz, 9H). **^{13}C NMR** (101 MHz, $CDCl_3$) δ 94.0, 77.2, 76.9, 75.5, 70.8, 65.8, 59.3, 57.1, 56.9, 56.8, 47.2, 33.7, 32.3, 31.7, 27.6, 25.8, 25.5 (3xC). **HRMS (ESI+) m/z** calc. for $C_{19}H_{36}N_3O_5$ $[M+H]^+$: 386.2578, found 380.2593.

Compound R6.

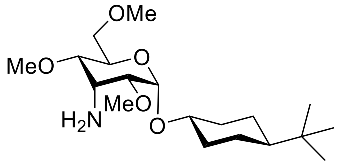

A solution of derivative **43α** (60 mg, 0.16 mmol) in THF (2.5 mL, 15 mL/mmol) was treated according to *general procedure XII*. The crude was purified by silica gel column chromatography (CH_2Cl_2/MeOH, 9:1) to give compound **R6** (51 mg, 91%). **^{1}H NMR** (400 MHz, CD_3OD) δ 5.06 (d, *J* = 3.7 Hz, 1H), 3.88 – 3.79 (m, 1H), 3.70 (t, *J* = 4.0 Hz, 1H), 3.61 – 3.56 (m, 2H), 3.52 – 3.43 (m, 1H), 3.41 (s, 3H), 3.37 (s, 3H), 3.35 (s, 3H), 3.28 (d, *J* = 4.0 Hz, 1H), 3.21 (dd, *J* = 10.3, 3.9 Hz, 1H), 2.07 (ddt, *J* = 12.4, 7.0, 3.2 Hz, 2H), 1.88 – 1.73 (m, 2H), 1.39 – 0.94 (m, 5H), 0.85 (s, 9H). **^{13}C NMR** (101 MHz, CD_3OD) δ 95.3, 77.3, 76.2, 75.6, 71.6, 65.3, 58.2, 55.5, 55.4, 47.3, 47.0, 33.8, 31.73, 31.69, 26.7, 25.5, 25.3 (3xC). **HRMS (ESI+) m/z** calc. for $C_{19}H_{38}NO_5$ $[M+H]^+$: 360.2744, found 360.2743.

Compound 44α.

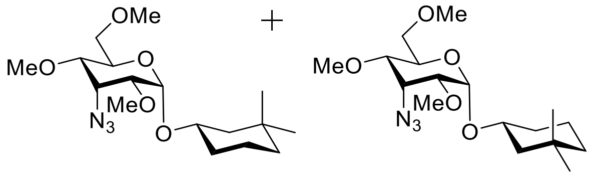

Compound **44α** was obtained by reaction of donor **D1** (300 mg, 0.884 mmol) and acceptor **A14** (147 mg, 1.2 mmol), following *general procedure VII*. The crude was purified by column chromatography on silica gel (Hex/AcOEt 7:3) to yield **44α** (α-anomer: 102 mg, 32%, α/β ratio: 1:1). Since the starting acceptor is a racemic mixture, the result of the glycosylation is two α-diastereoisomers that could not be separated in this step. **HRMS (ESI+) m/z** calc. for $C_{17}H_{32}N_3O_5$ $[M+H]^+$: 358.2364, found 358.2345.

Compound R7.

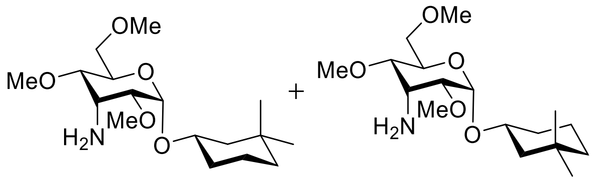

A solution of derivative **44α** (20 mg, 0.06 mmol) in THF (1 mL, 15 mL/mmol) was treated according to *general procedure XII*. The crude was purified by silica gel column chromatography (CH_2Cl_2/MeOH, 9:1) to give compound **R7** as a mixture of diastereomeric amines that could not be solved (15 mg, 83%). **HRMS (ESI+) m/z** calc. for $C_{17}H_{34}NO_5$ $[M+H]^+$: 332.2431, found 332.2429.

Compound 45α.

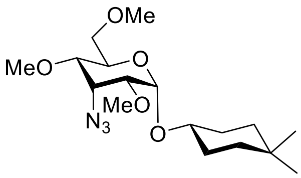

Compound **45α** was obtained by reaction of donor **D1** (300 mg, 0.884 mmol) and acceptor **A15** (147 mg, 1.15 mmol), following *general procedure VII*. The crude was purified by column chromatography on silica gel (Hex/AcOEt 7:3) to yield compound **45α** (α-anomer: 160 mg, 51%, α/β ratio: 1.1:1). **^{1}H NMR** (500 MHz, $CDCl_3$) δ 5.08 (dd, *J* = 4.0, 0.8 Hz, 1H), 4.42 (t, *J* = 3.6 Hz, 1H), 4.00 (dt, *J* = 9.8, 2.7 Hz, 1H), 3.63 (dd, *J* = 10.5, 3.2 Hz, 1H), 3.59 – 3.52 (m, 2H), 3.44 (s, 3H), 3.41 (s, 3H), 3.39 – 3.35 (m, 5H), 1.76 – 1.61 (m, 3H), 1.61 – 1.51 (m, 1H), 1.47 (ddt, *J* = 13.2, 5.5, 2.6 Hz, 2H), 1.15 (dddd, *J* = 17.8, 14.2, 10.7, 4.1 Hz, 2H), 0.90 (s, 3H), 0.87 (d, *J* = 0.8 Hz, 3H). **^{13}C NMR** (126 MHz, $CDCl_3$) δ 94.2, 77.5, 76.0, 75.3, 70.8, 65.9, 59.3, 57.1, 56.82, 56.77, 36.6, 36.3, 30.0, 29.7, 29.0, 27.2, 26.6. **HRMS (ESI+) m/z** calc. for $C_{17}H_{31}N_3NaO_5$ $[M+Na]^+$: 380.2156, found 380.2149.

Compound R8.

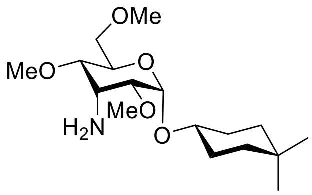

A solution of derivative **45α** (40 mg, 0.1 mmol) in THF (2 mL, 20 mL/mmol) was treated according to *general procedure XII*. The crude was purified by silica gel column chromatography (CH_2Cl_2/MeOH, 9:1) to give compound **R8** (30 mg, 81%). **^{1}H NMR** (400 MHz, CD_3OD) δ 5.06 (d, *J* = 3.6 Hz, 1H), 3.86 (dt, *J* = 10.3, 3.5 Hz, 1H), 3.72 (t, *J* = 4.0 Hz, 1H), 3.67 – 3.58 (m, 3H), 3.44 (s, 3H), 3.39 (s, 3H), 3.38 (s, 3H), 3.32 – 3.29 (m, 1H), 3.22 (dd, *J* = 10.3, 3.8 Hz, 1H), 1.78 (tdd, *J* = 14.3, 7.2, 3.4 Hz, 2H), 1.70 – 1.40 (m, 4H), 1.24 (dddt, *J* = 17.7, 13.7, 10.0, 4.5 Hz, 2H), 0.95 (s, 3H), 0.93 (s, 3H). **^{13}C NMR** (101 MHz, CD_3OD) δ 95.5, 76.5, 75.8 (2xC), 71.7, 65.4, 58.3, 55.5, 55.4, 47.0, 36.2, 35.8, 29.3, 28.8, 28.6, 26.6, 26.3. **HRMS (ESI+) m/z** calc. for $C_{17}H_{34}NO_5$ $[M+H]^+$: 332.2431, found 332. 2431.

Compound 46.

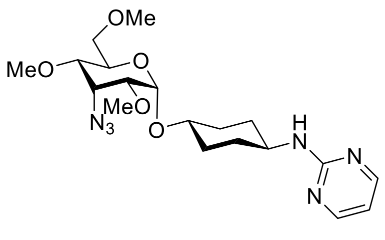

A solution of derivative **18** (55 mg, 0.16 mmol) in EtOH (1 mL) was treated according to *general procedure XV*. The resulting crude was purified by silica gel column chromatography (Hex/AcOEt, 7:3) to yield compound **46** (59 mg, 88%). **^{1}H NMR** (400 MHz, CD_3OD) δ 8.14 (d, *J* = 4.8 Hz, 2H), 6.46 (t, *J* = 4.8 Hz, 1H), 4.97 (d, *J* = 4.0 Hz, 1H), 4.44 (t, *J* = 3.6 Hz, 1H), 3.84 (dt, *J* = 10.0, 3.5 Hz, 1H), 3.71 – 3.62 (m, 1H), 3.58 – 3.42 (m, 3H), 3.36 (s, 3H), 3.34 (d, *J* = 4.3 Hz, 1H), 3.32 (s, 3H), 3.27 (s, 3H), 3.26 – 3.22 (m, 1H), 1.97 (t, *J* = 7.6 Hz, 4H), 1.56 – 1.17 (m, 4H). **^{13}C NMR** (101 MHz, CD_3OD) δ 171.6, 161.6, 157.9, 109.7, 94.4, 76.9, 75.9, 75.3, 71.2, 66.1, 58.2, 56.7, 55.7, 55.7, 48.8, 31.5, 30.1, 29.8, 29.6. **HRMS (ESI+) m/z** calc. for $C_{19}H_{31}N_6O_5$ $[M+H]^+$: 423.2350, found 423.2339.

Compound R9.

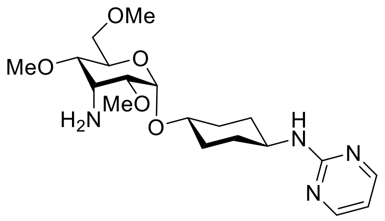

A solution of derivative **46** (48 mg, 0.11 mmol) in THF (2 mL) was treated according to *general procedure XII*. The crude was purified by silica gel column chromatography (CH_2Cl_2/MeOH, 9:1) to give compound **R9** (36 mg, 78%). **^{1}H NMR** (400 MHz, CD_3OD) δ 8.21 (d, *J* = 4.9 Hz, 2H), 6.54 (t, *J* = 4.8 Hz, 1H), 5.07 (d, *J* = 3.7 Hz, 1H), 3.89 – 3.80 (m, 1H), 3.78 – 3.68 (m, 2H), 3.66 – 3.55 (m, 3H), 3.42 (s, 3H), 3.36 (s, 3H), 3.36 (s, 3H), 3.31 (d, *J* = 3.9 Hz, 1H), 3.23 (dd, *J* = 10.2, 3.9 Hz, 1H), 2.04 (ddd, *J* = 15.4, 9.6, 5.8 Hz, 4H), 1.49 – 1.22 (m, 4H). **^{13}C NMR** (101 MHz, CD_3OD) δ 161.6, 158.0 (2xC), 109.8, 95.7, 76.3, 76.1, 75.4, 71.6, 65.4, 58.3, 55.6, 55.5, 48.8, 47.2, 31.7, 30.1, 29.9, 29.8. **HRMS (ESI+) m/z** calc. for $C_{19}H_{33}N_4O_5$ $[M+H]^+$: 397.2445, found 397.2445.

Compound 47.

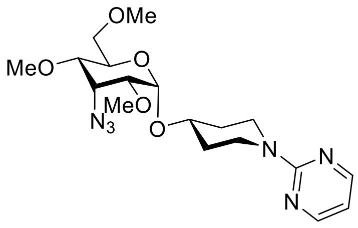

A solution of derivative **28** (50 mg, 0.15 mmol) in EtOH (1 mL) was treated according to *general procedure XV*. The resulting crude was purified by silica gel column chromatography (Hex/AcOEt, 7:3) to yield compound **47** (59 mg, 88%). **^{1}H NMR** (400 MHz, $CDCl_3$) δ 8.27 (d, *J* = 4.7 Hz, 2H), 6.42 (t, *J* = 4.7 Hz, 1H), 5.14 (d, *J* = 3.9 Hz, 1H), 4.46 (t, *J* = 3.5 Hz, 1H), 4.24 – 4.12 (m, 2H), 4.02 (dt, *J* = 9.8, 2.7 Hz, 1H), 3.91 (tt, *J* = 7.6, 3.7 Hz, 1H), 3.66 –

3.52 (m, 4H), 3.44 (s, 3H), 3.42 (s, 3H), 3.41 (dd, J = 6.2, 3.7 Hz, 1H), 3.39 (s, 3H), 3.36 (dd, J = 6.22, 3.5 Hz, 1H), 1.95 – 1.83 (m, 1H), 1.83 – 1.64 (m, 3H). **^{13}C NMR** (101 MHz, $CDCl_3$) δ 161.6, 157.7 (2xC), 109.4, 94.7, 77.7, 75.1, 73.8, 70.8, 66.3, 59.4, 57.1, 56.9, 56.5, 41.2, 41.0, 32.1, 30.3. **HRMS (ESI+) m/z** calc. for $C_{18}H_{29}N_6O_5$ $[M+H]^+$: 409.2194, found 409.2194.

Compound R10a.

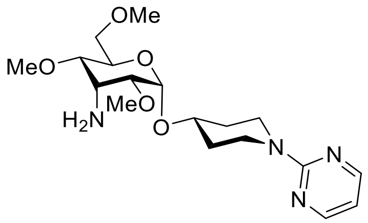

A solution of derivative **47** (25 mg, 0.06 mmol) in THF (1 mL) was treated according to *general procedure XII*. The crude was purified by silica gel column chromatography (CH_2Cl_2/MeOH, 9:1) to give compound **R10a** (21 mg, 91%). **^{1}H NMR** (400 MHz, $CDCl_3$) δ 8.22 (d, J = 4.8 Hz, 2H), 6.38 (t, J = 4.8 Hz, 1H), 5.07 (d, J = 3.7 Hz, 1H), 4.26 – 4.10 (m, 2H), 3.91 – 3.78 (m, 2H), 3.67 (t, J = 4.0 Hz, 1H), 3.59 – 3.52 (m, 2H), 3.41 (td, J = 9.1, 4.5 Hz, 2H), 3.37 (s, 3H), 3.34 (s, 3H), 3.32 (s, 3H), 3.23 (t, J = 4.0 Hz, 1H), 3.17 (dd, J = 10.2, 3.8 Hz, 1H), 1.93 – 1.82 (m, 2H), 1.71 – 1.53 (m, 2H). **^{13}C NMR** (101 MHz, $CDCl_3$) δ 161.6, 157.7 (2xC), 109.6, 96.0, 76.9, 75.9, 74.0, 71.5, 65.4, 59.4, 56.5, 56.4, 47.8, 41.5, 41.2, 32.3, 30.3. **HRMS (ESI+) m/z** calc. for $C_{18}H_{31}N_4O_5$ $[M+H]^+$: 383.2289, found 383.2290.

Compound 48.

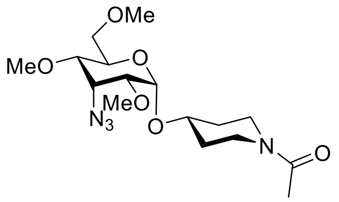

A solution of derivative **28** (50 mg, 0.15 mmol) in CH_2Cl_2 (3 mL) was treated according to *general procedure XVI*. The crude was purified by silica gel column chromatography (Hex/AcOEt, 1:1) to give compound **48** (42 mg, 76%). **^{1}H NMR** (400 MHz, $CDCl_3$) δ 5.07 (dd, J = 10.8, 3.6 Hz, 1H), 4.47 (q, J = 3.2 Hz, 1H), 3.97 (t, J = 9.4 Hz, 1H), 3.89 (s, 1H), 3.70 (s, 2H), 3.63 – 3.59 (m, 2H), 3.57 – 3.49 (m, 1H), 3.46 – 3.38 (m, 7H), 3.37 (s, 3H), 3.36 - 3.32 (m, 2H), 2.07 (s, 3H), 1.76 (s, 4H). **^{13}C NMR** (101 MHz, $CDCl_3$) δ 169.2, 94.4, 77.7, 74.9, 73.1, 71.9, 70.7, 66.4, 59.4, 57.0, 56.1, 43.2, 38.2, 31.3, 29.9, 21.5. **HRMS (ESI+) m/z** calc. for $C_{16}H_{29}N_4O_6$ $[M+H]^+$: 373.2082, found 373.2076.

Compound R10.

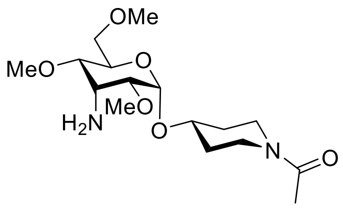

A solution of derivative **48** (42 mg, 0.11 mmol) in THF (2 mL) was treated according to *general procedure XII*. The crude was purified by silica gel column chromatography (CH_2Cl_2/MeOH, 9:1) to give compound **R10** (31

mg, 82%). **^{1}H NMR** (400 MHz, CD_3OD) δ 5.09 (t, *J* = 3.4 Hz, 1H), 3.95 – 3.84 (m, 2H), 3.84 – 3.65 (m, 3H), 3.65 – 3.54 (m, 2H), 3.50 – 3.41 (m, 4H), 3.40 – 3.35 (m, 7H), 3.33 (t, *J* = 4.0 Hz, 1H), 3.22 (dd, *J* = 10.2, 3.8 Hz, 1H), 2.10 (s, 3H), 2.01 – 1.53 (m, 4H). **^{13}C NMR** (101 MHz, CD_3OD) δ 170.1, 95.9, 76.3, 75.7, 72.8, 71.7, 65.6, 58.2, 55.5, 55.4, 46.8, 38.6, 38.3, 30.3, 29.2, 19.8. **HRMS (ESI+) m/z** calc. for $C_{16}H_{31}N_2O_6$ $[M+H]^+$: 347.2177, found 347.2171.

1.7 Synthesis of aromatic systems: aryl-acetaldehydes and 2-aryl-propionaldehydes

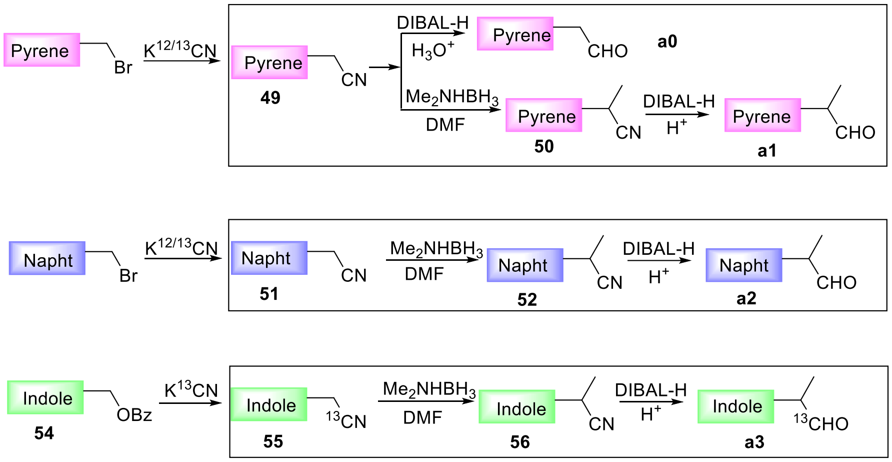

Scheme 1.4.- *Synthetic route for the preparation of aryl-acetaldehyde **a0** and 2-aryl-propionaldehydes **a1**, **a2** and **a3** with and without ^{13}C isotopic labeling.*

Compound 49

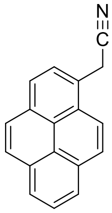

Commercially available 1-bromomethylpyrene (240 mg, 0.8 mmol) was dissolved in anhydrous CH_2Cl_2 (10 mL) and treated with KCN (104 mg, 1.6 mmol), according to *general procedure XVII*. The resulting crude was purified on silica gel column chromatography (Hex/AcOEt, 9:1) to yield compound **49** (175 mg, 89%). The spectroscopic data obtained are in agreement with those published in the bibliography.[10]

Compound 49-^{13}C

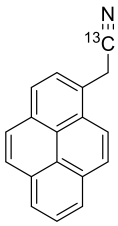

Commercially available 1-bromomethylpyrene (294 mg, 1.0 mmol) was dissolved in anhydrous CH_2Cl_2 (10 mL) and treated with $K^{13}CN$ (71 mg, 1.1 mmol), according to *general procedure XVII*. The resulting crude was purified on silica gel column chromatography

(Hex/AcOEt, 9:1) to yield compound **49-^{13}C** (220 mg, 91%). The spectroscopic data obtained are in agreement with those published in the bibliography. **HRMS (ESI+) m/z** calc. for $C_{17}{}^{13}CH_{14}N$ $[M+H]^+$: 245.1081, found 245.1087.

Compound a0

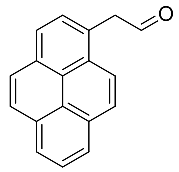

Derivative **a0** was obtained from compound **49** (175 mg, 0.72 mmol) following the guidelines of the *general procedure XVIII* for nitrile reduction. Silica gel column chromatography of the crude (Hex/AcOEt, 9:1) yielded the desired compound **a0** (^{12}C) (80 mg, 46%). The spectroscopic data obtained are in agreement with those published in the bibliography.[11]

Compound a0-^{13}C

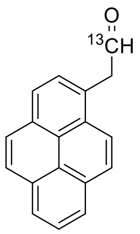

Derivative **a0-^{13}C** was obtained from compound **49-^{13}C** (67 mg, 0.25 mmol) following the guidelines of the *general procedure XVIII* for nitrile reduction. Silica gel column chromatography of the crude (Hex/AcOEt, 9:1) yielded the desired compound **a0-^{13}C** (34 mg, 53%). **^{1}H NMR** (400 MHz, $CDCl_3$) δ 9.89 (dt, $^3J_{H-H}$ = 4.8, 4.8 Hz, $^1J_{H-13C}$ = 176.5 Hz, 1H), 8.25 – 7.89 (m, 9H), 4.40 (dd, *J* = 7.3, 2.4 Hz, 2H). **^{13}C NMR** (101 MHz, $CDCl_3$) δ 199.4 (^{13}CHO), 131.4, 131.2, 130.8, 129.9, 128.6, 128.5, 127.7, 127.5, 126.3, 125.7, 125.6, 125.4, 125.2, 124.8, 122.9, 118.0, 48.9 (d, *J* = 37.4 Hz). **HRMS (ESI+) m/z** calc. for $C_{17}{}^{13}CH_{15}N$ $[M+H]^+$: 248.1077, found 248.1074.

Compound 50

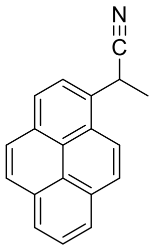

Compound **50** was obtained from a solution of compound **49** (50 mg, 0.2 mmol) in dry DMF (1 mL, 5 mL/mmol), following the guidelines of *general procedure XIX*. The resulting crude material was purified through a silica gel column chromatography (Hex/AcOEt, 9:1) to provide compound **50** (46 mg, 88%). **^{1}H NMR** (400 MHz, $CDCl_3$) δ 8.27 – 7.99 (m, 9H), 4.91 (qd, *J* = 7.2, 1.5 Hz, 1H), 1.88 (dd, *J* = 7.3, 1.5 Hz, 3H). **^{13}C NMR** (101 MHz, $CDCl_3$) δ 131.4, 131.3, 130.6, 130.1, 128.8, 128.0, 127.4, 126.4, 126.1, 125.9, 125.6, 125.4, 125.2, 124.8, 124.6, 122.2, 121.3, 28.8, 21.5. **HRMS (ESI+) m/z** calc. for $C_{19}H_{14}N$ $[M+H]^+$: 256.1205, found 256.1202.

Compound 50-^{13}C

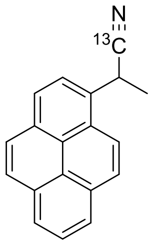

Compound **50-^{13}C** was obtained from a solution of compound **49-^{13}C** (100 mg, 0.4 mmol) in dry DMF (2 mL, 5 mL/mmol) following the guidelines of *general procedure XIX*. The resulting crude material was purified through a silica gel column chromatography (Hex/AcOEt, 9:1) to provide compound **50-^{13}C** (94 mg, 90%). **HRMS (ESI+) m/z** calc. for $C_{18}{}^{13}CH_{13}NNa$ $[M+Na]^+$: 279.0974, found 279.0975.

Compound a1

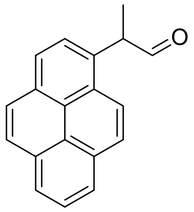

Derivative **a1** was obtained from compound **50** (46 mg, 0.18 mmol) following the guidelines of the *general procedure XVIII* for nitrile reduction. Silica gel column chromatography of the crude (Hex/AcOEt, 9:1) yielded the desired compound **a1** (32 mg, 69%). **^{1}H NMR** (400 MHz, $CDCl_3$) δ 9.90 (d, *J* = 1.2 Hz, 1H), 8.31 – 8.13 (m, 5H), 8.13 – 8.00 (m, 3H), 7.81 (d, *J* = 7.9 Hz, 1H), 4.68 (q, *J* = 7.0 Hz, 1H), 1.72 (d, *J* = 6.9 Hz, 3H). **^{13}C NMR** (101 MHz, $CDCl_3$) δ 201.3, 131.7, 131.5, 130.9, 130.8, 129.4, 128.5, 127.7, 127.5, 126.3, 125.7, 125.6, 125.4, 125.39, 125.36, 124.9, 122.4, 49.5, 15.3. **HRMS (ESI+) m/z** calc. for $C_{19}H_{15}O$ $[M+H]^+$: 259.1101, found 259.1106.

Compound a1-^{13}C

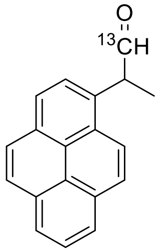

To obtain isotopically labeled **a1-^{13}C** from compound **50-^{13}C** (94 mg, 0.37 mmol), the guidelines of the *general procedure XIX* for nitrile reduction were followed. Silica gel column chromatography of the crude (Hex/AcOEt, 9:1) yielded the desired compound **a1-^{13}C** (36 mg, 38%). **HRMS (ESI) m/z** calc. for $C_{18}{}^{13}CH_{15}O$ $[M+H]^+$: 259.1156, found 259.1161.

Compound 51

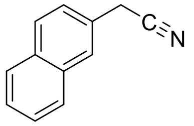

A solution of commercially available 2-methylnaphthalene bromide (180 mg, 0.8 mmol) in a 2:1 mixture of CH_2Cl_2/H_2O (1.9 mL, 2.4 mL/mmol) was treated according to the guidelines of *general procedure XVII*, but employing tetrabutylammonium cyanide (240 mg, 0.9 mmol) instead of KCN. The crude was purified by silica gel column chromatography (Hex/AcOEt, 9:1) to provide compound **51** (130 mg, 96%). The spectroscopic data obtained agree with those published in the bibliography.[12]

Compound 51-^{13}C

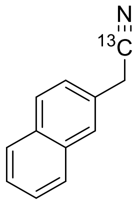

Isotopically labeled compound **51-^{13}C** was synthesized analogously to derivative **51**, prepared from commercially available 2-methylnaphthalene bromide (180 mg, 0.8 mmol). The reaction crude was purified by silica gel column chromatography (Hex/AcOEt, 9:1) to yield compound **51-^{13}C** (110 mg, 81%). The spectroscopic data obtained agree with those published in the bibliography.[12]

Compound 52.

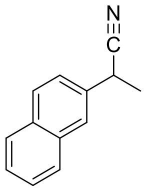

Compound **52** was obtained from a solution of compound **51** (50 mg, 0.3 mmol) in dry DMF (1.5 mL, 5 mL/mmol), following the guidelines of *general procedure XIX*. The resulting crude material was purified through a silica gel column chromatography (Hex/AcOEt, 9:1) to provide compound **52** (26 mg, 48%). The spectroscopic data obtained agree with those published in the bibliography.[13]

Compound 52-^{13}C

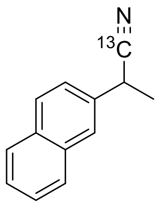

Isotopically labeled compound **52-^{13}C** was synthesized analogously to derivative **52**, prepared from nitrile **51-^{13}C** (95 mg, 0.6 mmol) following the guidelines of *general procedure XIX* for the α-methylation of benzylic nitriles. The reaction crude was purified by silica gel column chromatography (Hex/Toluene, 3:7) to yield compound **52-^{13}C** (26 mg, 25%).

Compound a2.

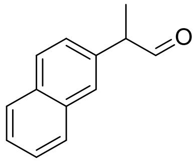

Derivative **a2** was obtained from compound **52** (20 mg, 0.11 mmol) following the guidelines of the *general procedure XVIII* for nitrile reduction. Silica gel column chromatography of the crude (Hex/AcOEt, 8:2) yielded the desired compound **a2** (10 mg, 50%). The spectroscopic data obtained agree with those published in the bibliography.[14]

Compound a2-^{13}C

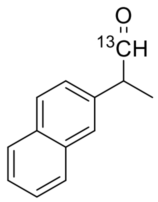

To obtain isotopically labeled **a2-^{13}C** from compound **52-^{13}C** (25 mg, 0.14 mmol), the guidelines of the *general procedure XIX* for nitrile reduction were followed. Silica gel column chromatography of the crude (Hex/AcOEt, 8:2) yielded the desired compound **a2-^{13}C** (15 mg, 60%). The spectroscopic data obtained agree with those published in the bibliography.[15]

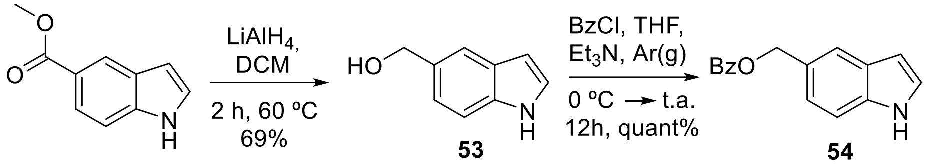

***Scheme 1.5.-** Preparation of synthetic intermediate **54**.*

Compound 53.

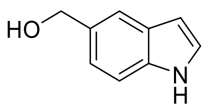

To a solution of commercially available methyl 5-indolecarboxylate (600 mg, 3.2 mmol) in anhydrous THF (3 mL, 1 mL/mmol), $LiAlH_4$ (301 mg, 7.9 mmol) was added at 0 °C. Subsequently, the reaction was stirred at 65 ºC for approximately 2 h. Then, it was cooled again to 0 ºC and diluted with Et_2O. Subsequently, it was washed sequentially with H_2O, 0.1M NaOH and again with H_2O. A white precipitate forms, which was filtered off and washed with Et_2O. The combined organic phases were evaporated under reduced pressure to give compound **53** (320 mg, 69%). The spectroscopic data obtained agree with those published in the bibliography.[16]

Compound 54.

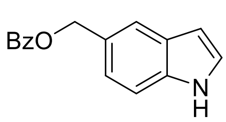

To a solution of alcohol **53** (320 mg, 2.2 mmol) in anhydrous THF was added, at 0 °C, benzoyl chloride (0.3 mL, 2.6 mmol) and Et_3N (0.9 mL, 6.5 mmol) under argon atmosphere. The reaction mixture was stirred at rt overnight. Subsequently, it was diluted with CH_2Cl_2 and washed with a saturated solution of $NaHCO_3$ and H_2O. The organic phase was dried over anhydrous $MgSO_4$ and the solvent was evaporated under reduced pressure to give crude compound **54** (546 mg, quant.). The spectroscopic data obtained agree with those published in the bibliography.[17]

Compound 55-^{13}C

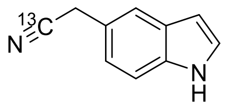

A solution of derivative **54** (272 mg, 1 mmol) in anhydrous DMF (5 mL, 5 mL/mmol) was treated with K^{13}CN (142 mg, 2.2 mmol). The reaction mixture was stirred at 105 °C under microwave irradiation for 5 h. The resulting mixture was evaporated under reduced pressure and the crude purified on a silica gel column chromatography (Hex/AcOEt, 8:2) to obtain compound **55-^{13}C** (57 mg, 34%). **HRMS (ESI+) m/z** calc. for $C_9{}^{13}CH_9N_2$ $[M+H]^+$: 158.0794, found 158.0792.

Compound 56-^{13}C

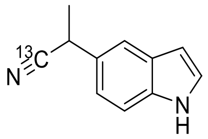

Compound **56-^{13}C** was obtained from a solution of compound **55-^{13}C** (36 mg, 0.23 mmol) in dry DMF (2.3 mL, 10 mL/mmol) following the guidelines of *general procedure XIX*. The resulting crude material was purified through a silica gel column chromatography (Hex/Toluene, 3:7) to provide compound **56-^{13}C** (10 mg, 26%). **^{1}H NMR** (400 MHz, $CDCl_3$) δ 8.23 (s, 1H), 7.62 (d, *J* = 1.8 Hz, 1H), 7.45 – 7.35 (m, 1H), 7.26 – 7.25 (m, 1H), 7.20 – 7.10 (m, 1H), 6.55 (td, *J* = 2.1, 1.0 Hz, 1H), 3.99 (dq, *J* = 10.1, 7.3 Hz, 1H), 1.68 (ddd, *J* = 7.2, 5.8, 2.6 Hz, 3H). **^{13}C NMR** (101 MHz, $CDCl_3$) δ 135.4, 128.8, 128.3, 125.4, 122.6, 120.9, 118.7, 111.8, 102.9, 31.2, 22.2. **HRMS (ESI+) m/z** calc. for $C_{10}{}^{13}CH_{11}N_2$ $[M+H]^+$: 171.0881, found 171.0877.

Compound a3-^{13}C

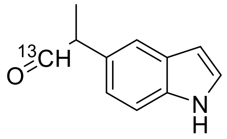

To obtain isotopically labeled **a6-^{13}C** from compound **56-^{13}C** (10 mg, 0.06 mmol), the guidelines of the *general procedure XVIII* for nitrile reduction were followed. Silica gel column chromatography of the crude (Hex/AcOEt, 8:2) yielded the desired compound **a6-^{13}C** (3 mg, 30%). **^{1}H NMR** (500 MHz, $CDCl_3$) δ 9.72 (dt, $^3J_{H-H}$ = 1.5 Hz, 1.5 Hz, $^1J_{H-13C}$ = 170.2 Hz, 1H), 8.18 (sa, 1H), 7.49 (dp, *J* = 1.5, 0.7 Hz, 1H), 7.41 (dt, *J* = 8.2, 0.9 Hz, 1H), 7.24 (ddd, *J* = 3.4, 2.5, 0.4 Hz, 1H), 7.03 (dd, *J* = 8.3, 1.8 Hz, 1H), 6.55 (ddd, *J* = 3.1, 2.0, 0.9 Hz, 1H), 3.72 (pd, *J* = 7.0, 1.5 Hz, 1H), 1.49 (dd, *J* = 7.0, 5.0 Hz, 3H). **^{13}C NMR** (126 MHz, $CDCl_3$) δ 201.7, 135.2, 129.0, 128.5, 124.9, 122.5, 120.4, 111.6, 102.6, 53.1 (d, ^1J = 37.2 Hz), 15.0. **HRMS** (ESI+): calc. for $C_{10}{}^{13}CH_{12}NO$ $[M+H]^+$: 175.0947, found 175.0947.

2. Copies of the ^{1}H and $^{13}C\{^{1}H\}$ NMR spectra of purified synthetic intermediates

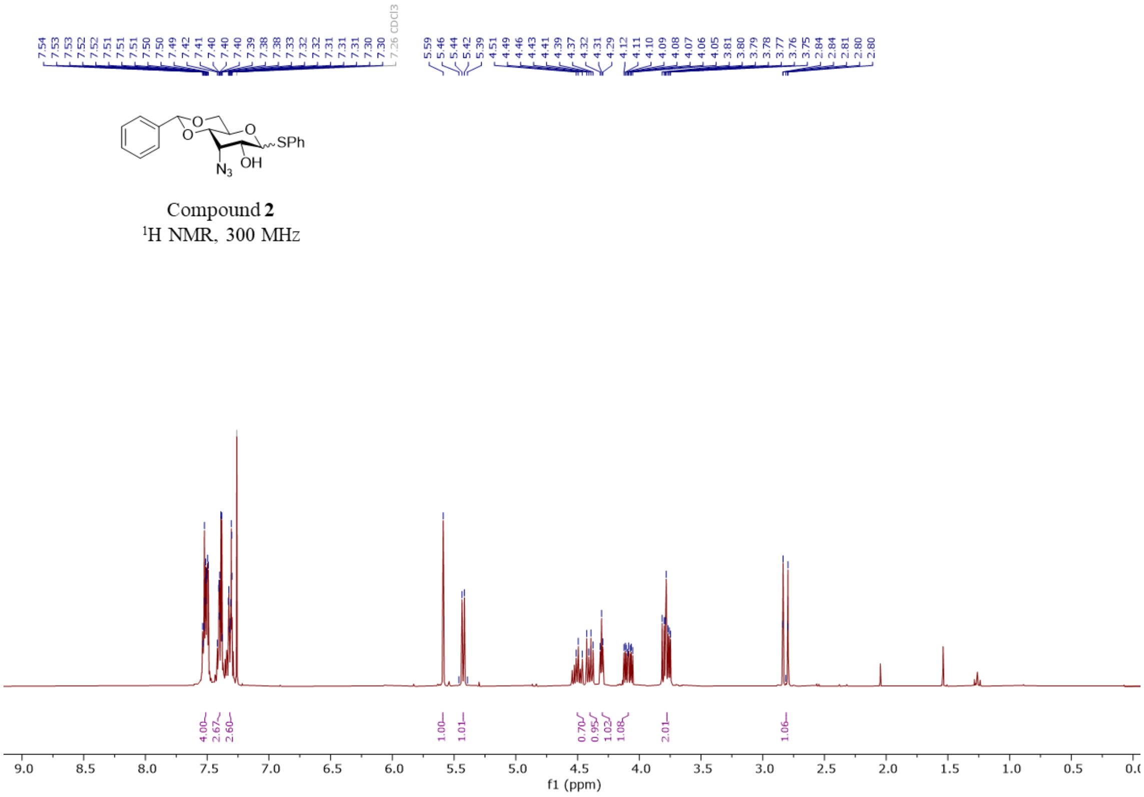

Compound **2**
^{1}H NMR, 300 MHz

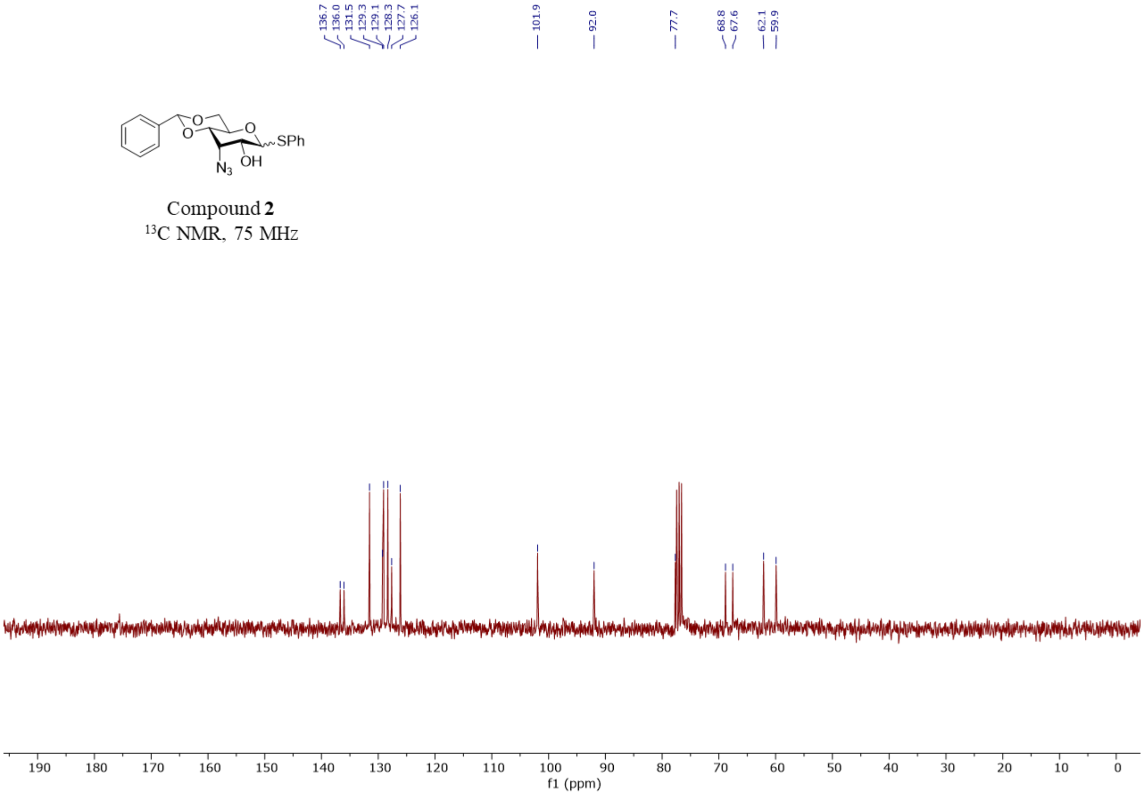

Compound **2**
^{13}C NMR, 75 MHz

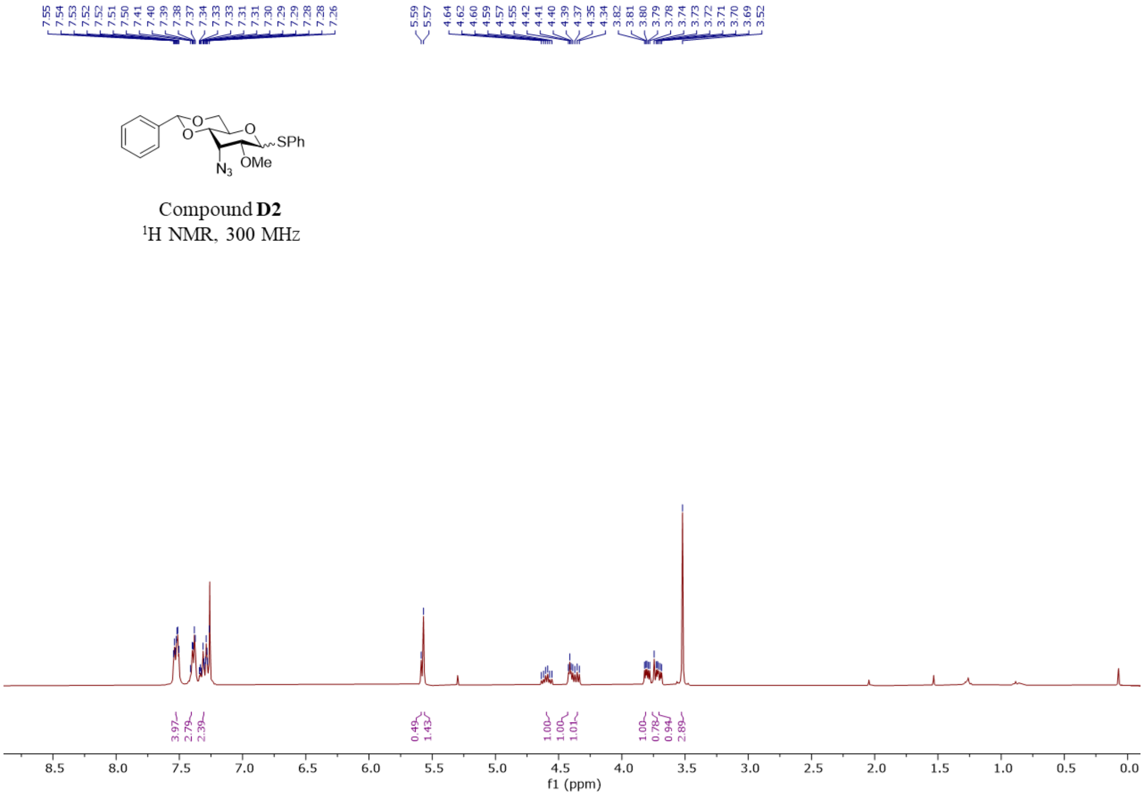

Compound **D2**
^{1}H NMR, 300 MHz

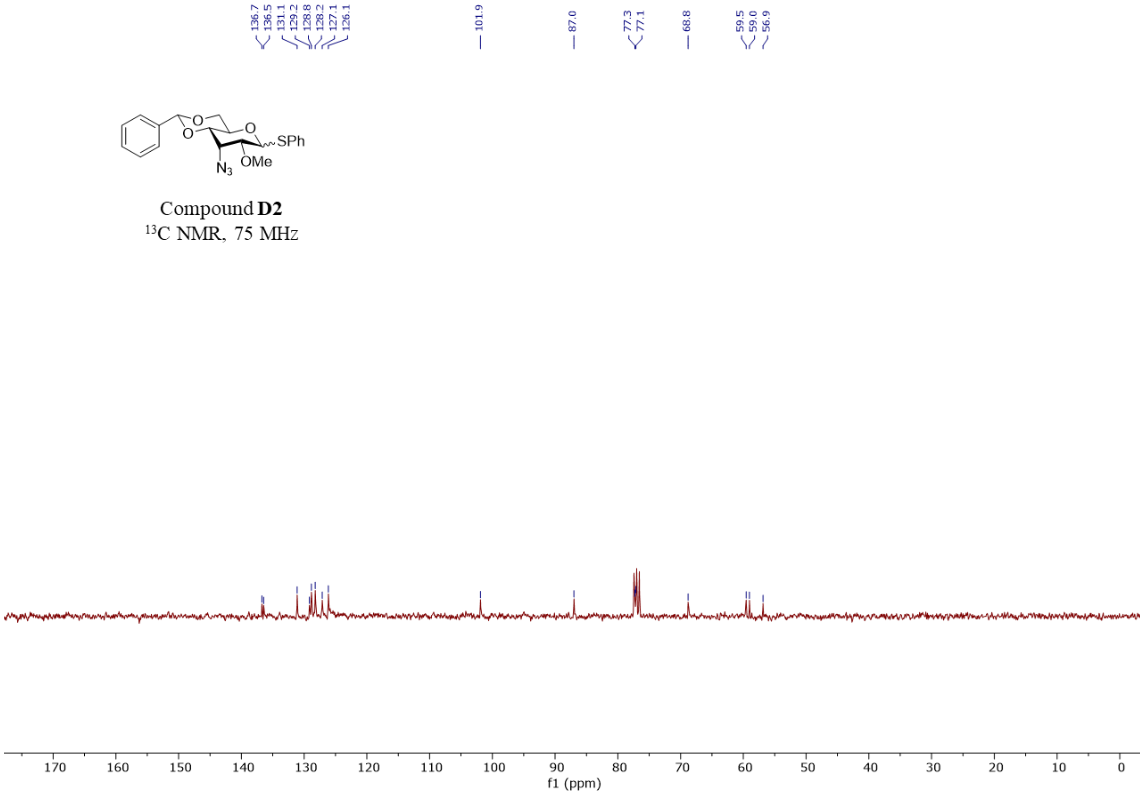

Compound **D2**
^{13}C NMR, 75 MHz

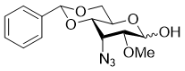

Compound **3**
^{1}H NMR, 300 MHz

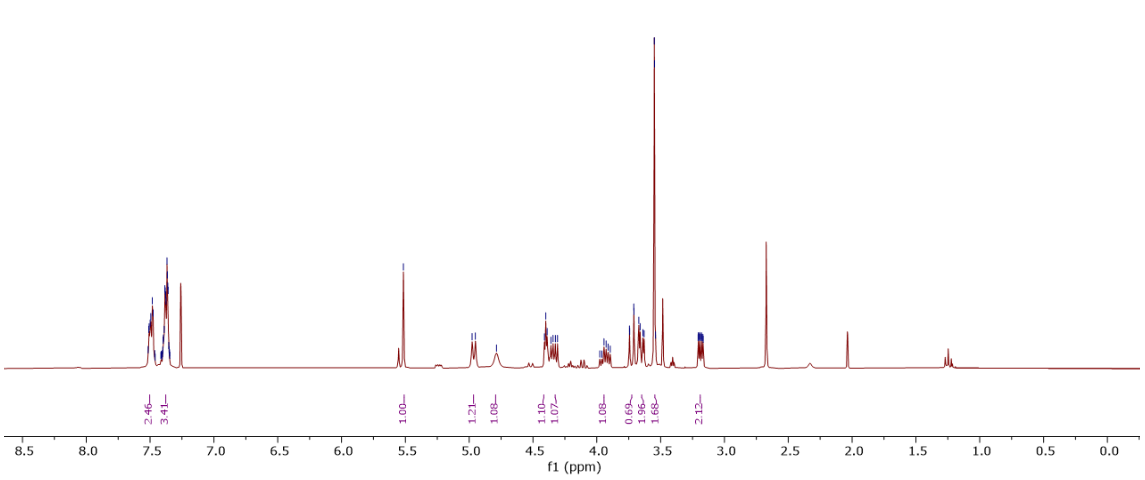

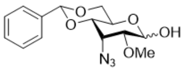

Compound **3**
^{13}C NMR, 75 MHz

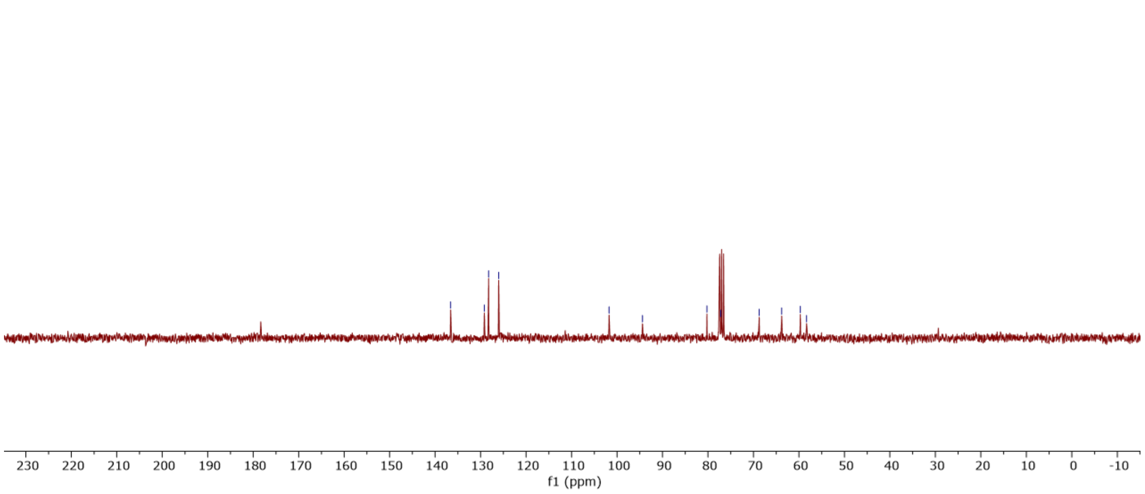

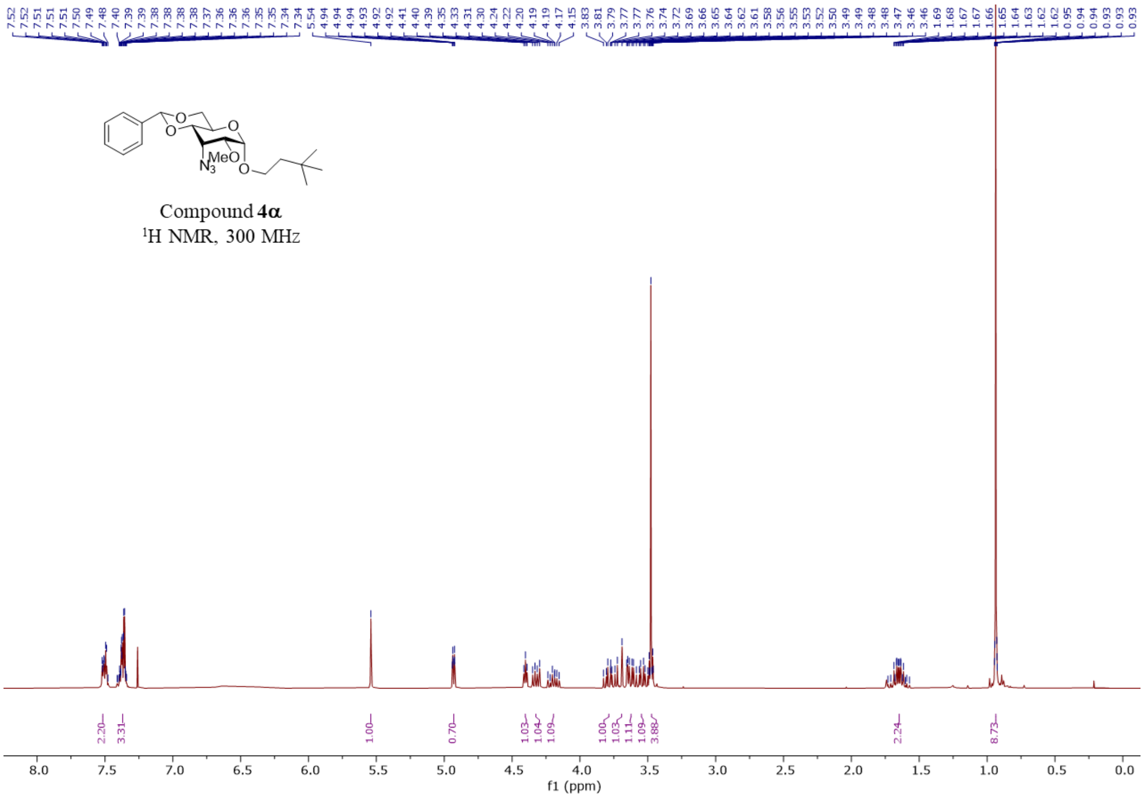

Compound **4α**
^{1}H NMR, 300 MHz

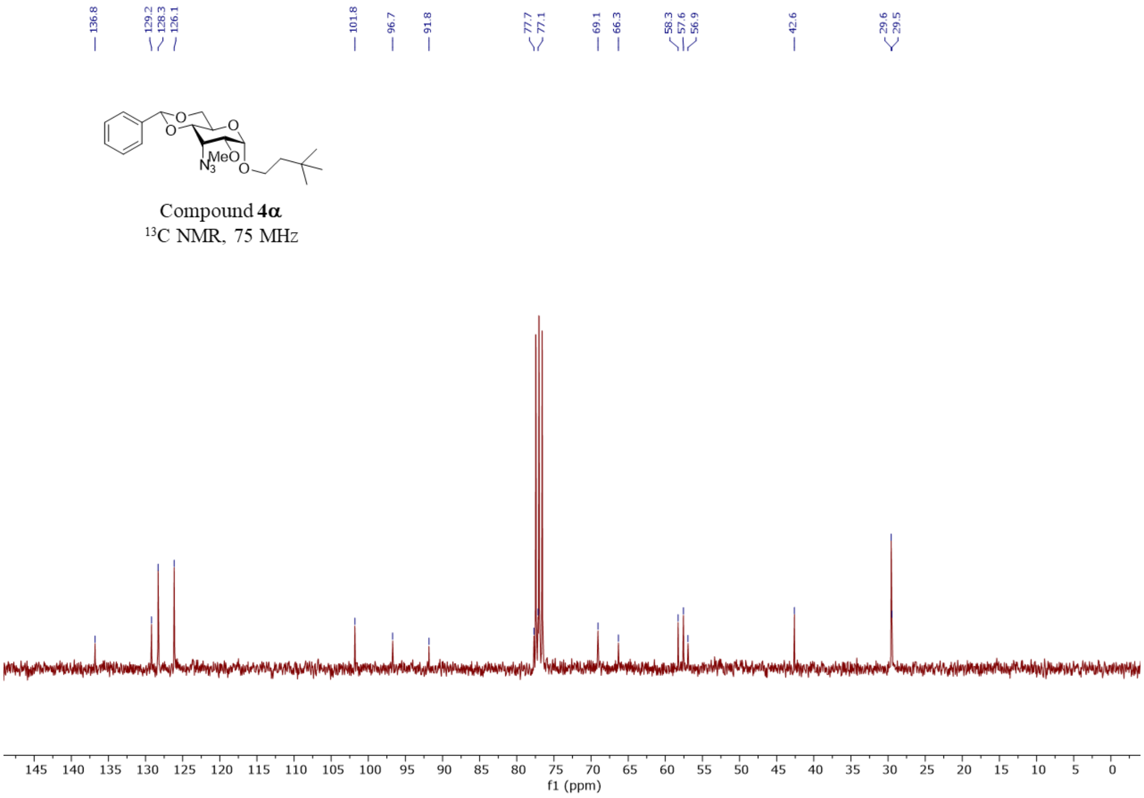

Compound **4α**
^{13}C NMR, 75 MHz

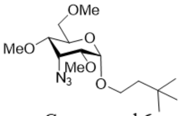

Compound **6**
^{1}H NMR, 400 MHz

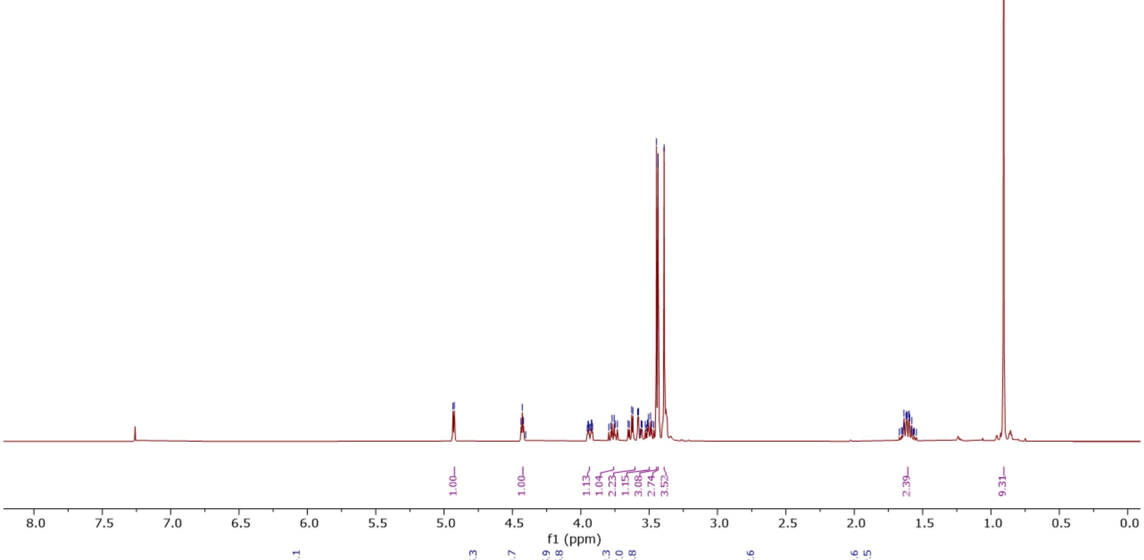

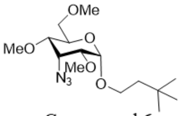

Compound **6**
^{13}C NMR, 101 MHz

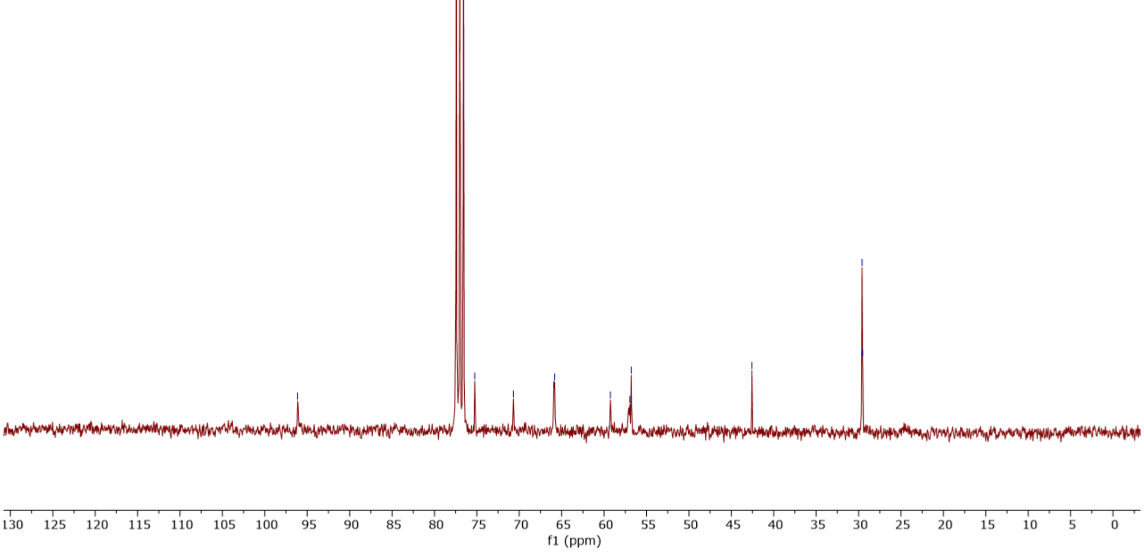

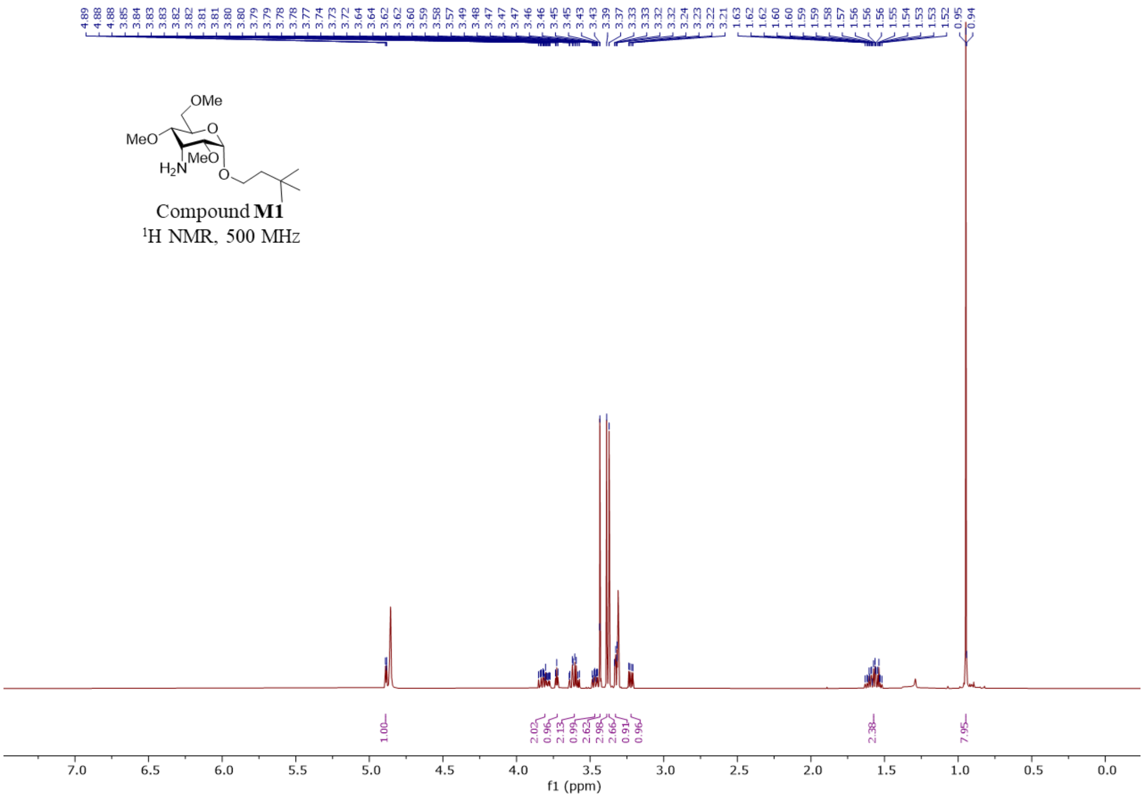

Compound **M1**
^{1}H NMR, 500 MHz

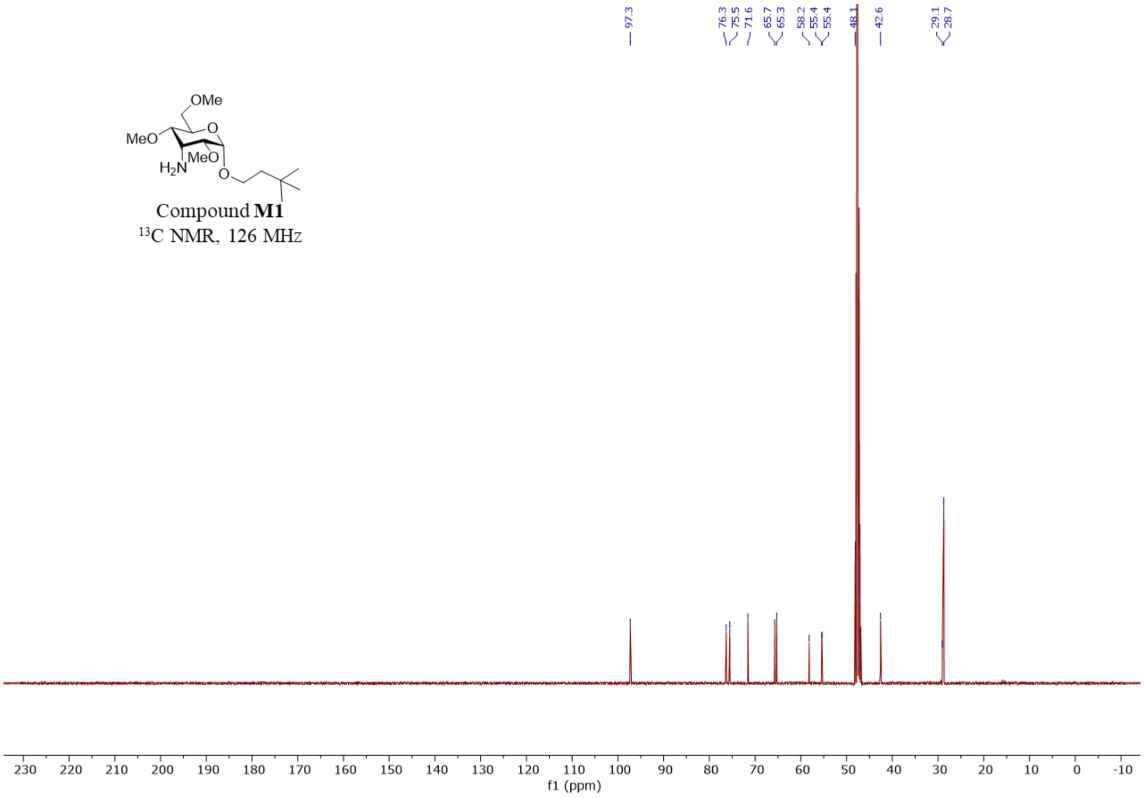

Compound **M1**
^{13}C NMR, 126 MHz

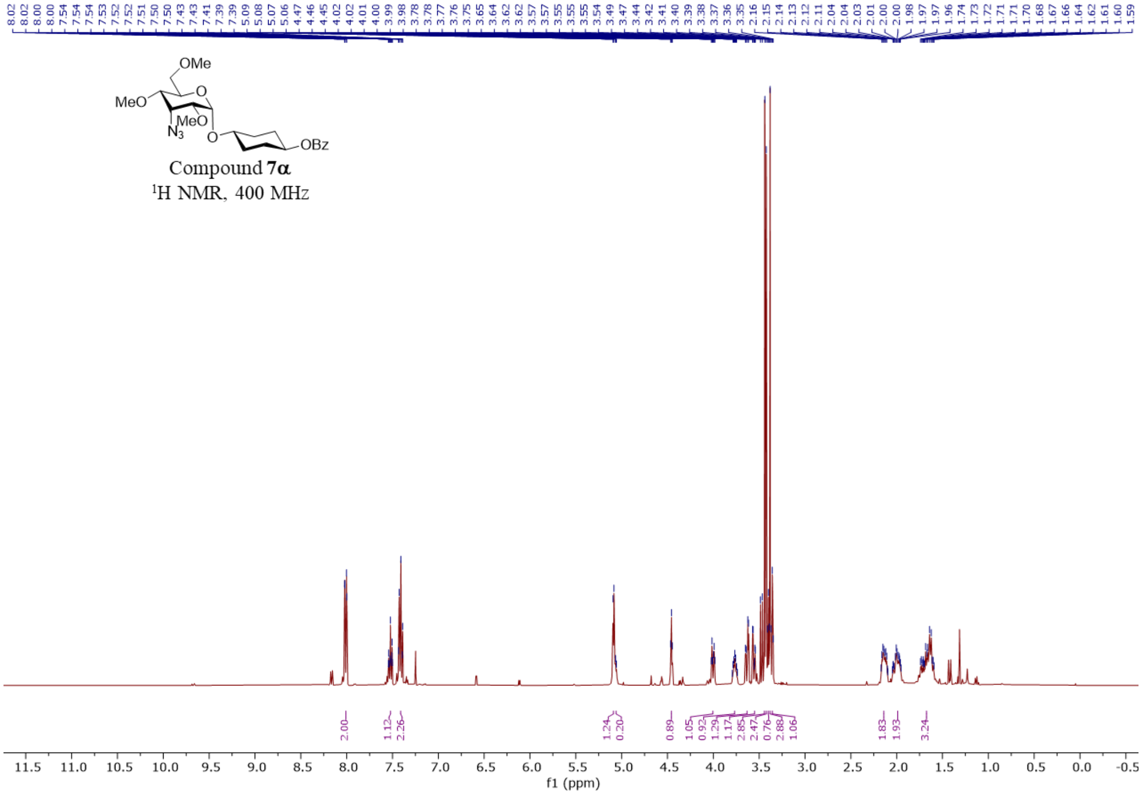

Compound **7α**
^{1}H NMR, 400 MHz

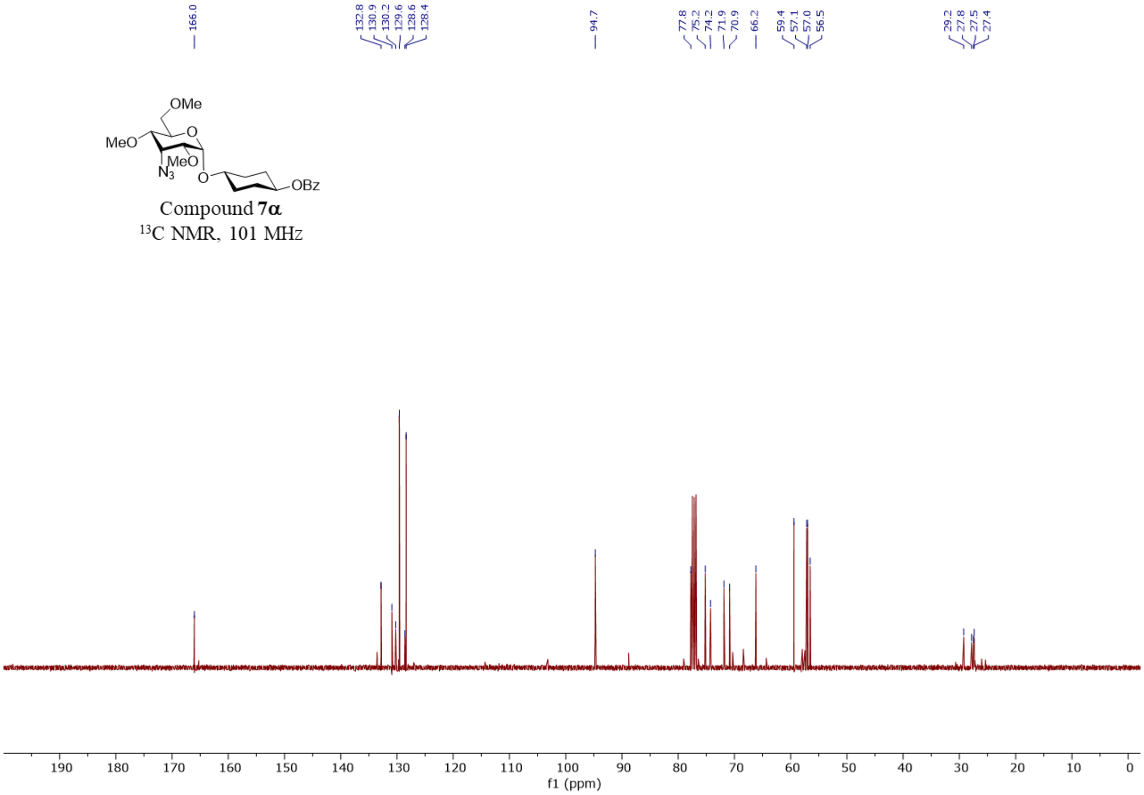

Compound **7α**
^{13}C NMR, 101 MHz

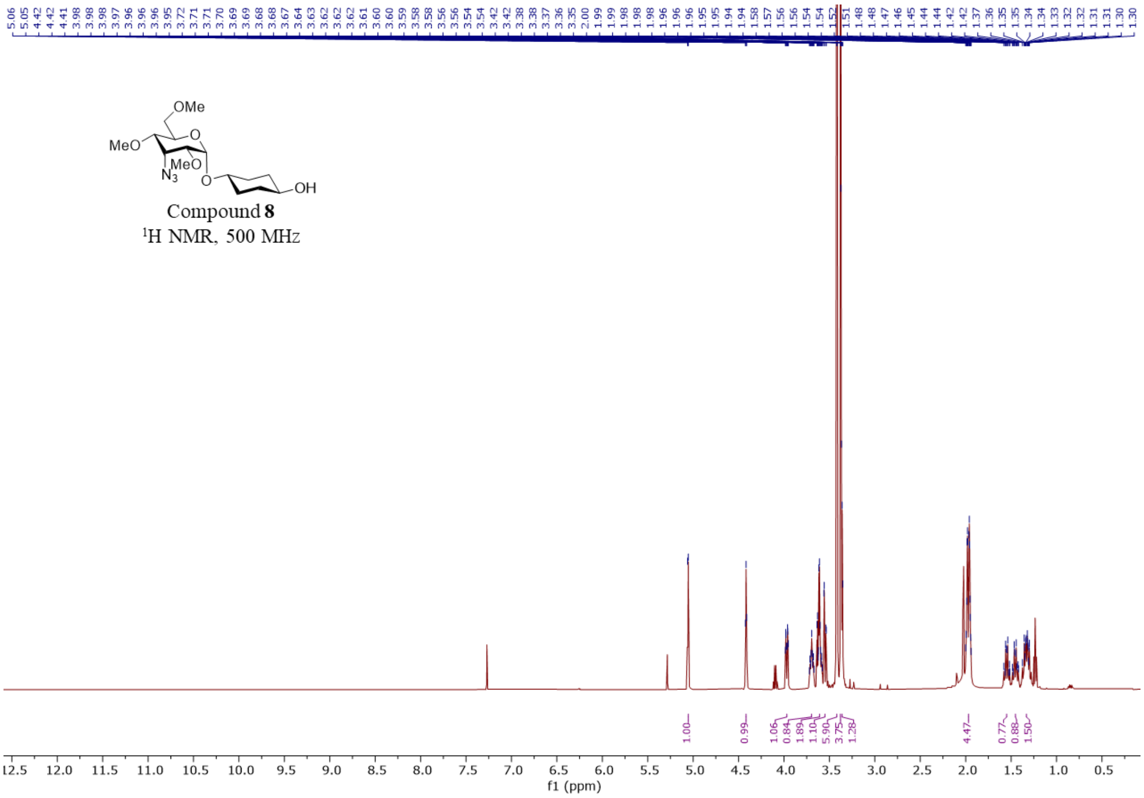

Compound **8**
^{1}H NMR, 500 MHz

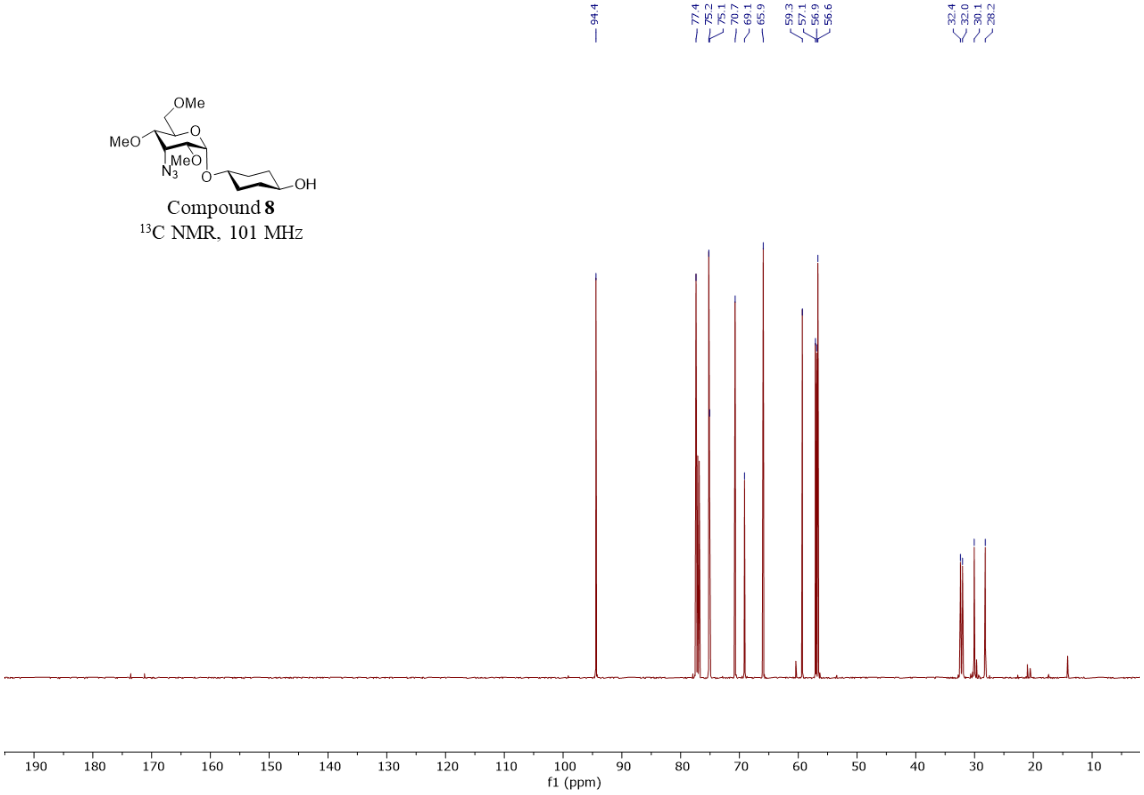

Compound **8**
^{13}C NMR, 101 MHz

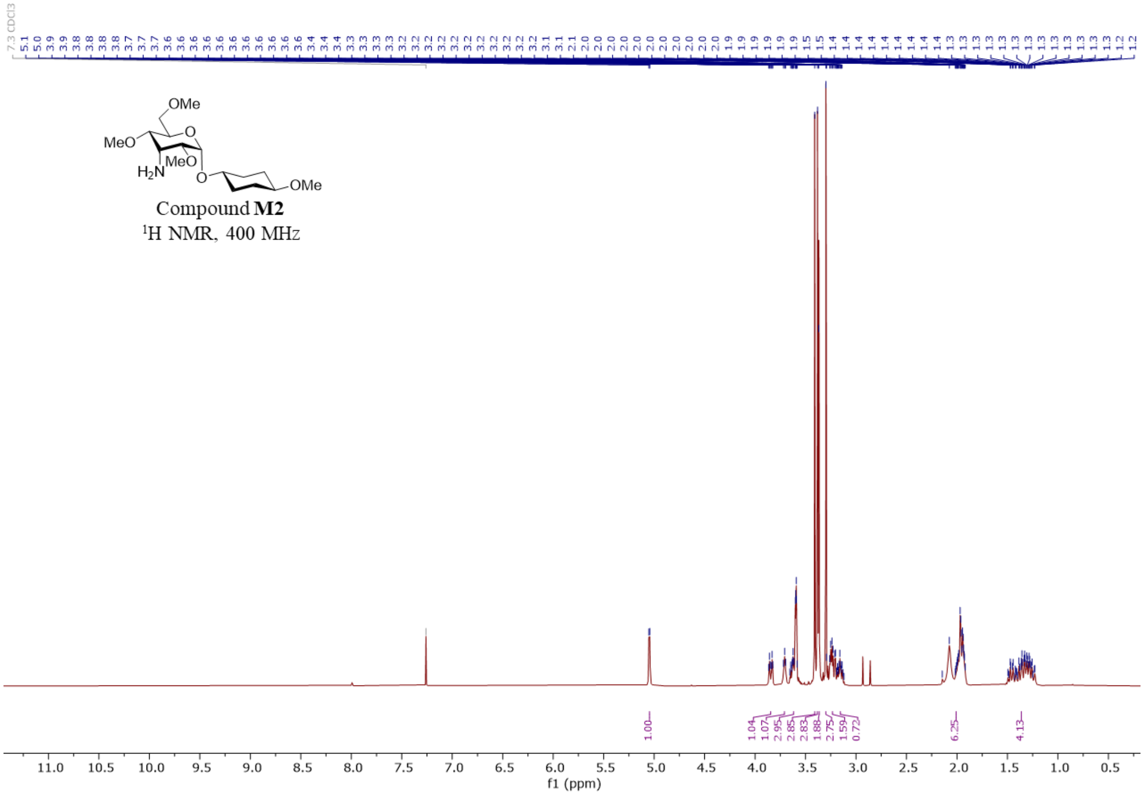
Compound **M2**
^{1}H NMR, 400 MHz
f1 (ppm)

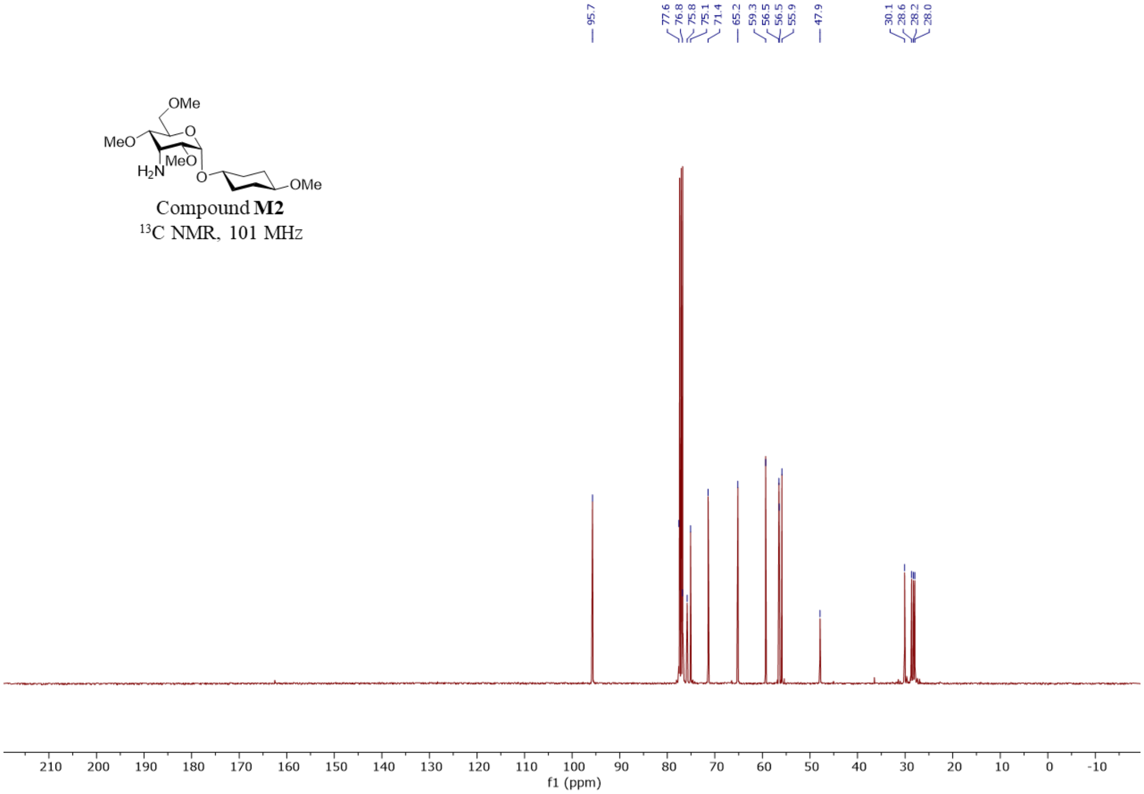
Compound **M2**
^{13}C NMR, 101 MHz
f1 (ppm)

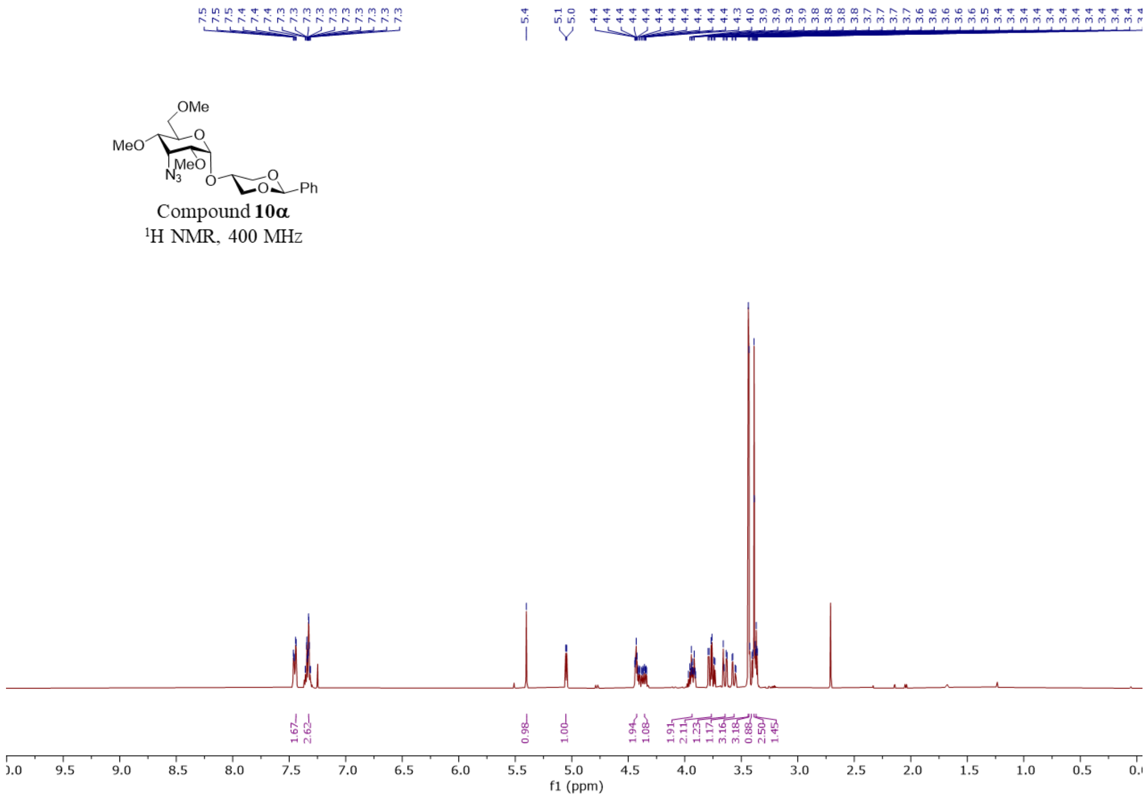

Compound **10α**
^{1}H NMR, 400 MHz

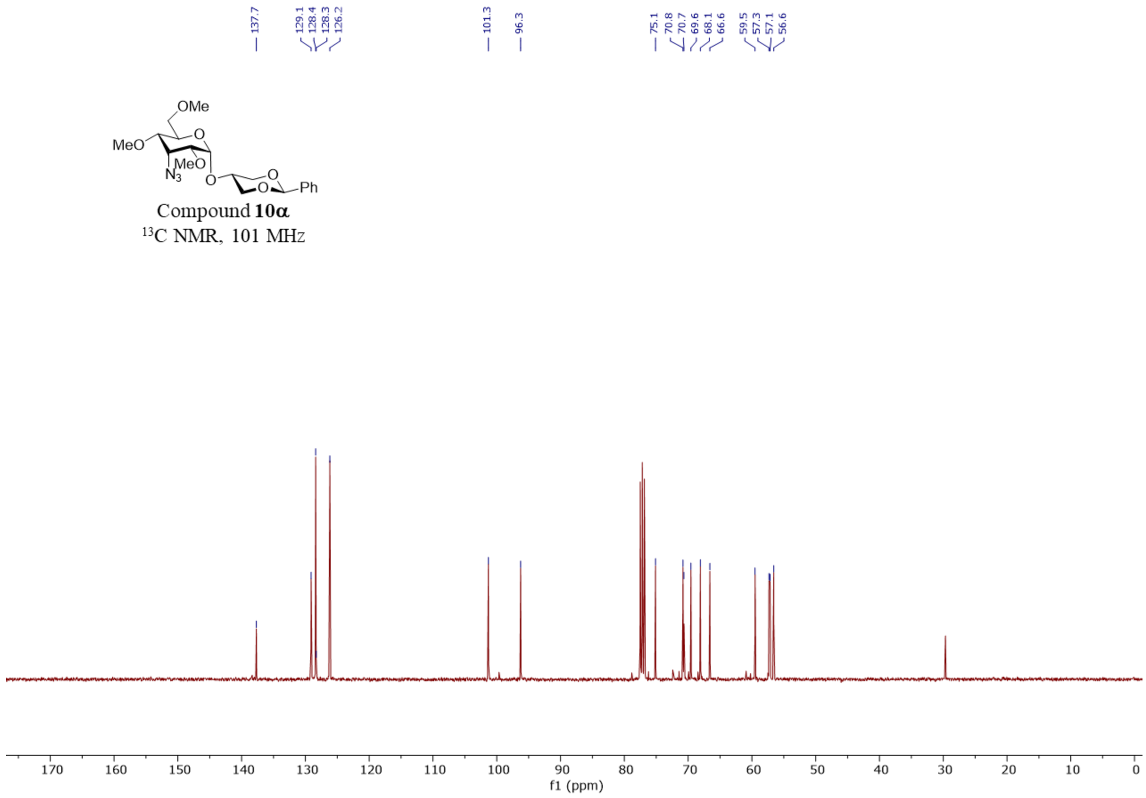

Compound **10α**
^{13}C NMR, 101 MHz

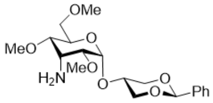

Compound **M3**
^{1}H NMR, 400 MHz

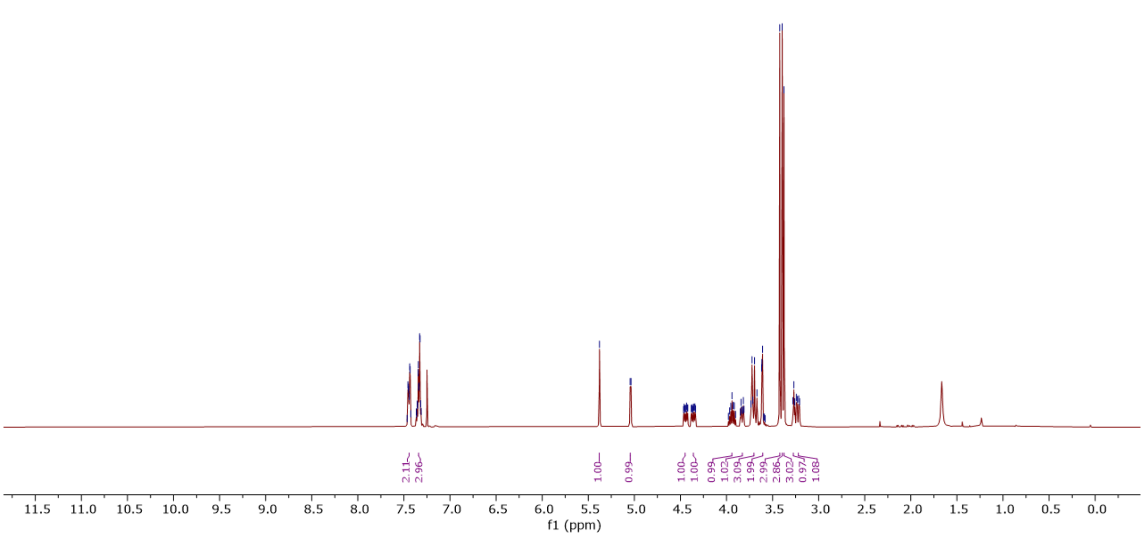

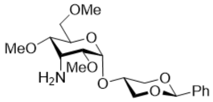

Compound **M3**
^{13}C NMR, 101 MHz

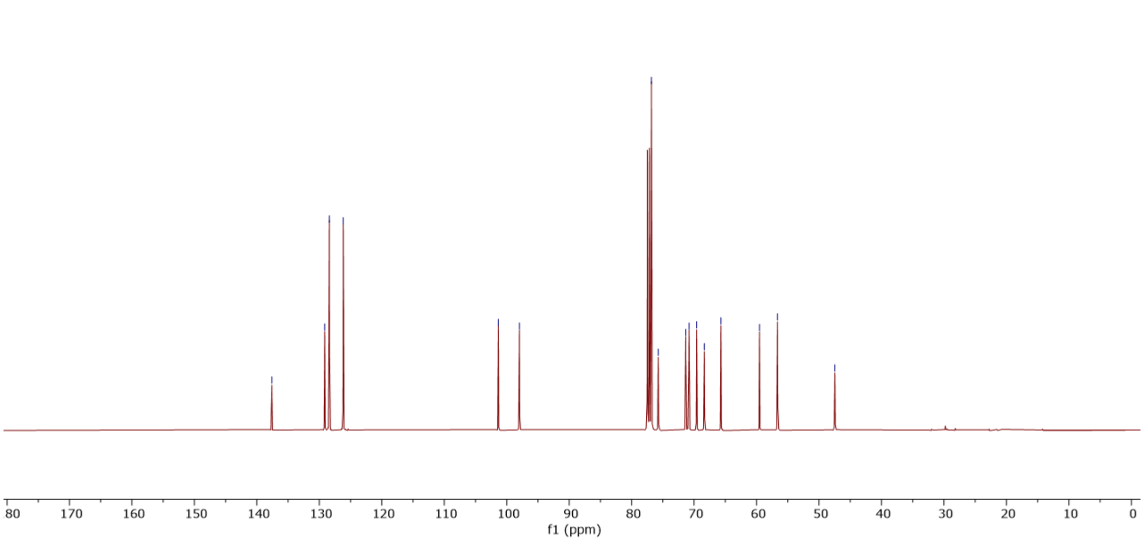

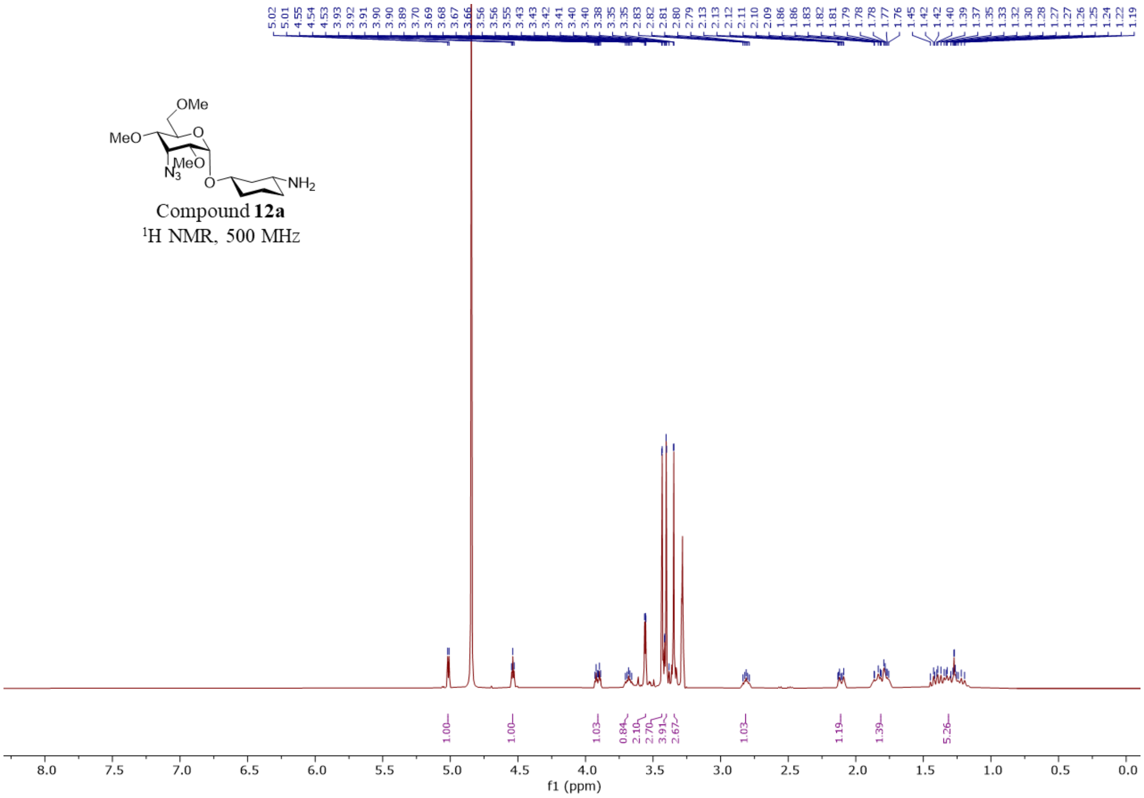
5.02
5.01
4.55
4.54
4.53
3.93
3.92
3.91
3.90
3.89
3.70
3.69
3.68
3.67
3.66
3.56
3.56
3.55
3.43
3.43
3.42
3.41
3.40
3.40
3.38
3.35
3.35
2.83
2.82
2.81
2.80
2.79
2.13
2.13
2.12
2.11
2.10
2.09
1.86
1.86
1.83
1.82
1.81
1.79
1.78
1.78
1.77
1.76
1.45
1.42
1.42
1.40
1.39
1.37
1.35
1.33
1.32
1.30
1.28
1.27
1.27
1.26
1.25
1.24
1.22
1.19
OMe
MeO
O
MeO
N_3
O
NH_2
Compound **12a**
^{1}H NMR, 500 MHz
1.00
1.00
1.03
0.84
2.10
2.70
3.91
2.67
1.03
1.19
1.39
5.26
8.0 7.5 7.0 6.5 6.0 5.5 5.0 4.5 4.0 3.5 3.0 2.5 2.0 1.5 1.0 0.5 0.0
f1 (ppm)

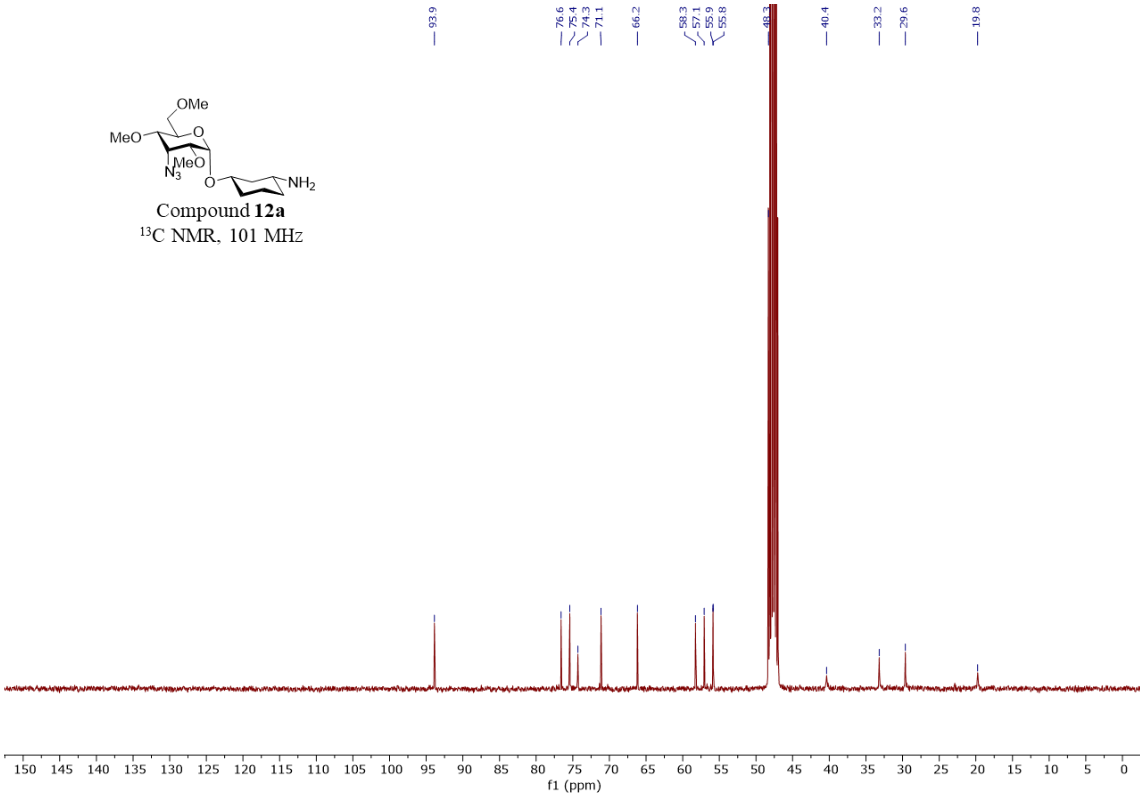
93.9
76.6
75.4
74.3
71.1
66.2
58.3
57.1
55.9
55.8
48.3
40.4
33.2
29.6
19.8
OMe
MeO
O
MeO
N_3
O
NH_2
Compound **12a**
^{13}C NMR, 101 MHz
150 145 140 135 130 125 120 115 110 105 100 95 90 85 80 75 70 65 60 55 50 45 40 35 30 25 20 15 10 5 0
f1 (ppm)

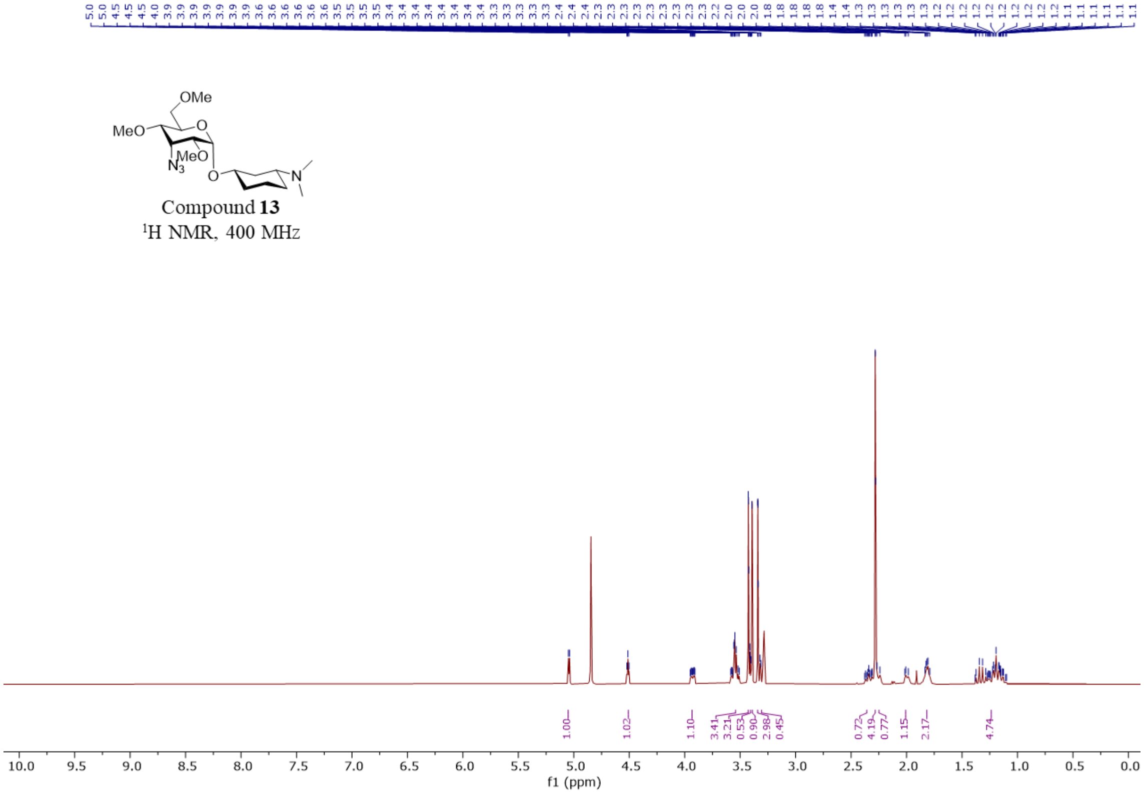
OMe
MeO
O
MeO
N_3
O
N
Compound **13**
^{1}H NMR, 400 MHz
f1 (ppm)

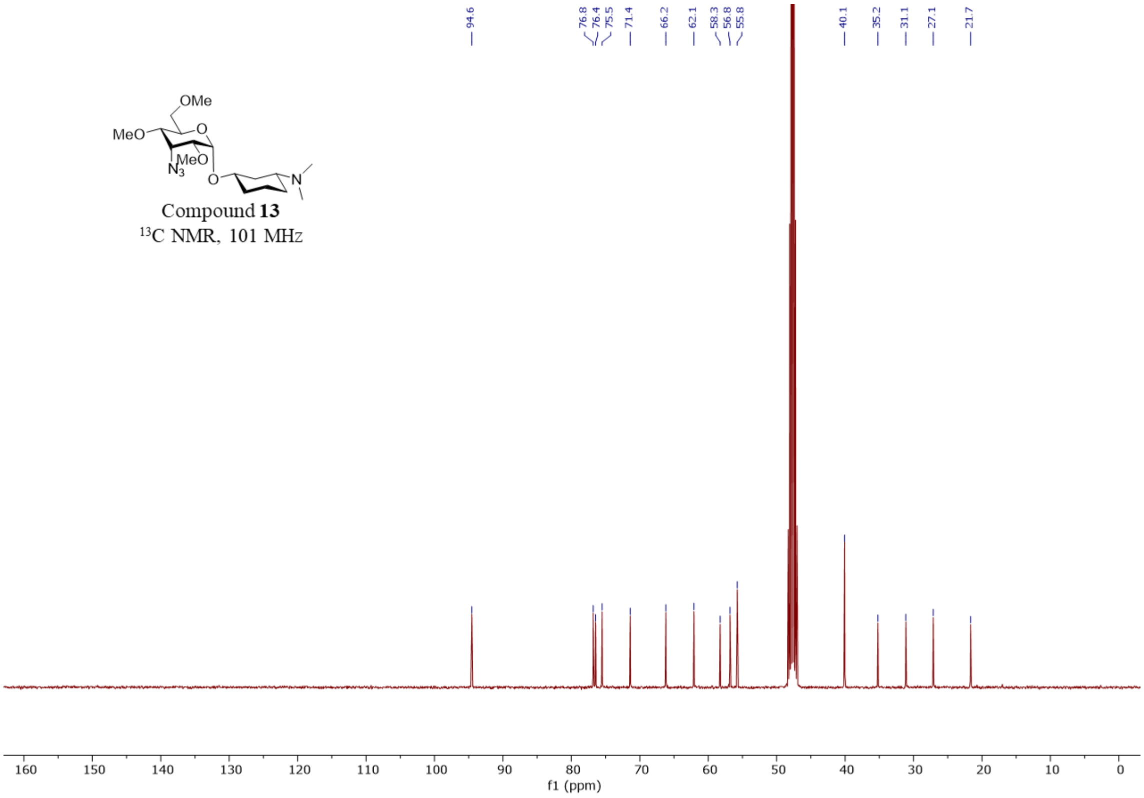
94.6
76.8
76.4
75.5
71.4
66.2
62.1
58.3
56.8
55.8
40.1
35.2
31.1
27.1
21.7
OMe
MeO
O
MeO
N_3
O
N
Compound **13**
^{13}C NMR, 101 MHz
f1 (ppm)

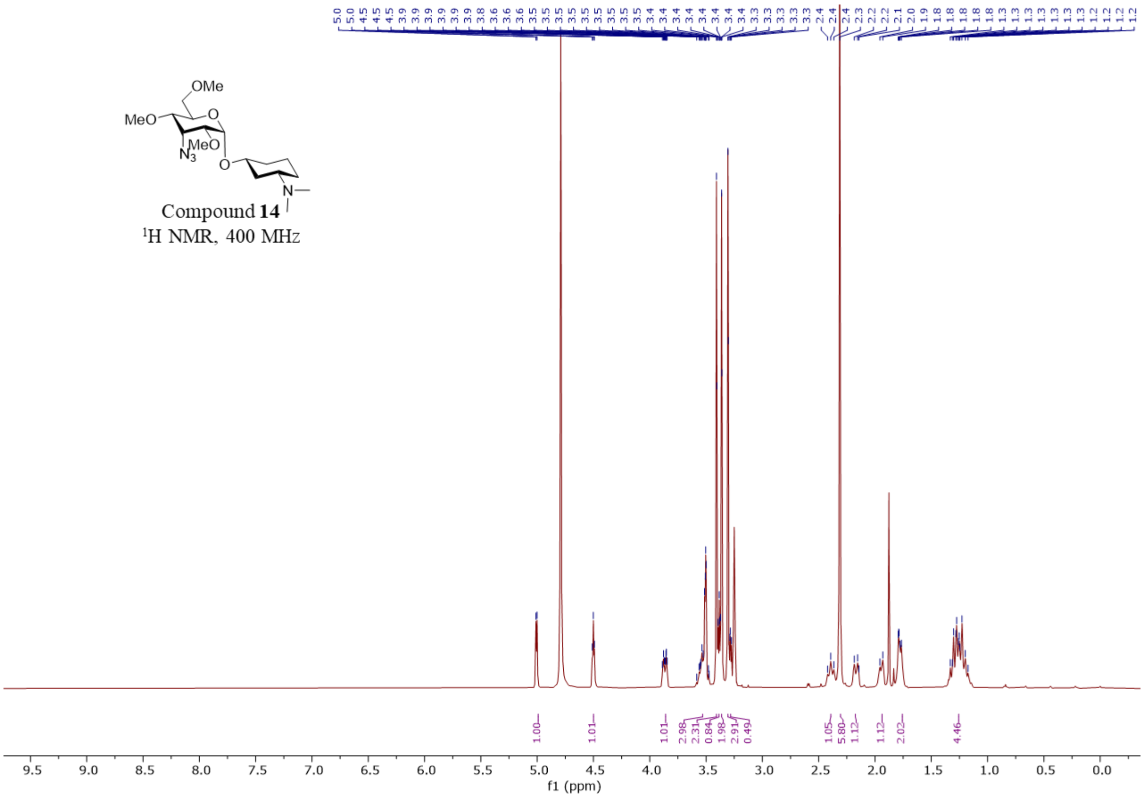
OMe
MeO
O
N3
MeO
O
N
Compound 14
1H NMR, 400 MHz
f1 (ppm)

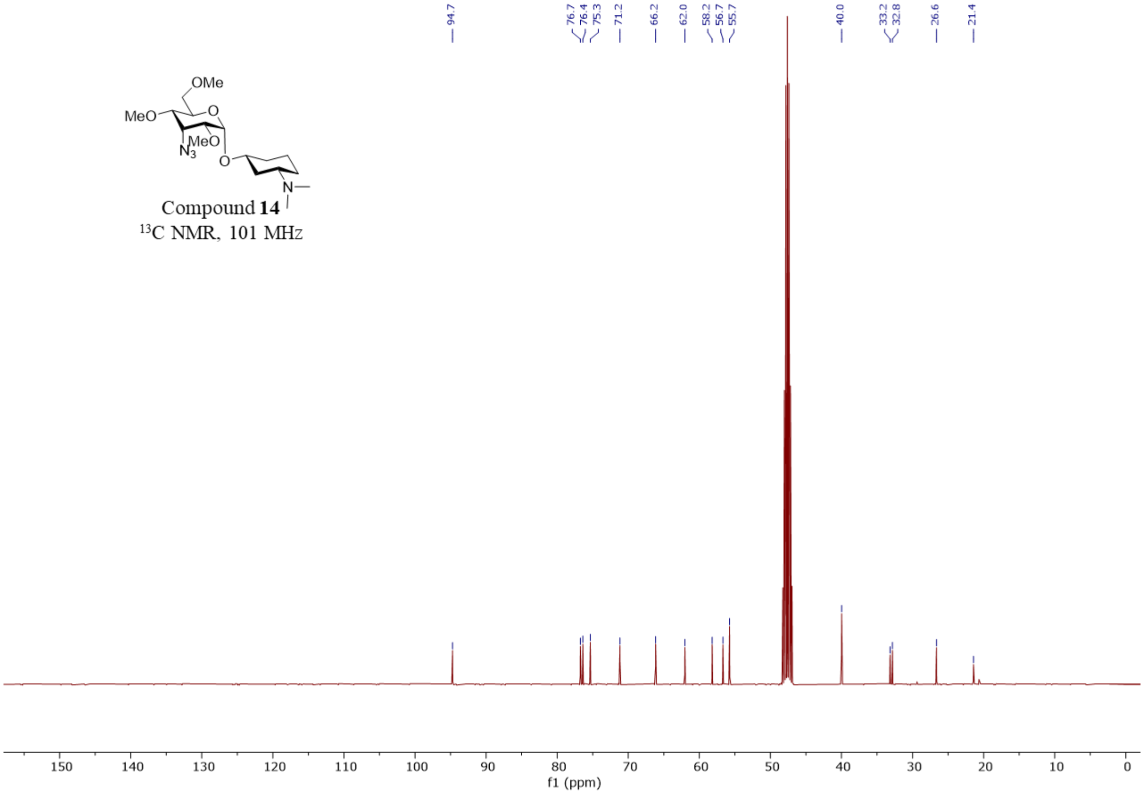
OMe
MeO
O
N3
MeO
O
N
Compound 14
13C NMR, 101 MHz
94.7
76.7
76.4
75.3
71.2
66.2
62.0
58.2
56.7
55.7
40.0
33.2
32.8
26.6
21.4
f1 (ppm)

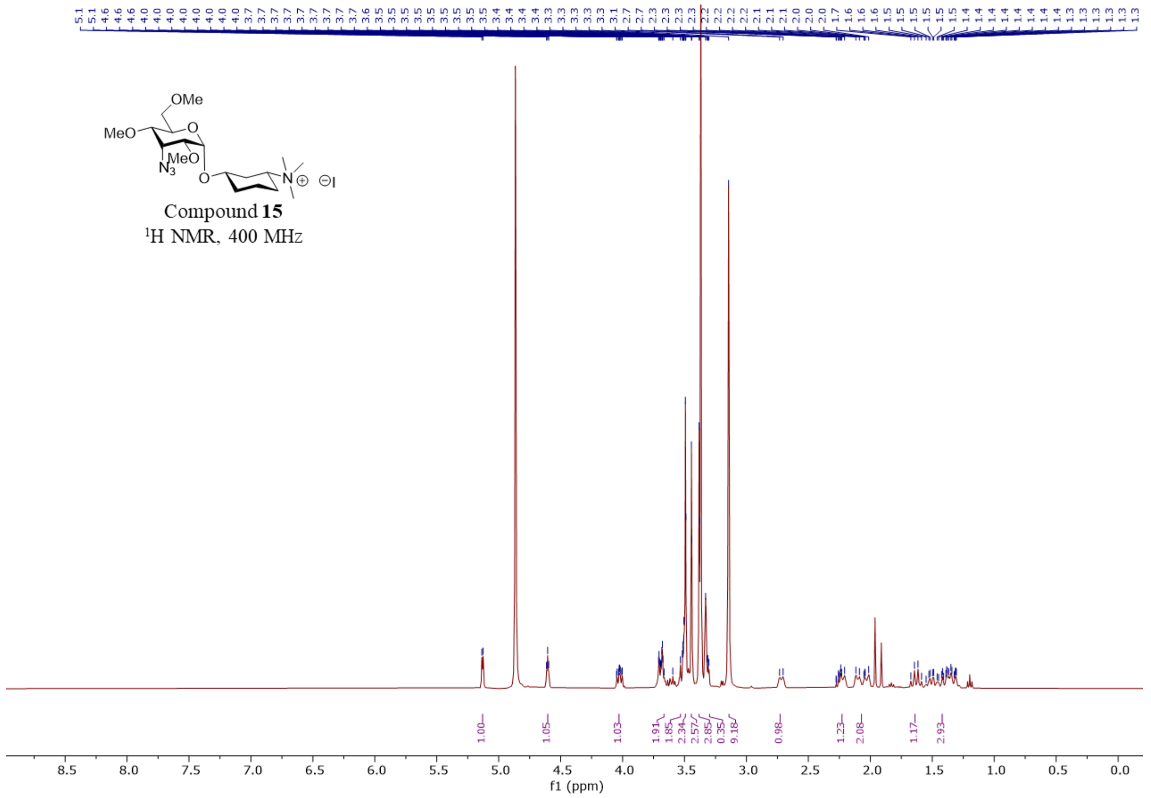
Compound 15
1H NMR, 400 MHz
f1 (ppm)

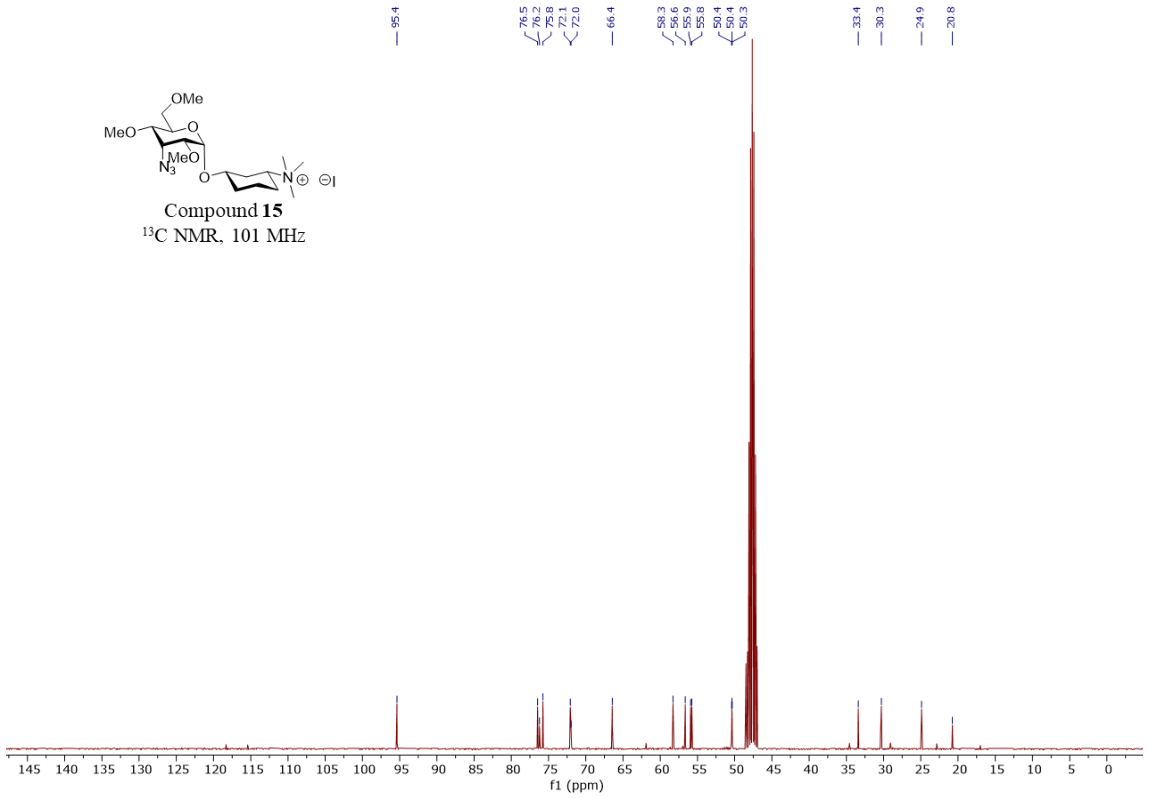
Compound 15
13C NMR, 101 MHz
95.4
76.5
76.2
75.8
72.1
72.0
66.4
58.3
56.6
55.9
55.8
50.4
50.4
50.3
33.4
30.3
24.9
20.8
f1 (ppm)

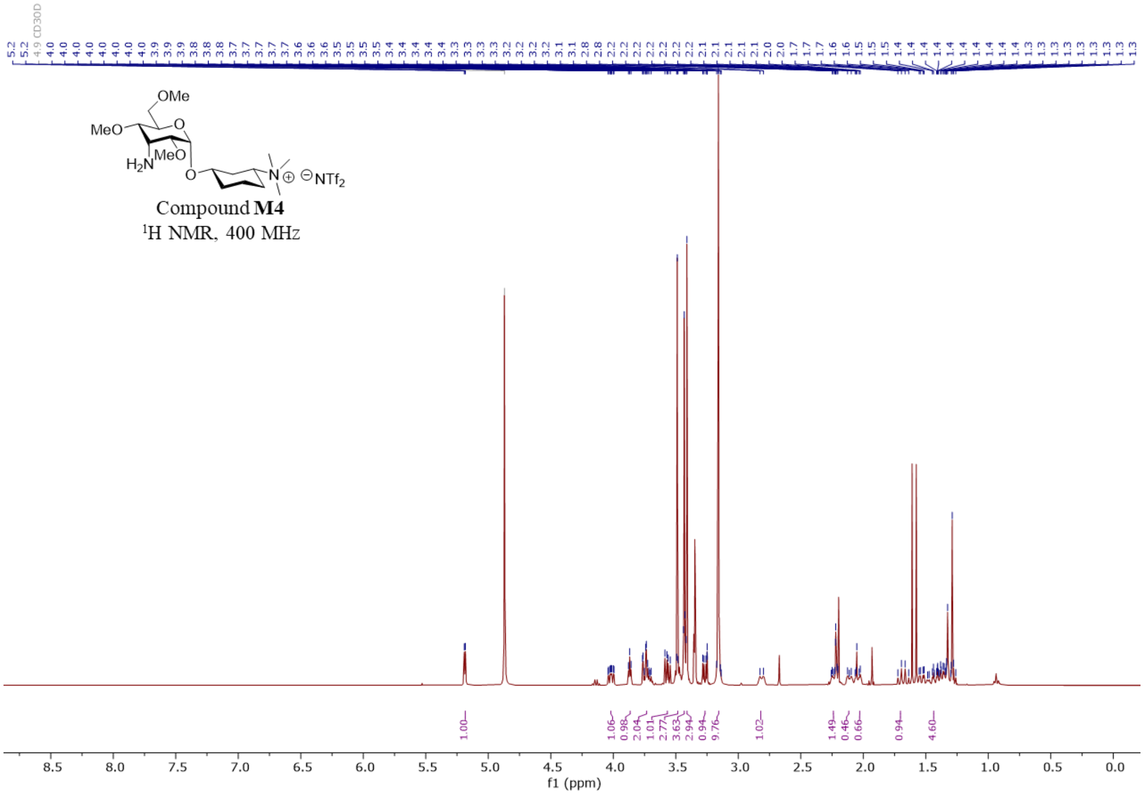
OMe
MeO
O
H2N
MeO
O
N⊕ ⊖NTf2
Compound **M4**
1H NMR, 400 MHz
f1 (ppm)

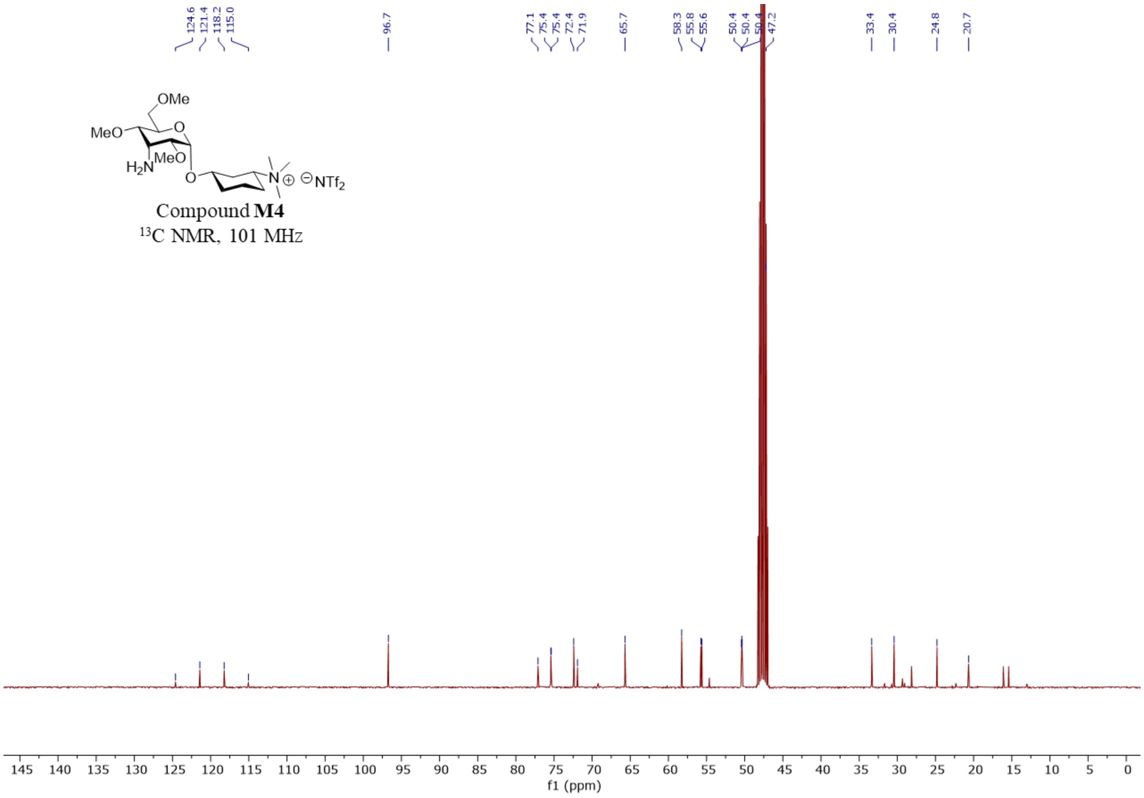
124.6
121.4
118.2
115.0
96.7
77.1
75.4
75.4
72.4
71.9
65.7
58.3
55.8
55.6
50.4
50.4
47.2
33.4
30.4
24.8
20.7
OMe
MeO
O
H2N
MeO
O
N⊕ ⊖NTf2
Compound **M4**
13C NMR, 101 MHz
f1 (ppm)

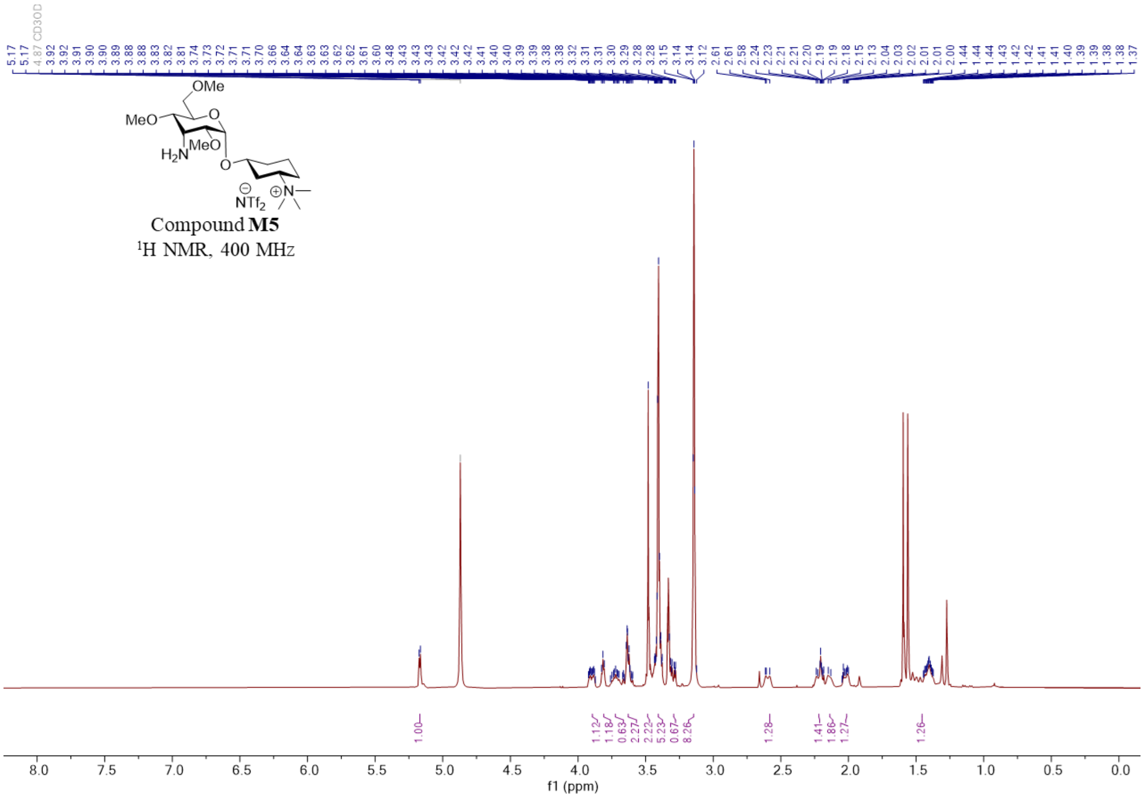
Compound M5
1H NMR, 400 MHz
f1 (ppm)

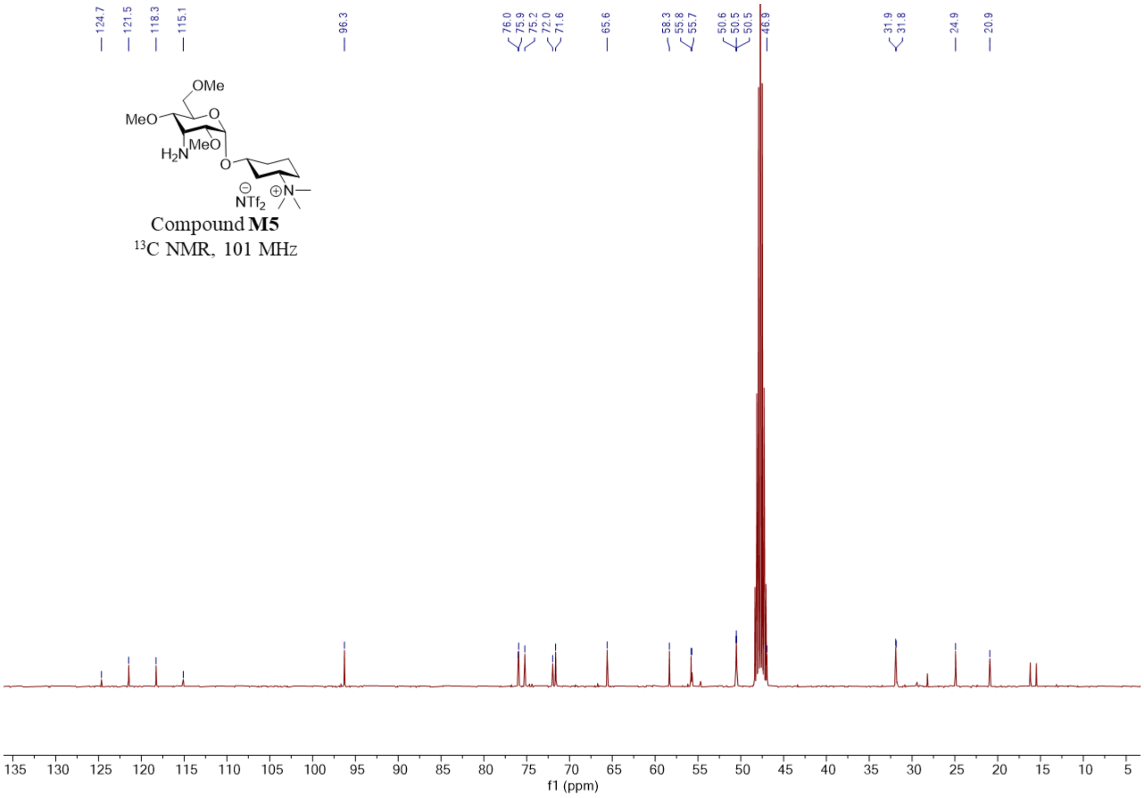
Compound M5
13C NMR, 101 MHz
f1 (ppm)

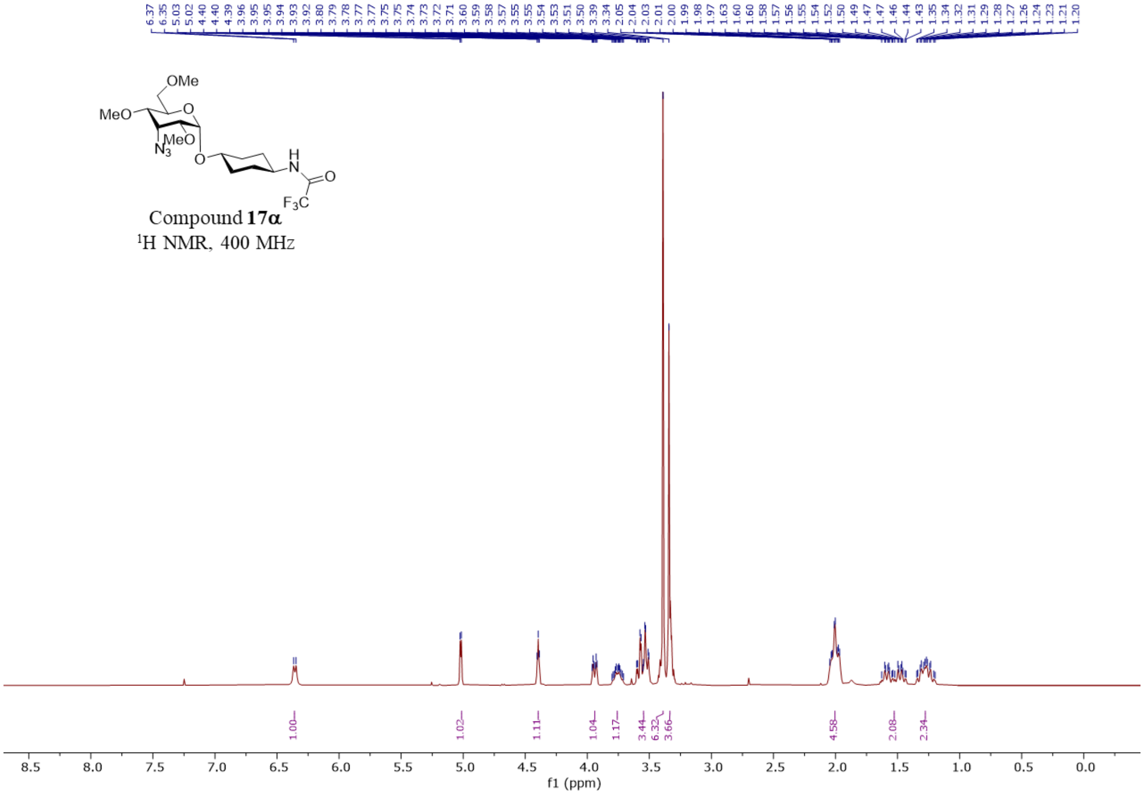

Compound **17α**
^{1}H NMR, 400 MHz

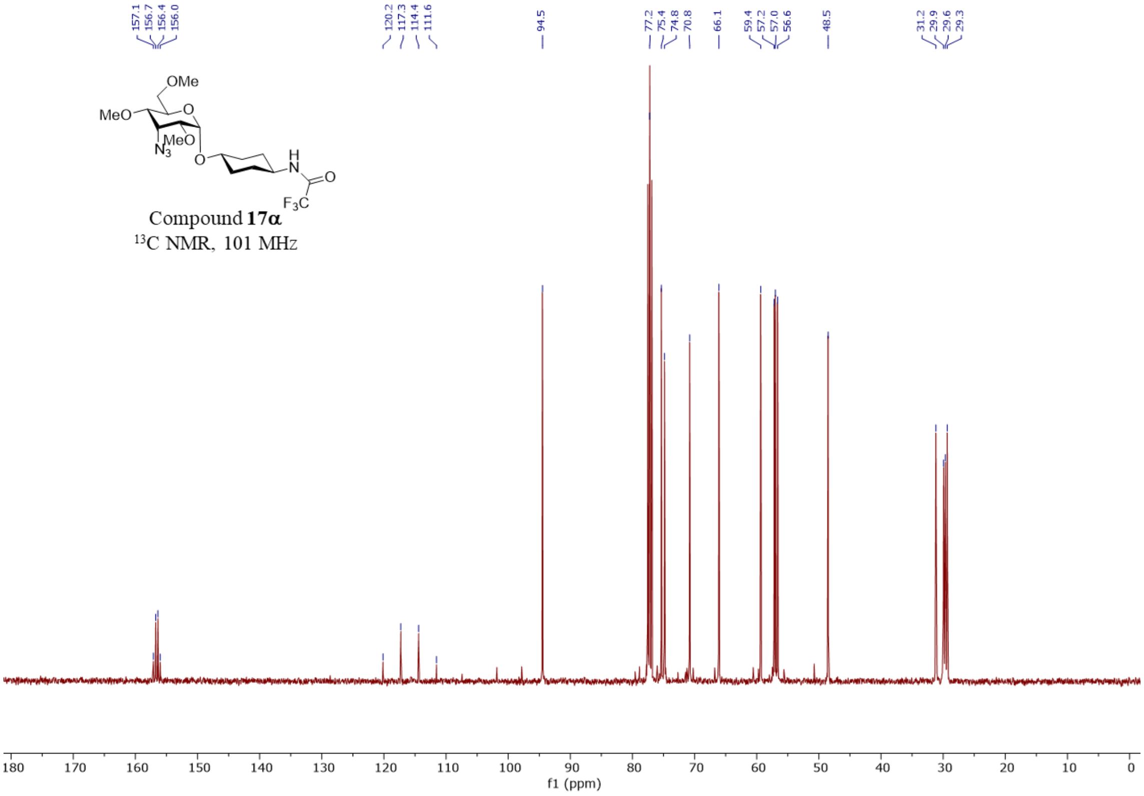

Compound **17α**
^{13}C NMR, 101 MHz

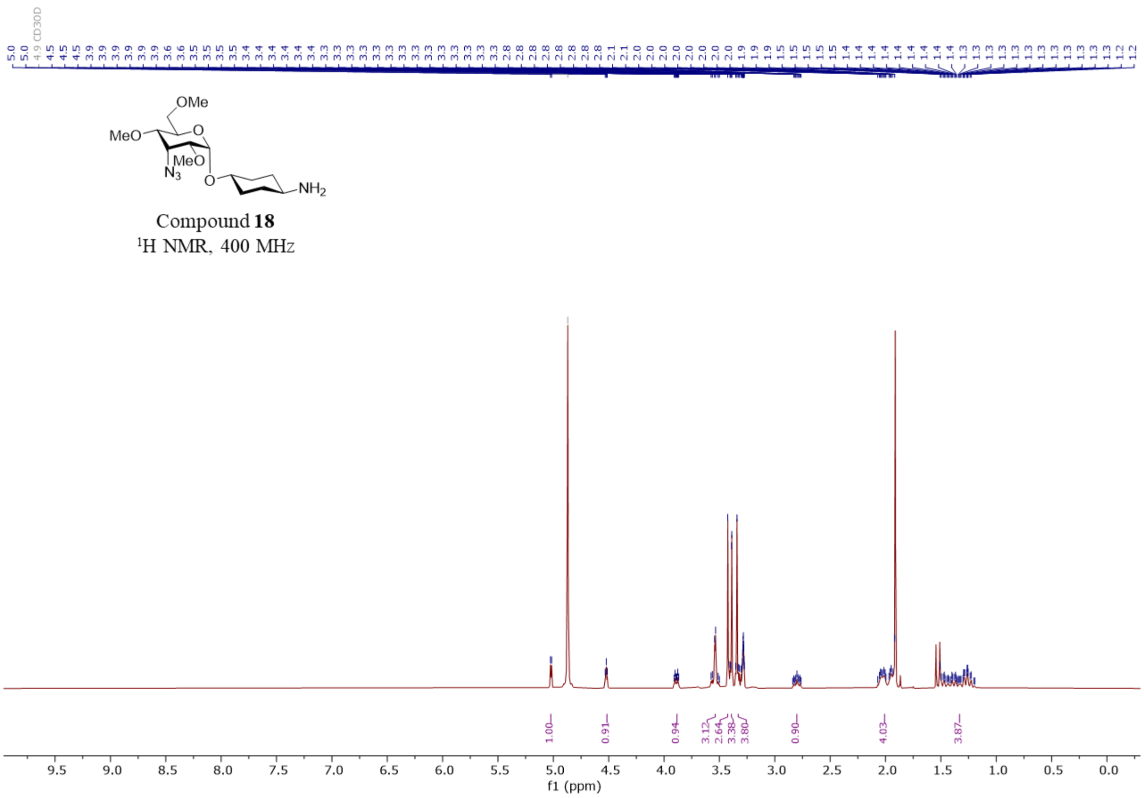

Compound **18**
^{1}H NMR, 400 MHz

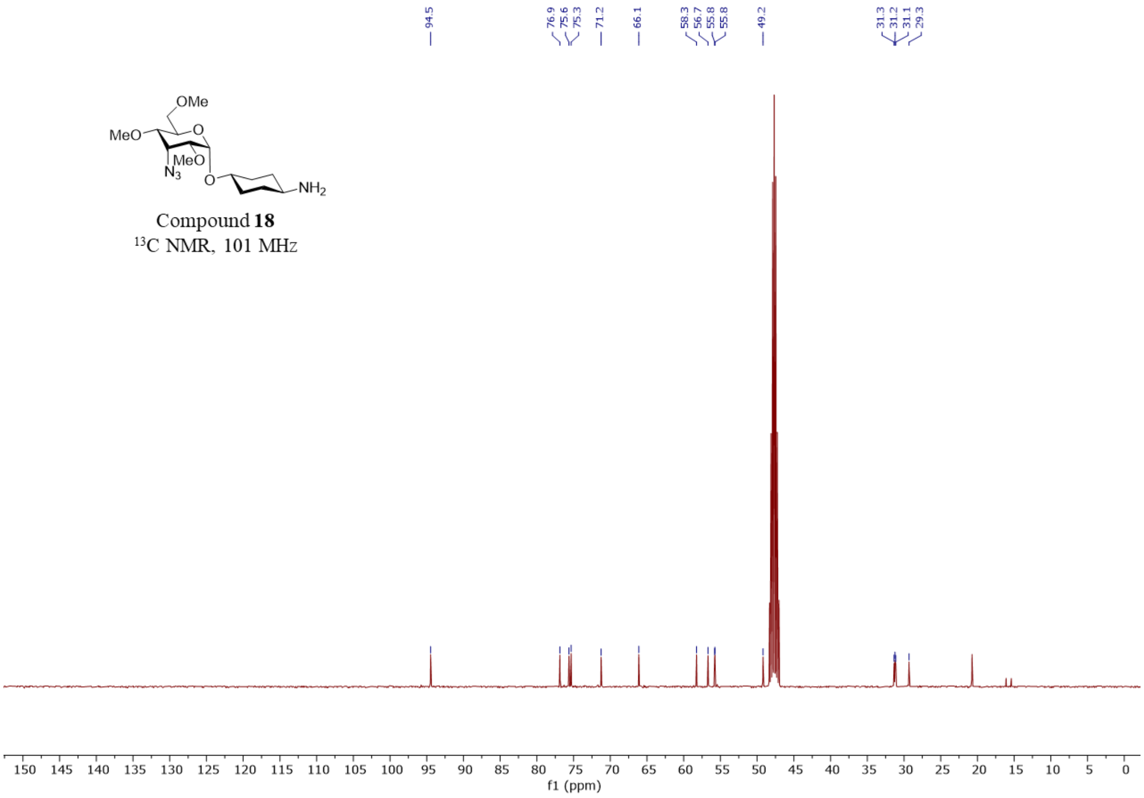

Compound **18**
^{13}C NMR, 101 MHz

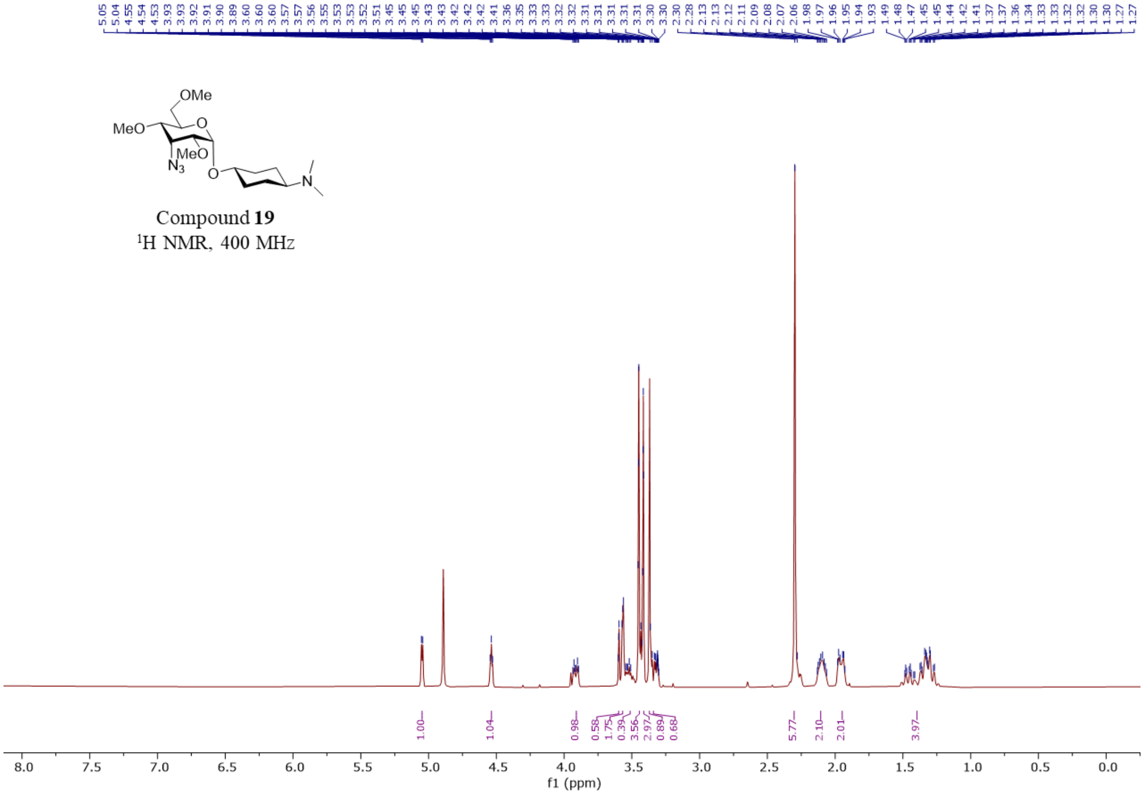
OMe
MeO
O
MeO
N3
O
N
Compound 19
1H NMR, 400 MHz
f1 (ppm)

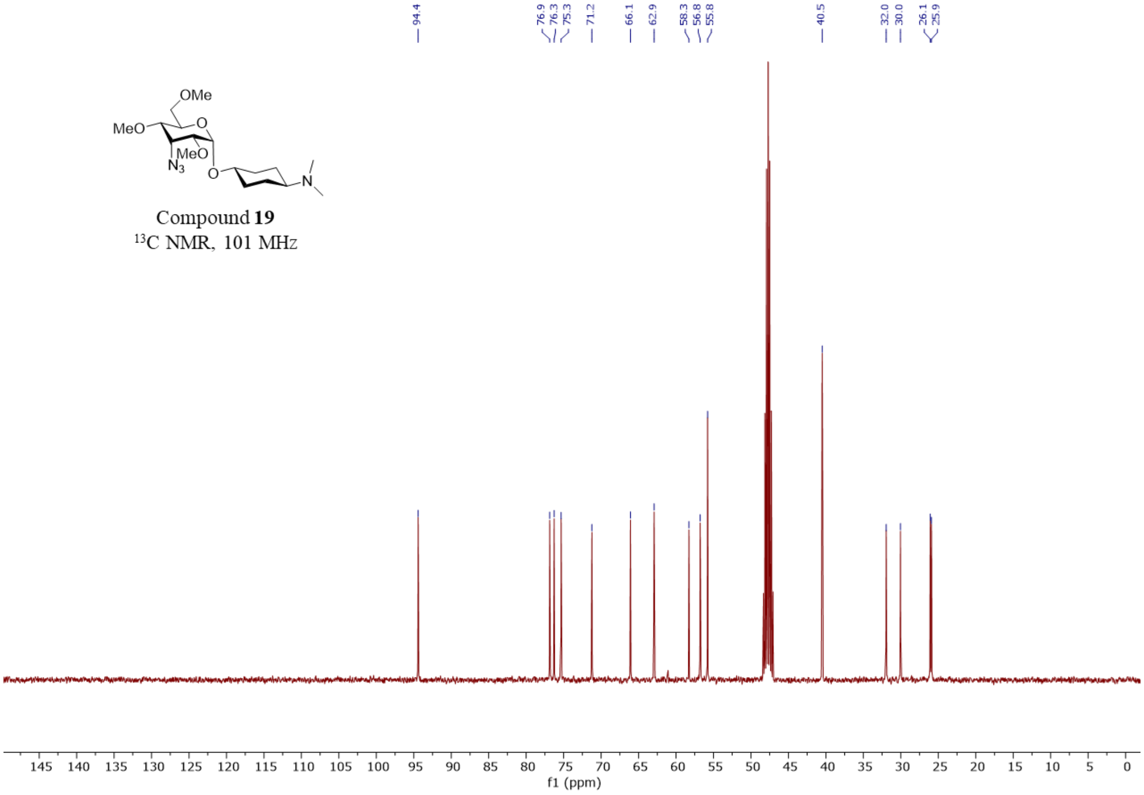
OMe
MeO
O
MeO
N3
O
N
Compound 19
13C NMR, 101 MHz
94.4
76.9
76.3
75.3
71.2
66.1
62.9
58.3
56.8
55.8
40.5
32.0
30.0
26.1
25.9
f1 (ppm)

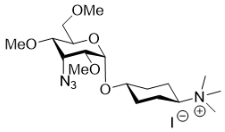

Compound **20**
^{1}H NMR, 400 MHz

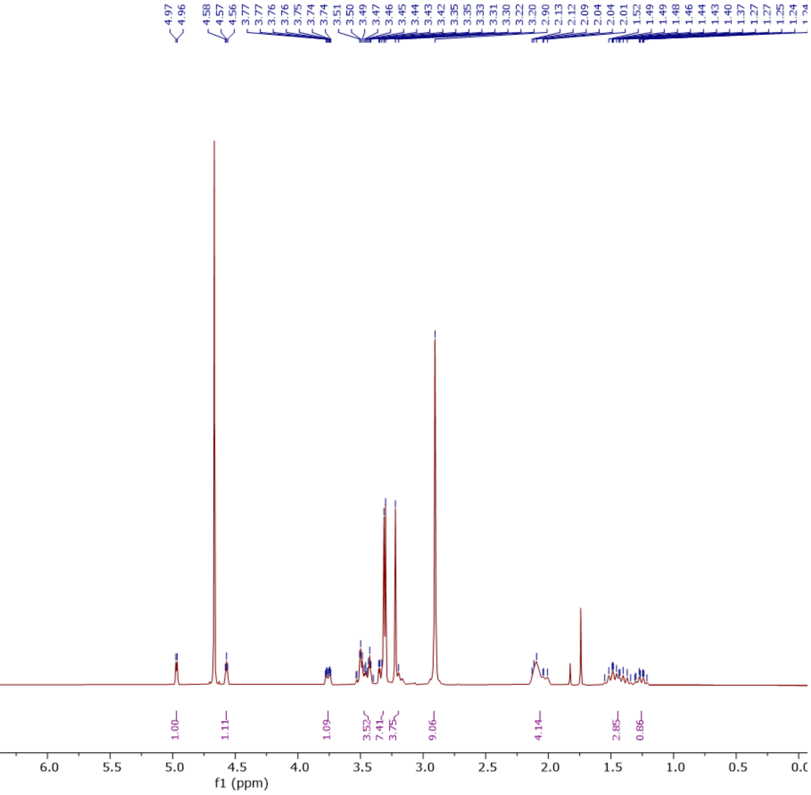

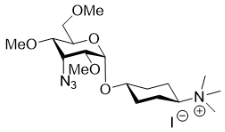

Compound **20**
^{13}C NMR, 101 MHz

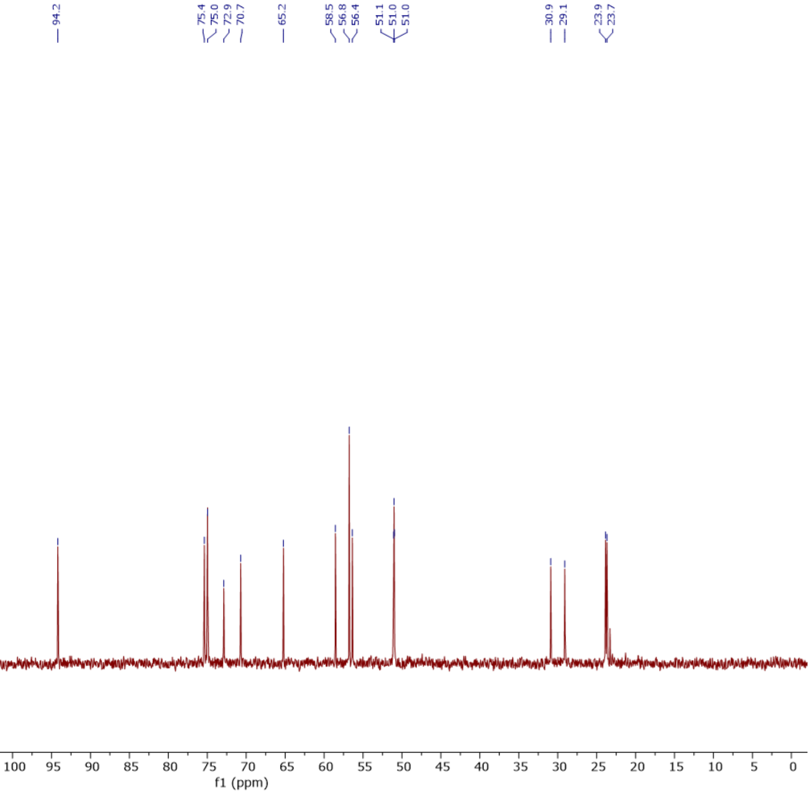

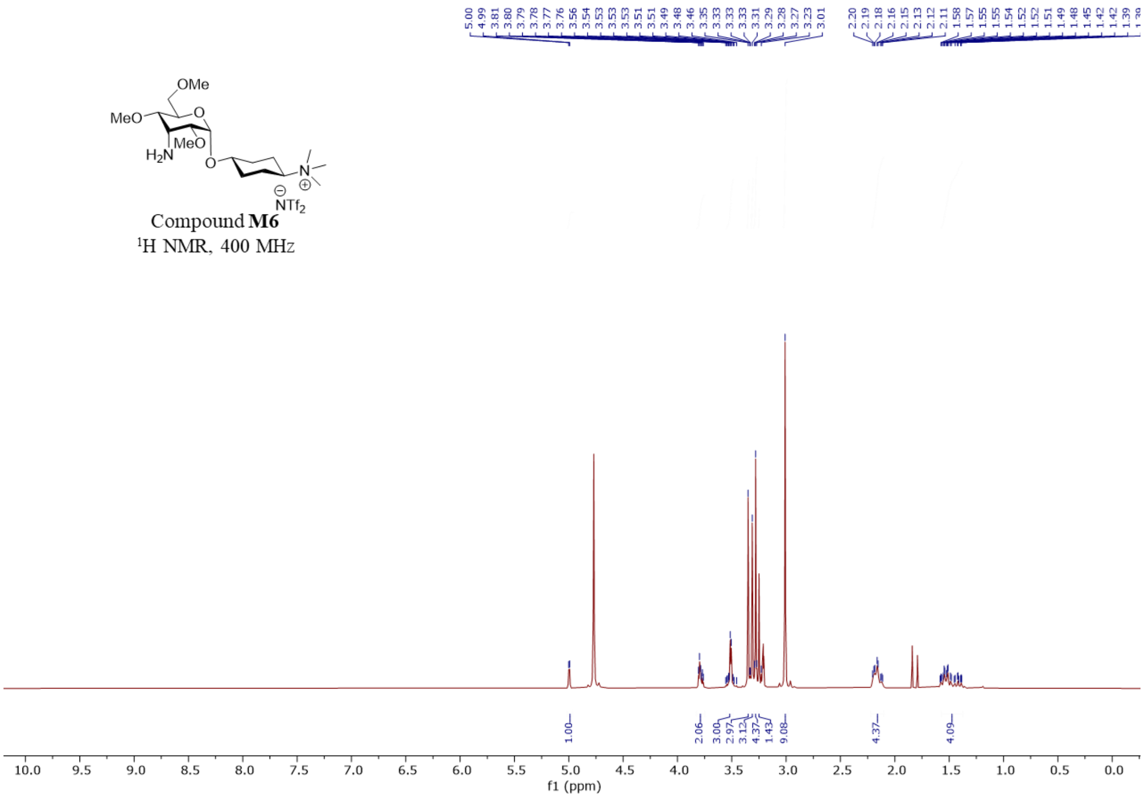

Compound **M6**
^{1}H NMR, 400 MHz

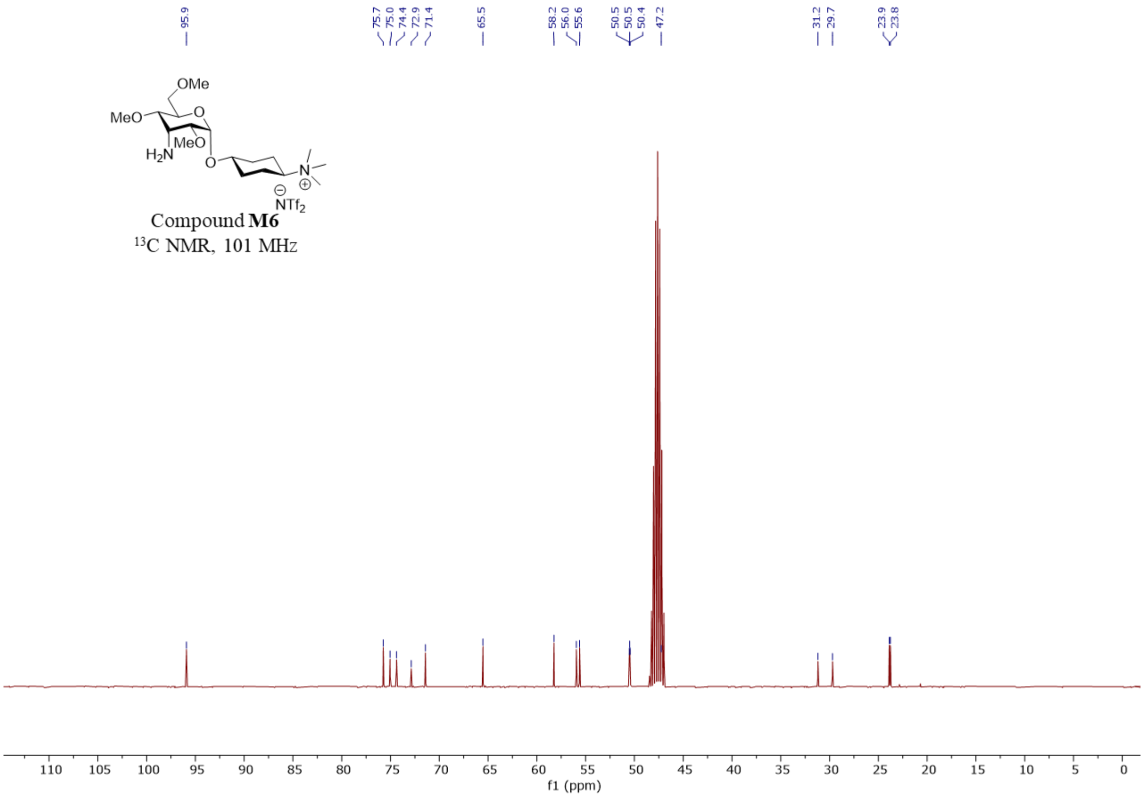

Compound **M6**
^{13}C NMR, 101 MHz

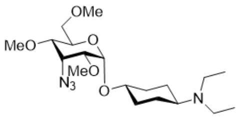

Compound **21**
^{1}H NMR, 400 MHz

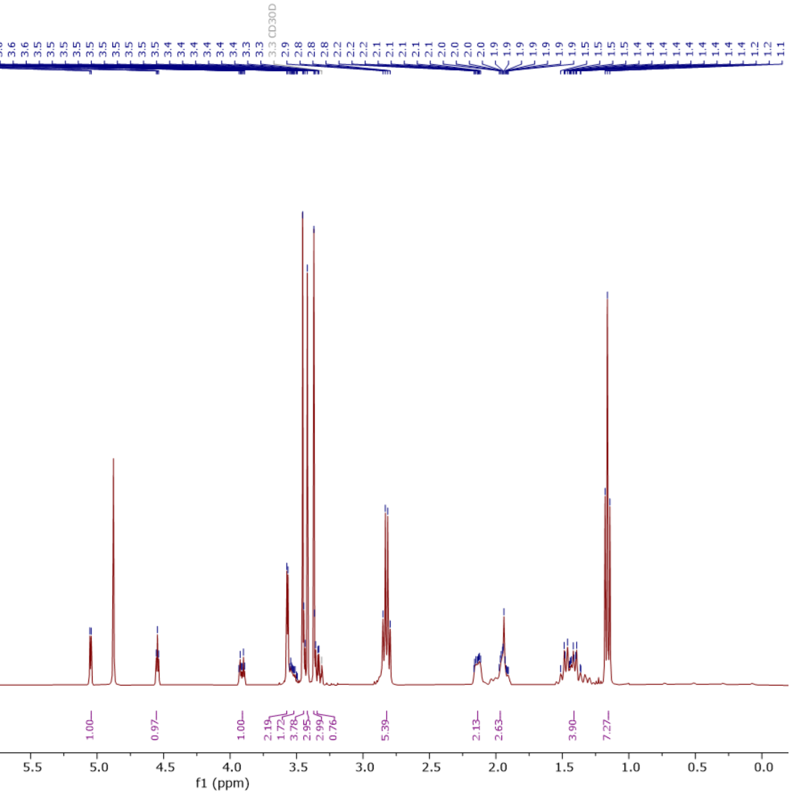

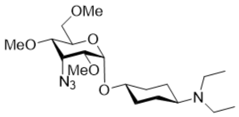

Compound **21**
^{13}C NMR, 101 MHz

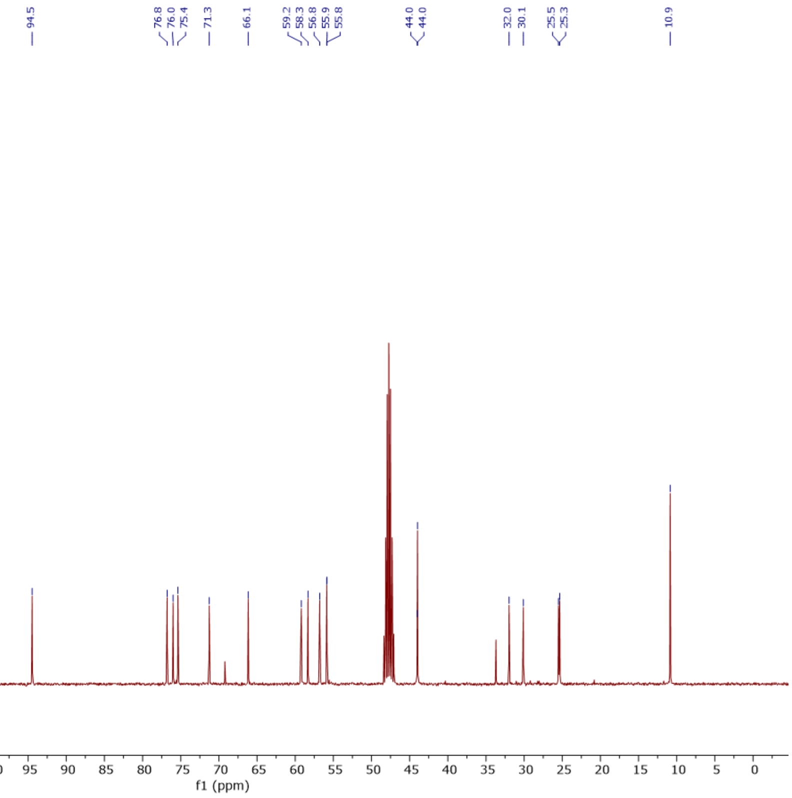

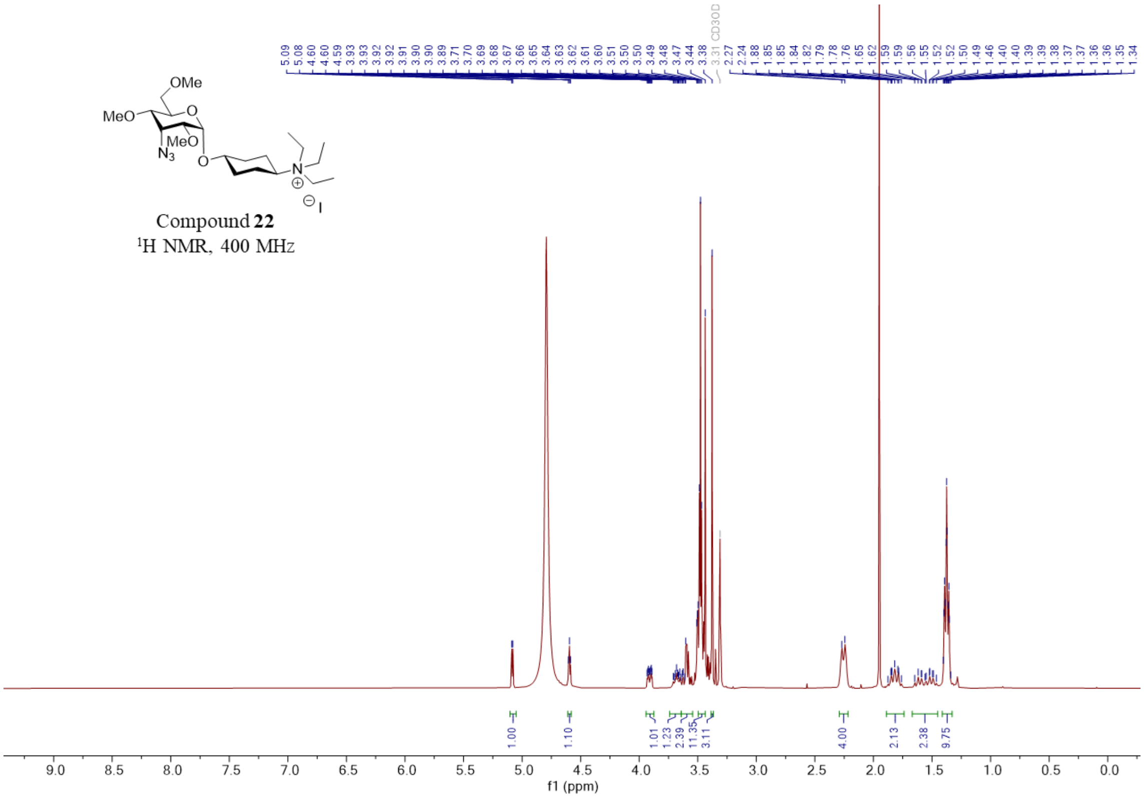
OMe
MeO
O
MeO
N3
O
N
I
Compound 22
1H NMR, 400 MHz
f1 (ppm)

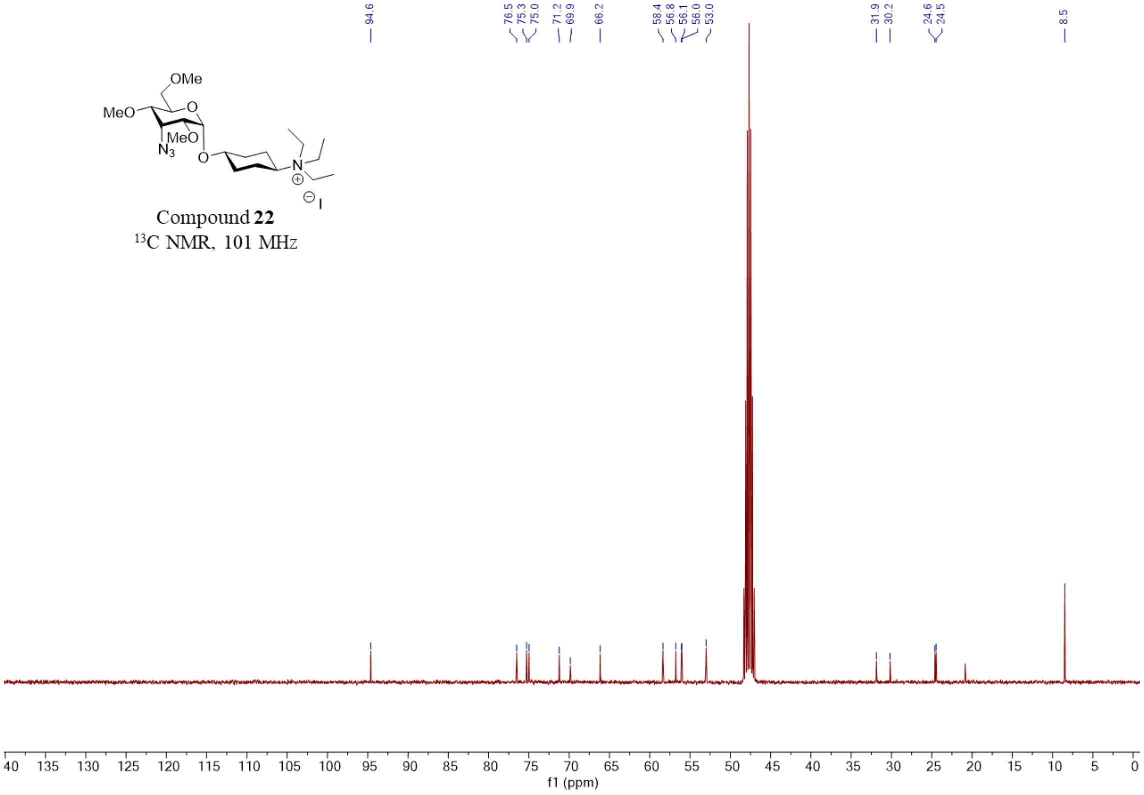
94.6
76.5
75.3
75.0
71.2
69.9
66.2
58.4
56.8
56.1
56.0
53.0
31.9
30.2
24.6
24.5
8.5
OMe
MeO
O
MeO
N3
O
N
I
Compound 22
13C NMR, 101 MHz
f1 (ppm)

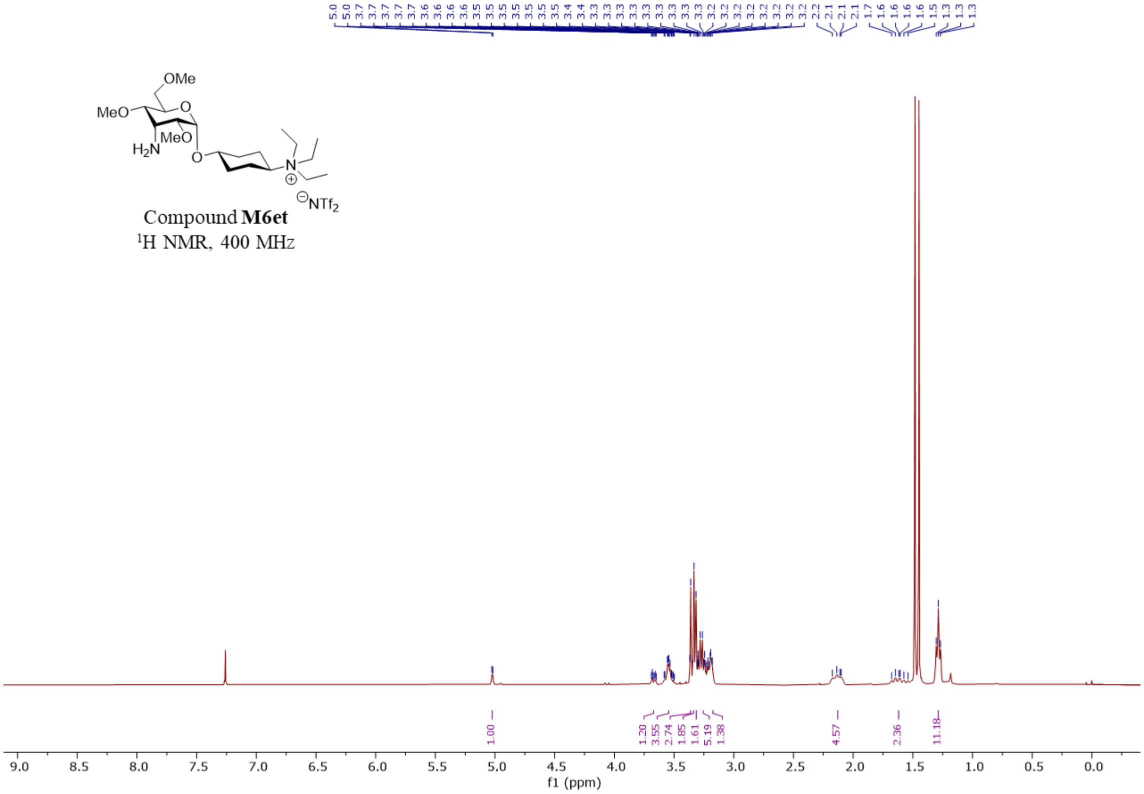
Compound **M6et**
^{1}H NMR, 400 MHz
f1 (ppm)

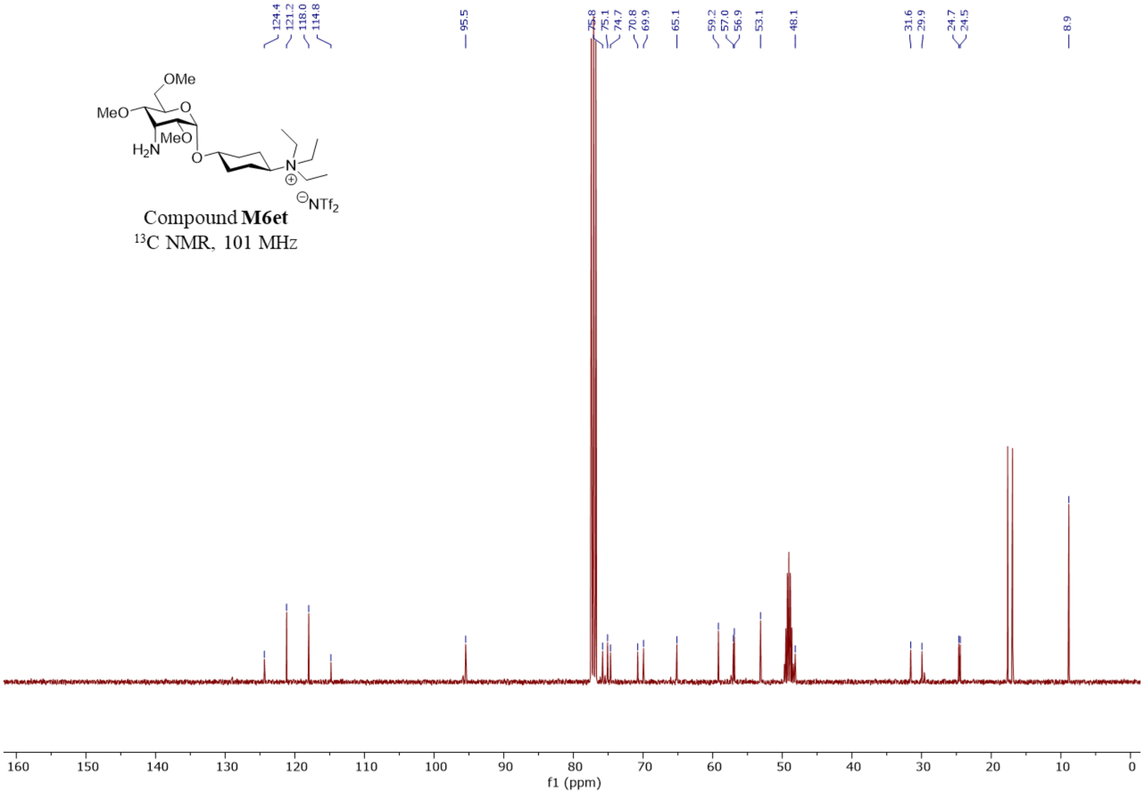
Compound **M6et**
^{13}C NMR, 101 MHz
f1 (ppm)

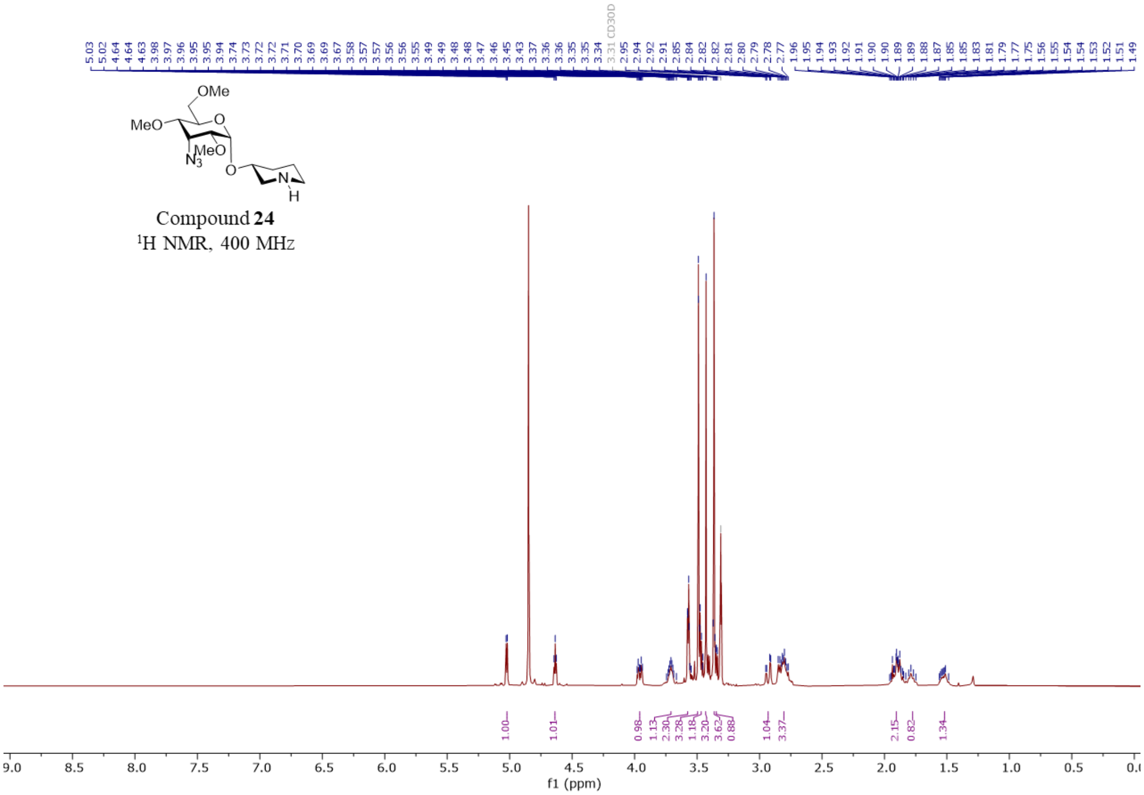
5.03
5.02
4.64
4.64
4.63
3.98
3.97
3.96
3.95
3.95
3.94
3.74
3.73
3.72
3.72
3.71
3.70
3.69
3.69
3.67
3.58
3.57
3.57
3.56
3.56
3.55
3.49
3.49
3.48
3.48
3.47
3.46
3.45
3.43
3.37
3.36
3.36
3.35
3.35
3.35
3.34
3.31 CD3OD
2.95
2.94
2.92
2.91
2.85
2.84
2.82
2.82
2.81
2.80
2.79
2.78
2.77
1.96
1.95
1.94
1.93
1.92
1.91
1.90
1.90
1.89
1.89
1.88
1.87
1.85
1.85
1.83
1.81
1.79
1.77
1.75
1.56
1.55
1.54
1.54
1.53
1.52
1.51
1.49
OMe
MeO
O
N3
MeO
O
N
H
Compound 24
1H NMR, 400 MHz
1.00
1.01
0.98
1.13
2.30
3.28
1.18
3.20
3.62
0.88
1.04
3.37
2.15
0.82
1.34
9.0
8.5
8.0
7.5
7.0
6.5
6.0
5.5
5.0
4.5
4.0
3.5
3.0
2.5
2.0
1.5
1.0
0.5
0.0
f1 (ppm)

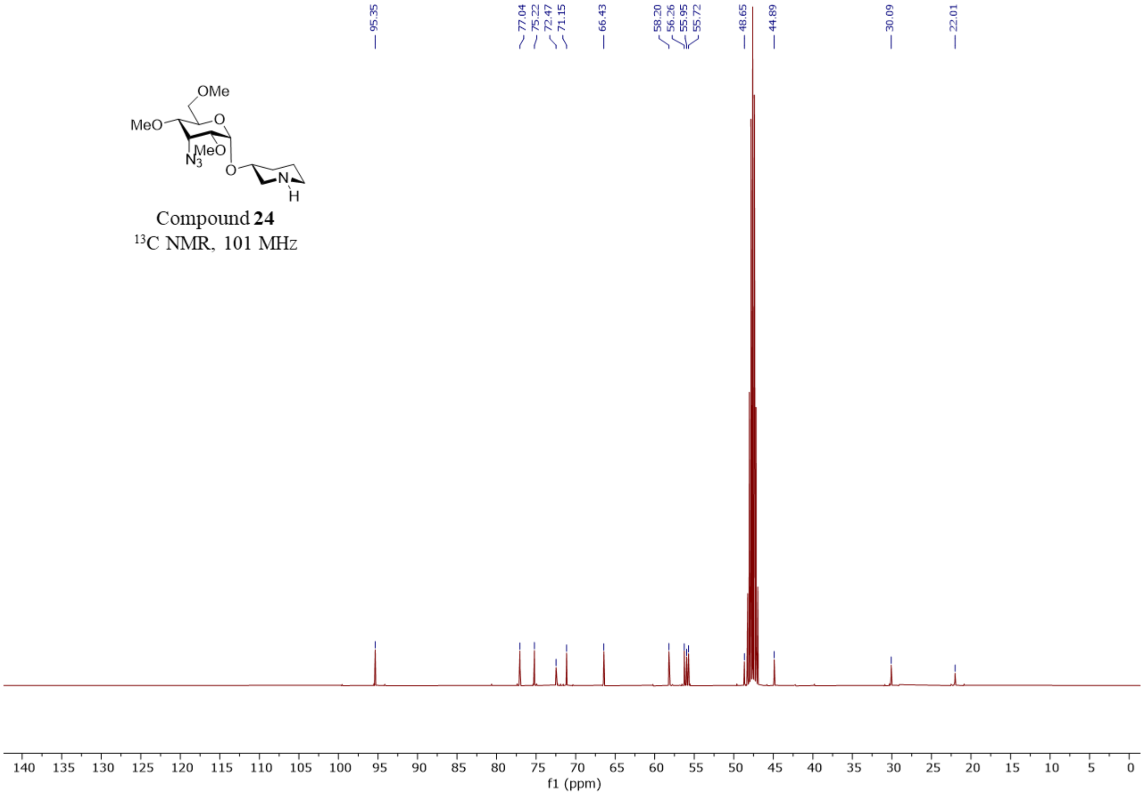
95.35
77.04
75.22
72.47
71.15
66.43
58.20
56.26
55.95
55.72
48.65
44.89
30.09
22.01
OMe
MeO
O
N3
MeO
O
N
H
Compound 24
13C NMR, 101 MHz
140
135
130
125
120
115
110
105
100
95
90
85
80
75
70
65
60
55
50
45
40
35
30
25
20
15
10
5
0
f1 (ppm)

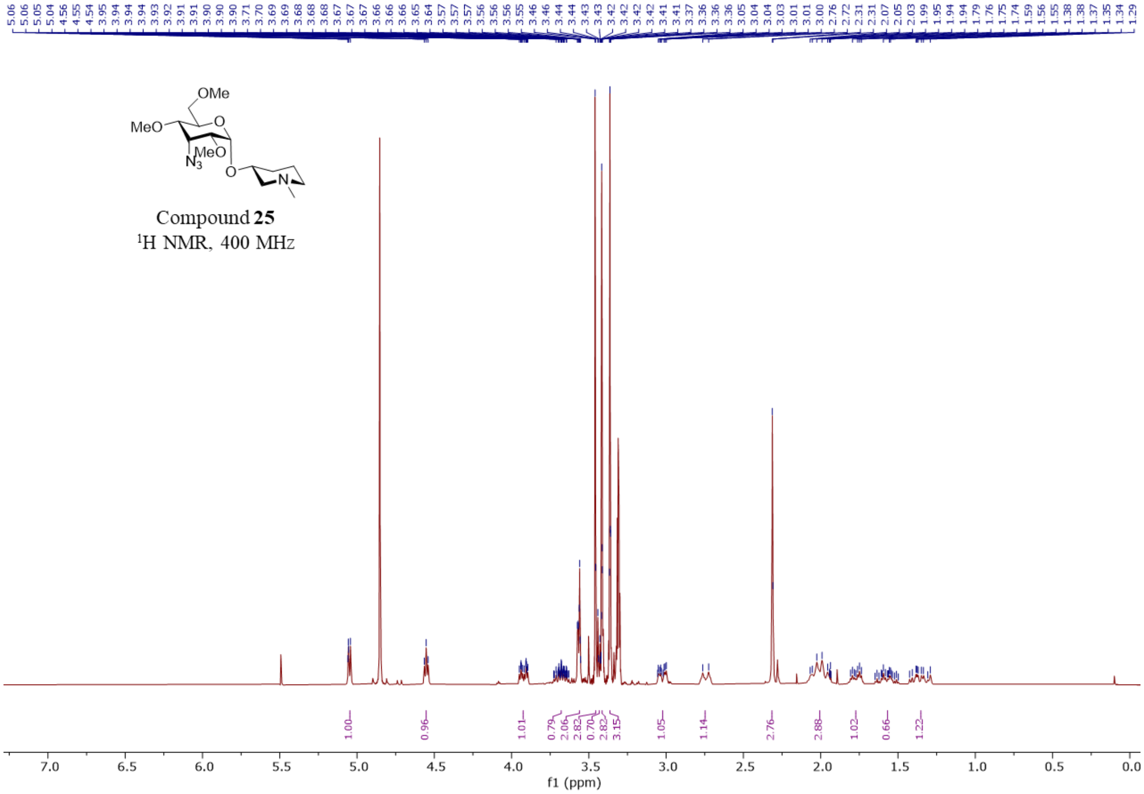
OMe
MeO
O
MeO
N3
O
N
Compound 25
1H NMR, 400 MHz
f1 (ppm)

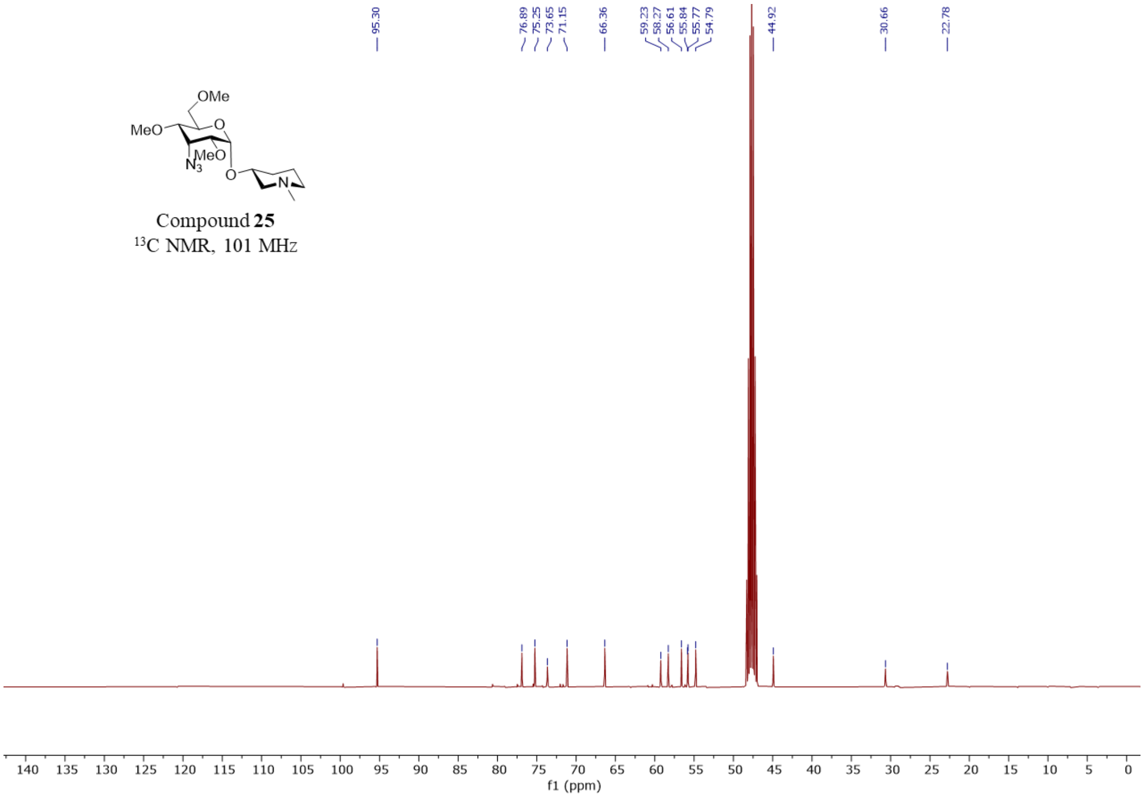
95.30
76.89
75.25
73.65
71.15
66.36
59.23
58.27
56.61
55.84
55.77
54.79
44.92
30.66
22.78
OMe
MeO
O
MeO
N3
O
N
Compound 25
13C NMR, 101 MHz
f1 (ppm)

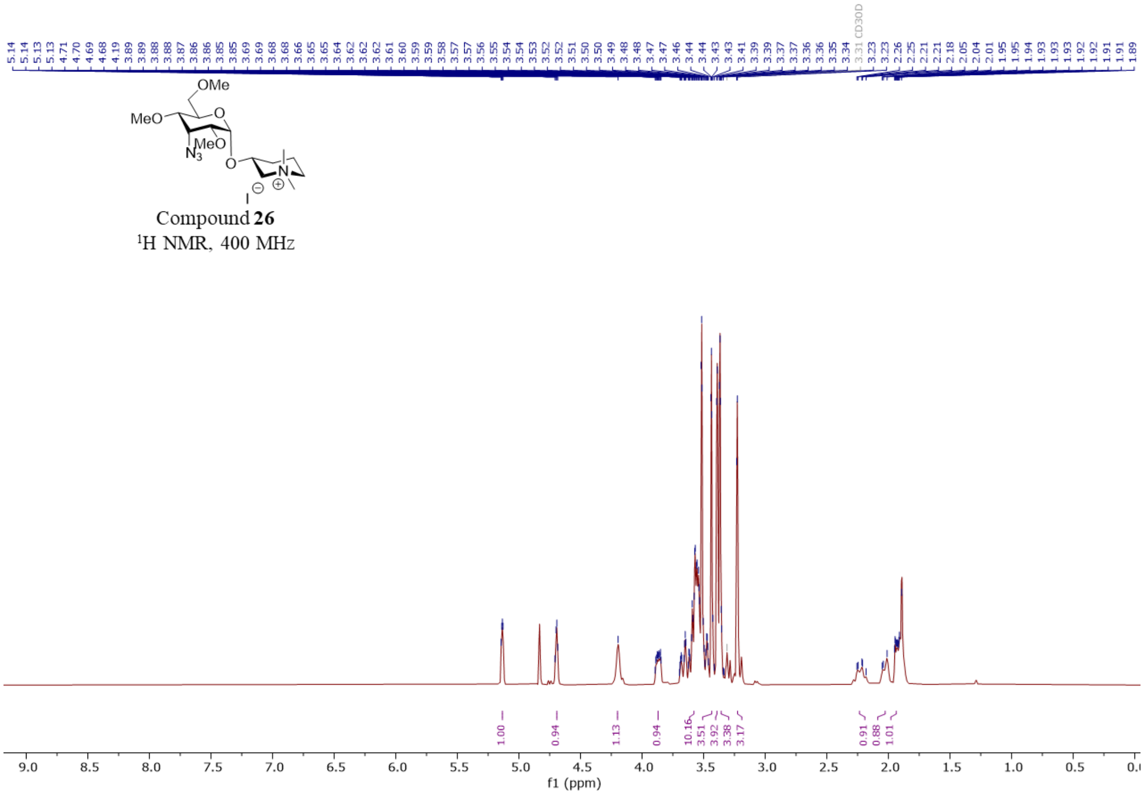

Compound **26**
^{1}H NMR, 400 MHz

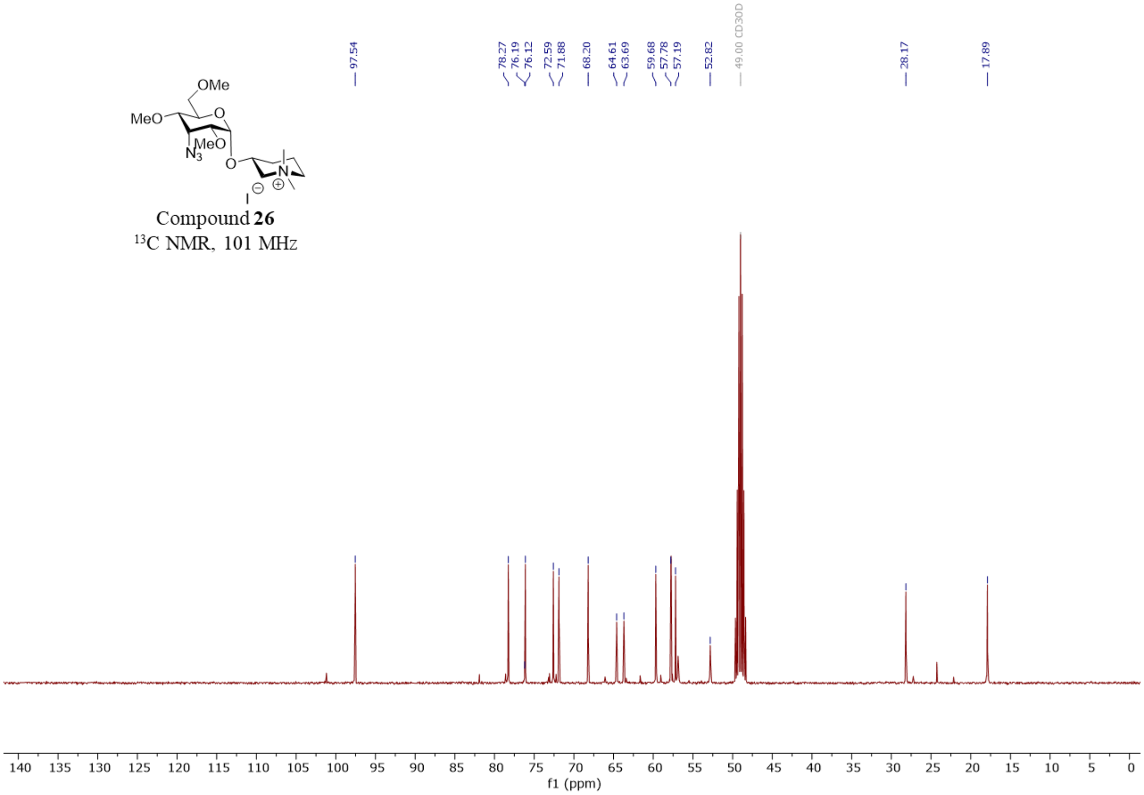

Compound **26**
^{13}C NMR, 101 MHz

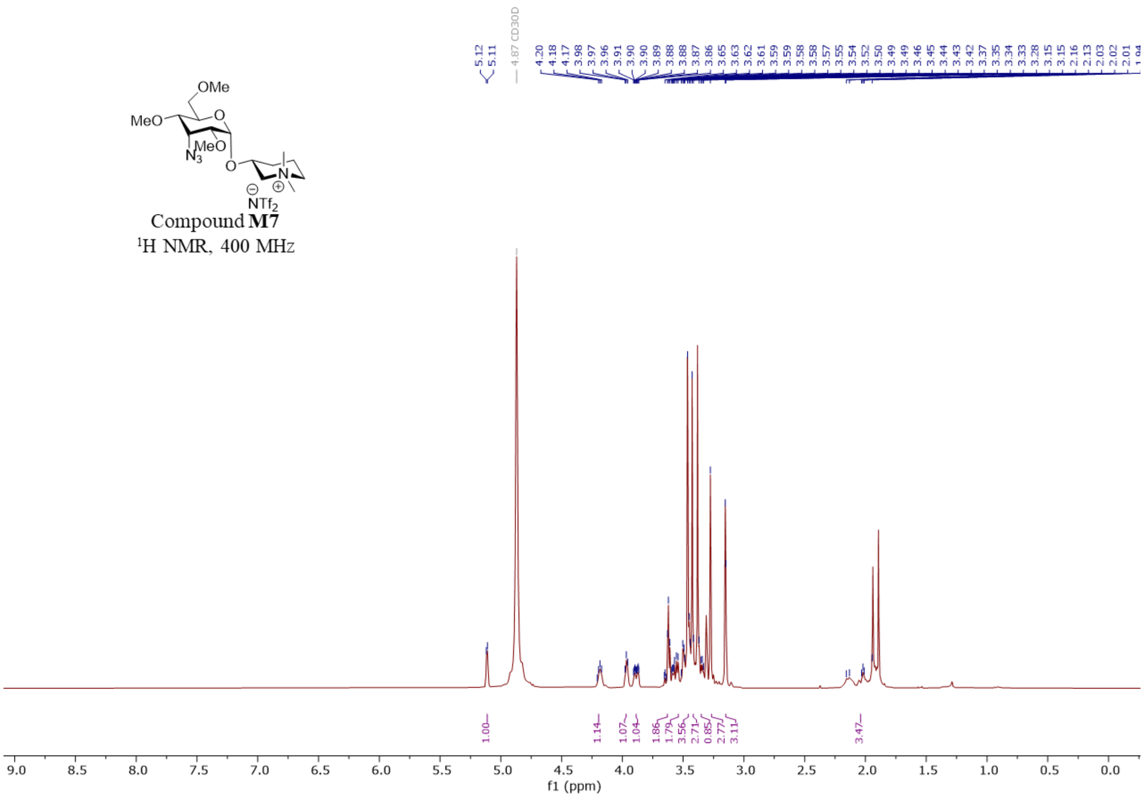

Compound **M7**
^{1}H NMR, 400 MHz

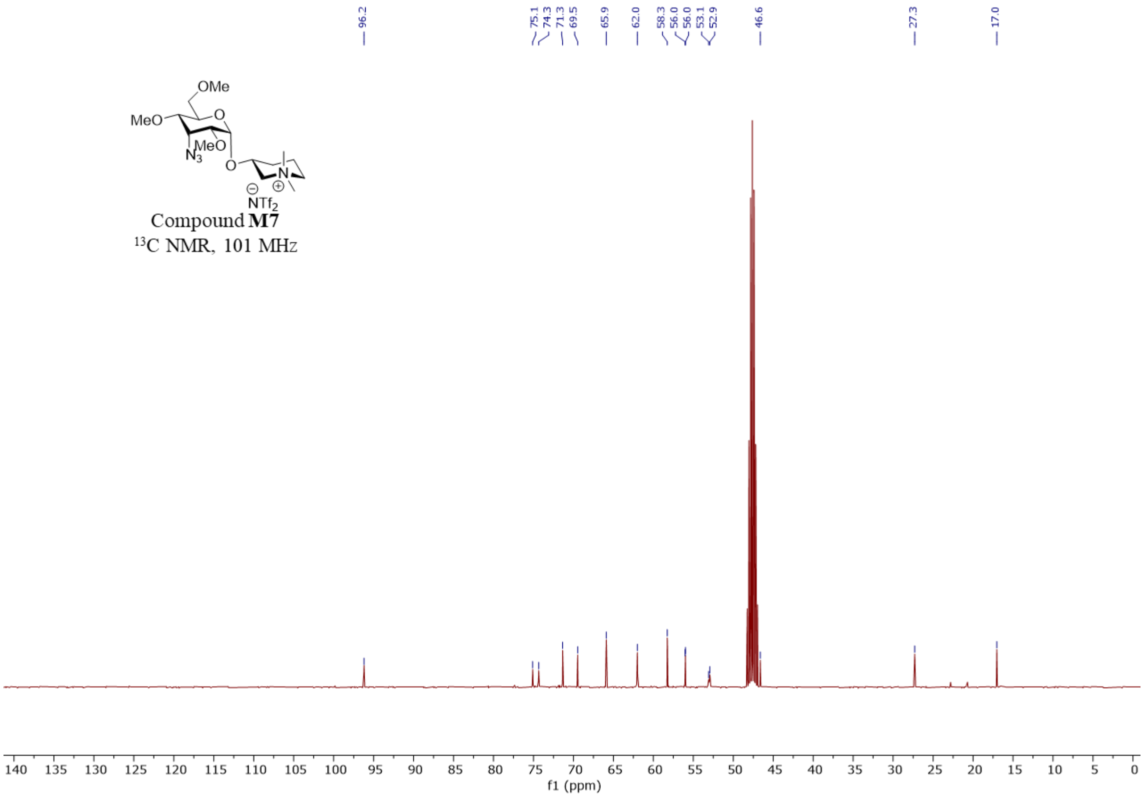

Compound **M7**
^{13}C NMR, 101 MHz

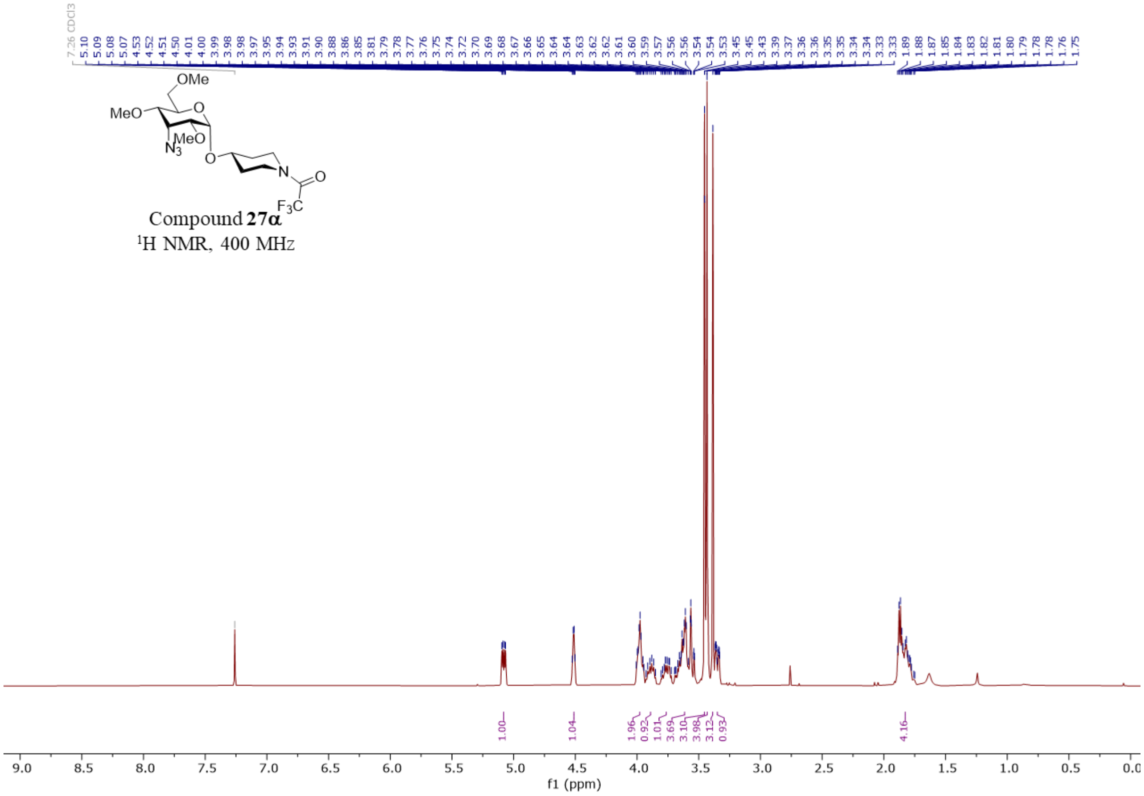

OMe
MeO
O
MeO
N3
O
N
O
F3C
Compound 27α
1H NMR, 400 MHz
f1 (ppm)

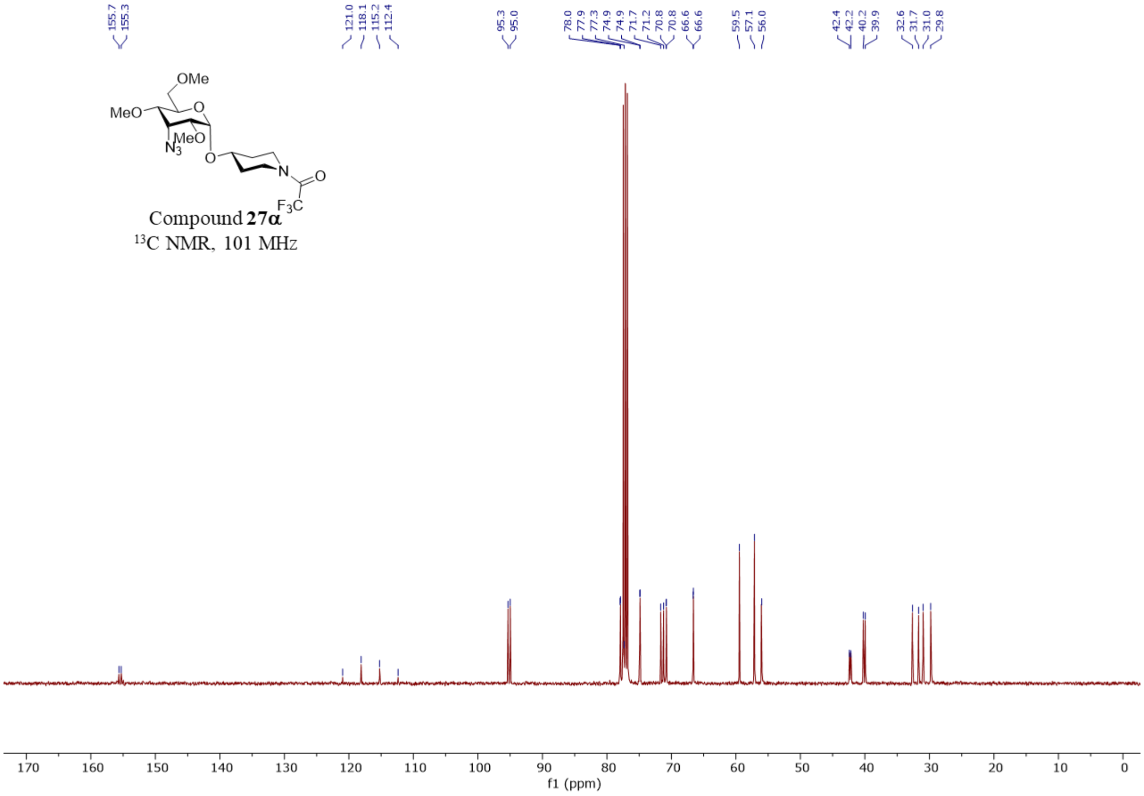

OMe
MeO
O
MeO
N3
O
N
O
F3C
Compound 27α
13C NMR, 101 MHz
f1 (ppm)

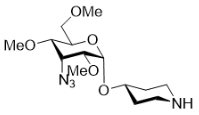

Compound **28**
^{1}H NMR, 400 MHz

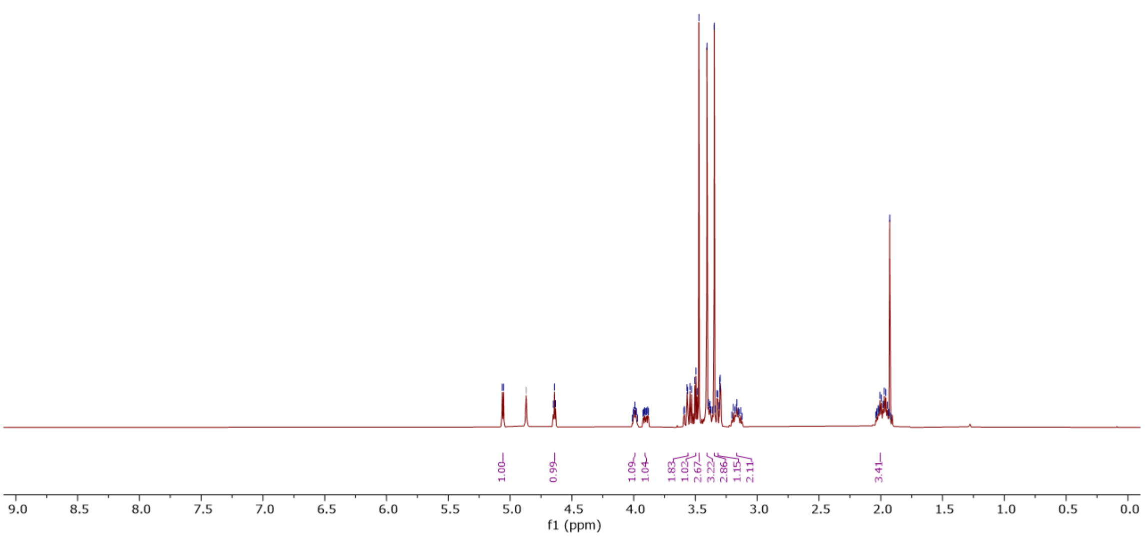

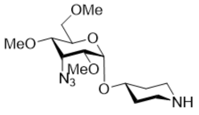

Compound **28**
^{13}C NMR, 101 MHz

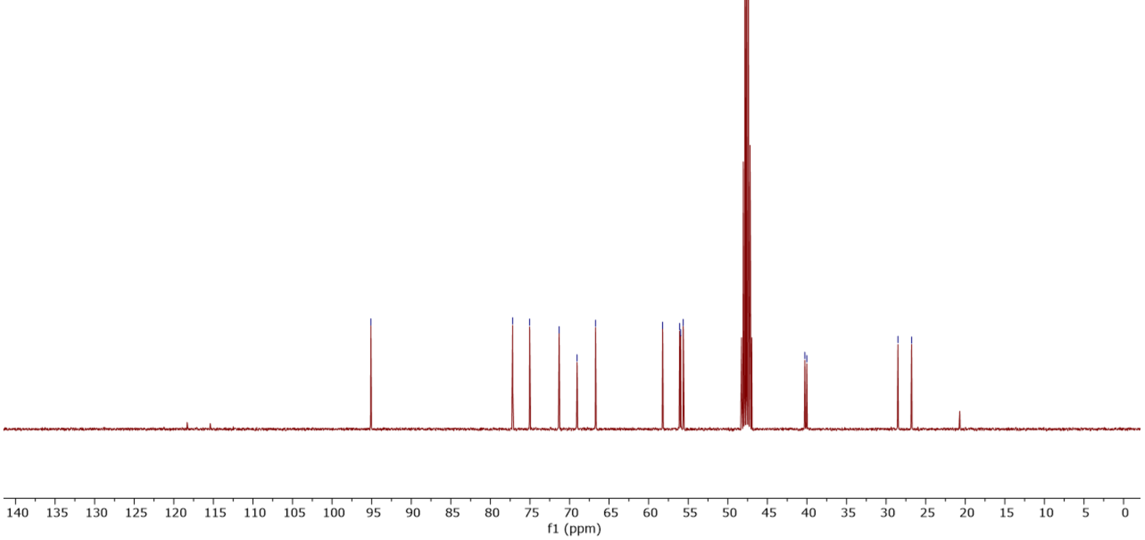

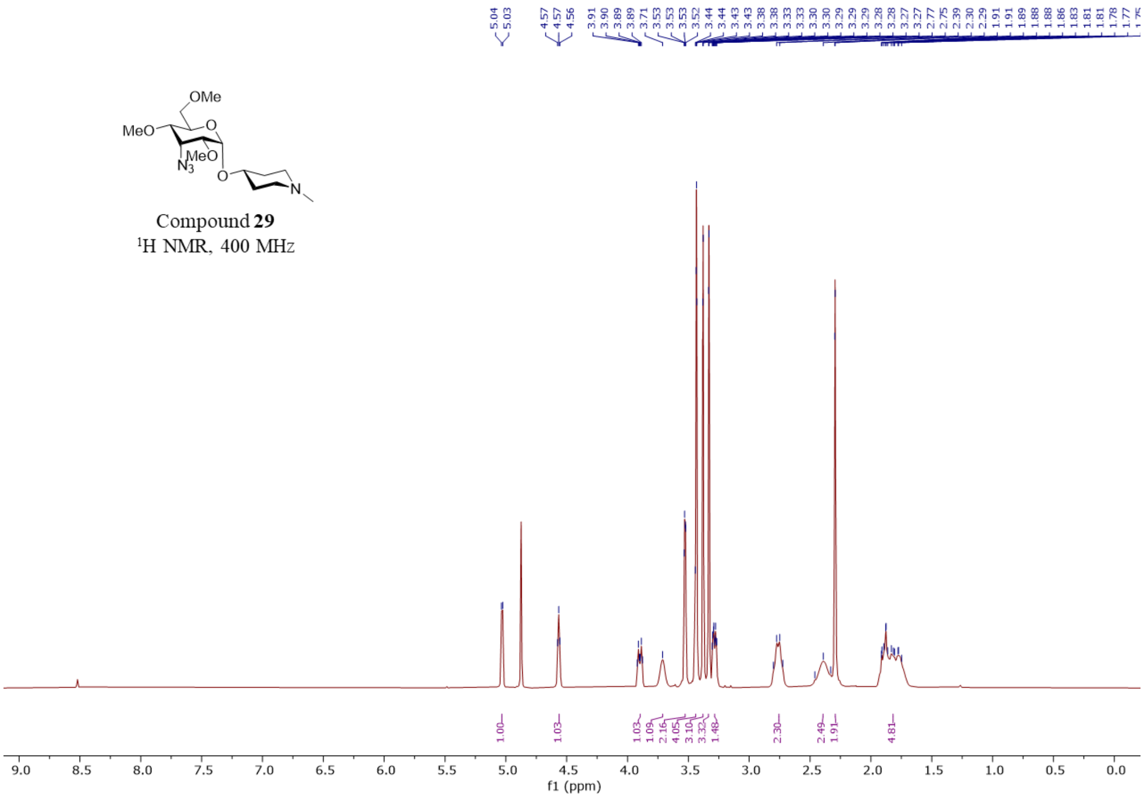
OMe
MeO
O
MeO
N3
O
N
Compound 29
1H NMR, 400 MHz
f1 (ppm)

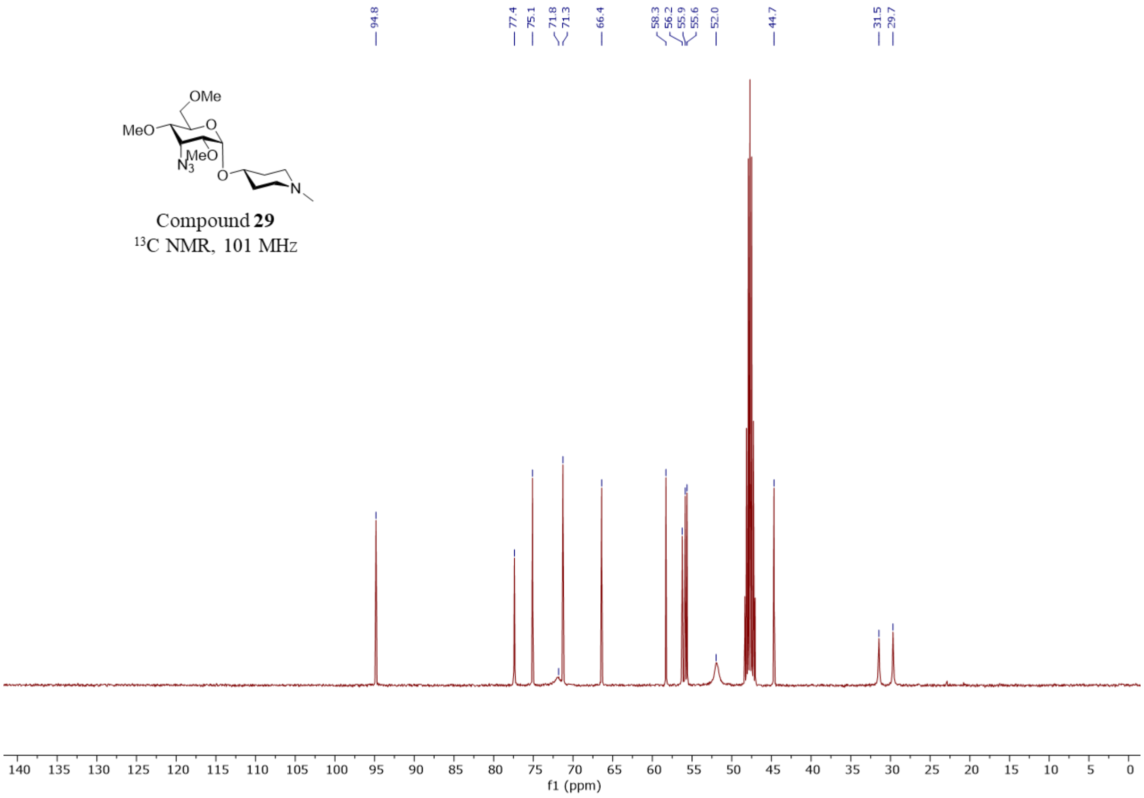
OMe
MeO
O
MeO
N3
O
N
Compound 29
13C NMR, 101 MHz
94.8
77.4
75.1
71.8
71.3
66.4
58.3
56.2
55.9
55.6
52.0
44.7
31.5
29.7
f1 (ppm)

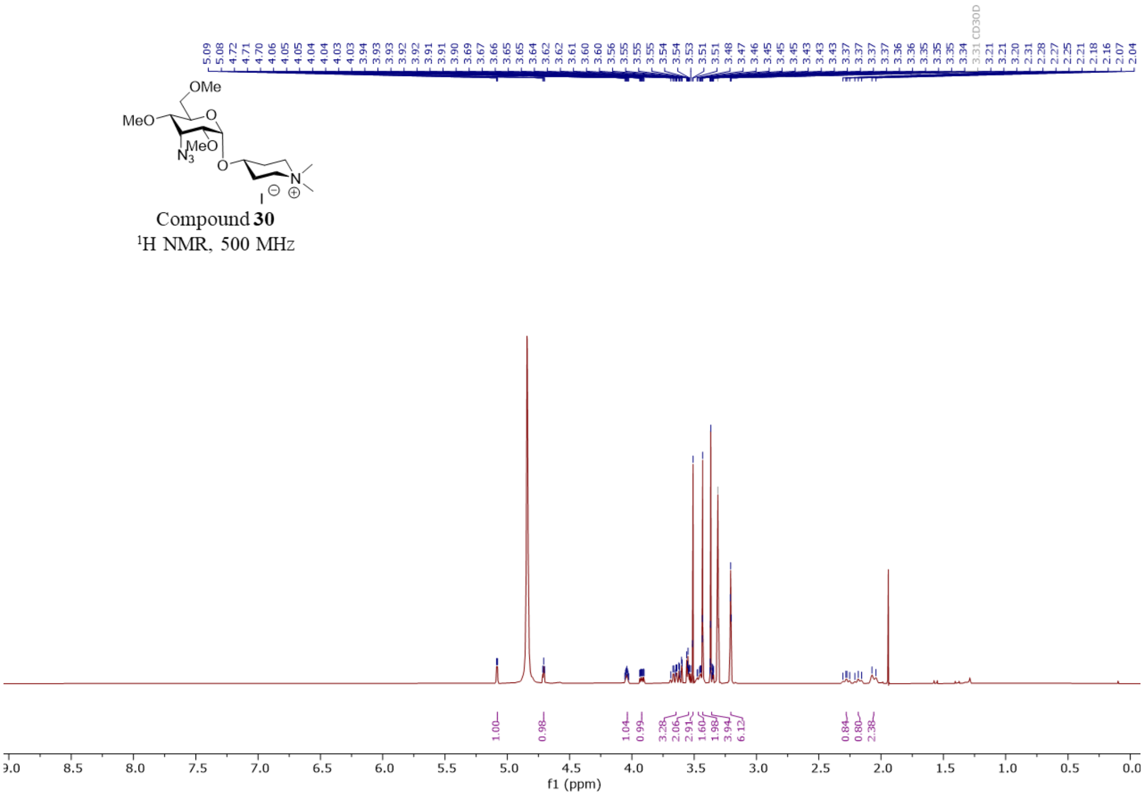

Compound **30**
^{1}H NMR, 500 MHz

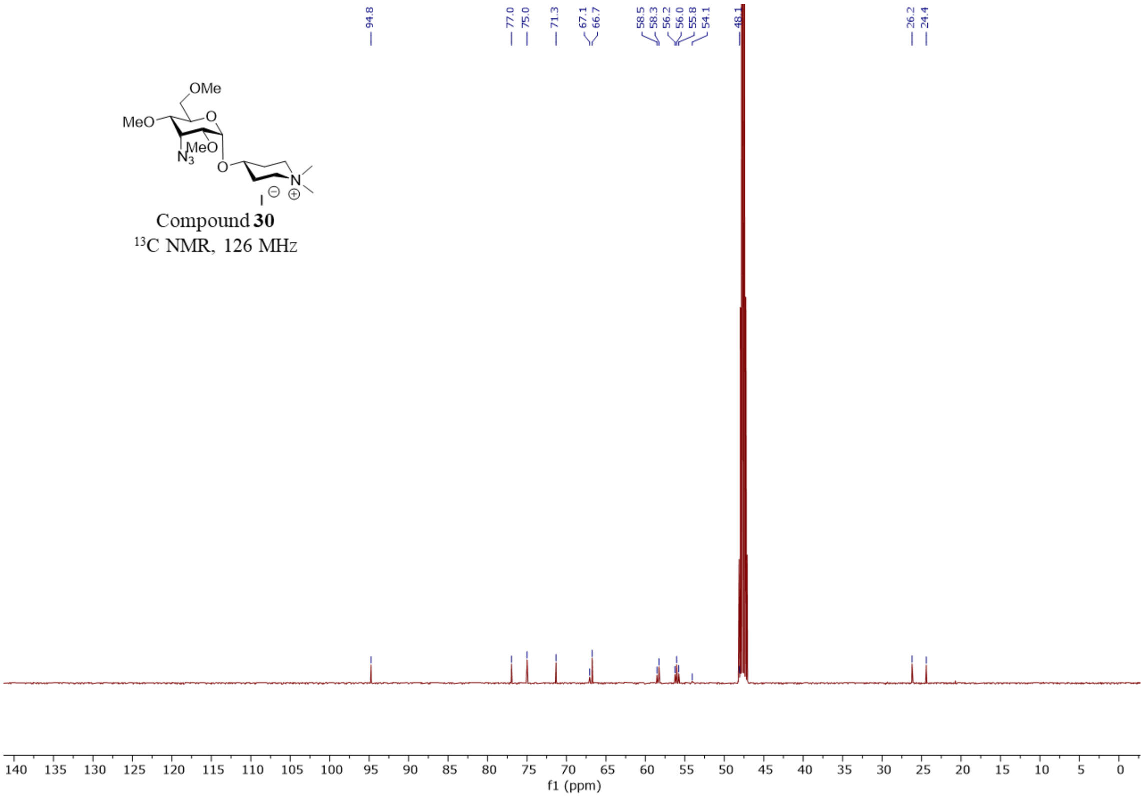

Compound **30**
^{13}C NMR, 126 MHz

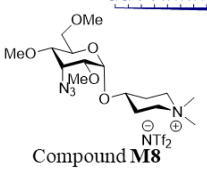

Compound **M8**
^{1}H NMR, 400 MHz

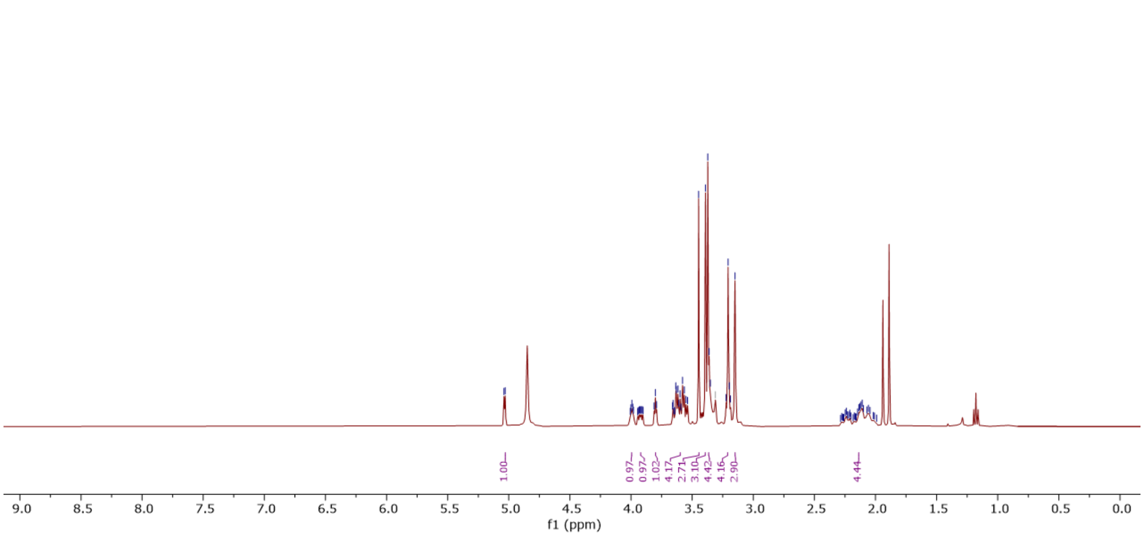

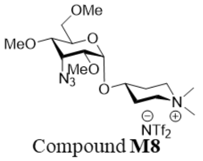

Compound **M8**
^{13}C NMR, 101 MHz

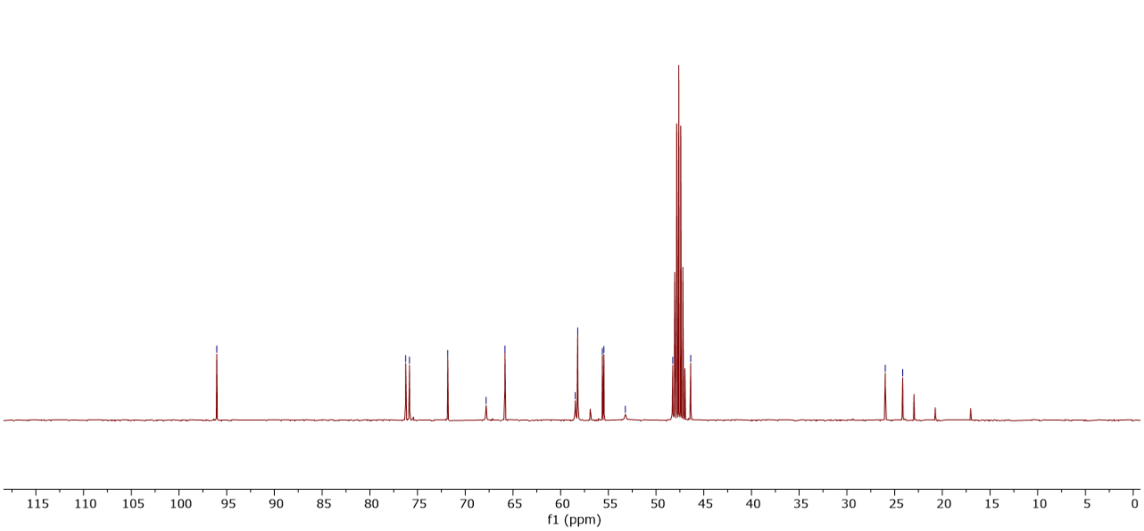

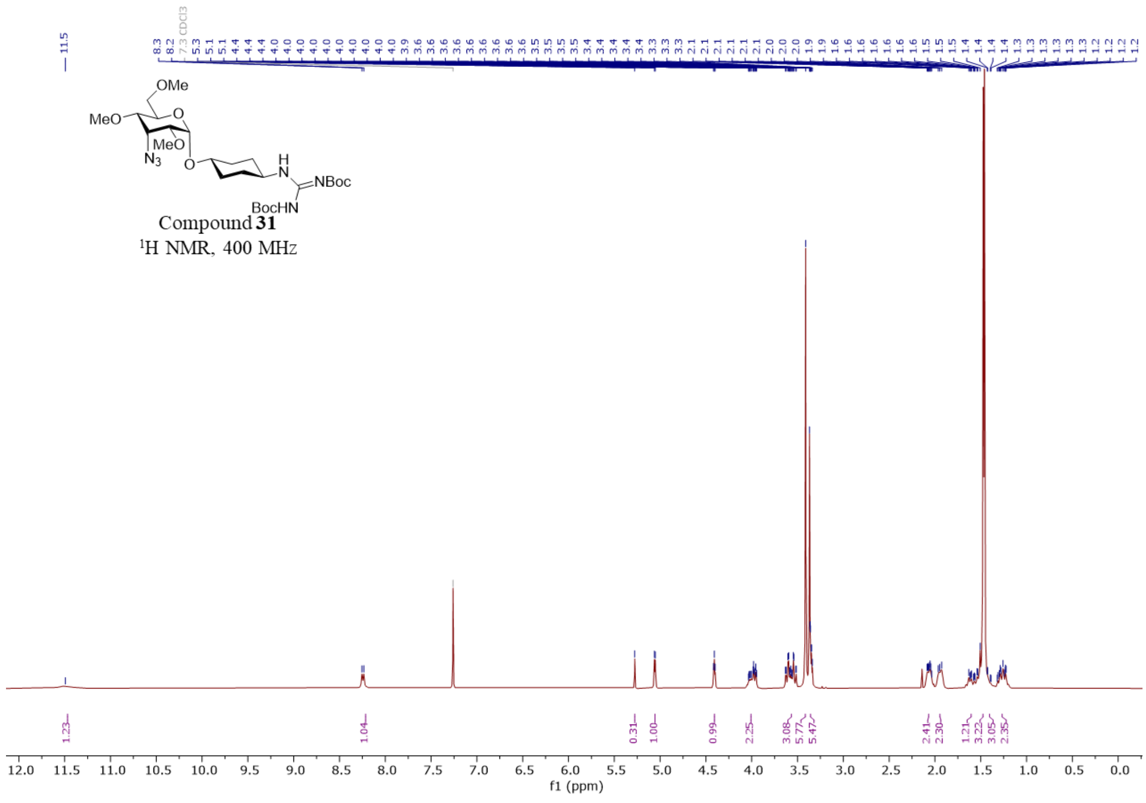
OMe
MeO
O
MeO
N3
O
H
N
NBoc
BocHN
Compound 31
1H NMR, 400 MHz
f1 (ppm)

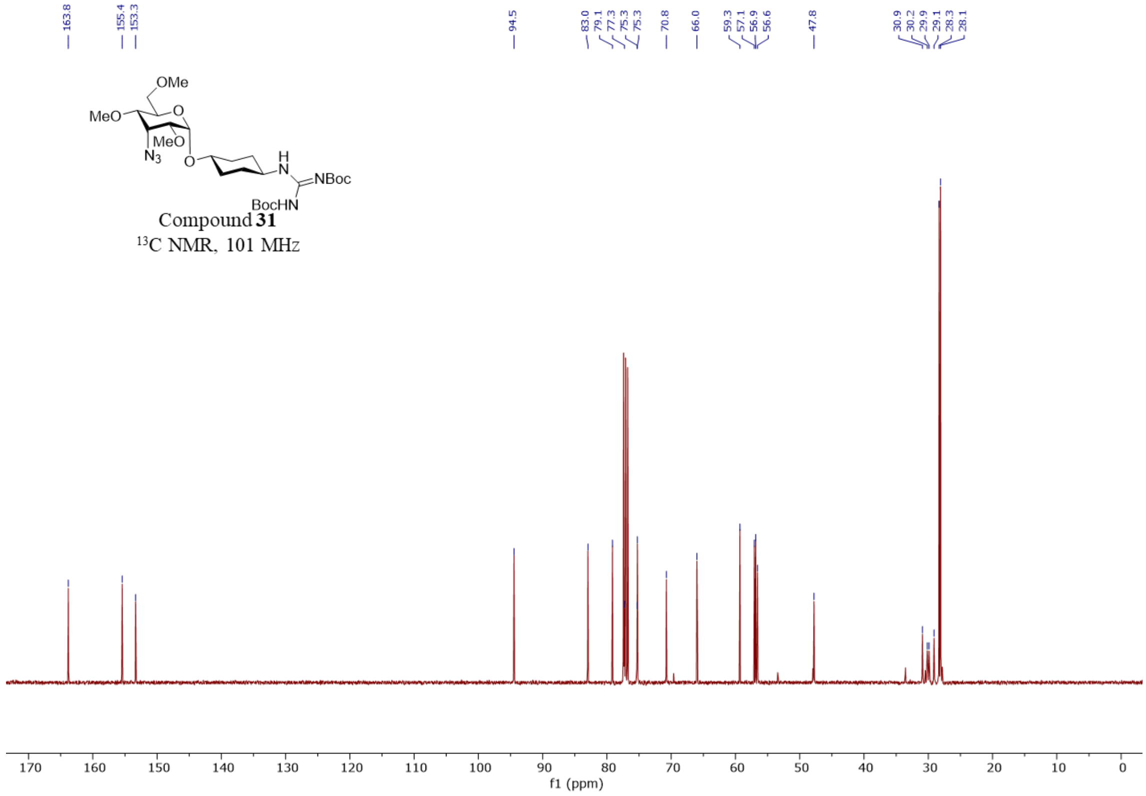
OMe
MeO
O
MeO
N3
O
H
N
NBoc
BocHN
Compound 31
13C NMR, 101 MHz
f1 (ppm)

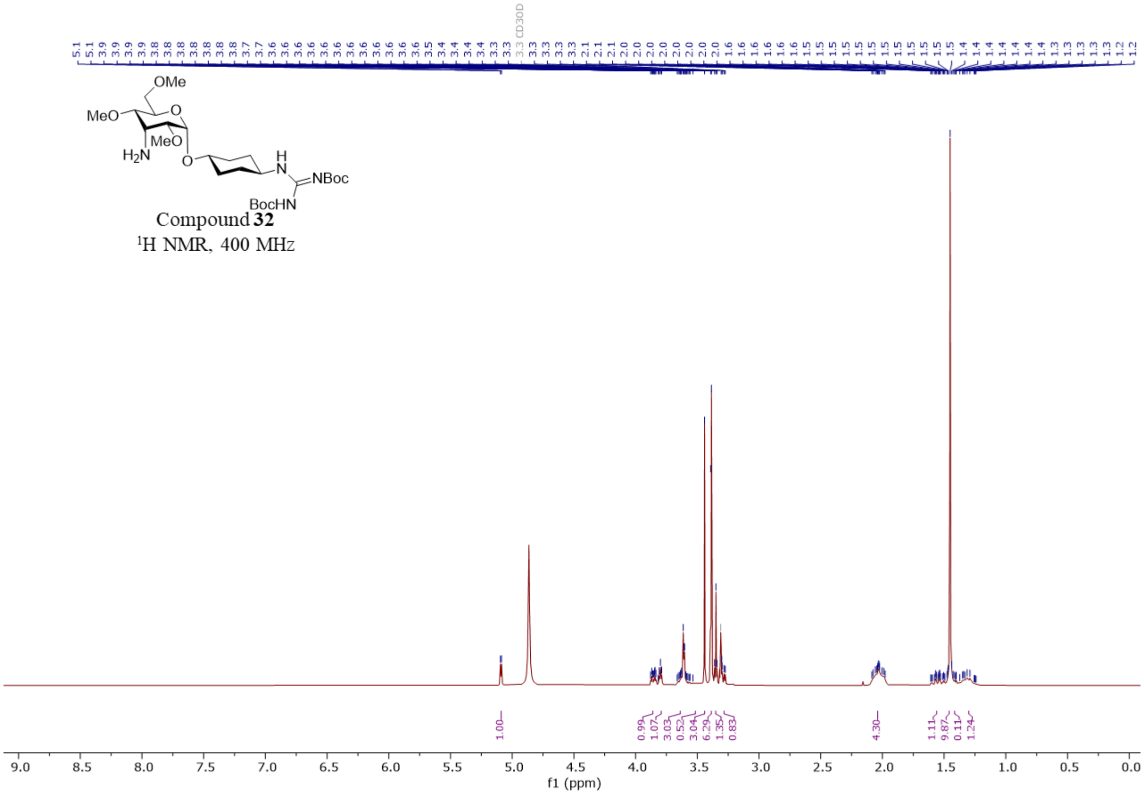
OMe
MeO
O
MeO
H2N
O
H
N
NBoc
BocHN
Compound 32
1H NMR, 400 MHz
CD3OD
1.00
0.99
1.07
3.03
0.52
3.04
6.29
1.35
0.83
4.30
1.11
9.87
0.11
1.24
f1 (ppm)

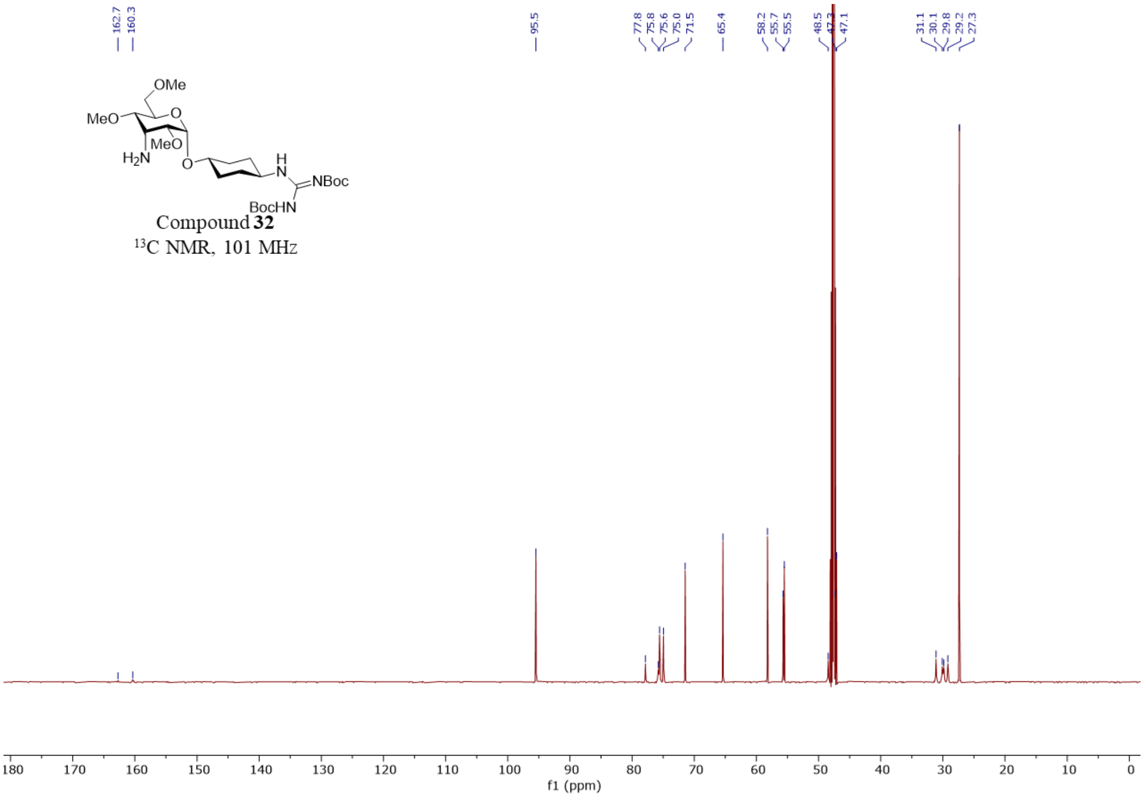
162.7
160.3
95.5
77.8
75.8
75.6
75.0
71.5
65.4
58.2
55.7
55.5
48.5
47.3
47.1
31.1
30.1
29.8
29.2
27.3
OMe
MeO
O
MeO
H2N
O
H
N
NBoc
BocHN
Compound 32
13C NMR, 101 MHz
f1 (ppm)

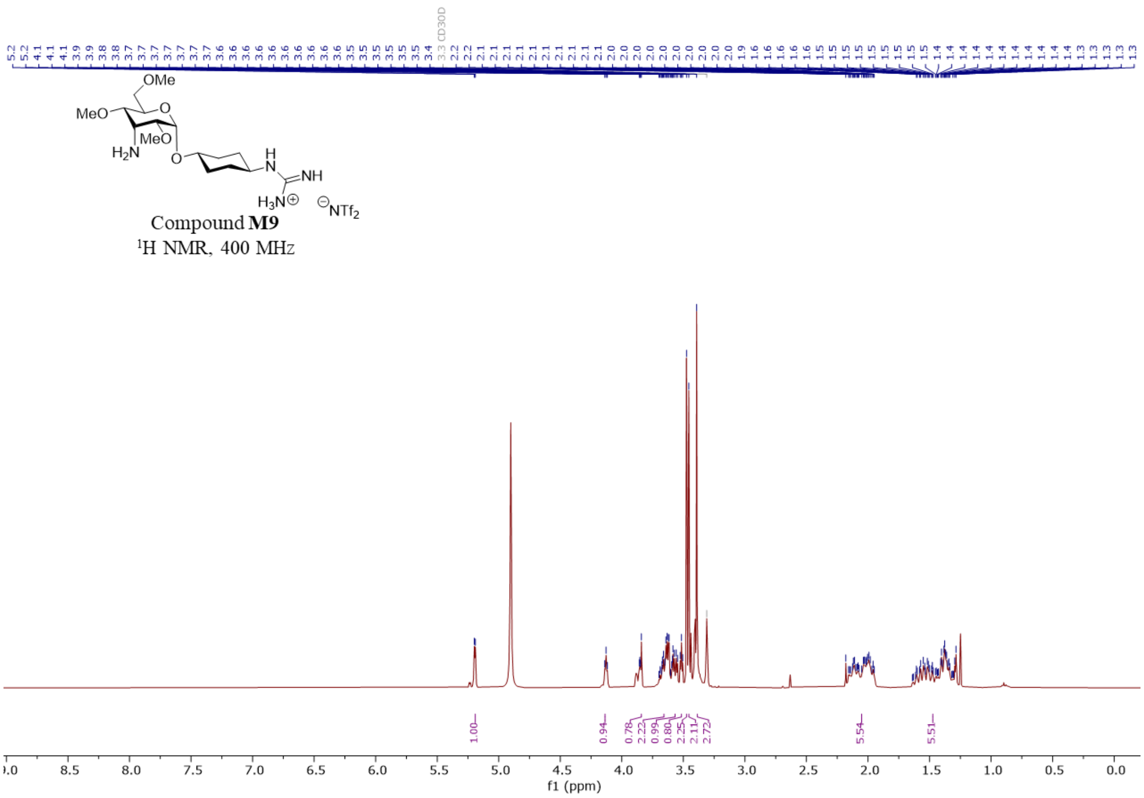
Compound M9
1H NMR, 400 MHz
f1 (ppm)

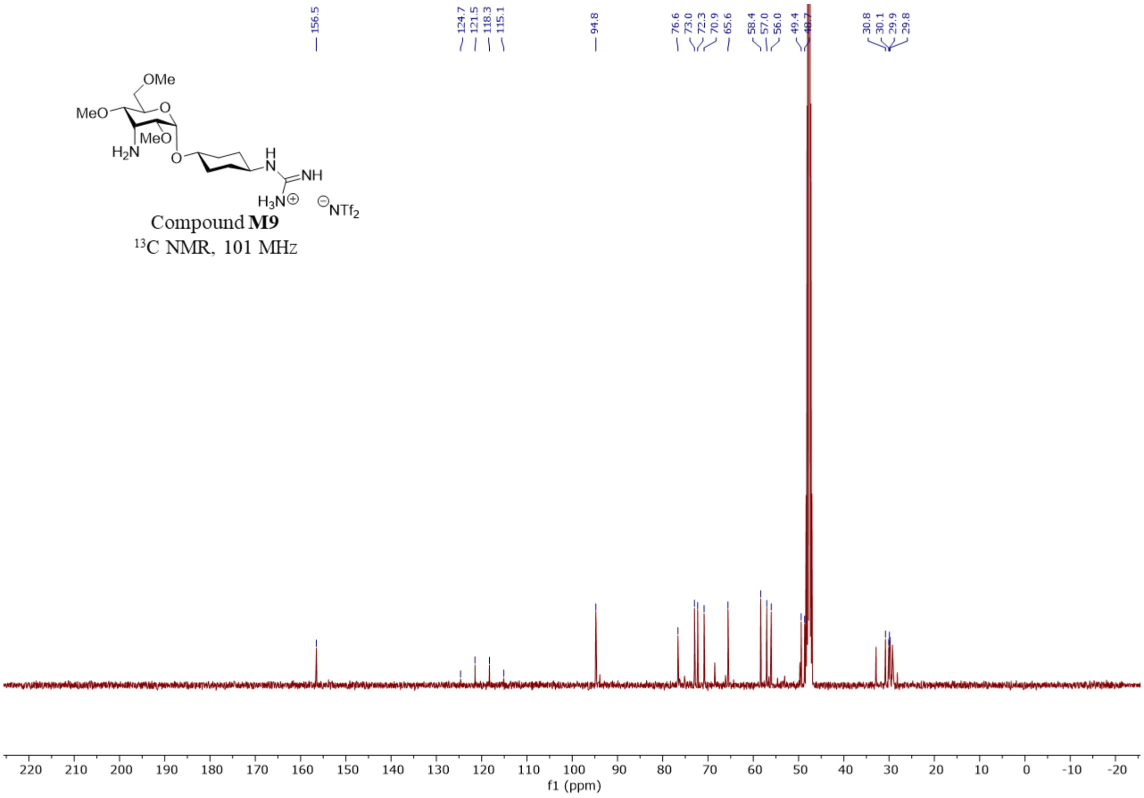
Compound M9
13C NMR, 101 MHz
156.5
124.7
121.5
118.3
115.1
94.8
76.6
73.0
72.3
70.9
65.6
58.4
57.0
56.0
49.4
30.8
30.1
29.9
29.8
f1 (ppm)

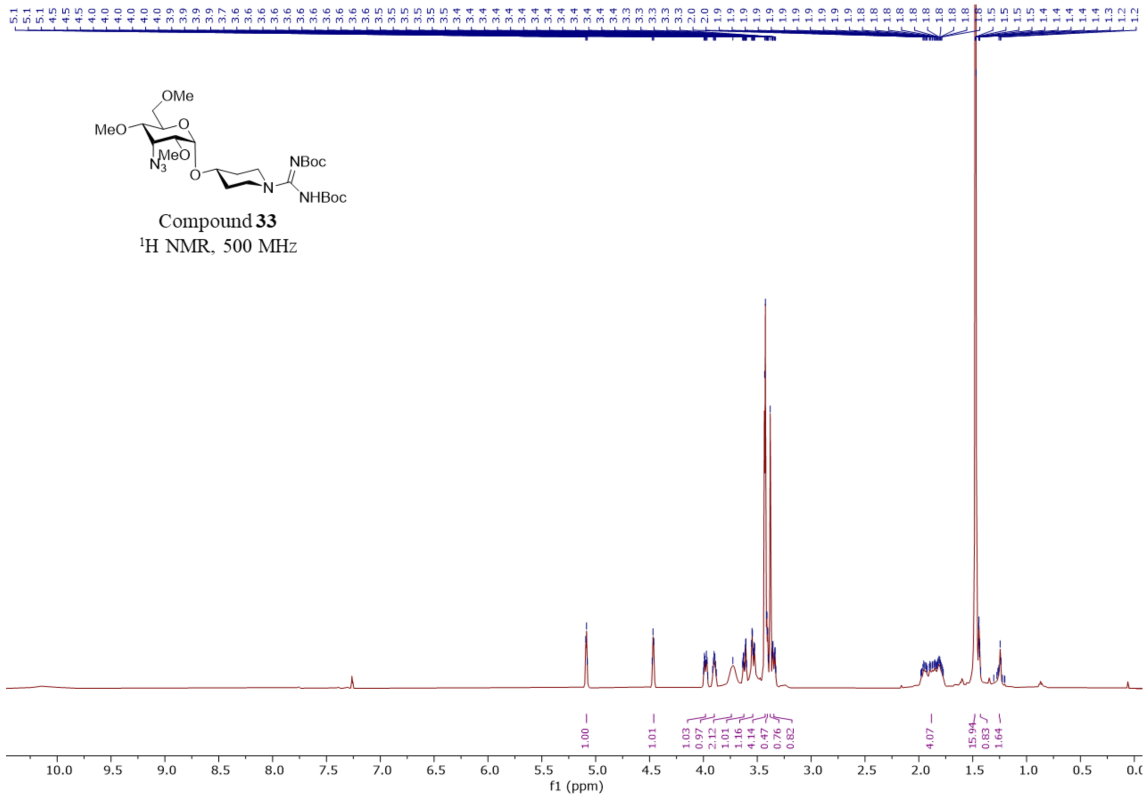

Compound **33**
^{1}H NMR, 500 MHz

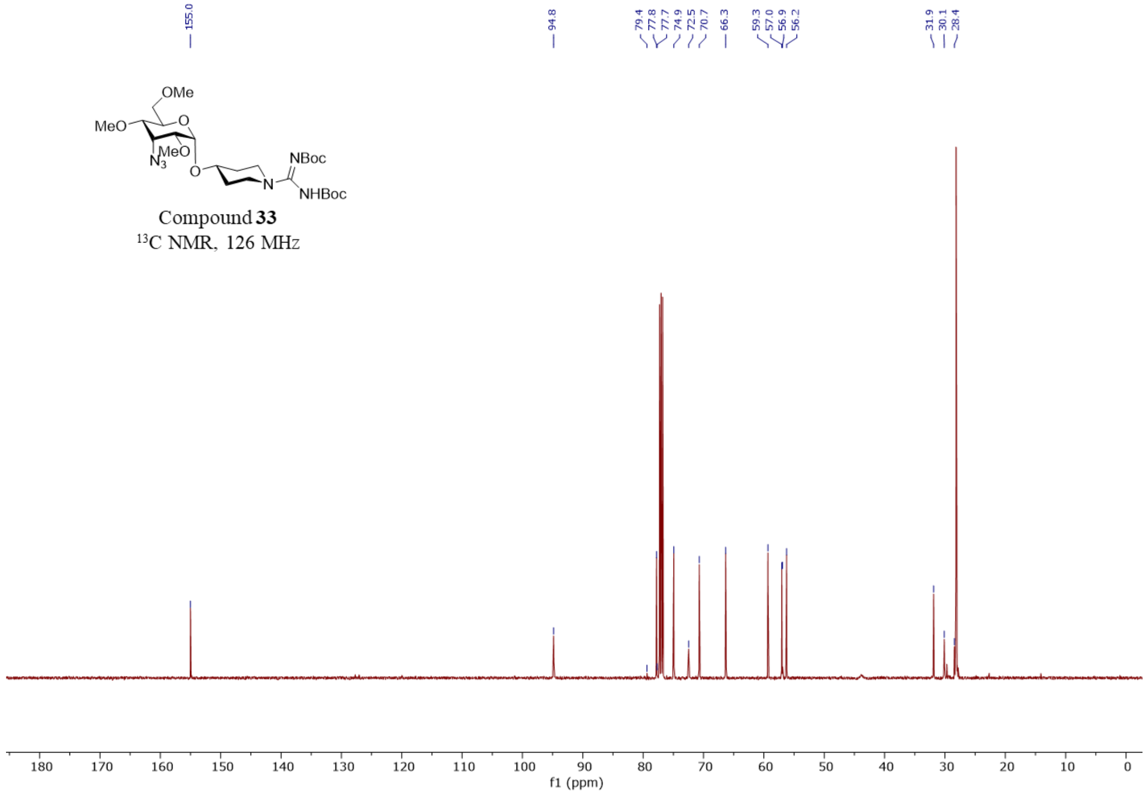

Compound **33**
^{13}C NMR, 126 MHz

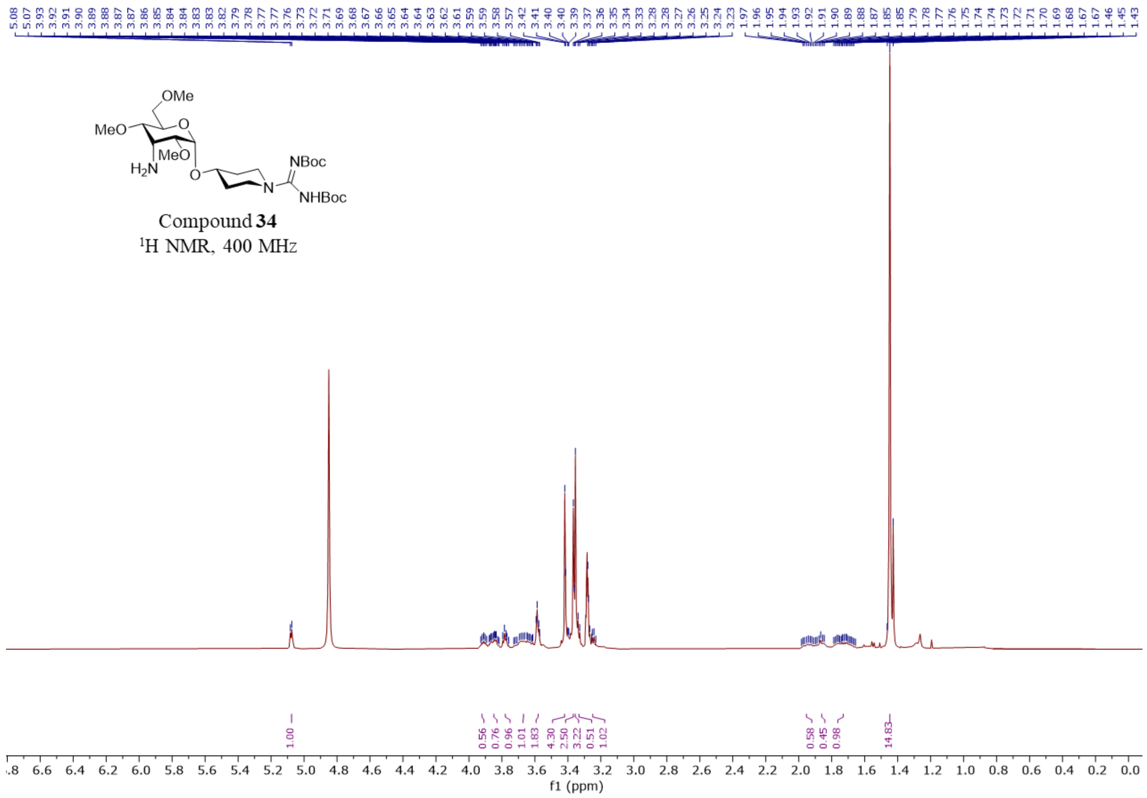
OMe
MeO
O
MeO
H_2N
O
NBoc
N
NHBoc
Compound **34**
^{1}H NMR, 400 MHz
1.00
0.56
0.76
0.96
1.01
1.83
4.30
2.50
3.22
0.51
1.02
0.58
0.45
0.98
14.83
f1 (ppm)

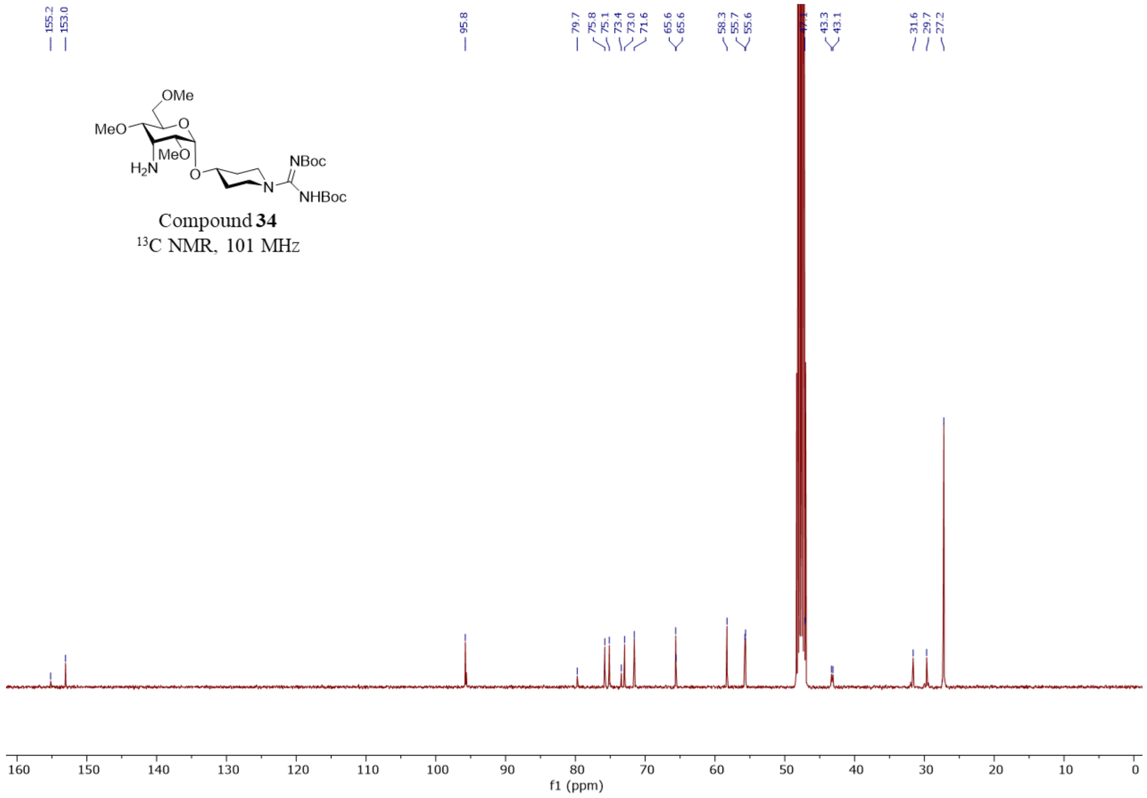
155.2
153.0
95.8
79.7
75.8
75.1
73.4
73.0
71.6
65.6
65.6
58.3
55.7
55.6
43.3
43.1
31.6
29.7
27.2
OMe
MeO
O
MeO
H_2N
O
NBoc
N
NHBoc
Compound **34**
^{13}C NMR, 101 MHz
f1 (ppm)

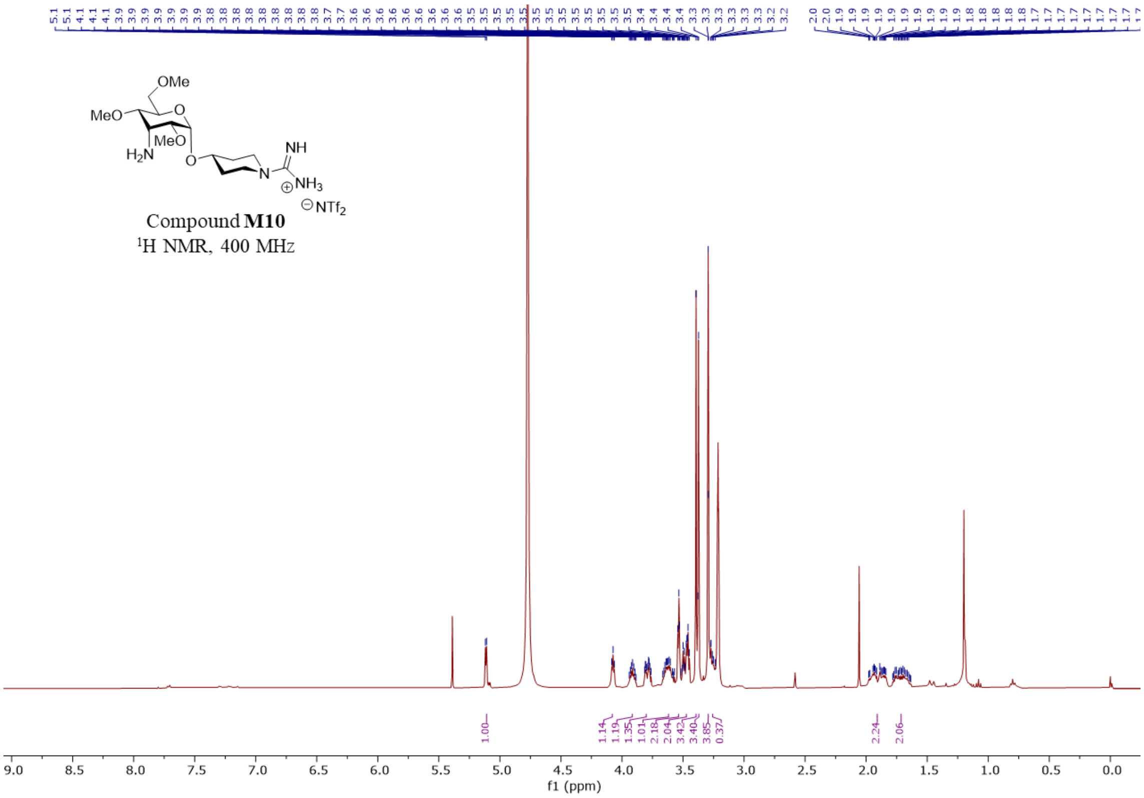
OMe
MeO
O
MeO
H2N
O
NH
N
NH3
NTf2
Compound **M10**
1H NMR, 400 MHz
1.00
1.14
1.19
1.35
1.01
2.18
2.04
3.42
3.40
3.85
0.37
2.24
2.06
f1 (ppm)

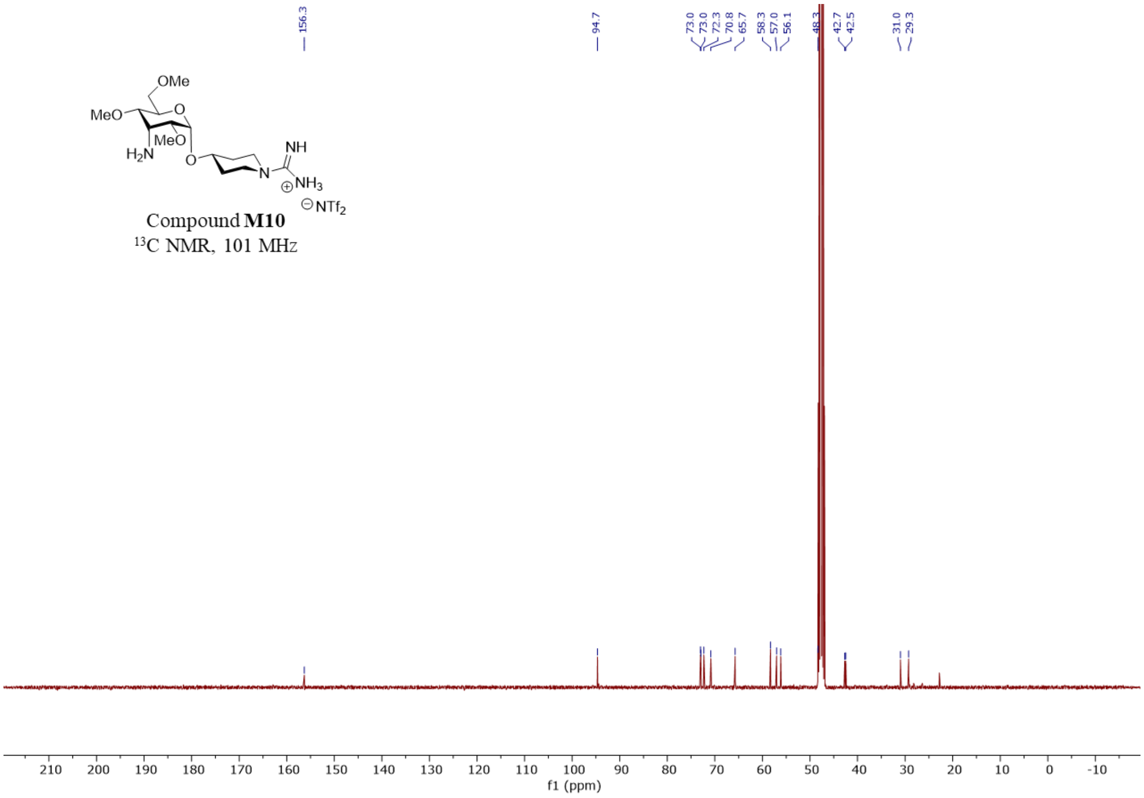
156.3
94.7
73.0
73.0
72.3
70.8
65.7
58.3
57.0
56.1
48.3
42.7
42.5
31.0
29.3
OMe
MeO
O
MeO
H2N
O
NH
N
NH3
NTf2
Compound **M10**
13C NMR, 101 MHz
f1 (ppm)

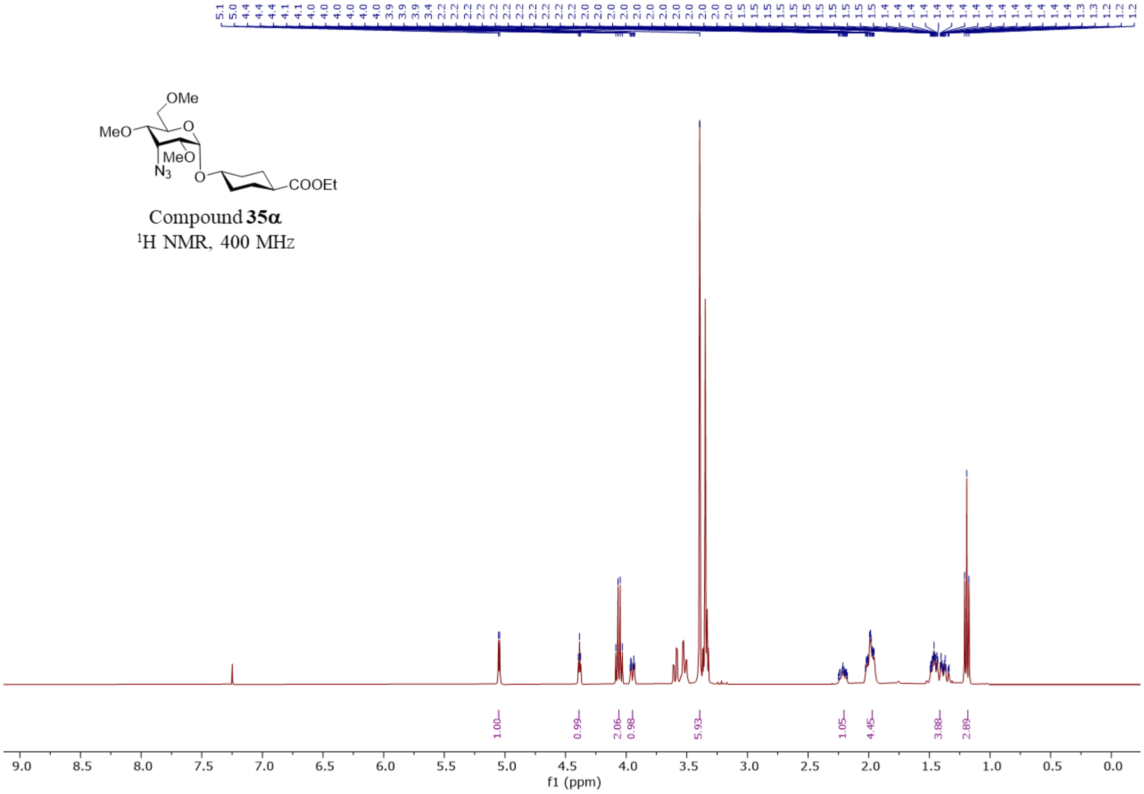

Compound **35α**
^{1}H NMR, 400 MHz

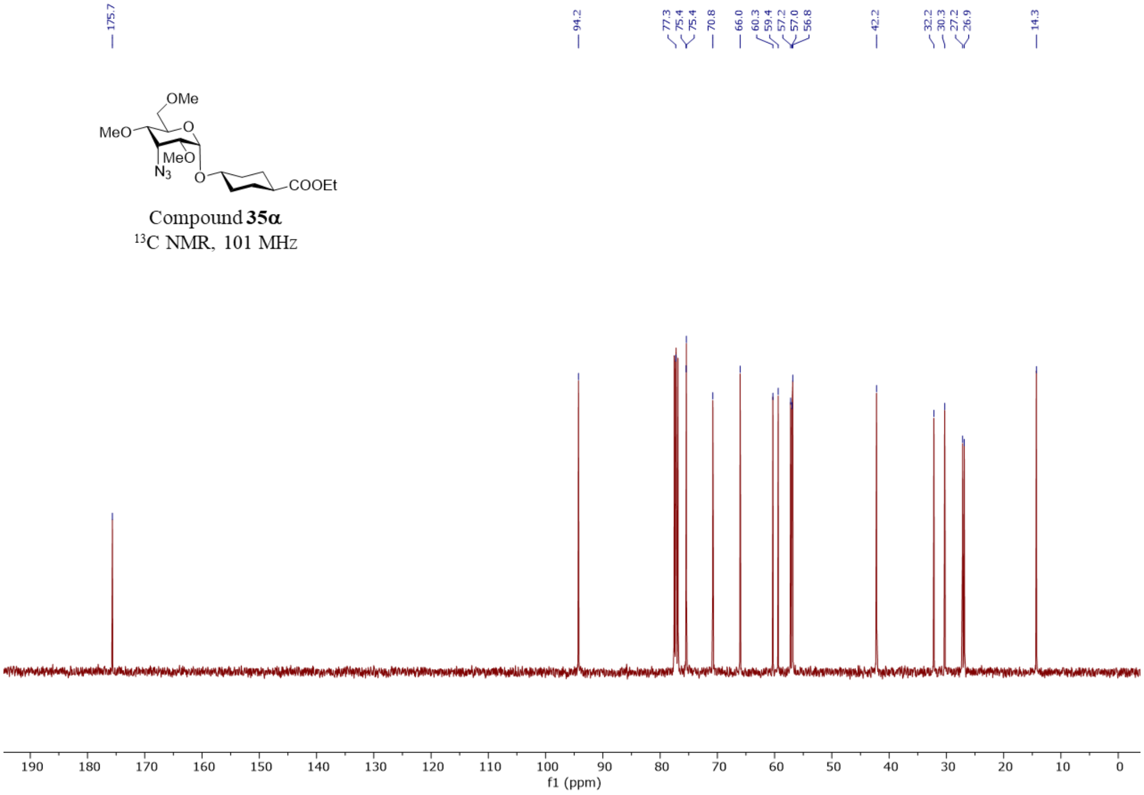

Compound **35α**
^{13}C NMR, 101 MHz

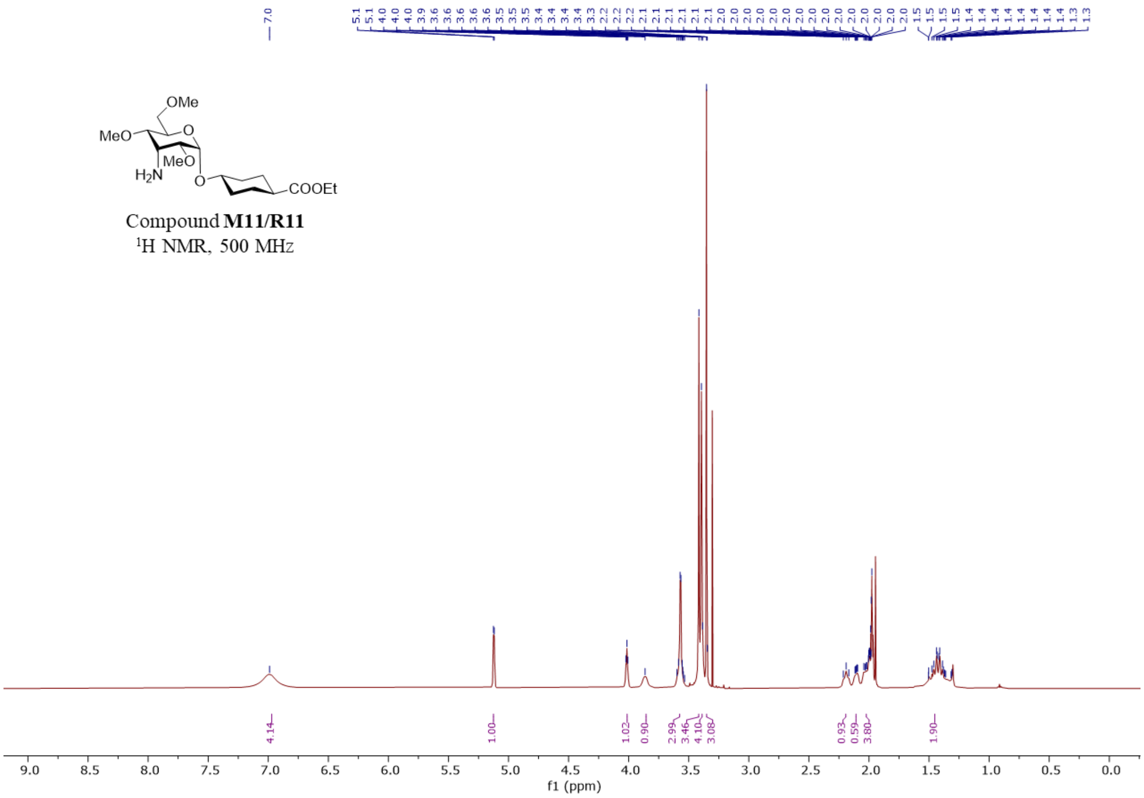
OMe
MeO
O
MeO
H_2N
O
COOEt
Compound **M11/R11**
^{1}H NMR, 500 MHz
f1 (ppm)

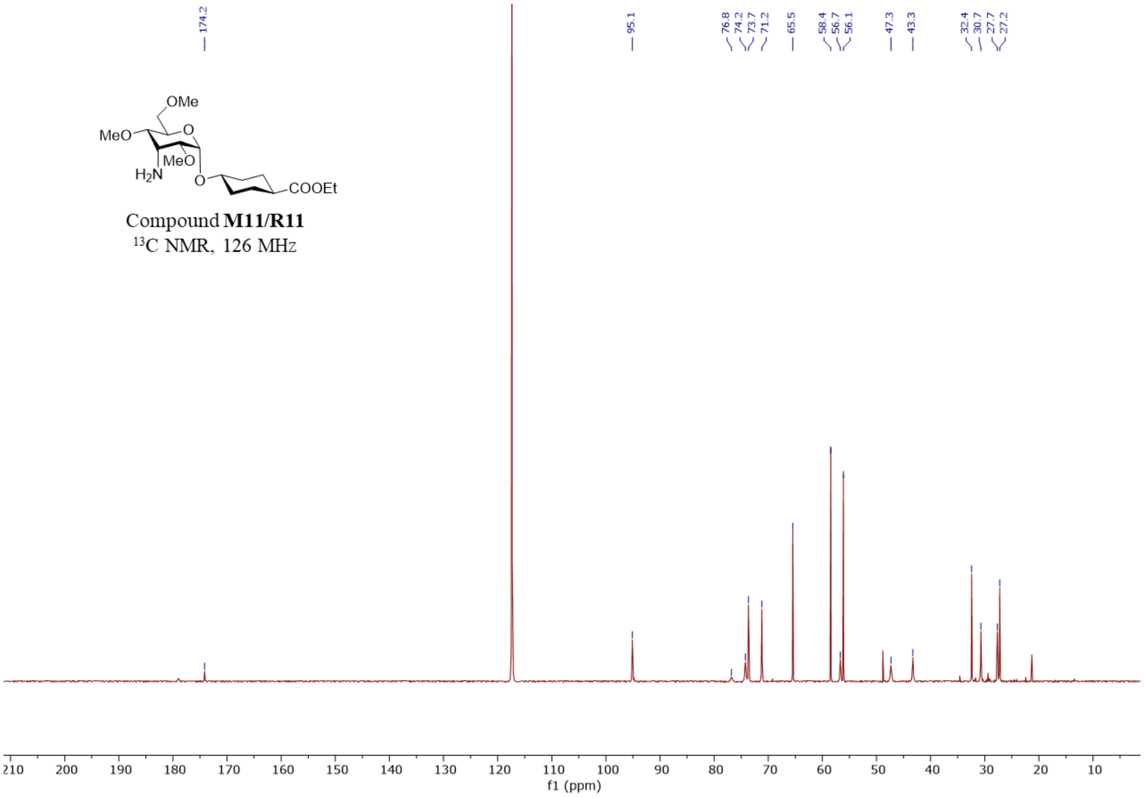
OMe
MeO
O
MeO
H_2N
O
COOEt
Compound **M11/R11**
^{13}C NMR, 126 MHz
f1 (ppm)

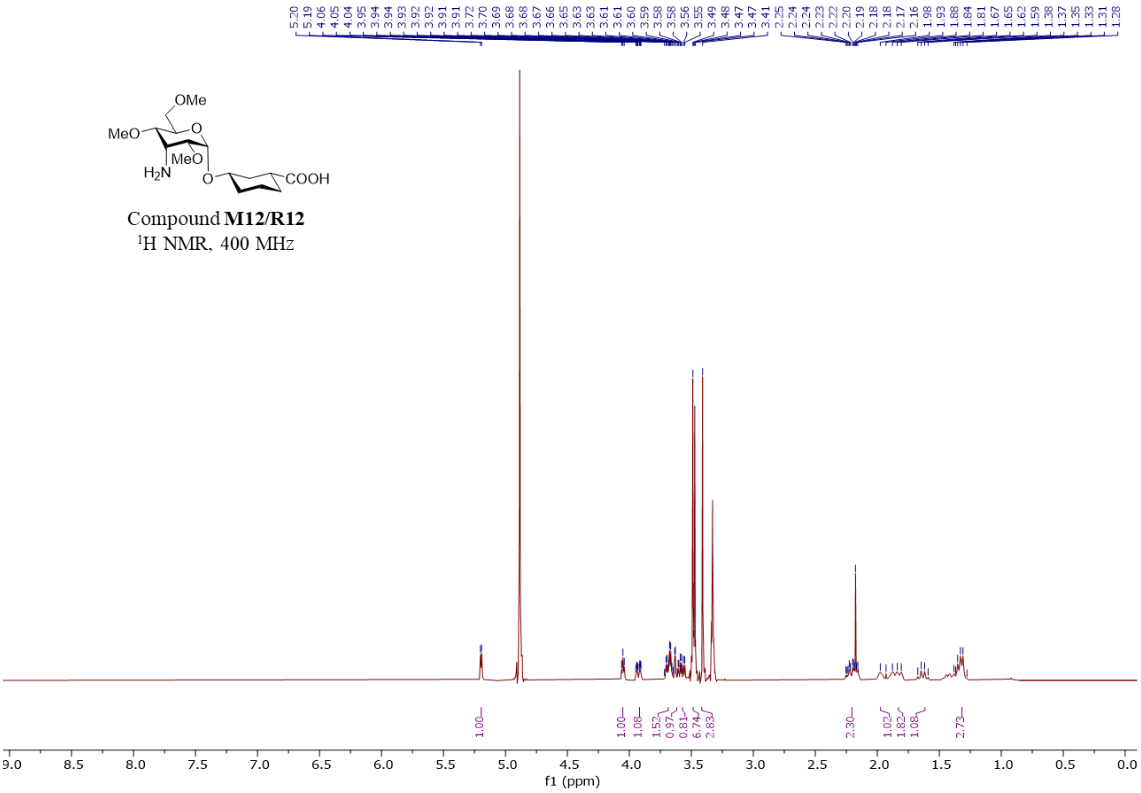
5.20 5.19 4.06 4.05 4.04 3.95 3.94 3.94 3.93 3.92 3.92 3.91 3.91 3.72 3.70 3.69 3.68 3.68 3.67 3.66 3.65 3.63 3.63 3.61 3.61 3.60 3.59 3.58 3.58 3.56 3.55 3.49 3.48 3.47 3.47 3.41 2.25 2.24 2.24 2.23 2.22 2.20 2.19 2.18 2.17 2.16 1.98 1.93 1.88 1.84 1.81 1.67 1.65 1.62 1.59 1.38 1.37 1.35 1.33 1.31 1.28
OMe
MeO
O
MeO
H_2N
O
COOH
Compound **M12/R12**
^{1}H NMR, 400 MHz
1.00 1.00 1.08 1.52 0.97 0.81 6.74 2.83 2.30 1.02 1.82 1.08 2.73
9.0 8.5 8.0 7.5 7.0 6.5 6.0 5.5 5.0 4.5 4.0 3.5 3.0 2.5 2.0 1.5 1.0 0.5 0.0
f1 (ppm)

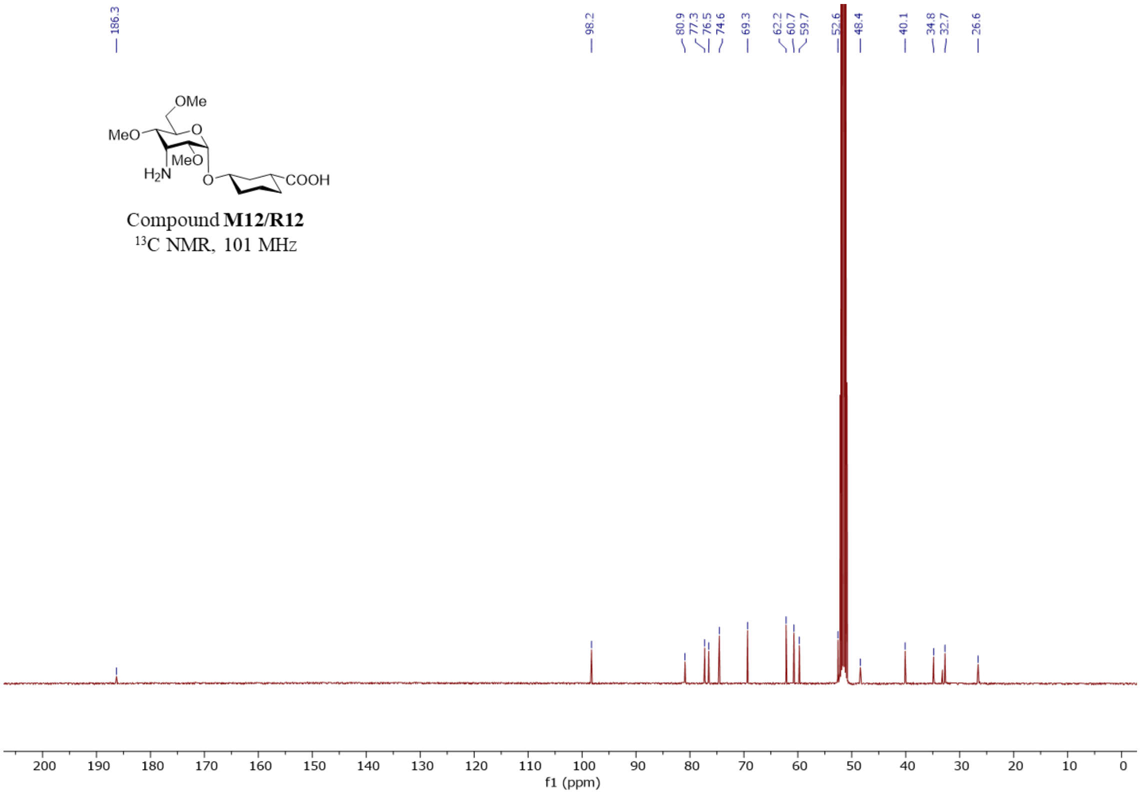
186.3 98.2 80.9 77.3 76.5 74.6 69.3 62.2 60.7 59.7 52.6 48.4 40.1 34.8 32.7 26.6
OMe
MeO
O
MeO
H_2N
O
COOH
Compound **M12/R12**
^{13}C NMR, 101 MHz
200 190 180 170 160 150 140 130 120 110 100 90 80 70 60 50 40 30 20 10 0
f1 (ppm)

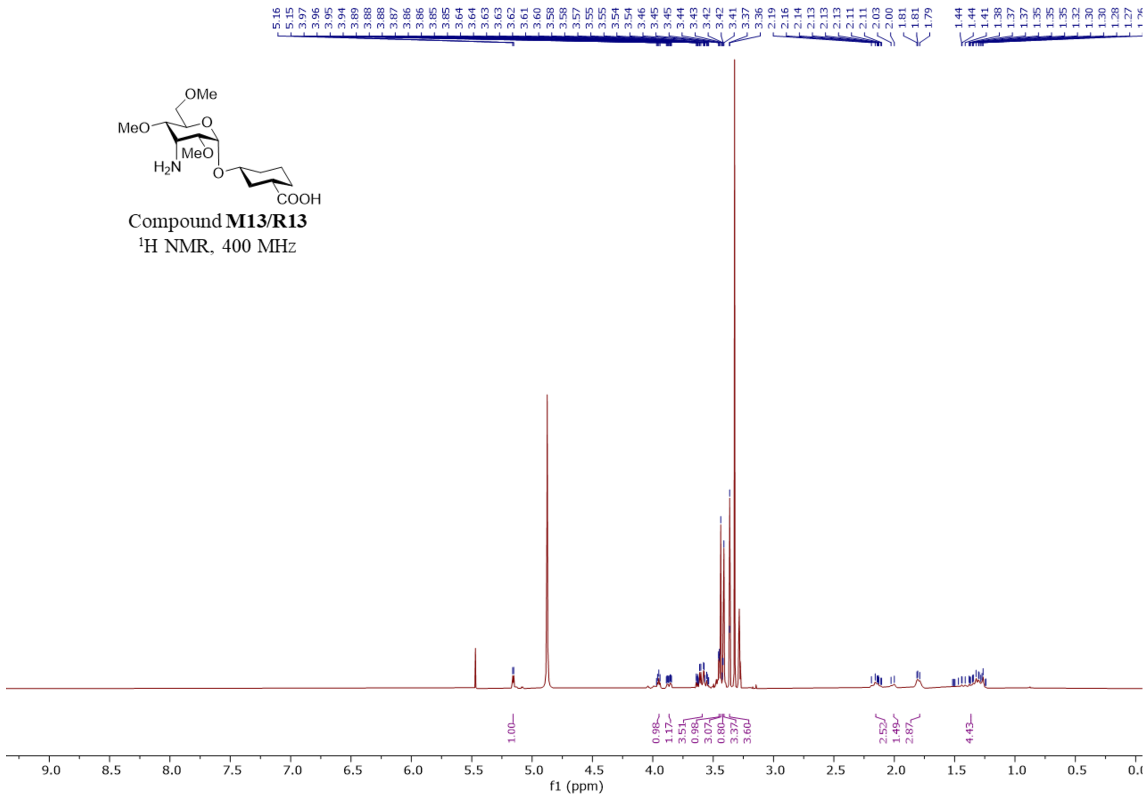
OMe
MeO
O
MeO
H2N
O
COOH
Compound M13/R13
1H NMR, 400 MHz
f1 (ppm)

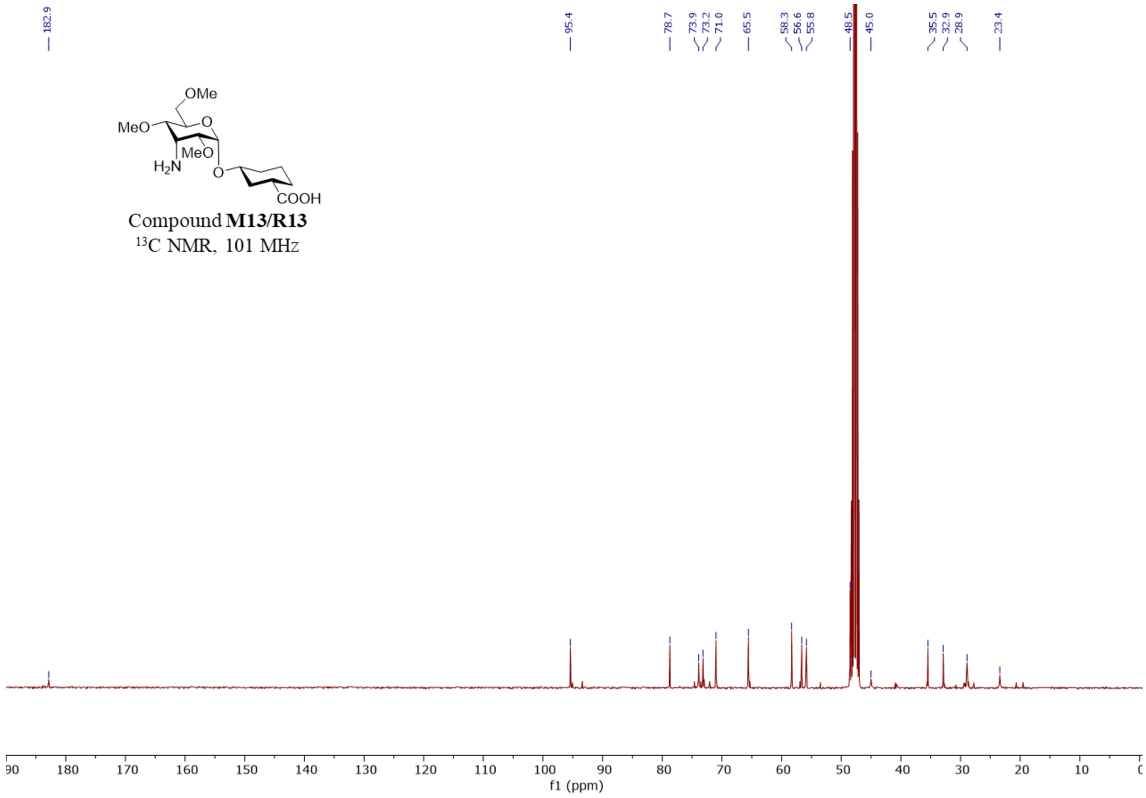
182.9
95.4
78.7
73.9
73.2
71.0
65.5
58.3
56.6
55.8
48.5
45.0
35.5
32.9
28.9
23.4
OMe
MeO
O
MeO
H2N
O
COOH
Compound M13/R13
13C NMR, 101 MHz
f1 (ppm)

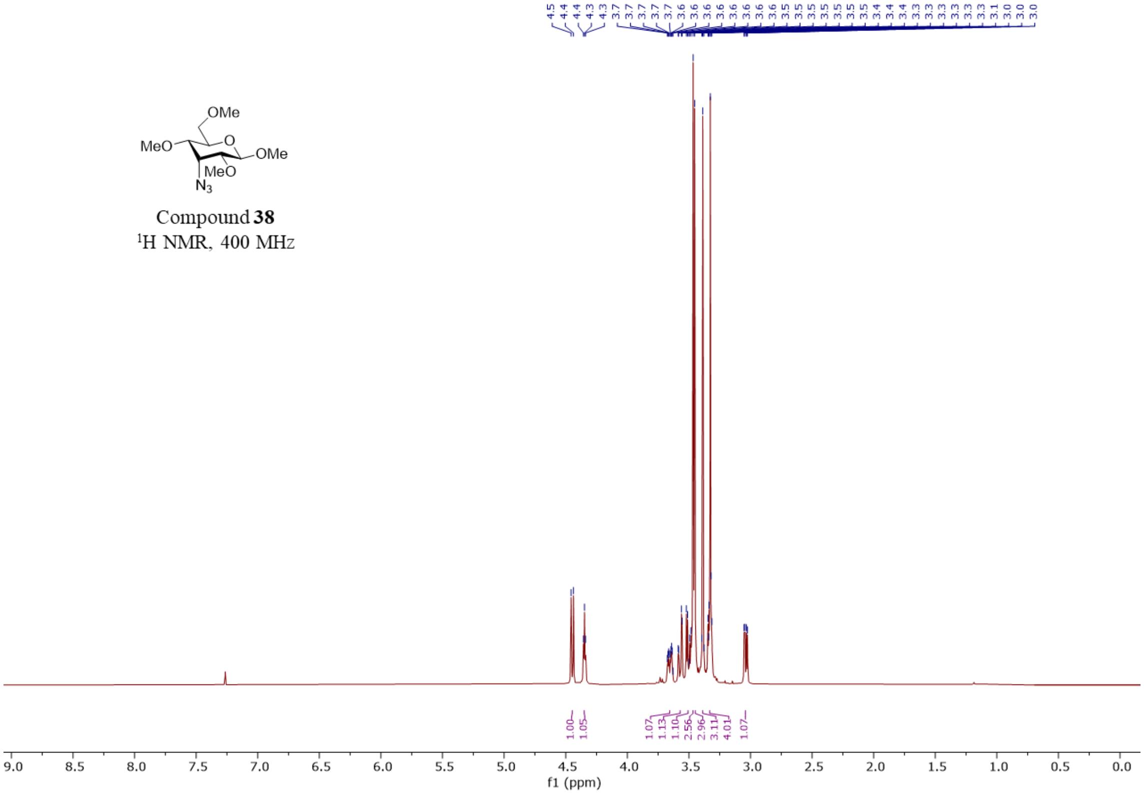
OMe
MeO
O
OMe
MeO
N3
Compound 38
1H NMR, 400 MHz
1.00
1.05
1.07
1.13
1.10
2.56
2.96
3.11
4.01
1.07
f1 (ppm)

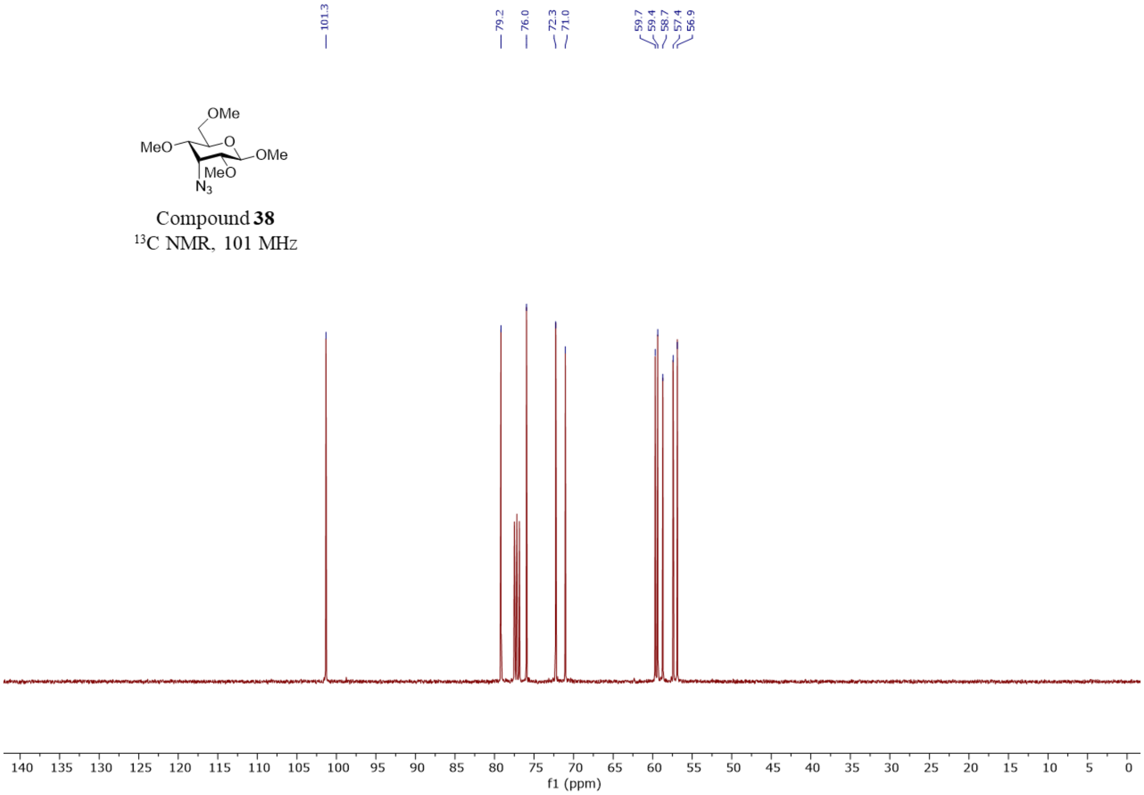
101.3
79.2
76.0
72.3
71.0
59.7
59.4
58.7
57.4
56.9
OMe
MeO
O
OMe
MeO
N3
Compound 38
13C NMR, 101 MHz
f1 (ppm)

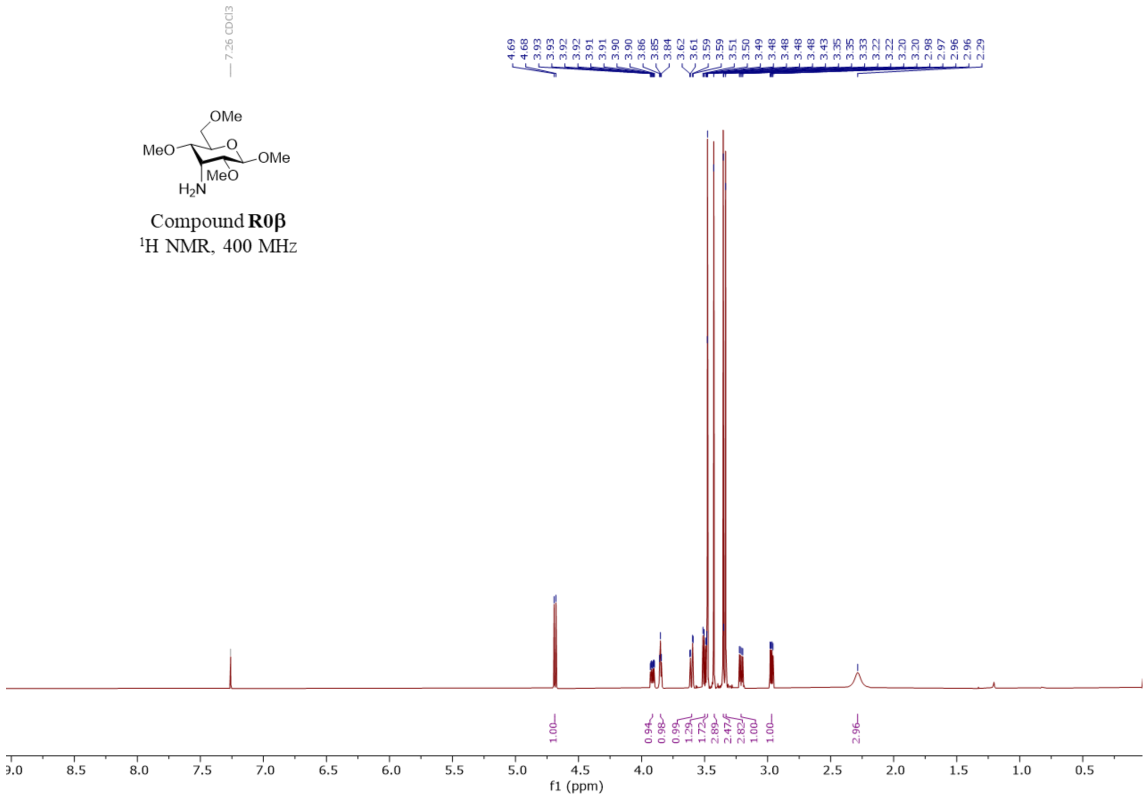
7.26 cdcl3
4.69
4.68
3.93
3.93
3.92
3.92
3.91
3.91
3.90
3.90
3.86
3.85
3.84
3.62
3.61
3.59
3.59
3.51
3.50
3.49
3.48
3.48
3.48
3.48
3.43
3.35
3.35
3.33
3.22
3.22
3.20
3.20
2.98
2.97
2.96
2.96
2.29
OMe
MeO
O
OMe
MeO
H2N
Compound R0β
1H NMR, 400 MHz
1.00
0.94
0.98
0.99
1.29
1.72
2.89
2.47
2.82
1.00
1.00
2.96
f1 (ppm)

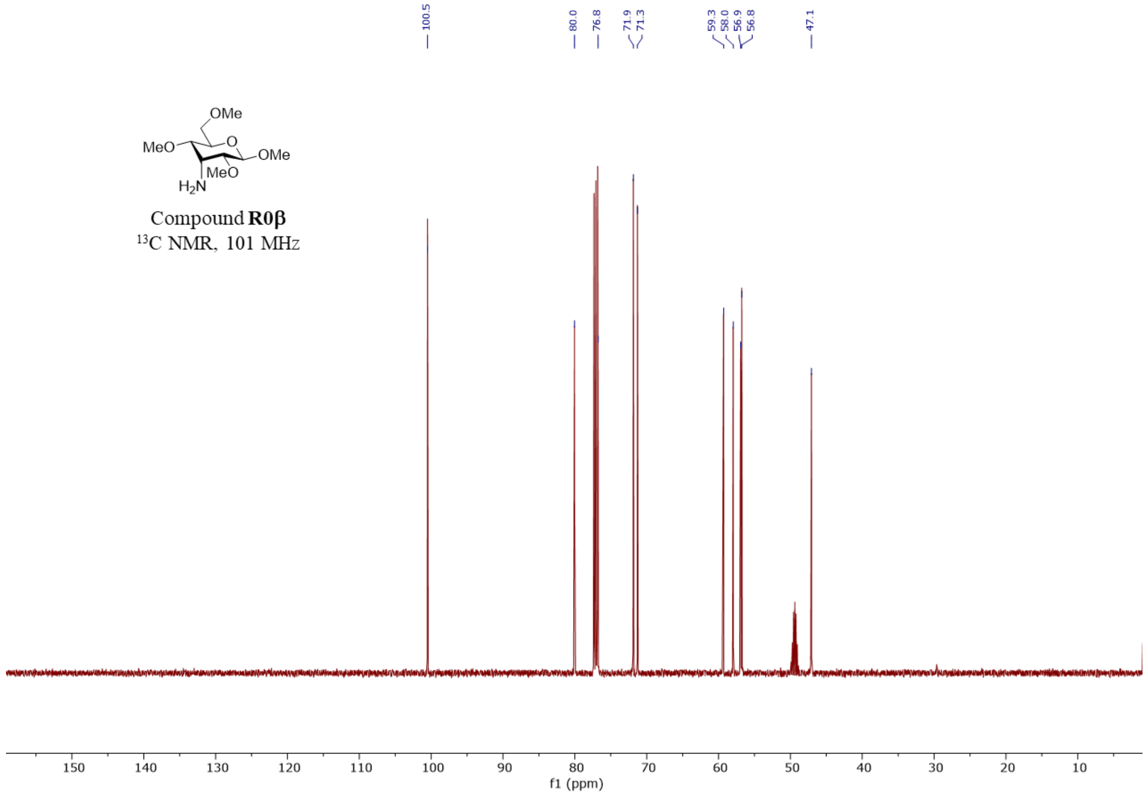
100.5
80.0
76.8
71.9
71.3
59.3
58.0
56.9
56.8
47.1
OMe
MeO
O
OMe
MeO
H2N
Compound R0β
13C NMR, 101 MHz
f1 (ppm)

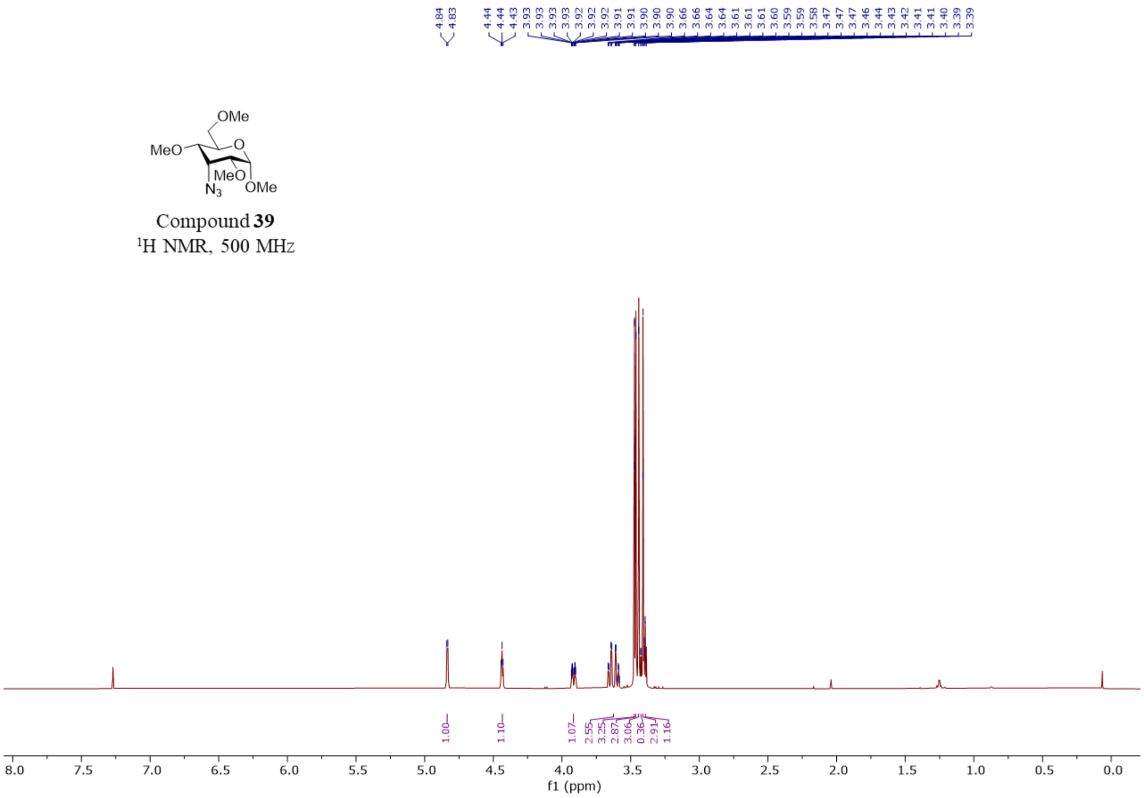
4.84
4.83
4.44
4.44
4.43
3.93
3.93
3.93
3.93
3.92
3.92
3.91
3.91
3.90
3.90
3.90
3.66
3.66
3.64
3.64
3.61
3.61
3.60
3.59
3.59
3.58
3.47
3.47
3.46
3.44
3.43
3.42
3.41
3.40
3.39
3.39
OMe
MeO
O
MeO
N_3
OMe
Compound **39**
^{1}H NMR, 500 MHz
1.00
1.10
1.07
2.55
3.25
2.87
3.06
0.36
2.91
1.16
8.0 7.5 7.0 6.5 6.0 5.5 5.0 4.5 4.0 3.5 3.0 2.5 2.0 1.5 1.0 0.5 0.0
f1 (ppm)

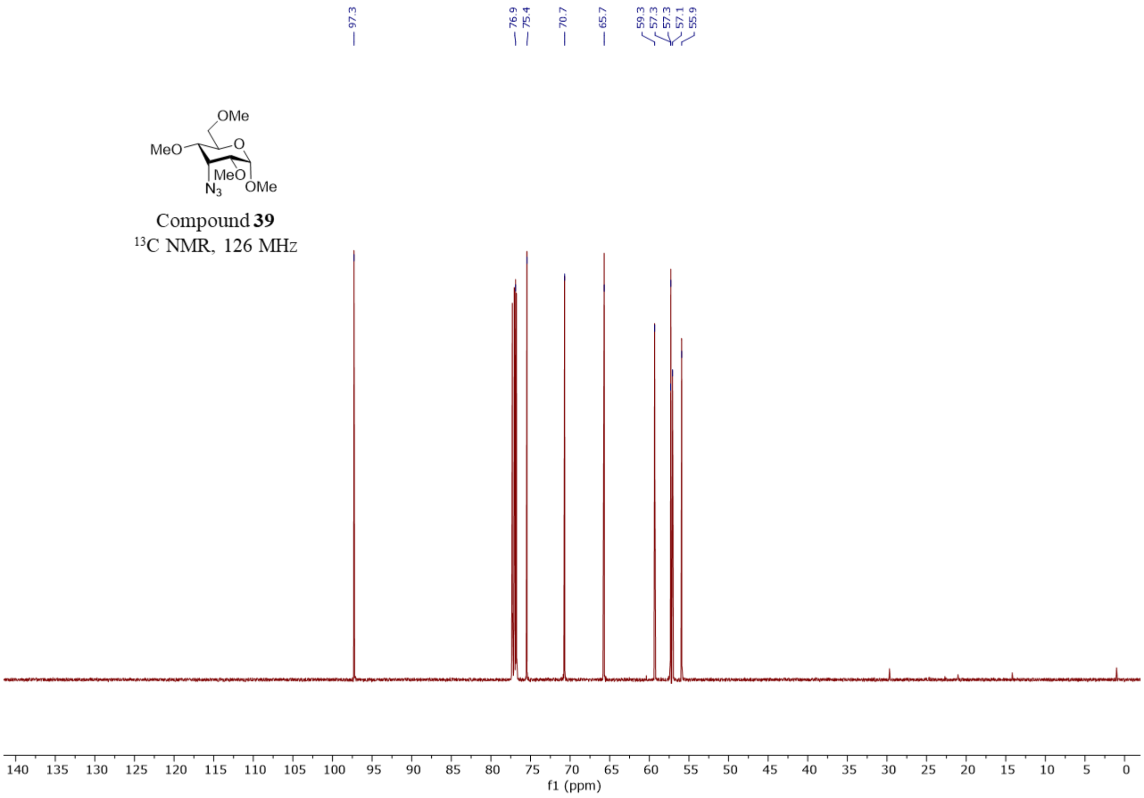
97.3
76.9
75.4
70.7
65.7
59.3
57.3
57.3
57.1
55.9
OMe
MeO
O
MeO
N_3
OMe
Compound **39**
^{13}C NMR, 126 MHz
140 135 130 125 120 115 110 105 100 95 90 85 80 75 70 65 60 55 50 45 40 35 30 25 20 15 10 5 0
f1 (ppm)

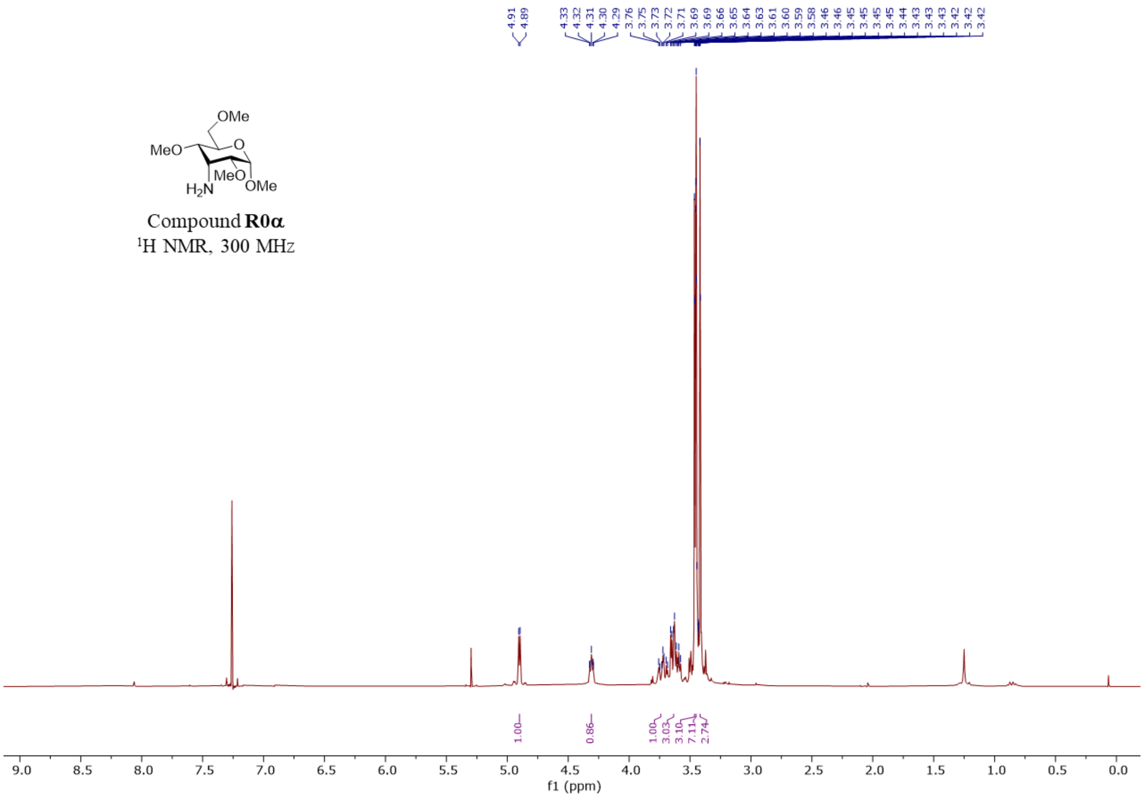

Compound R0α
¹H NMR, 300 MHz
f1 (ppm)

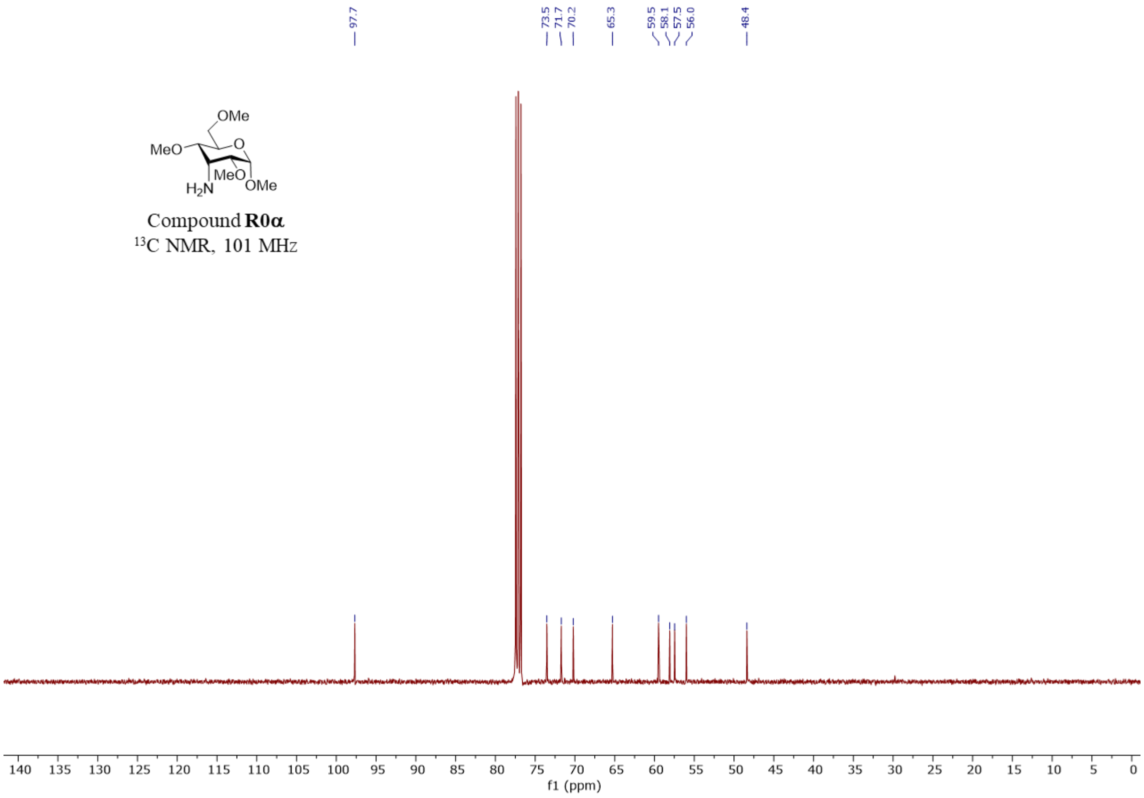

Compound R0α
¹³C NMR, 101 MHz
97.7
73.5
71.7
70.2
65.3
59.5
58.1
57.5
56.0
48.4
f1 (ppm)

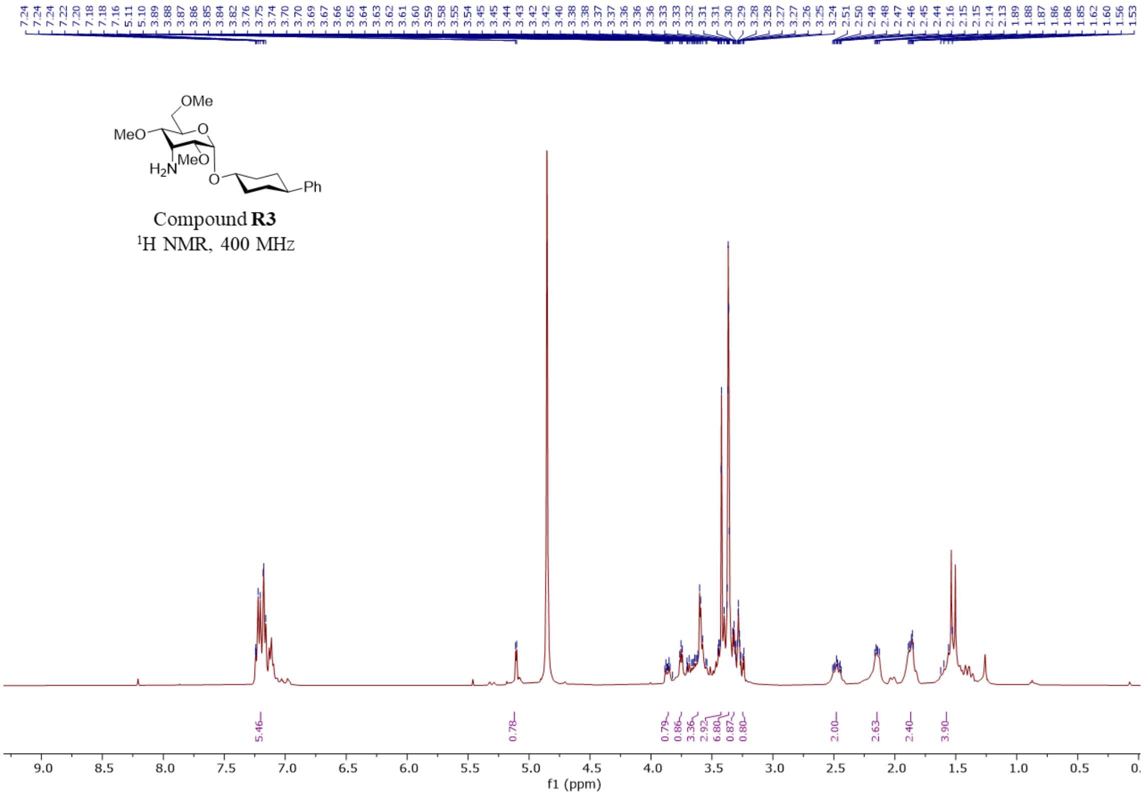
OMe
MeO
O
H_2N
MeO
O
Ph
Compound **R3**
^{1}H NMR, 400 MHz
f1 (ppm)

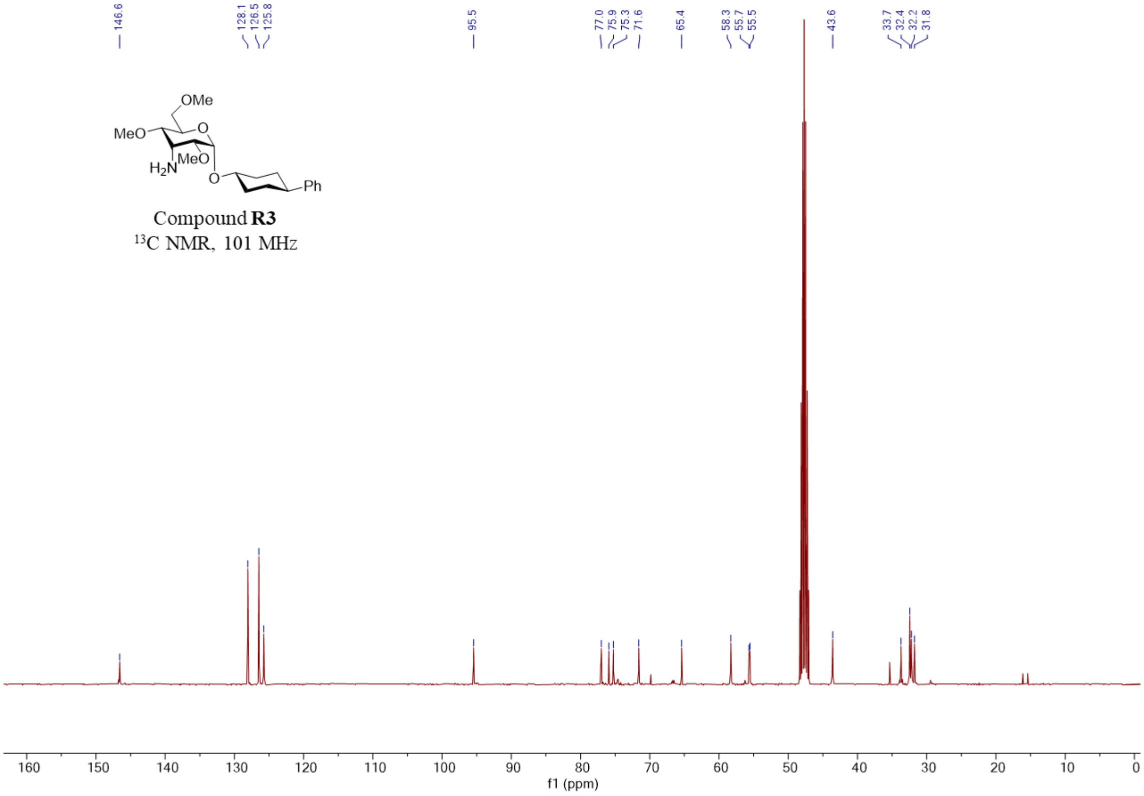
146.6
128.1
126.5
125.8
95.5
77.0
75.9
75.3
71.6
65.4
58.3
55.7
55.5
43.6
33.7
32.4
32.2
31.8
OMe
MeO
O
H_2N
MeO
O
Ph
Compound **R3**
^{13}C NMR, 101 MHz
f1 (ppm)

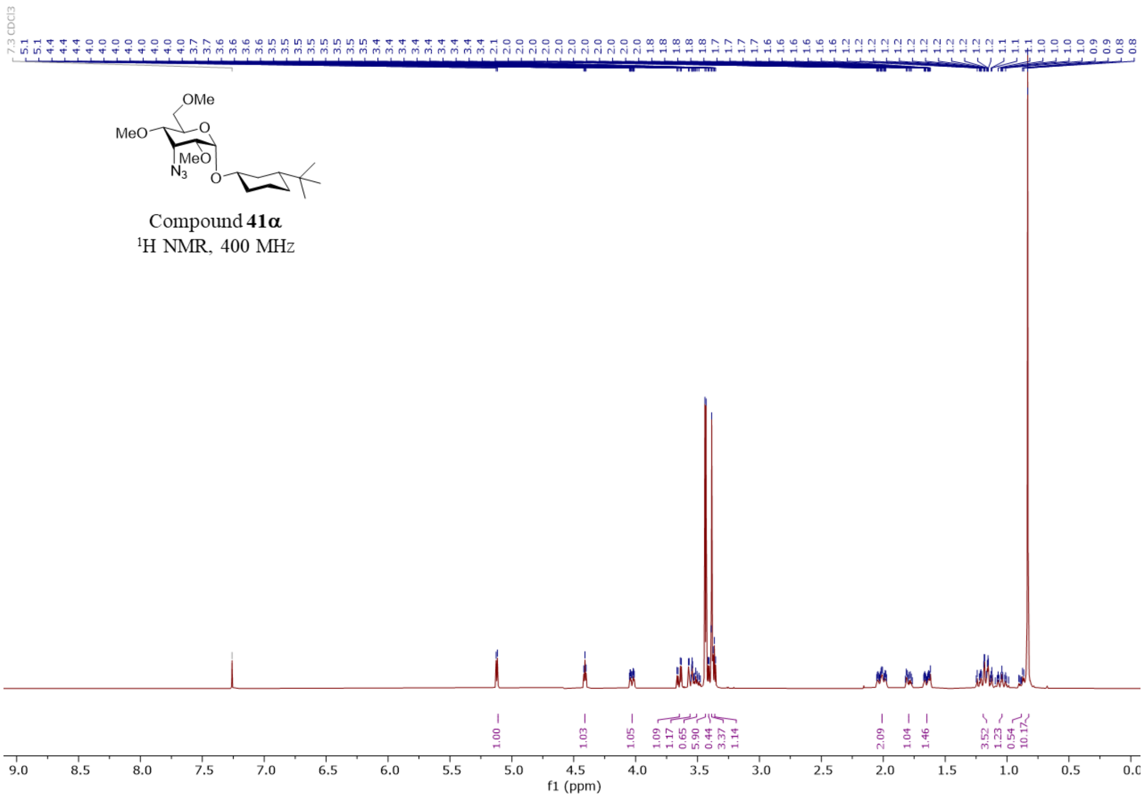

Compound **41α**
^{1}H NMR, 400 MHz

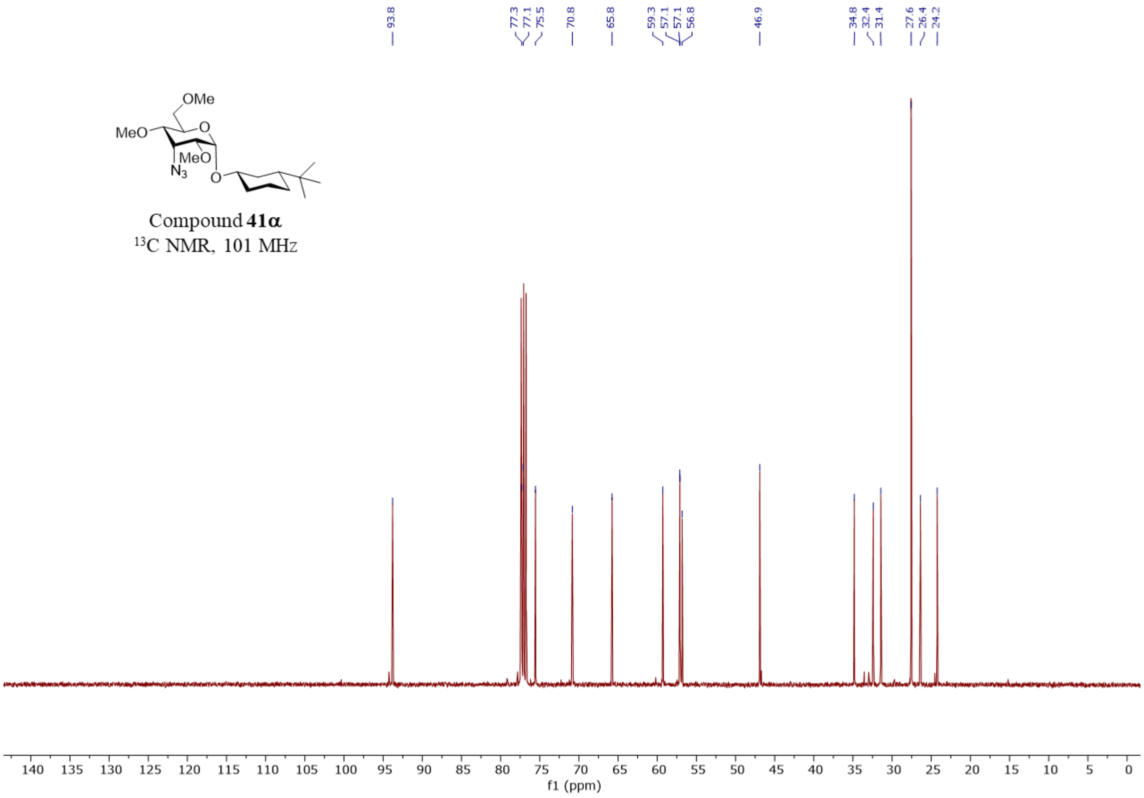

Compound **41α**
^{13}C NMR, 101 MHz

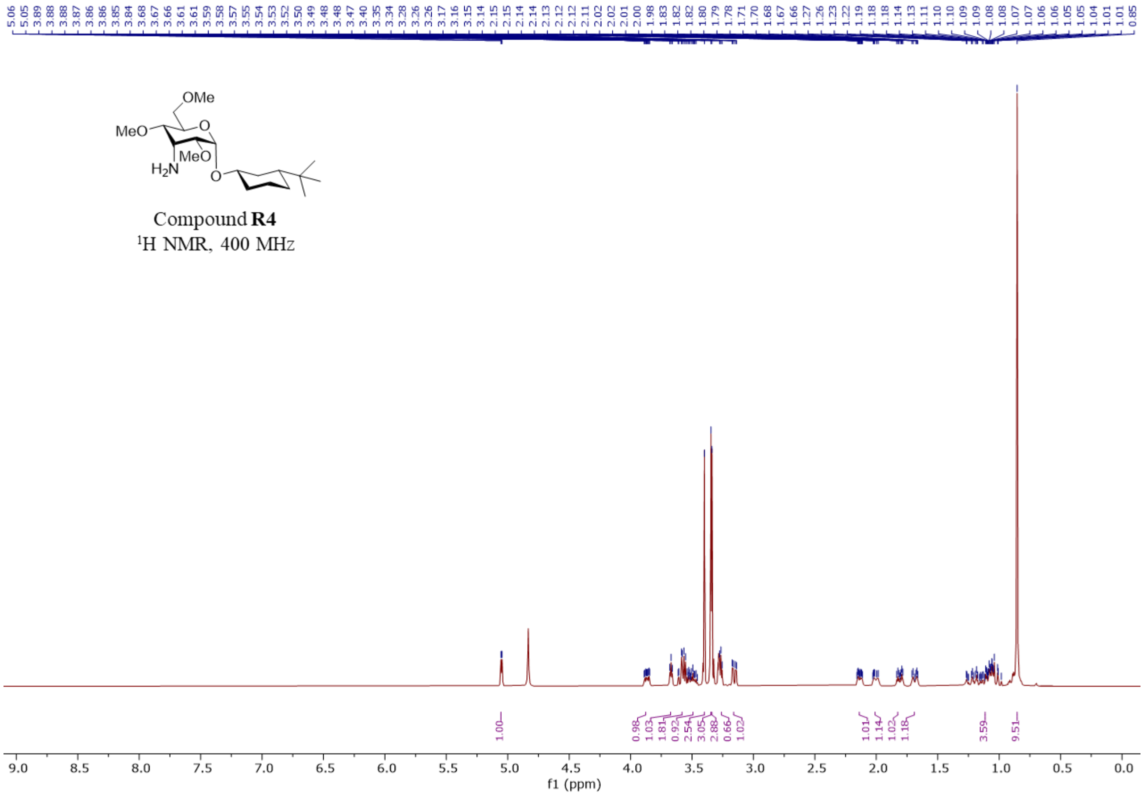
5.06 5.05 3.89 3.88 3.87 3.86 3.85 3.84 3.68 3.67 3.66 3.61 3.61 3.59 3.58 3.57 3.55 3.54 3.53 3.52 3.50 3.49 3.48 3.47 3.40 3.35 3.34 3.28 3.26 3.26 3.17 3.16 3.15 3.14 2.15 2.15 2.14 2.14 2.13 2.12 2.12 2.11 2.02 2.02 2.01 2.00 1.98 1.83 1.82 1.82 1.80 1.79 1.78 1.71 1.70 1.68 1.67 1.66 1.27 1.26 1.23 1.22 1.19 1.18 1.18 1.14 1.13 1.11 1.10 1.10 1.09 1.09 1.08 1.08 1.07 1.06 1.06 1.05 1.05 1.04 1.01 1.01 0.85
OMe
MeO
O
MeO
H_2N
O
Compound **R4**
^{1}H NMR, 400 MHz
1.00 0.98 1.03 1.81 0.92 2.54 3.05 2.88 0.66 1.02 1.01 1.14 1.02 1.18 3.59 9.51
9.0 8.5 8.0 7.5 7.0 6.5 6.0 5.5 5.0 4.5 4.0 3.5 3.0 2.5 2.0 1.5 1.0 0.5 0.0
f1 (ppm)

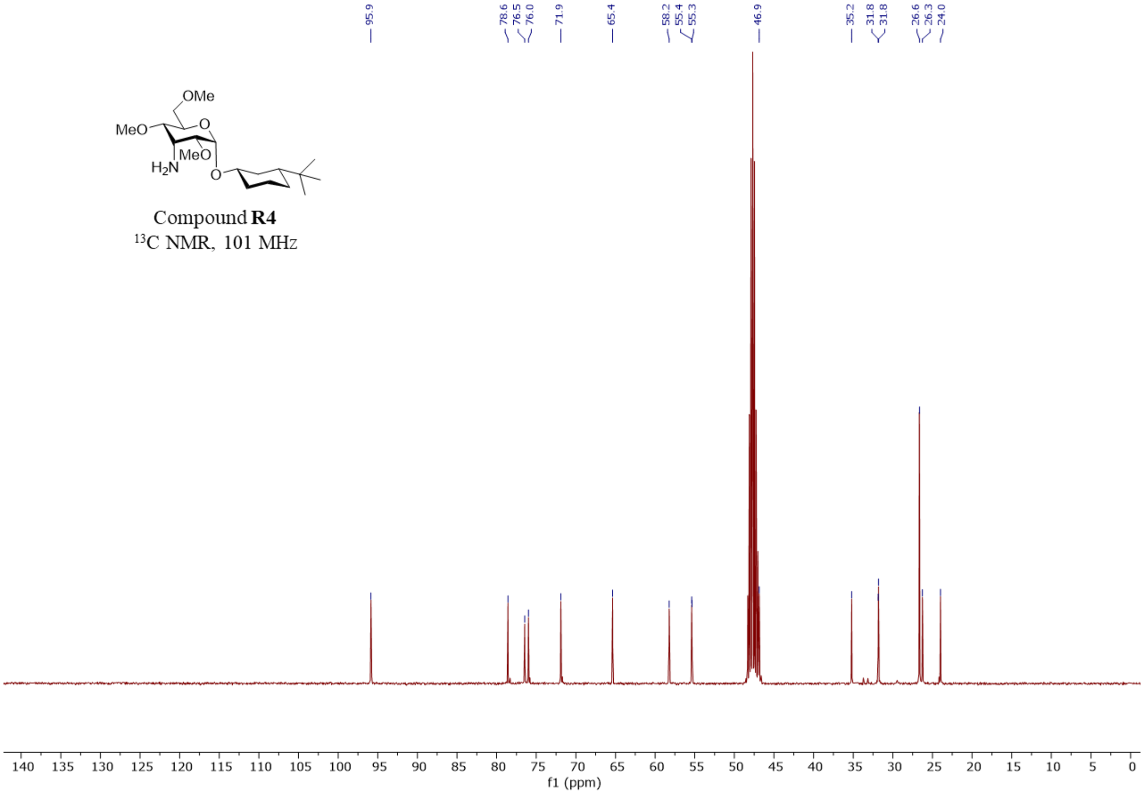
95.9 78.6 76.5 76.0 71.9 65.4 58.2 55.4 55.3 46.9 35.2 31.8 31.8 26.6 26.3 24.0
OMe
MeO
O
MeO
H_2N
O
Compound **R4**
^{13}C NMR, 101 MHz
140 135 130 125 120 115 110 105 100 95 90 85 80 75 70 65 60 55 50 45 40 35 30 25 20 15 10 5 0
f1 (ppm)

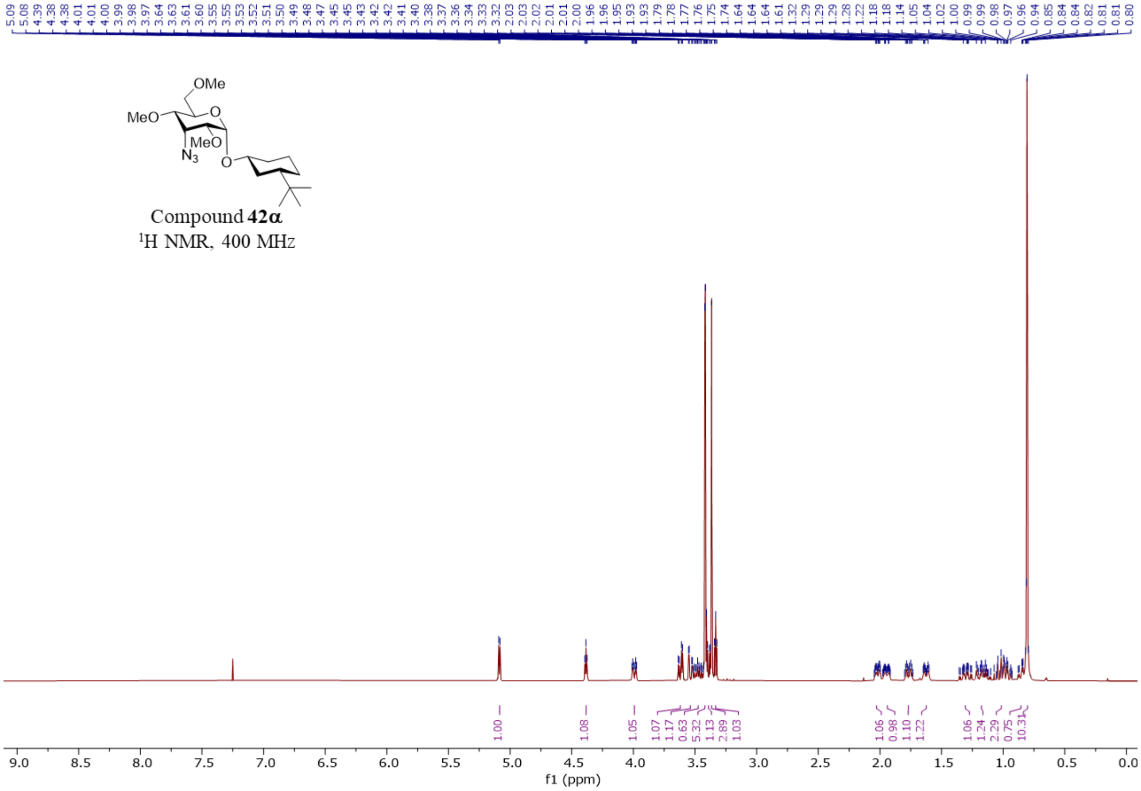
OMe
MeO
O
MeO
N_3
O
Compound **42α**
^{1}H NMR, 400 MHz
f1 (ppm)

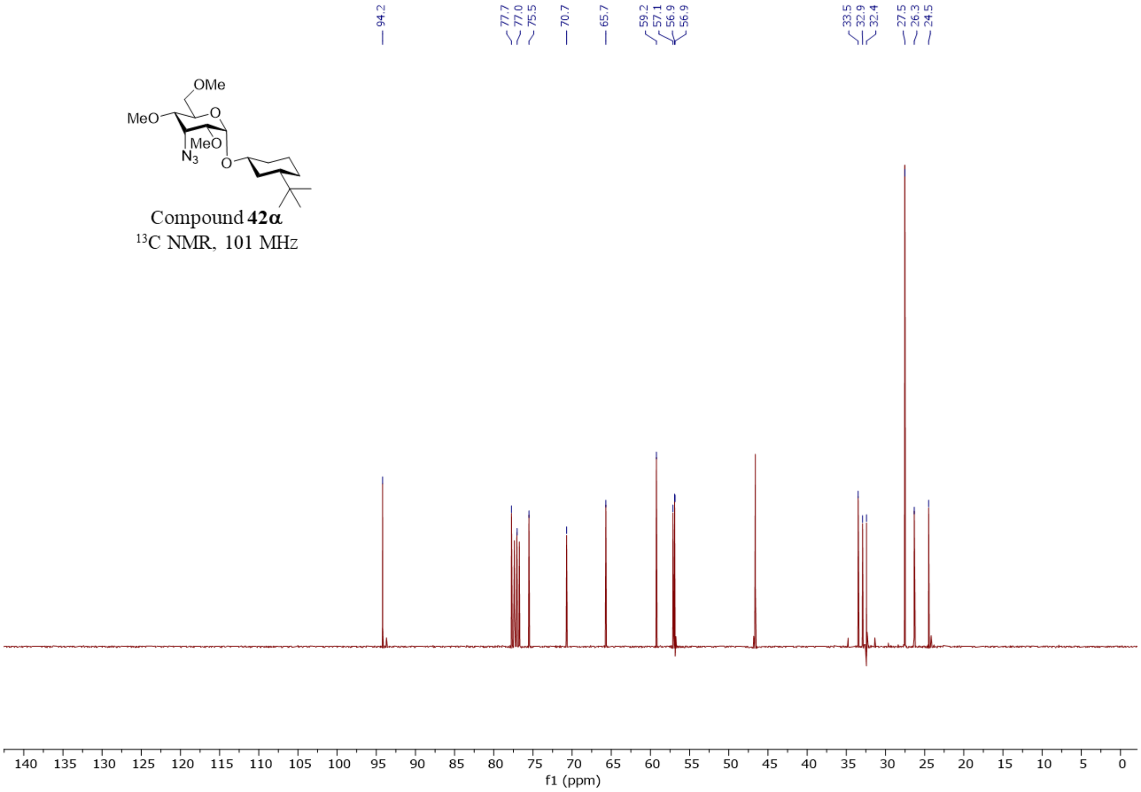
94.2
77.7
77.0
75.5
70.7
65.7
59.2
57.1
56.9
56.9
33.5
32.9
32.4
27.5
26.3
24.5
OMe
MeO
O
MeO
N_3
O
Compound **42α**
^{13}C NMR, 101 MHz
f1 (ppm)

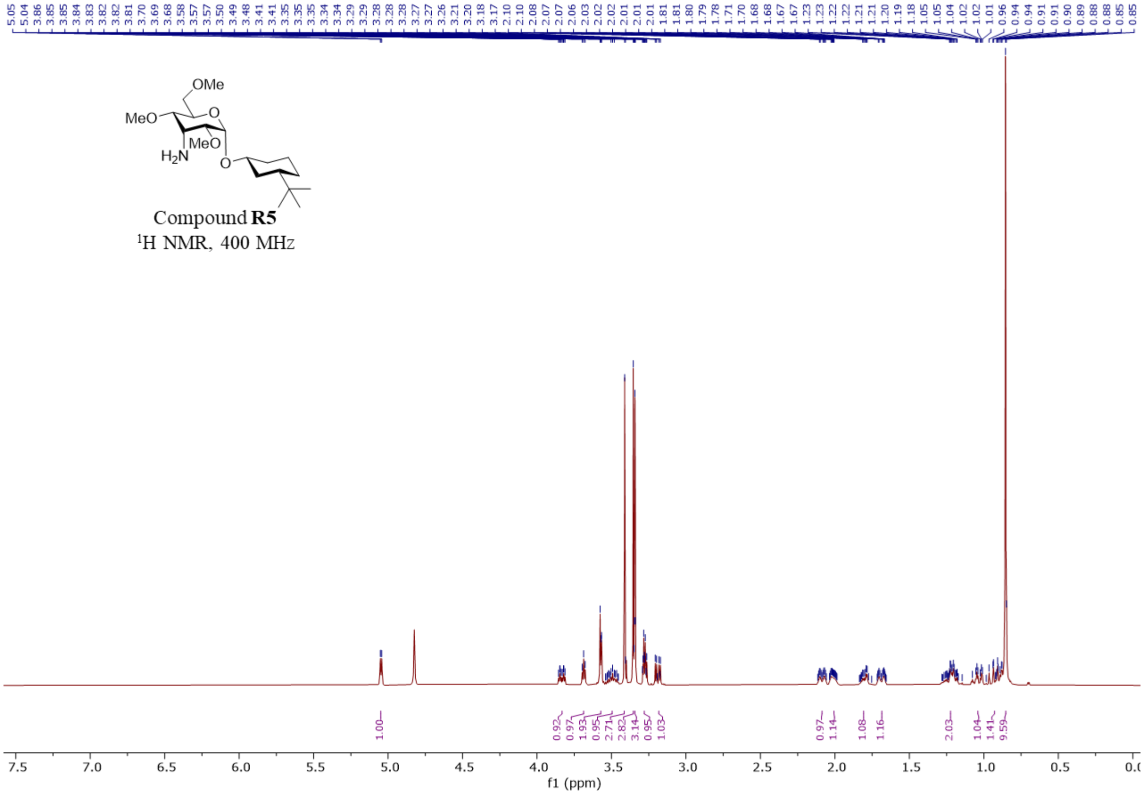

Compound R5
1H NMR, 400 MHz
f1 (ppm)

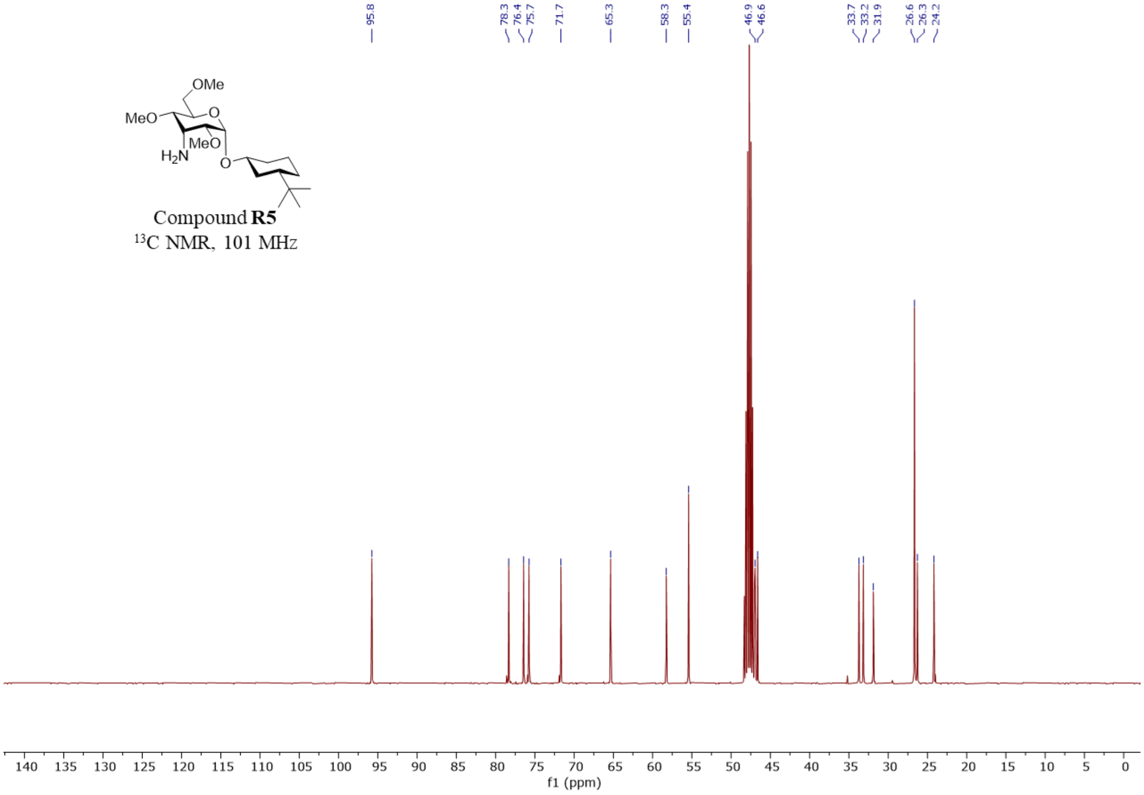

Compound R5
13C NMR, 101 MHz
95.8
78.3
76.4
75.7
71.7
65.3
58.3
55.4
46.9
46.6
33.7
33.2
31.9
26.6
26.3
24.2
f1 (ppm)

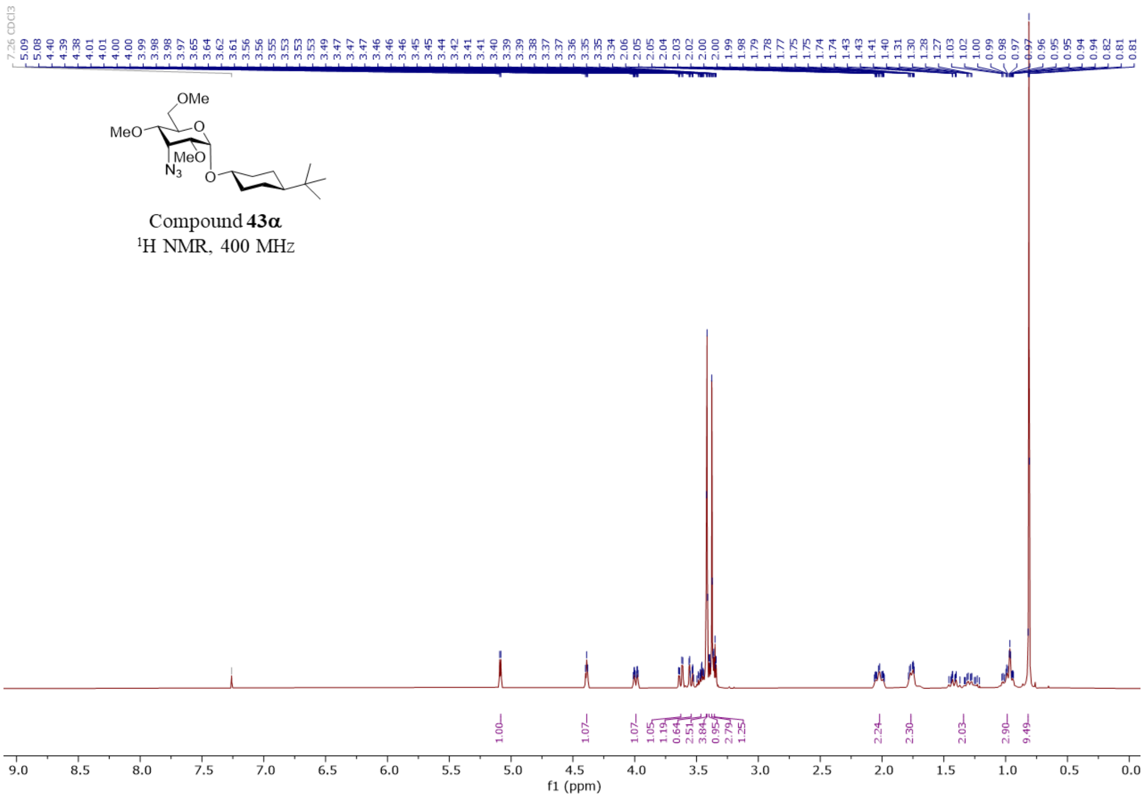
OMe
MeO
O
MeO
N_3
O
Compound **43α**
^{1}H NMR, 400 MHz
f1 (ppm)

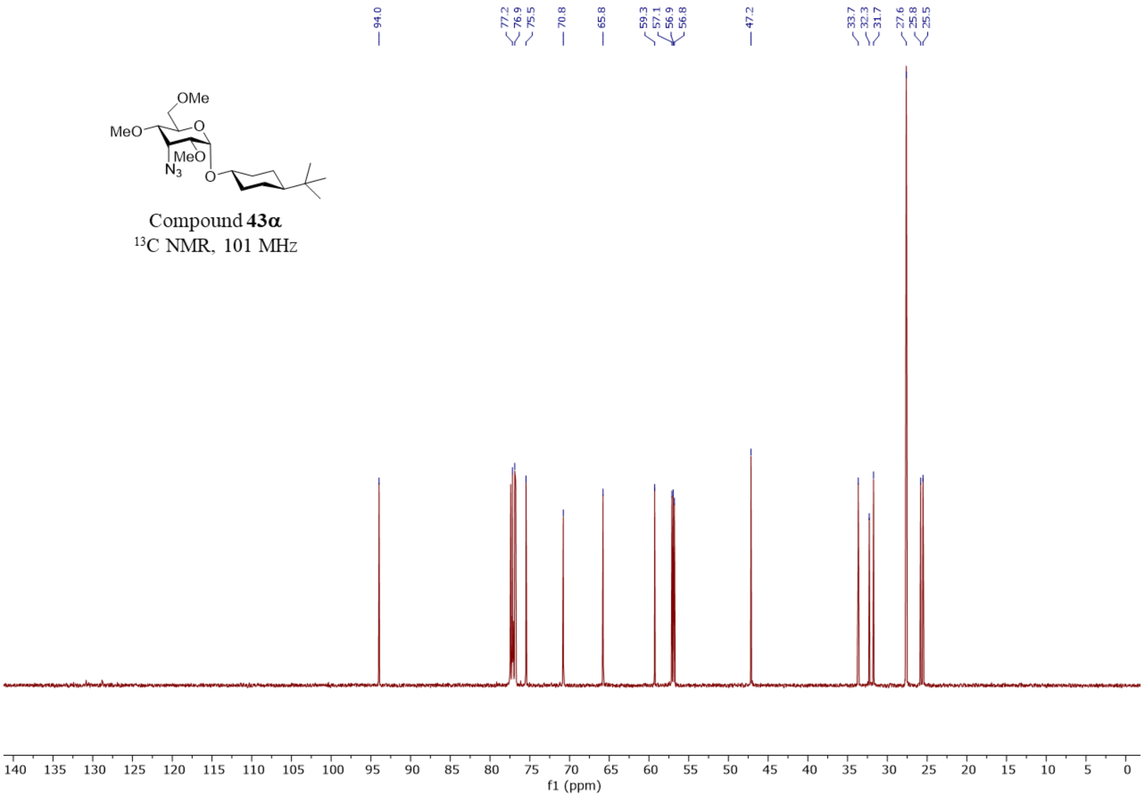
94.0
77.2
76.9
75.5
70.8
65.8
59.3
57.1
56.9
56.8
47.2
33.7
32.3
31.7
27.6
25.8
25.5
OMe
MeO
O
MeO
N_3
O
Compound **43α**
^{13}C NMR, 101 MHz
f1 (ppm)

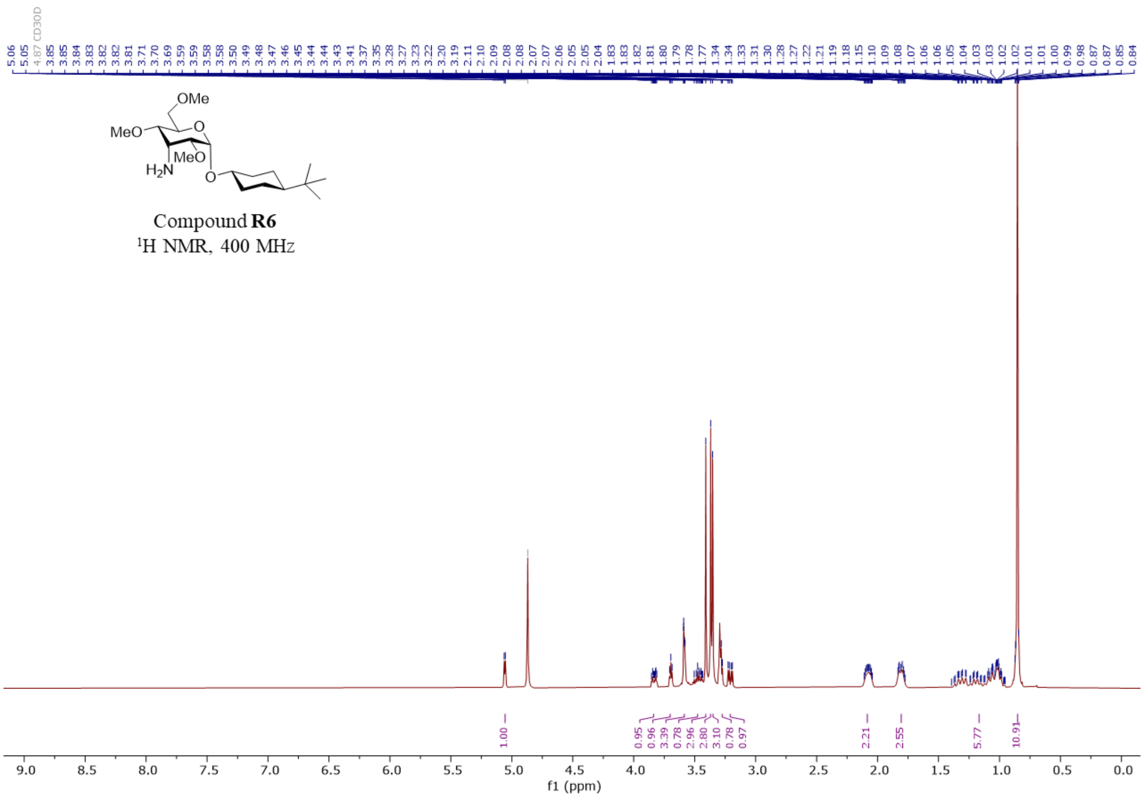
Compound R6
^{1}H NMR, 400 MHz
f1 (ppm)

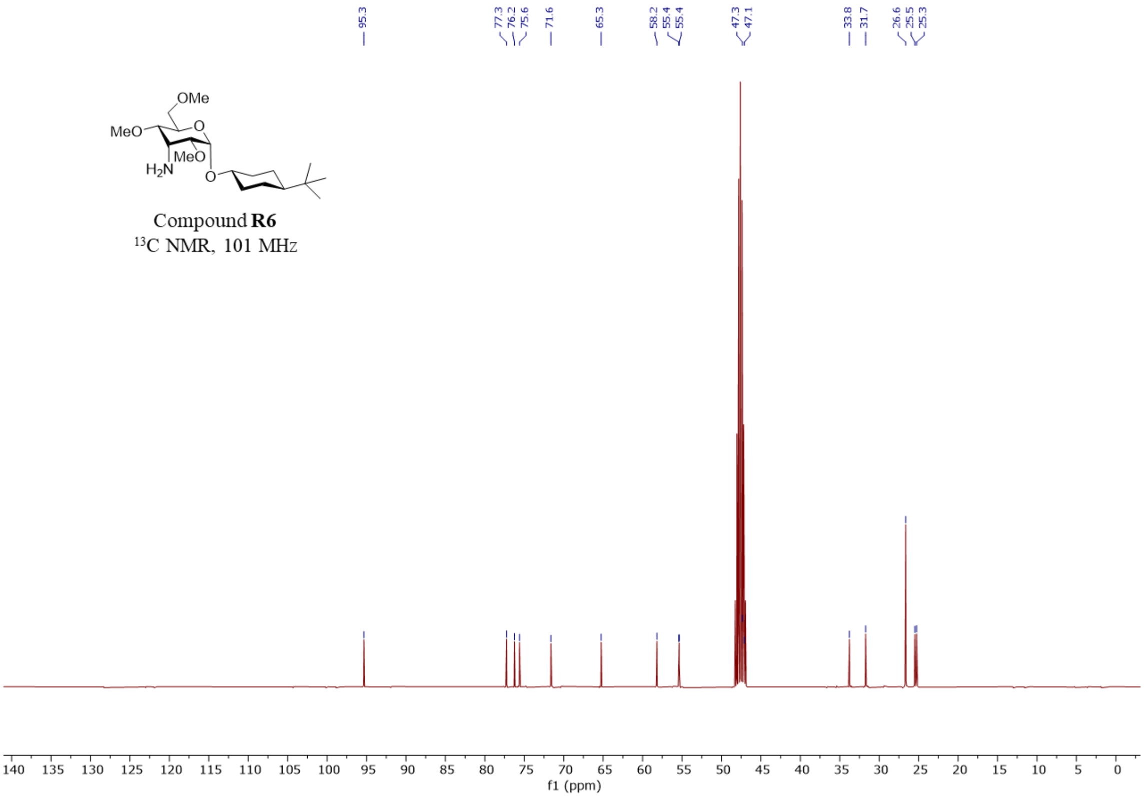
Compound R6
^{13}C NMR, 101 MHz
f1 (ppm)

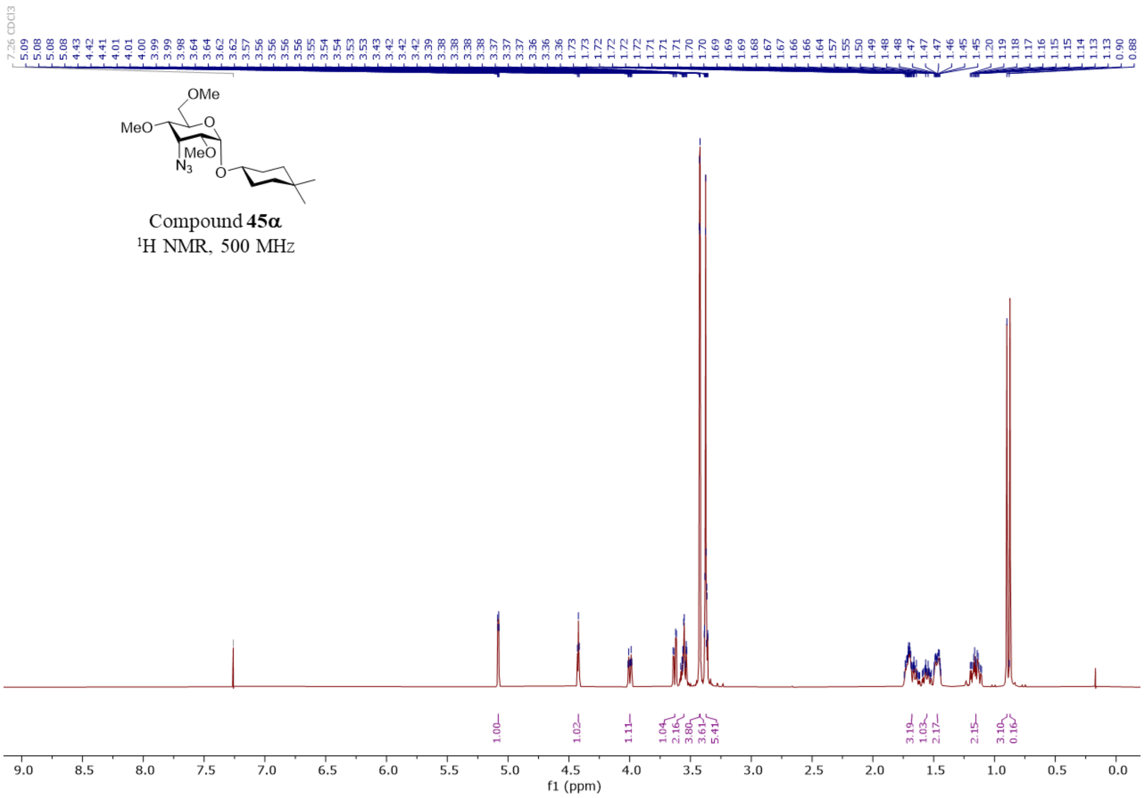

OMe
MeO
O
MeO
N3
O
Compound 45α
1H NMR, 500 MHz
f1 (ppm)

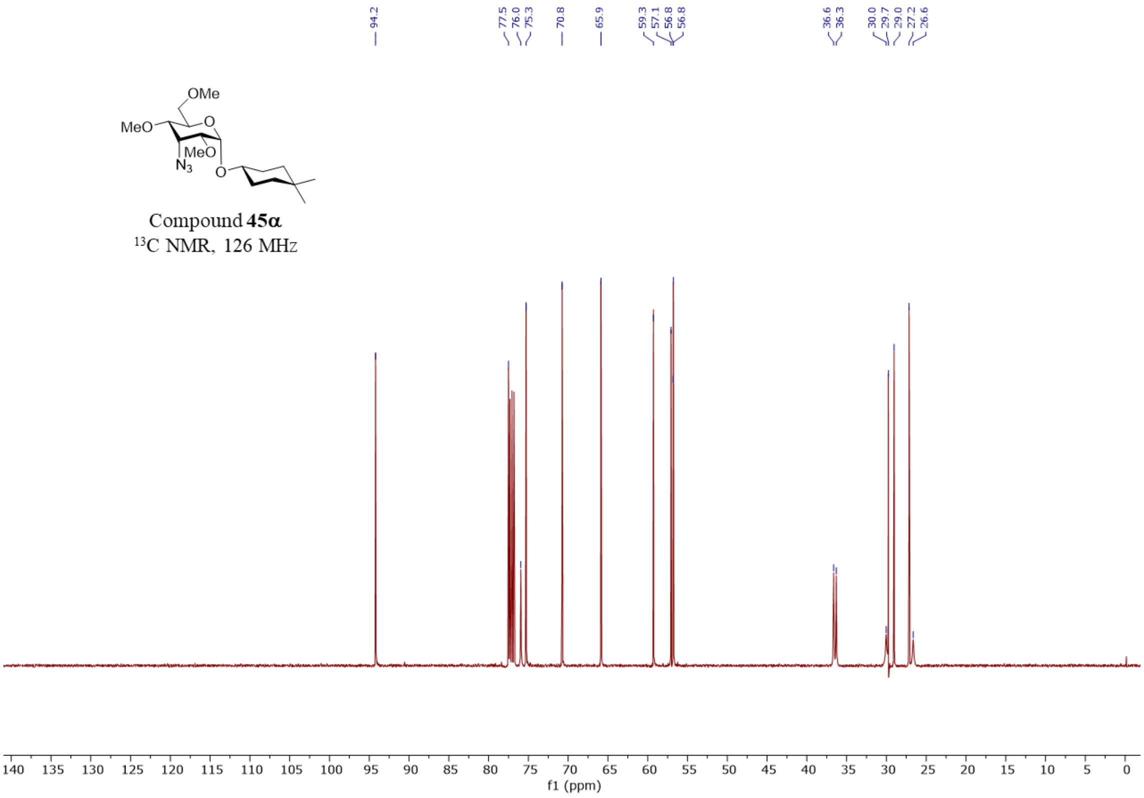

94.2
77.5
76.0
75.3
70.8
65.9
59.3
57.1
56.8
56.8
36.6
36.3
30.0
29.7
29.0
27.2
26.6
OMe
MeO
O
MeO
N3
O
Compound 45α
13C NMR, 126 MHz
f1 (ppm)

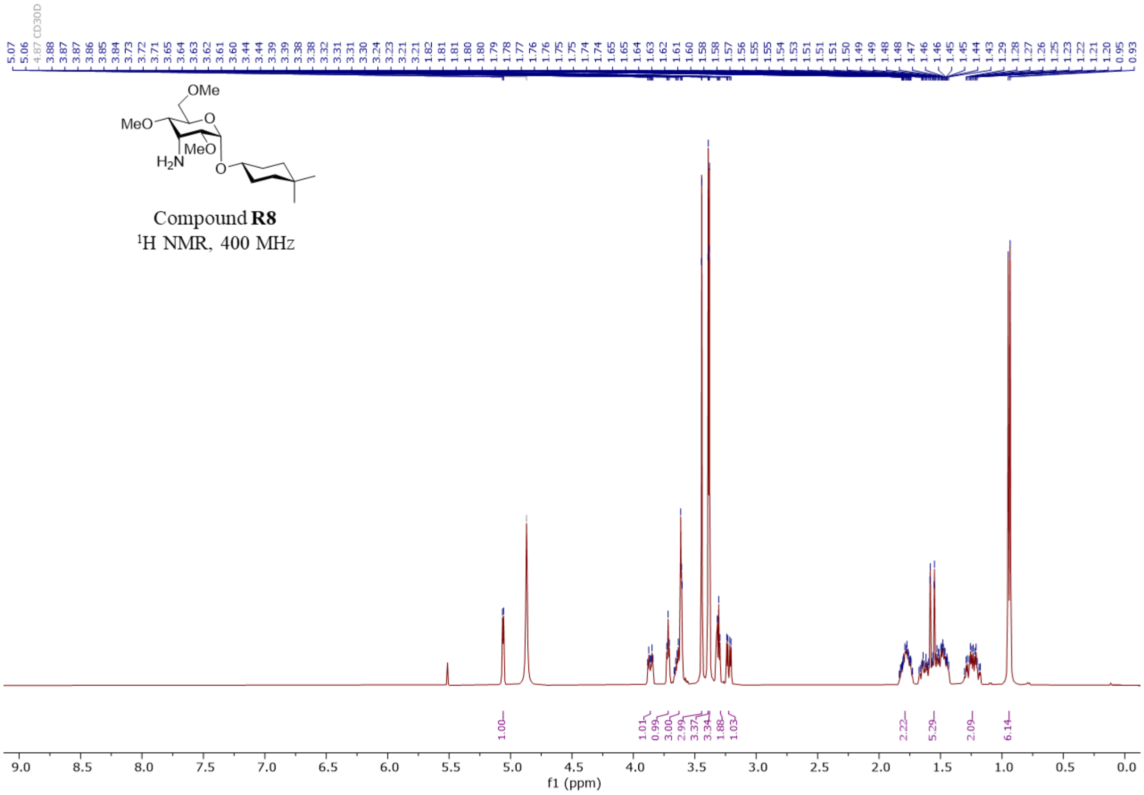

Compound **R8**
^{1}H NMR, 400 MHz

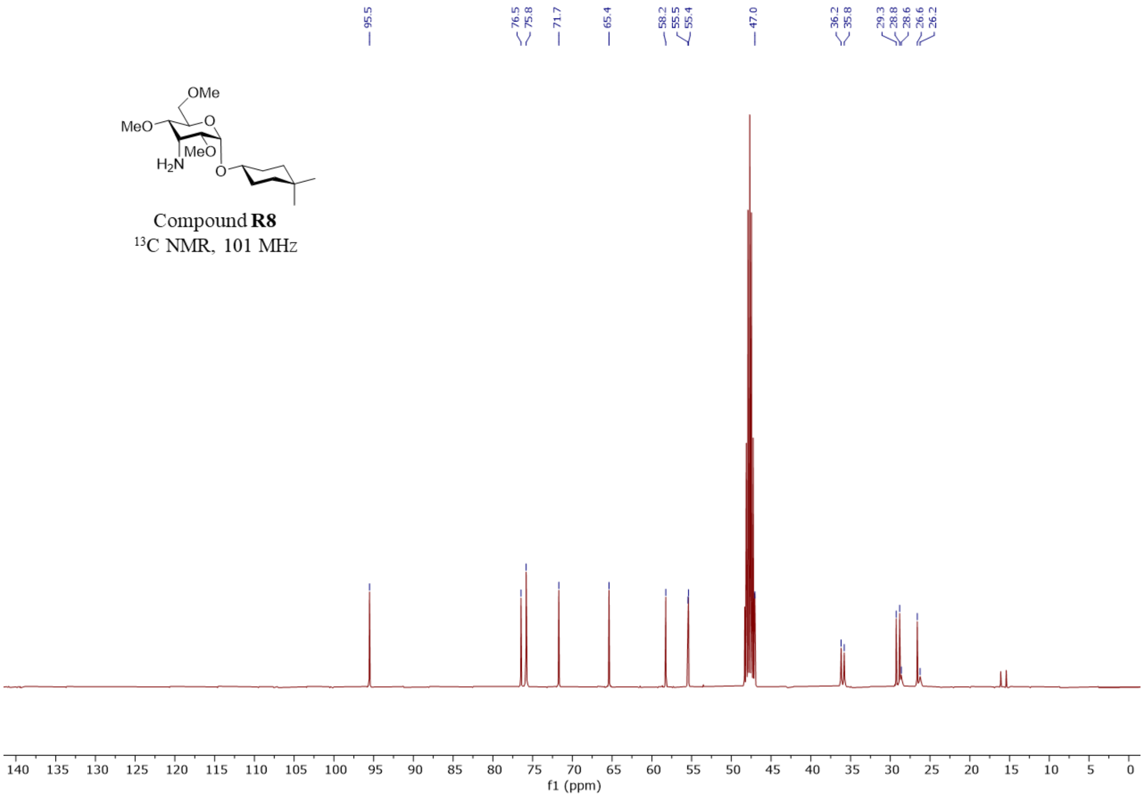

Compound **R8**
^{13}C NMR, 101 MHz

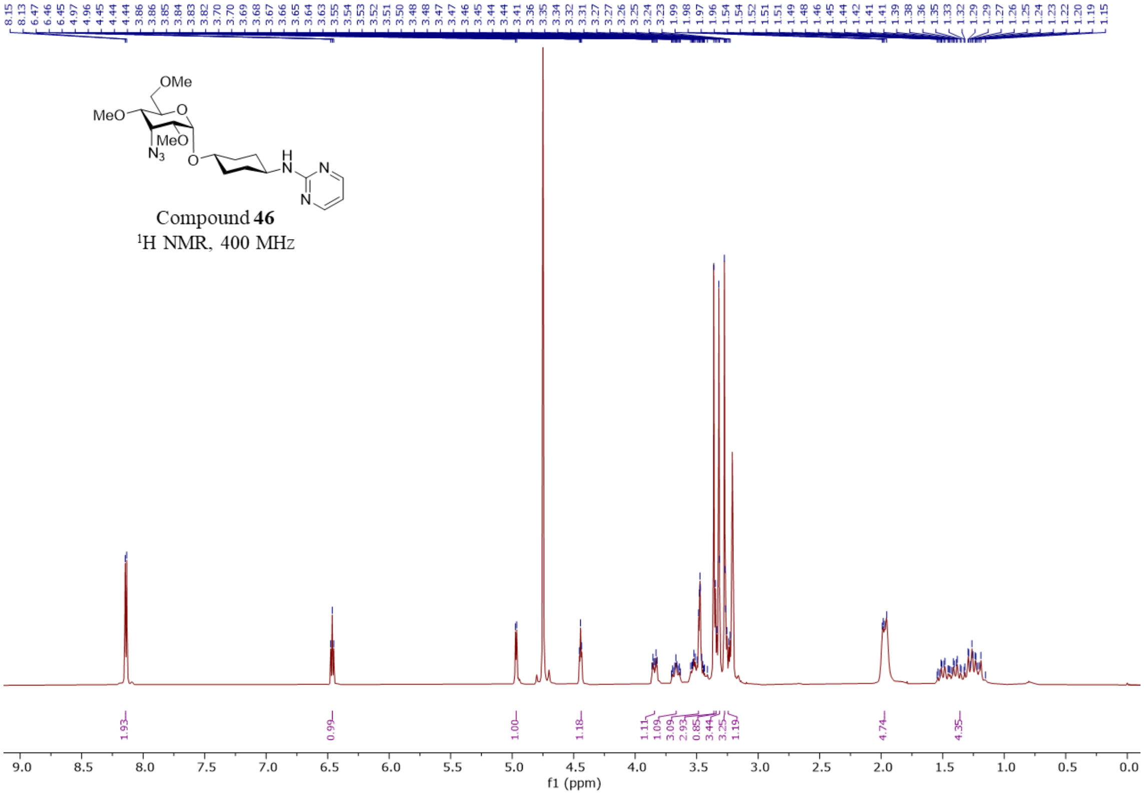
8.15 8.13 6.47 6.46 6.45 4.97 4.96 4.45 4.44 4.44 3.86 3.86 3.85 3.84 3.83 3.82 3.70 3.70 3.69 3.68 3.67 3.66 3.65 3.64 3.63 3.55 3.54 3.53 3.52 3.51 3.50 3.48 3.48 3.47 3.47 3.46 3.45 3.44 3.44 3.41 3.36 3.35 3.34 3.32 3.31 3.27 3.27 3.26 3.25 3.24 3.23 1.99 1.98 1.97 1.96 1.54 1.54 1.52 1.51 1.51 1.49 1.48 1.46 1.45 1.44 1.42 1.41 1.41 1.39 1.38 1.36 1.35 1.33 1.32 1.29 1.29 1.27 1.26 1.25 1.24 1.23 1.22 1.20 1.19 1.15
OMe
MeO
O
MeO
N_3
O
H
N
N
N
Compound **46**
^{1}H NMR, 400 MHz
1.93 0.99 1.00 1.18 1.11 1.09 3.09 2.93 0.85 3.44 3.25 1.19 4.74 4.35
9.0 8.5 8.0 7.5 7.0 6.5 6.0 5.5 5.0 4.5 4.0 3.5 3.0 2.5 2.0 1.5 1.0 0.5 0.0
f1 (ppm)

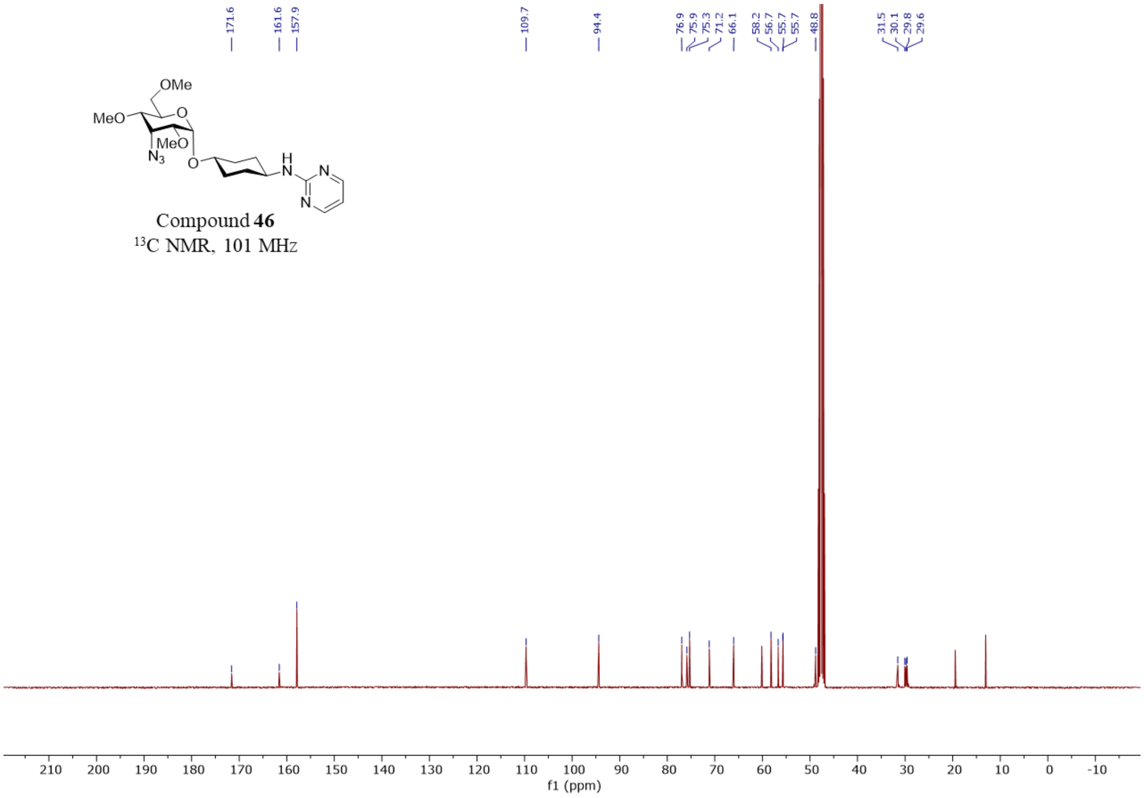
171.6 161.6 157.9 109.7 94.4 76.9 75.9 75.3 71.2 66.1 58.2 56.7 55.7 55.7 48.8 31.5 30.1 29.8 29.6
OMe
MeO
O
MeO
N_3
O
H
N
N
N
Compound **46**
^{13}C NMR, 101 MHz
210 200 190 180 170 160 150 140 130 120 110 100 90 80 70 60 50 40 30 20 10 0 -10
f1 (ppm)

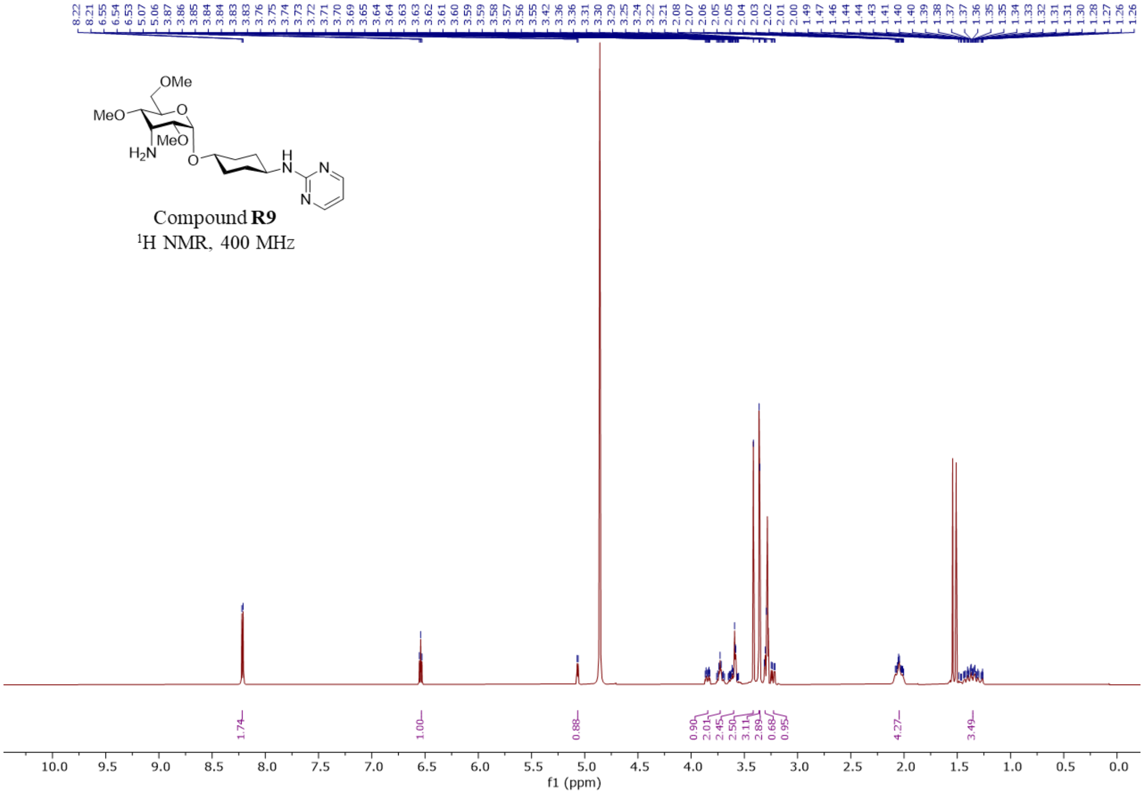
8.22 8.21 6.55 6.54 6.53 5.07 5.06 3.87 3.86 3.85 3.84 3.84 3.83 3.83 3.76 3.75 3.74 3.73 3.72 3.71 3.70 3.69 3.65 3.64 3.64 3.63 3.63 3.62 3.61 3.60 3.59 3.59 3.58 3.57 3.56 3.55 3.42 3.36 3.36 3.31 3.30 3.29 3.25 3.24 3.22 3.21 2.08 2.07 2.06 2.05 2.05 2.04 2.03 2.02 2.01 2.00 1.49 1.47 1.46 1.44 1.44 1.43 1.41 1.40 1.40 1.39 1.38 1.37 1.37 1.36 1.35 1.35 1.34 1.33 1.32 1.31 1.31 1.30 1.28 1.27 1.26 1.26
OMe
MeO
MeO
O
H2N
O
H
N
N
N
Compound R9
1H NMR, 400 MHz
1.74
1.00
0.88
0.90 2.01 2.45 2.50 3.11 2.89 0.68 0.95
4.27
3.49
10.0 9.5 9.0 8.5 8.0 7.5 7.0 6.5 6.0 5.5 5.0 4.5 4.0 3.5 3.0 2.5 2.0 1.5 1.0 0.5 0.0
f1 (ppm)

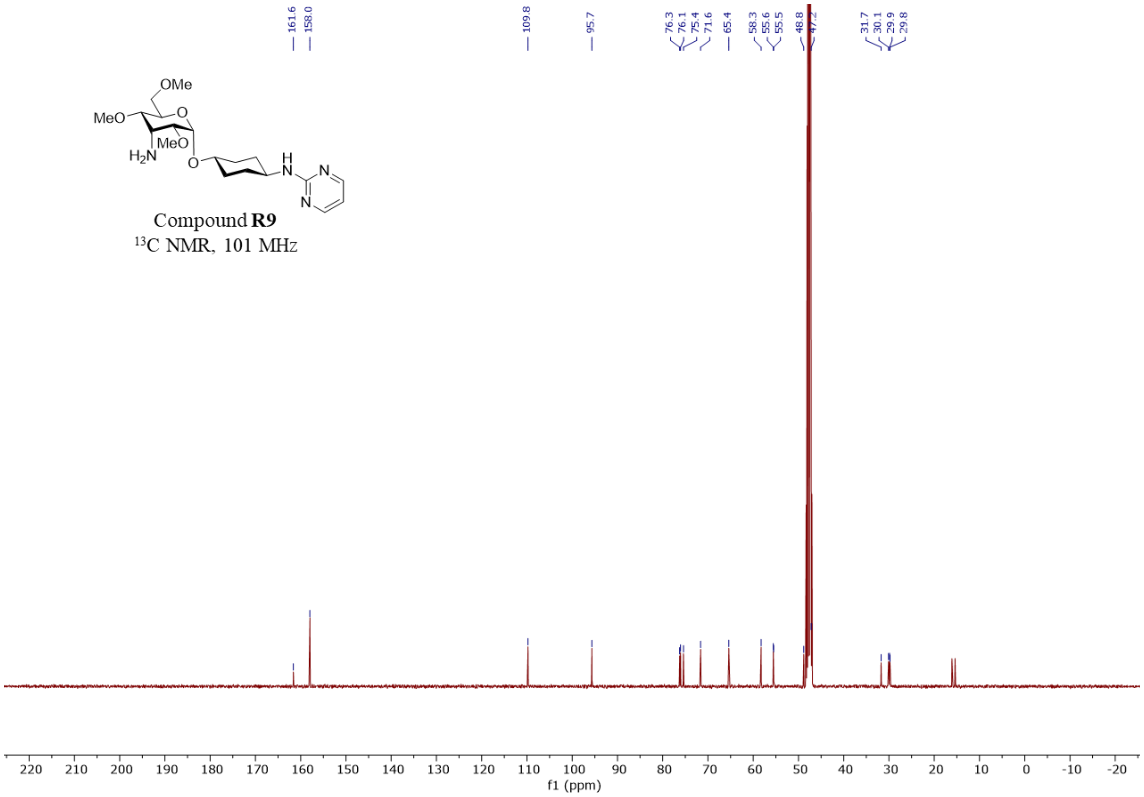
161.6 158.0
109.8
95.7
76.3 76.1 75.4 71.6 65.4 58.3 55.6 55.5 48.8 47.2
31.7 30.1 29.9 29.8
OMe
MeO
MeO
O
H2N
O
H
N
N
N
Compound R9
13C NMR, 101 MHz
220 210 200 190 180 170 160 150 140 130 120 110 100 90 80 70 60 50 40 30 20 10 0 -10 -20
f1 (ppm)

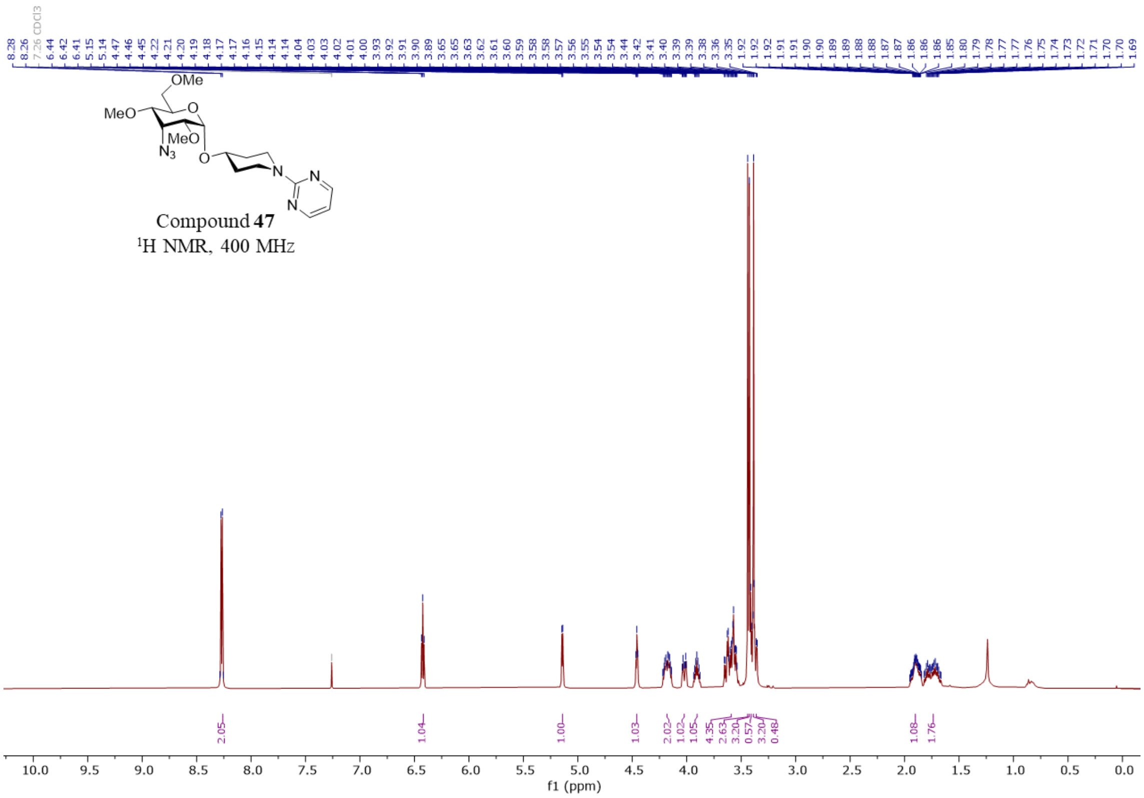
8.28
8.26
7.26 CDCl3
6.44
6.42
6.41
5.15
5.14
4.47
4.46
4.45
4.22
4.21
4.20
4.19
4.18
4.17
4.17
4.16
4.15
4.14
4.14
4.04
4.03
4.03
4.02
4.01
4.00
3.93
3.92
3.91
3.90
3.89
3.65
3.65
3.63
3.62
3.61
3.60
3.59
3.58
3.58
3.57
3.56
3.55
3.54
3.54
3.44
3.42
3.41
3.40
3.39
3.39
3.38
3.36
3.35
1.92
1.92
1.92
1.91
1.90
1.90
1.89
1.89
1.88
1.88
1.87
1.87
1.86
1.86
1.86
1.85
1.80
1.79
1.78
1.77
1.77
1.76
1.75
1.74
1.73
1.72
1.71
1.70
1.69
OMe
MeO
O
MeO
N3
O
N
N
N
Compound 47
1H NMR, 400 MHz
2.05
1.04
1.00
1.03
2.02
1.02
1.05
4.35
2.63
3.20
0.57
3.20
3.20
0.48
1.08
1.76
10.0
9.5
9.0
8.5
8.0
7.5
7.0
6.5
6.0
5.5
5.0
4.5
4.0
3.5
3.0
2.5
2.0
1.5
1.0
0.5
0.0
f1 (ppm)

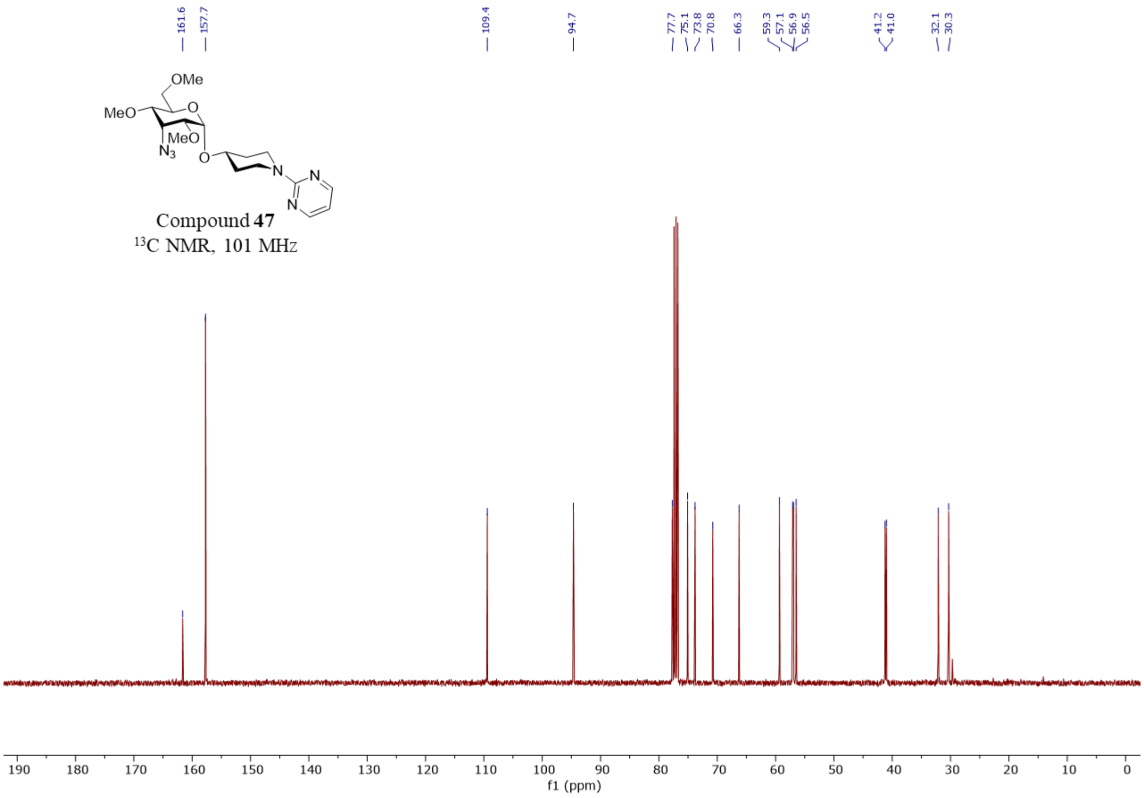
161.6
157.7
109.4
94.7
77.7
75.1
73.8
70.8
66.3
59.3
57.1
56.9
56.5
41.2
41.0
32.1
30.3
OMe
MeO
O
MeO
N3
O
N
N
N
Compound 47
13C NMR, 101 MHz
190
180
170
160
150
140
130
120
110
100
90
80
70
60
50
40
30
20
10
0
f1 (ppm)

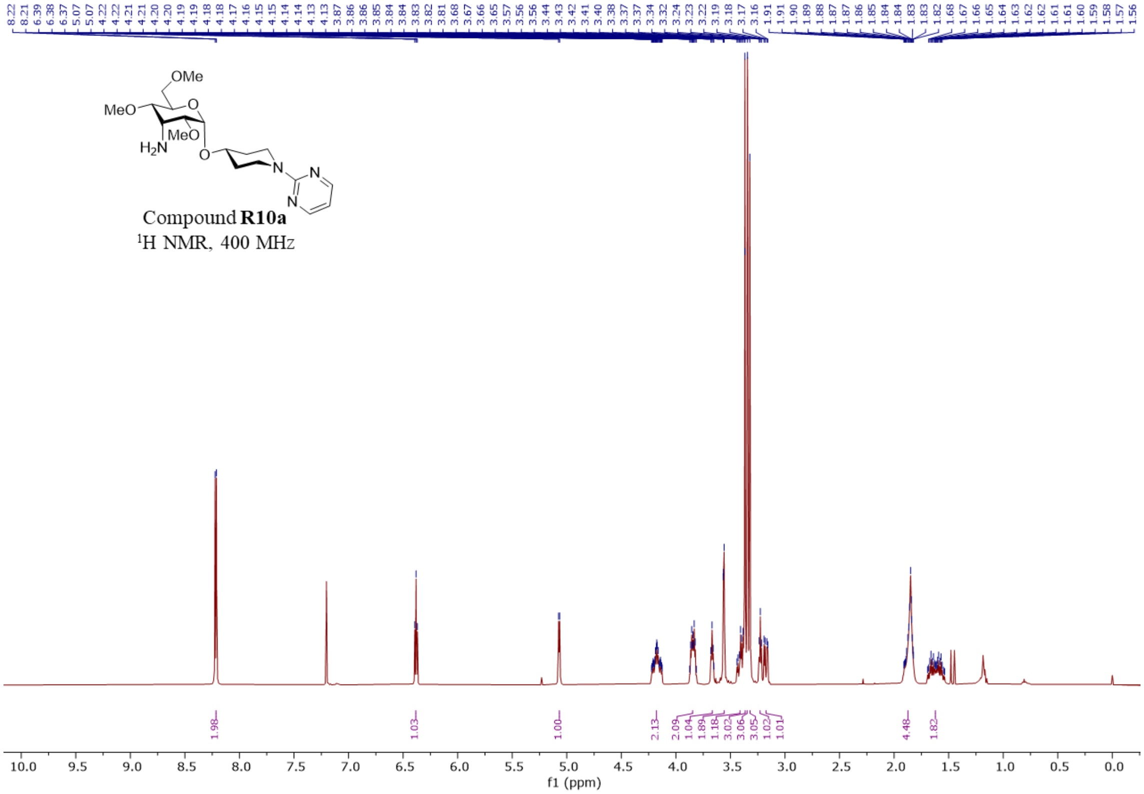
8.22 8.21 6.39 6.38 6.37 5.07 5.07 4.22 4.22 4.21 4.21 4.20 4.20 4.19 4.19 4.18 4.18 4.17 4.16 4.15 4.15 4.14 4.14 4.13 4.13 3.87 3.86 3.86 3.85 3.84 3.84 3.83 3.82 3.81 3.68 3.67 3.66 3.65 3.57 3.56 3.56 3.44 3.43 3.42 3.41 3.40 3.38 3.37 3.34 3.32 3.24 3.23 3.22 3.19 3.18 3.17 3.16 1.91 1.91 1.90 1.89 1.88 1.87 1.87 1.86 1.85 1.84 1.84 1.83 1.83 1.82 1.68 1.67 1.66 1.65 1.64 1.63 1.62 1.62 1.61 1.61 1.60 1.59 1.58 1.57 1.56
OMe
MeO
MeO
H2N
O
N
N
N
Compound **R10a**
1H NMR, 400 MHz
1.98 1.03 1.00 2.13 2.09 1.04 1.89 1.18 3.02 3.06 3.05 1.02 1.01 4.48 1.82
10.0 9.5 9.0 8.5 8.0 7.5 7.0 6.5 6.0 5.5 5.0 4.5 4.0 3.5 3.0 2.5 2.0 1.5 1.0 0.5 0.0
f1 (ppm)

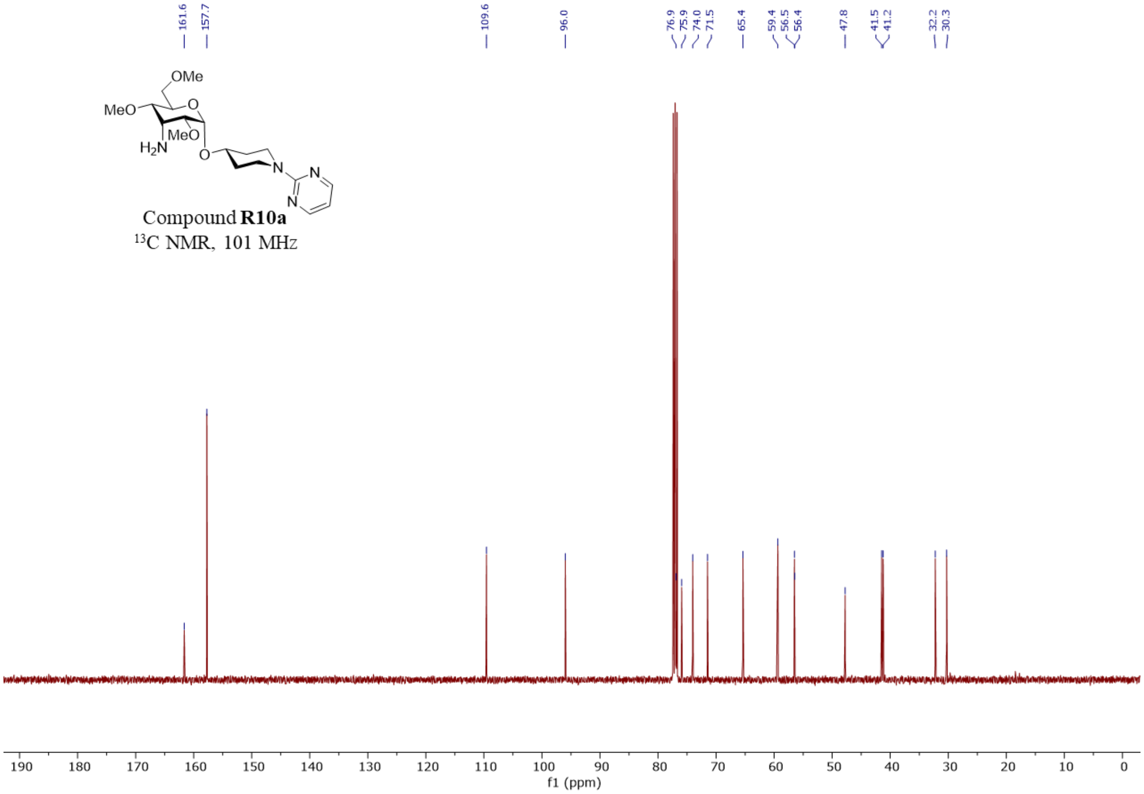
161.6 157.7 109.6 96.0 76.9 75.9 74.0 71.5 65.4 59.4 56.5 56.4 47.8 41.5 41.2 32.2 30.3
OMe
MeO
MeO
H2N
O
N
N
N
Compound **R10a**
13C NMR, 101 MHz
190 180 170 160 150 140 130 120 110 100 90 80 70 60 50 40 30 20 10 0
f1 (ppm)

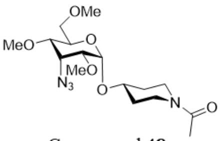

Compound **48**
^{1}H NMR, 400 MHz

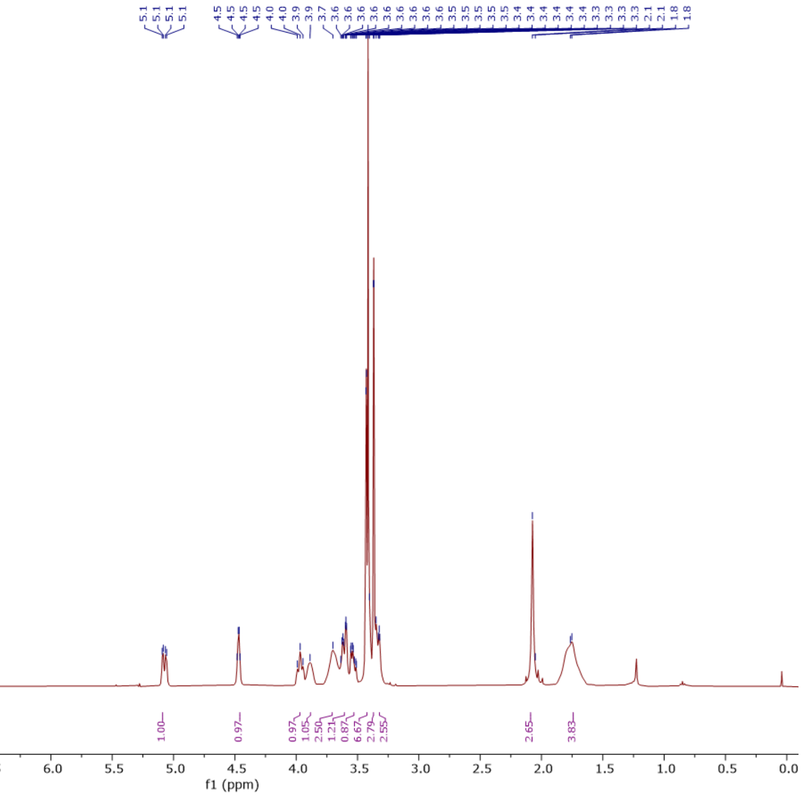

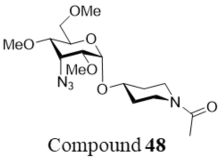

Compound **48**
^{13}C NMR, 101 MHz

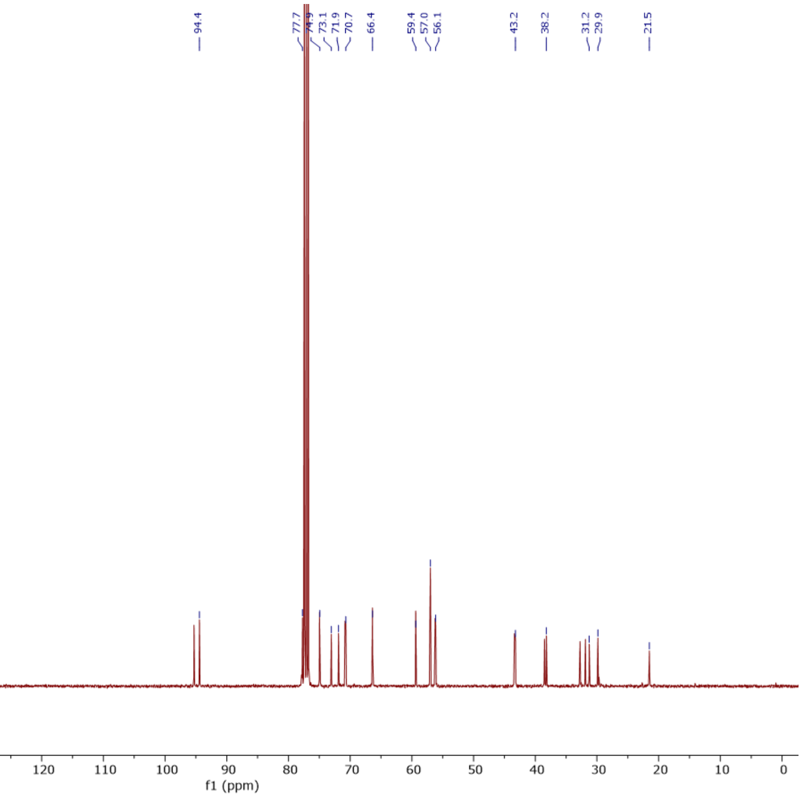

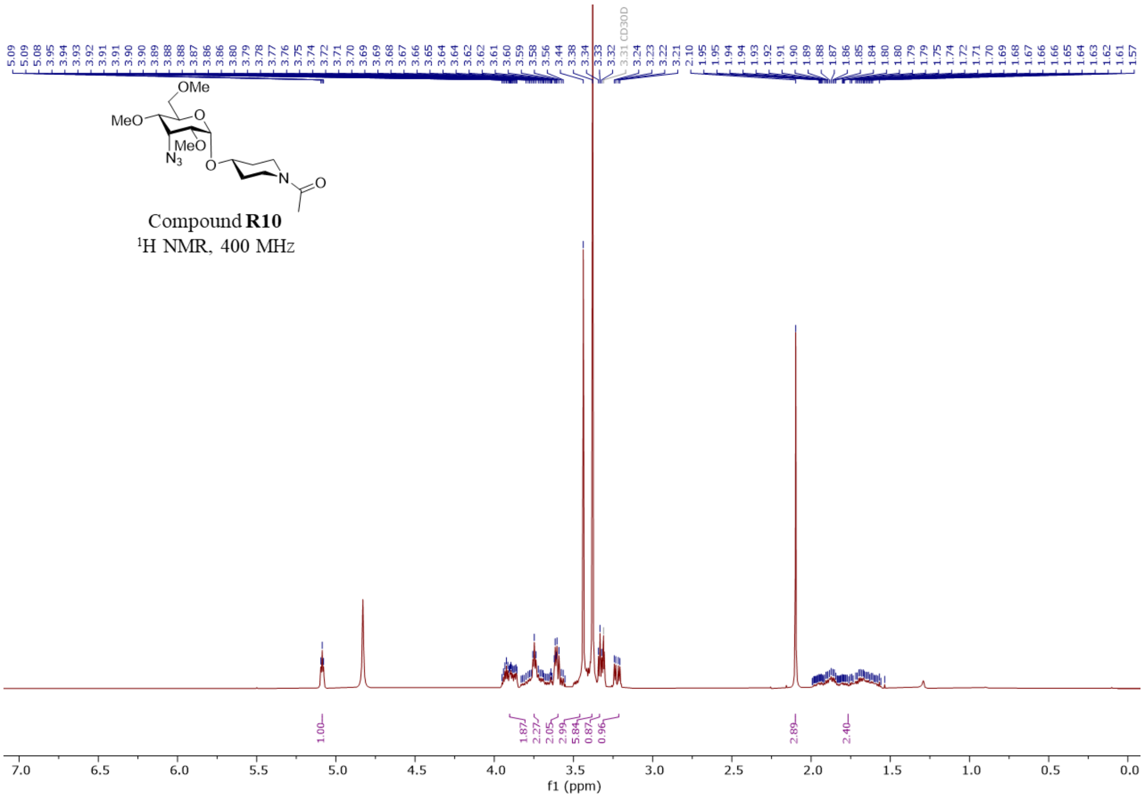
Compound **R10**
^{1}H NMR, 400 MHz
f1 (ppm)

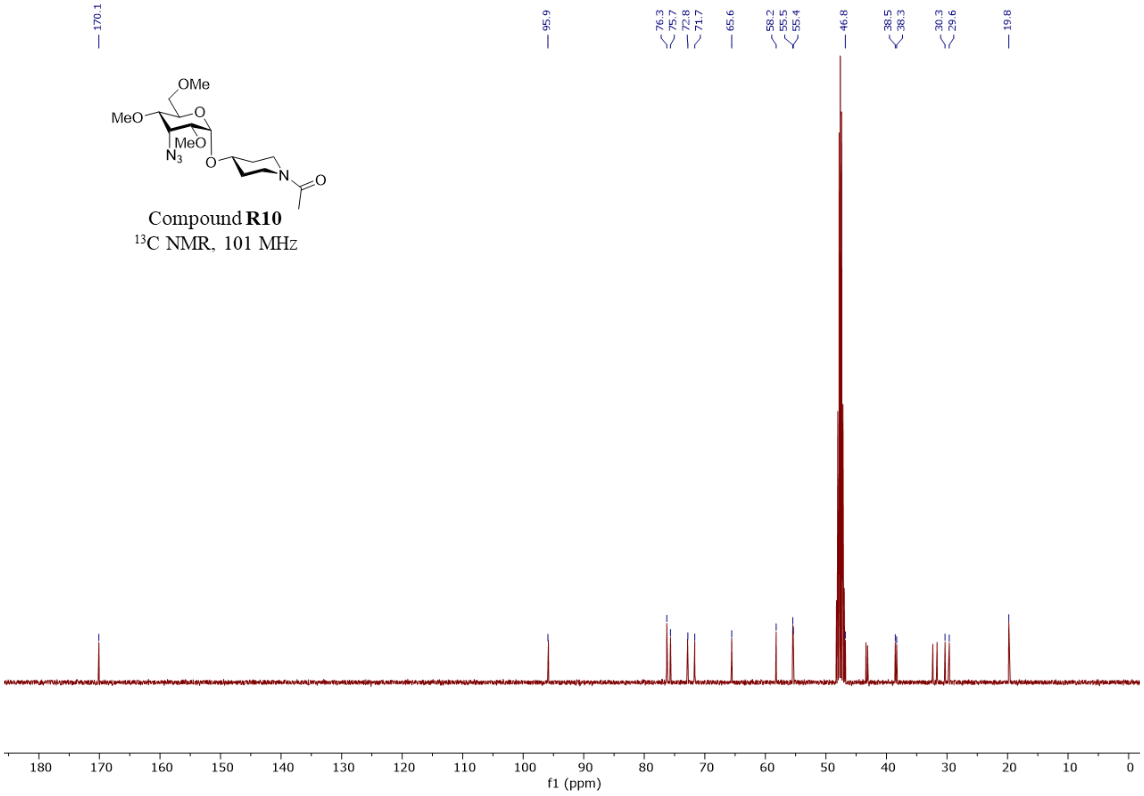
Compound **R10**
^{13}C NMR, 101 MHz
170.1
95.9
76.3
75.7
72.8
71.7
65.6
58.2
55.5
55.4
46.8
38.5
38.3
30.3
29.6
19.8
f1 (ppm)

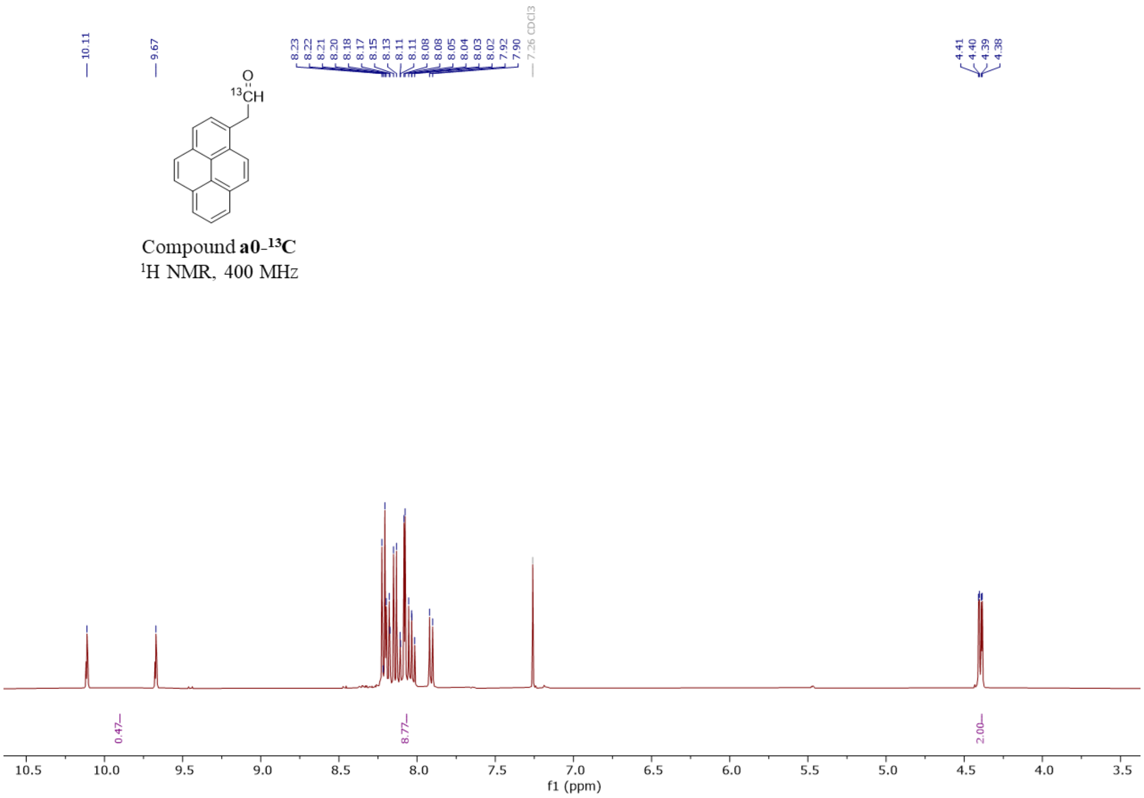

Compound **a0-^{13}C**
^{1}H NMR, 400 MHz

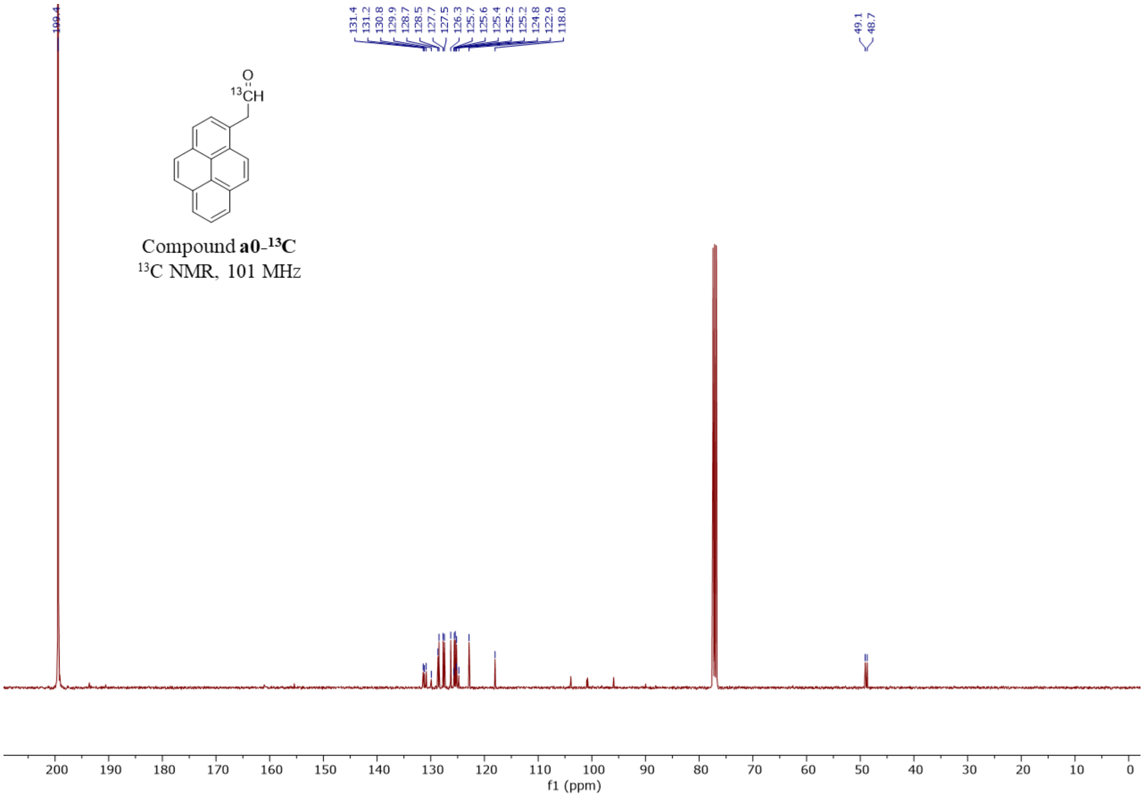

Compound **a0-^{13}C**
^{13}C NMR, 101 MHz

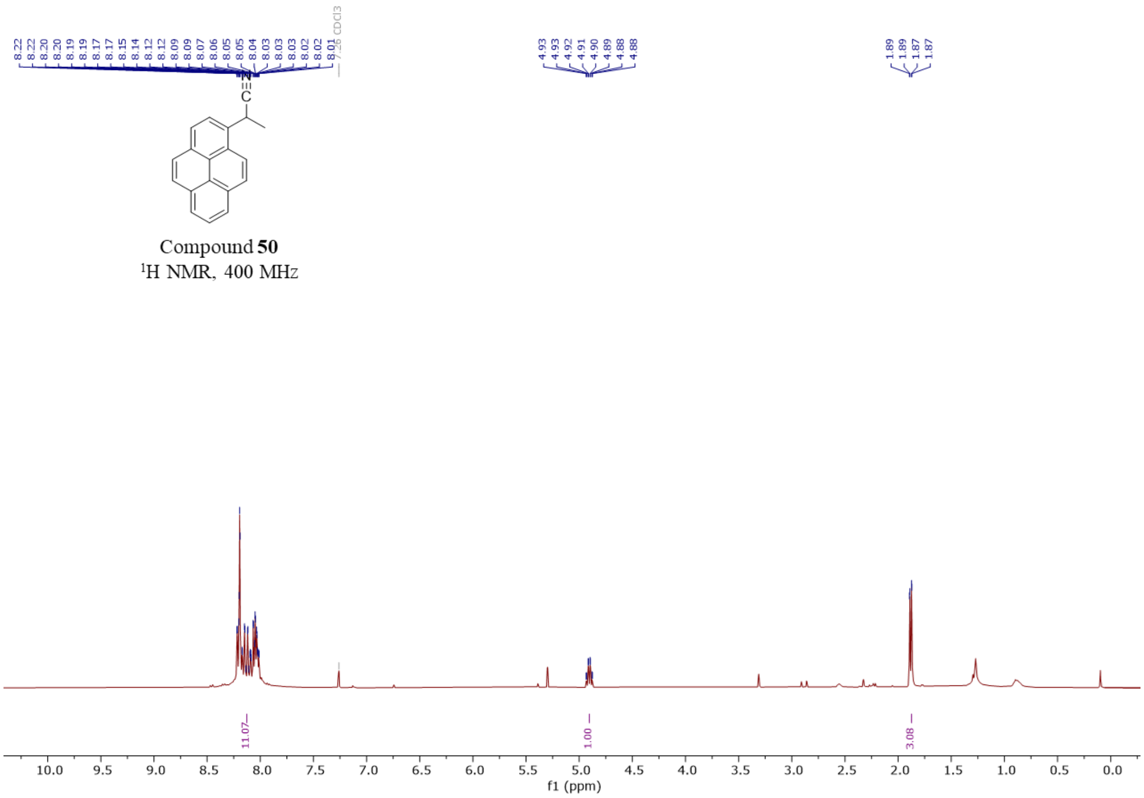
Compound 50
1H NMR, 400 MHz
f1 (ppm)

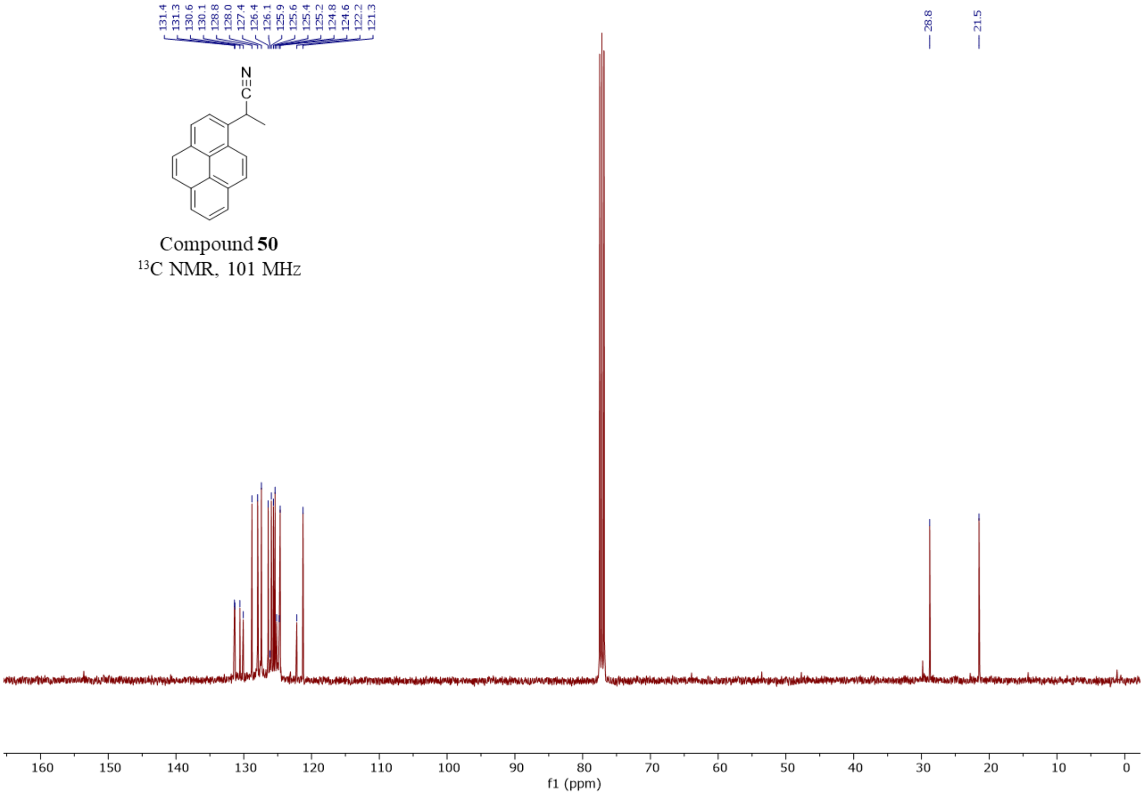
Compound 50
13C NMR, 101 MHz
f1 (ppm)

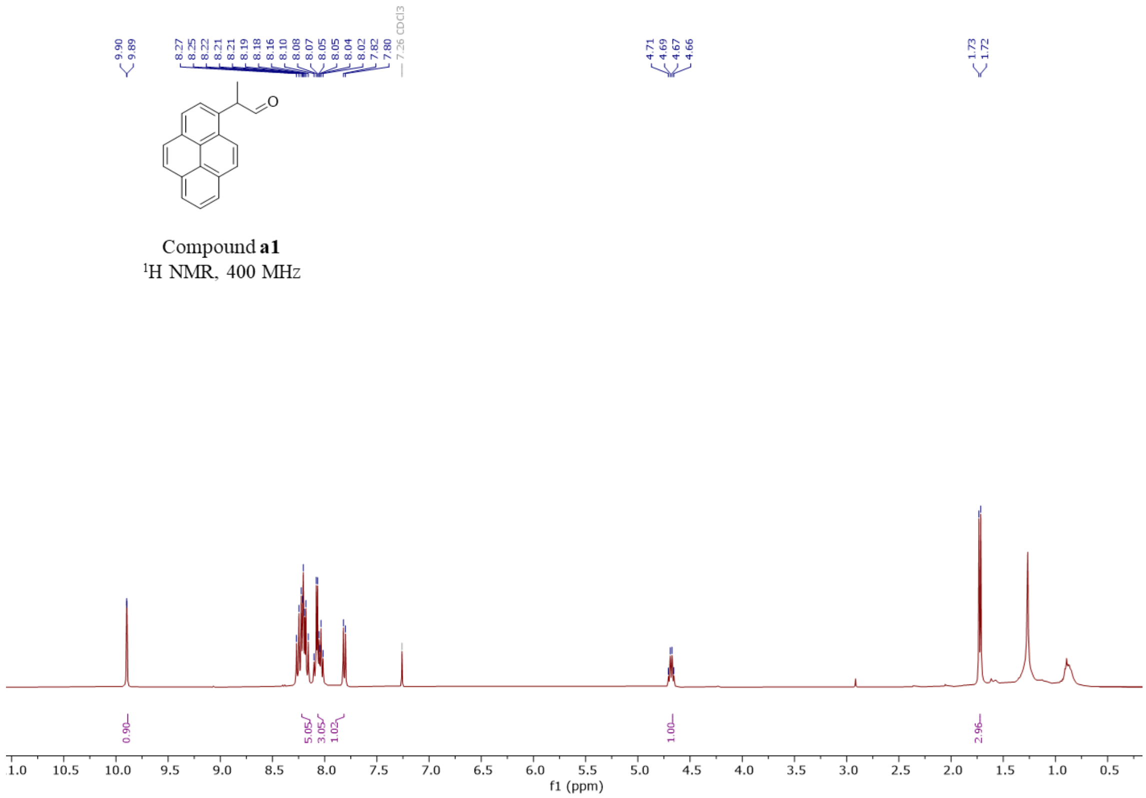
Compound **a1**
^{1}H NMR, 400 MHz
f1 (ppm)

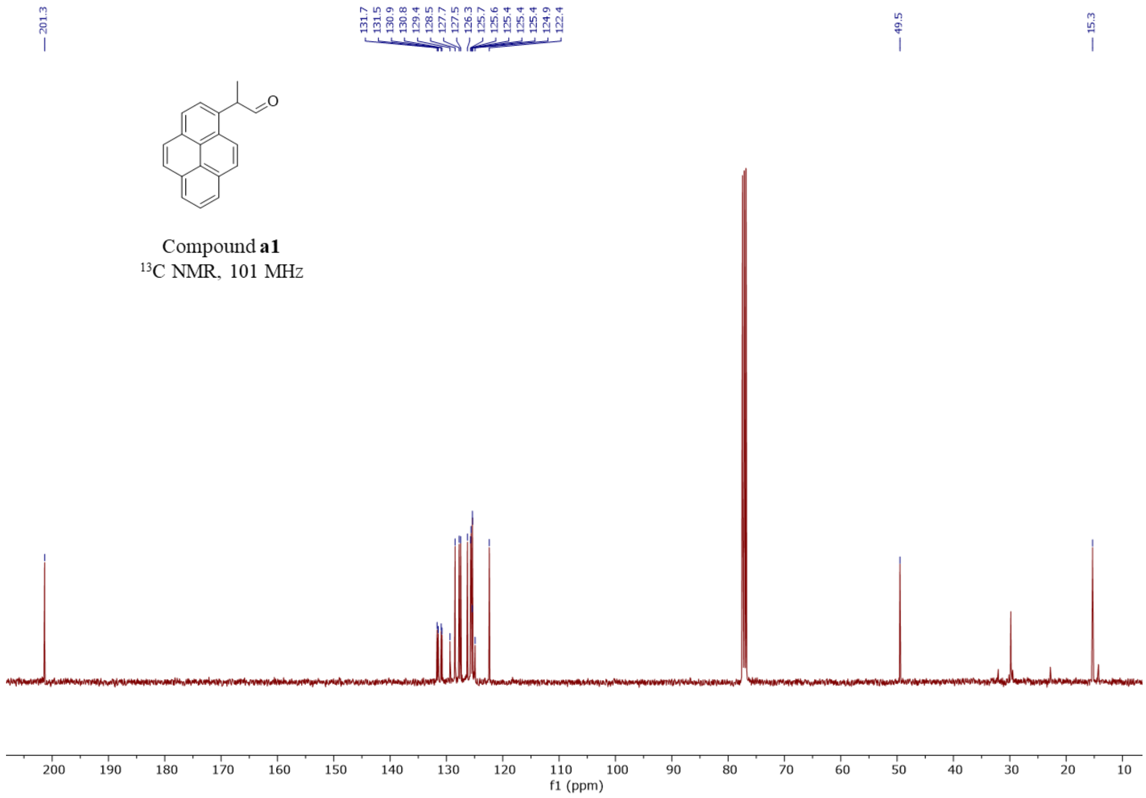
Compound **a1**
^{13}C NMR, 101 MHz
f1 (ppm)

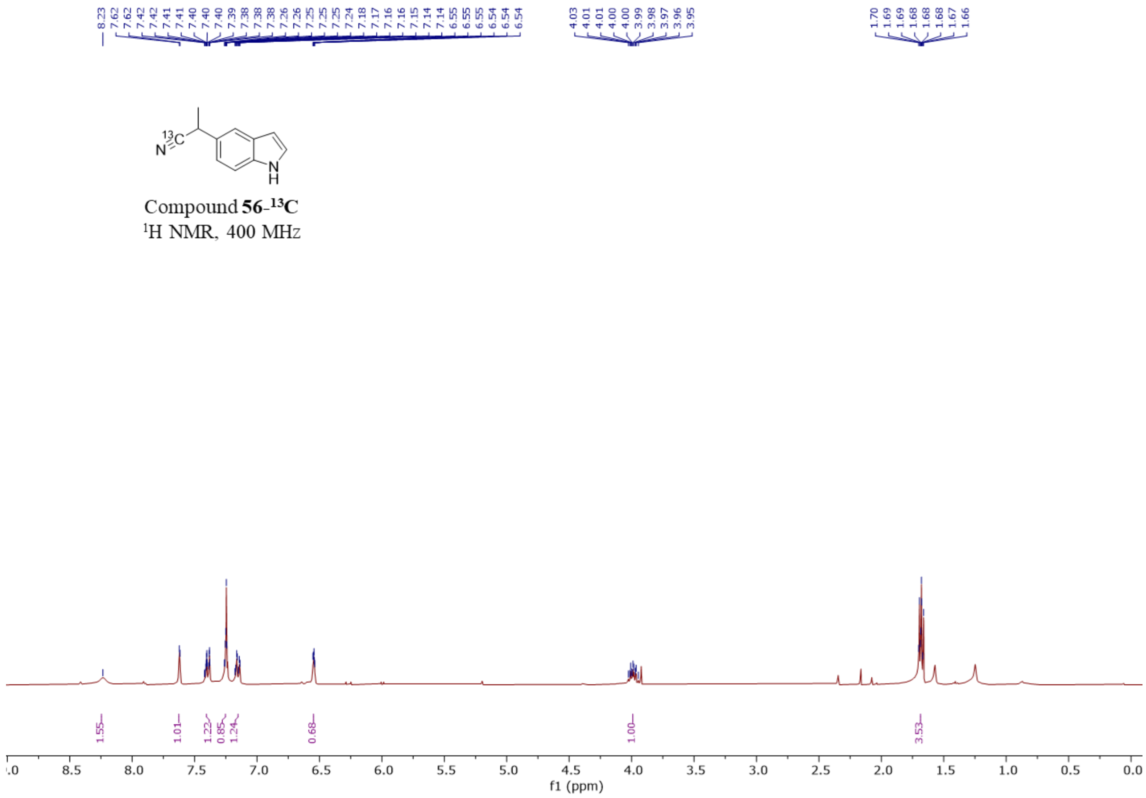
Compound 56-13C
1H NMR, 400 MHz
f1 (ppm)

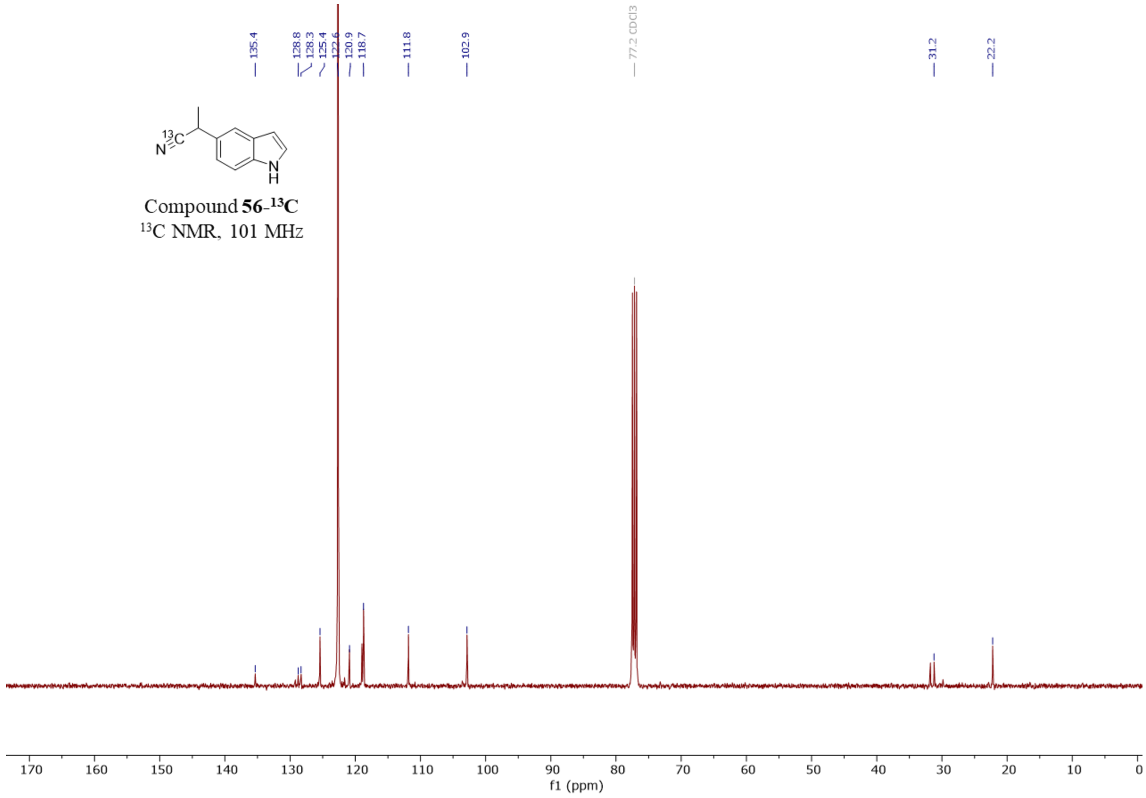
Compound 56-13C
13C NMR, 101 MHz
f1 (ppm)

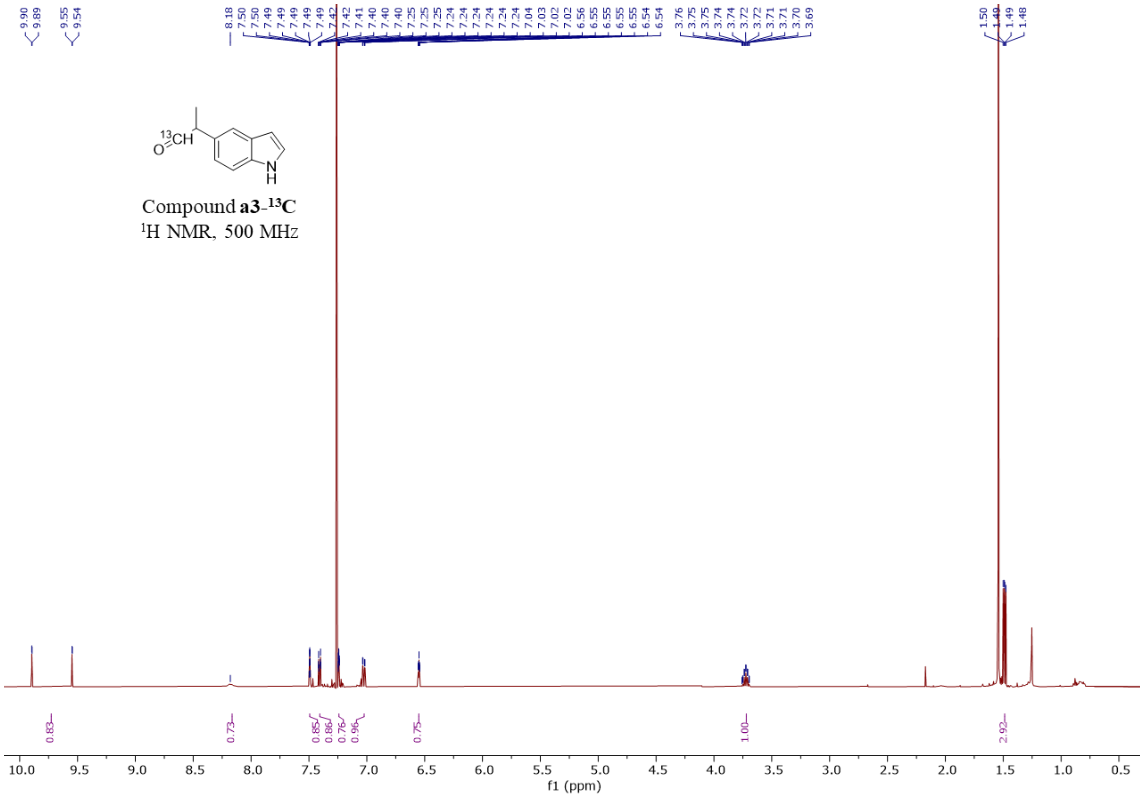

Compound **a3**-**^{13}C**
^{1}H NMR, 500 MHz

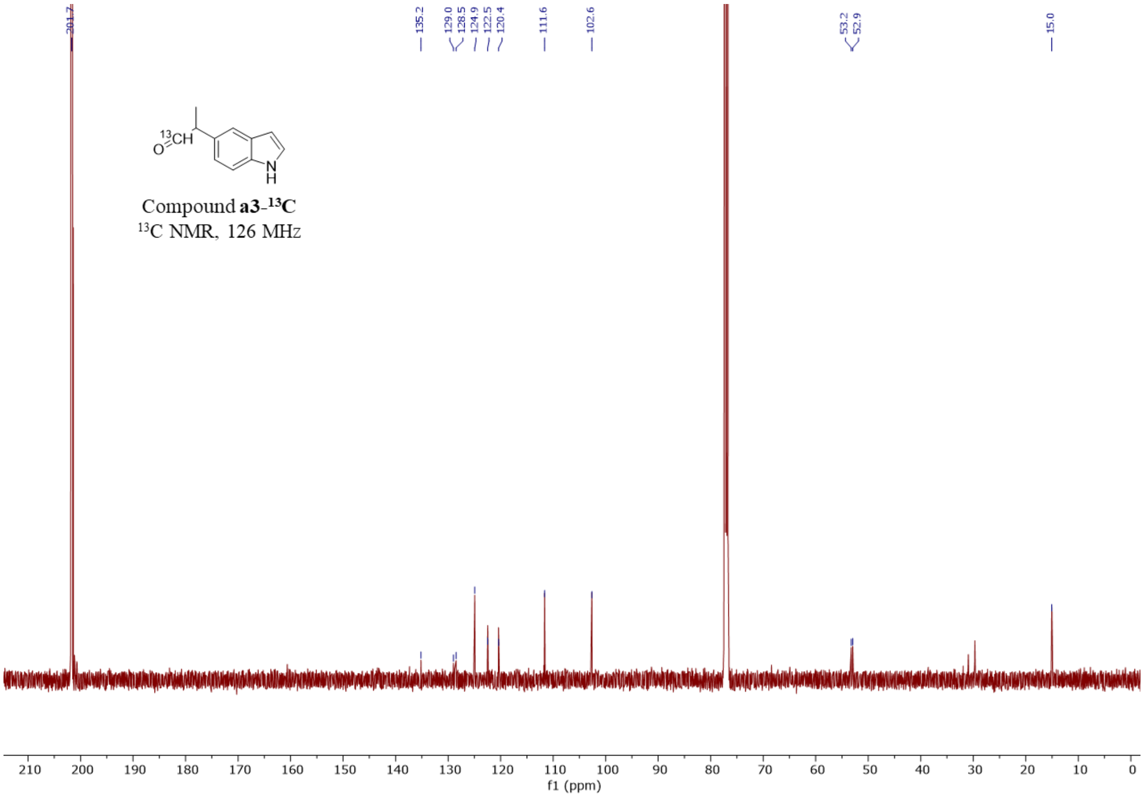

Compound **a3**-**^{13}C**
^{13}C NMR, 126 MHz

3. References:

1. Jiménez-Moreno, E.; Gómez, A. M.; Bastida, A.; Corzana, F.; Jiménez-Oses, G.; Jiménez-Barbero, J.; Asensio, J. L. Modulating Weak Interactions for Molecular Recognition: A Dynamic Combinatorial Analysis for Assessing the Contribution of Electrostatics to the Stability of CH-π Bonds in Water. *Angew. Chem. Int. Ed.* **2015**, *54* (14), 4344–4348.
2. A. Glenn, R.; R. Bailey, J. The Nitrogen Compounds in Petroleum Distillates. XIX. The Isolation from California Petroleum and Synthesis of 2,3,8-Trimethyl-4-Ethylquinoline. *J. Am. Chem. Soc*. **1941**, *63* (3), 637–638.
3. Wollenburg, M.; Heusler, A.; Bergander, K.; Glorius, F. Trans -Selective and Switchable Arene Hydrogenation of Phenol Derivatives. *ACS Catal*. **2020**, *10* (19), 11365–11370.
4. Seifert, K.; Büttner, A.; Rigol, S.; Eilert, N.; Wandel, E.; Giannis, A. Potent Small Molecule Hedgehog Agonists Induce VEGF Expression in Vitro. *Bioorg. Med. Chem*. **2012**, *20* (21), 6465–6481.
5. Trost, B. M.; Osipov, M.; Krüger, S.; Zhang, Y. A Catalytic Asymmetric Total Synthesis of (−)-Perophoramidine. *Chem. Sci*. **2015**, *6* (1), 349–353.
6. Povie, G.; Tran, A.-T.; Bonnaffé, D.; Habegger, J.; Hu, Z.; Le Narvor, C.; Renaud, P. Repairing the Thiol-Ene Coupling Reaction. *Angew. Chem. Int. Ed*. **2014**, *53* (15), 3894–3898.
7. Zhang, Y.; Xie, S.; Yan, M.; Ramström, O. Dynamic Covalent Chemistry of Aldehyde Enamines: Bi III - and Sc III -Catalysis of Amine-Enamine Exchange. *Eur. J. Chem*. **2017**, *23* (49), 11908–11912.
8. Santana, Andrés G.; Francisco, Cosme G.; Suarez, Ernesto; Gonzalez, Concepcion C. *J. Org. Chem*. **2010**, *75*, 5371-5374.
9. van Kalkeren, H. A.; Bruins, J. J.; Rutjes, F. P. J. T.; van Delft, F. L. Organophosphorus-Catalysed Staudinger Reduction. *Adv. Synth. Catal*. **2012**, *354* (8), 1417–1421.
10. Garst, M. E.; Bonfiglio, J. N.; Grudoski, D. A.; Marks, J. Specific Enolates from alpha-Amino Ketones. *J. Org. Chem*. **1980**, *45* (12), 2307–2315.
11. Wessig, P.; Möllnitz, K. Nanoscale Molecular Rods with a New Building Block for Solubility Enhancement. *J. Org. Chem*. **2008**, *73* (12), 4452–4457.
12. Melnykov, K. P.; Nosik, P. S.; Kurpil, B. B.; Sibgatulin, D. A.; Volochnyuk, D. M.; Ryabukhin, S. v.; Grygorenko, O. O. Synthesis of Gem -Difluorocyclopentane/Hexane Building Blocks. *J. Fluor Chem*. **2017**, *199*, 60–66.

13. Xia, H.; Zhang, F.; Ye, T.; Wang, Y. Selective A-Monomethylation by an Amine-Borane/ N,N-Dimethylformamide System as the Methyl Source. *Angew. Chem. Int. Ed*. **2018**, *57* (36), 11770–11775.

14. Klassen, S. E.; Daub, G. H.; VanderJagt, D. L. Carbon-13 Labeled Benzo[a]Pyrenes and Derivatives. 4. Labeling the 7-10 Positions. *J. Org. Chem*. **1983**, *48* (23), 4361–4366.

15. Miyamoto, K.; Tsutsumi, T.; Terao, Y.; Ohta, H. Stereochemistry of Decarboxylation of Arylmalonate Catalyzed by Mutant Enzymes. *Chem. Lett*. **2007**, *36* (5), 656–657.

16. Ellingboe, J. W.; Lombardo, L. J.; Alessi, T. R.; Nguyen, T. T.; Guzzo, F.; Guinosso, C. J.; Bullington, J.; Browne, E. N. C.; Bagli, J. F. Antihyperglycemic Activity of Novel Naphthalenylmethyl-3H-1,2,3,5-Oxathiadiazole 2-Oxides. *J. Med. Chem.* **1993**, *36* (17), 2485–2493.

17. Jiménez-Moreno, E.; Jiménez-Osés, G.; Gómez, A. M.; Santana, A. G.; Corzana, F.; Bastida, A.; Jiménez-Barbero, J.; Asensio, J. L. A Thorough Experimental Study of CH/π Interactions in Water: Quantitative Structure–Stability Relationships for Carbohydrate/Aromatic Complexes. *Chem. Sci*. **2015**, *6* (11), 6076–6085.

4. Tables S1-S6

Table S1. Equilibrium $K_{Z/E}$ values (given by [Z]/[E]) together the resulting free energies $\Delta G_{Z/E}$ (kcal/mol, in bold) measured with our 3-amine-3-deoxy-allose derivatives and aldehyde **a1** in various environments.

	**$CDCl_3/CD_2Cl_2$ 90/10**	**$CDCl_3/CD_2Cl_2$ 75/25**	**CD_2Cl_2**	**Dioxane-d_8**	**Nitromethane-d_3**	**THF-d_8**	**Nitrobenzene-d_5**	**CD_3OD**	**Pyridine-d_5**	**Acetonitrile-d_3**	**DMSO-d_6**
R0α	0.36<br>**0.59±0.03**	0.36<br>**0.59±0.03**	0.45<br>**0.46±0.03**	0.42<br>**0.51±0.03**	0.59<br>**0.31±0.03**	0.40<br>**0.53±0.03**	0.39<br>**0.55±0.03**	3.0<br>**-0.64±0.03**	0.40<br>**0.53±0.03**	0.77<br>**0.15±0.03**	0.91<br>**0.05±0.03**
M1	**X**	0.44<br>**0.48±0.03**	0.56<br>**0.34±0.03**	0.83<br>**0.11±0.03**	1.1<br>**-0.06±0.03**	0.56<br>**0.34±0.03**	0.50<br>**0.40±0.03**	**X**	0.63<br>**0.27±0.03**	1.4<br>**-0.20±0.03**	1.3<br>**-0.15±0.03**
M2	**X**	1.2<br>**-0.11±0.03**	1.4<br>**-0.20±0.03**	1.5<br>**-0.24±0.03**	2.7<br>**-0.58±0.03**	1.2<br>**-0.11±0.03**	0.83<br>**0.11±0.03**	**X**	1.2<br>**-0.11±0.03**	3.9<br>**-0.79±0.03**	2.3<br>**-0.48±0.03**
R3	1.0<br>**0±0.03**	1.1<br>**-0.06±0.03**	1.3<br>**-0.15±0.03**	1.5<br>**-0.24±0.03**	2.7<br>**-0.58±0.03**	1.3<br>**-0.15±0.03**	0.91<br>**0.05±0.03**	11.0<br>**-1.391±0.08**	1.3<br>**-0.15±0.03**	3.9<br>**-0.79±0.03**	2.2<br>**-0.46±0.03**
M3	2.8<br>**-0.60±0.03**	2.7<br>**-0.58±0.03**	3.0<br>**-0.63±0.03**	2.4<br>**-0.51±0.03**	4.8<br>**-0.91±0.03**	2.0<br>**-0.40±0.03**	1.7<br>**-0.31±0.03**	25.0<br>**-1.87±0.10**	2.2<br>**-0.46±0.03**	6.9<br>**-1.12±0.05**	3.3<br>**-0.69±0.03**
R4	**X**	0.33<br>**0.64±0.03**	0.39<br>**0.55±0.03**	0.71<br>**0.20±0.03**	0.91<br>**0.05±0.03**	0.59<br>**0.31±0.03**	0.33<br>**0.64±0.03**	**X**	0.44<br>**0.48±0.03**	1.0<br>**-0±0.03**	0.91<br>**0.05±0.03**
M4	**X**	9.0<br>**-1.27±0.05**	8.0<br>**-1.21±0.05**	4.8<br>**-0.91±0.03**	3.5<br>**-0.73±0.03**	**X**	1.8<br>**-0.34±0.03**	**X**	3.0<br>**-0.64±0.03**	3.5<br>**-0.73±0.03**	1.1<br>**-0.06±0.03**
R5	**X**	0.63<br>**0.27±0.03**	0.77<br>**0.15±0.03**	1.3<br>**-0.15±0.03**	1.7<br>**-0.31±0.03**	0.77<br>**0.15±0.03**	0.53<br>**0.37±0.03**	**X**	0.77<br>**0.15±0.03**	2.3<br>**-0.48±0.03**	2.2<br>**-0.46±0.03**
M5	**X**	6.5<br>**-1.09±0.05**	5.6<br>**-1.00±0.05**	6.0<br>**-1.04±0.05**	3.9<br>**-0.79±0.03**	**X**	1.7<br>**-0.31±0.03**	**X**	3.9<br>**-0.79±0.03**	4.5<br>**-0.87±0.03**	2.3<br>**-0.48±0.03**
R6	0.91<br>**0.05±0.03**	1.0<br>**0±0.03**	1.3<br>**-0.14±0.03**	1.8<br>**-0.34±0.03**	3.0<br>**-0.64±0.03**	1.3<br>**-0.15±0.03**	0.91<br>**0.05±0.03**	9.0<br>**-1.27±0.05**	1.3<br>**-0.15±0.03**	4.2<br>**-0.83±0.03**	2.3<br>**-0.48±0.03**
M6	9.7<br>**-1.32±0.05**	8.6<br>**-1.25±0.05**	8.0<br>**-1.21±0.05**	4.7<br>**-0.90±0.03**	4.1<br>**-0.82±0.03**	2.6<br>**-0.55±0.03**	1.8<br>**-0.34±0.03**	15.4<br>**-1.58±0.10**	1.8<br>**-0.34±0.03**	4.7<br>**-0.90±0.03**	1.6<br>**-0.27±0.03**
M6$_{Et}$	**X**	4.5<br>**-0.87±0.03**	3.6<br>**-0.74±0.03**	2.4<br>**-0.51±0.03**	3.6<br>**-0.74±0.03**	1.6<br>**-0.27±0.03**	1.5<br>**-0.24±0.03**	**X**	1.5<br>**-0.24±0.03**	3.8<br>**-0.77±0.03**	1.3<br>**-0.15±0.03**
R7	**X**	0.67<br>**0.23±0.03**	0.91<br>**0.05±0.03**	1.2<br>**-0.11±0.03**	1.7<br>**-0.31±0.03**	0.91<br>**0.05±0.03**	0.67<br>**0.23±0.03**	**X**	1.1<br>**-0.06±0.03**	2.3<br>**-0.48±0.03**	1.8<br>**-0.34±0.03**

	$CDCl_3/CD_2Cl_2$ 90/10	$CDCl_3/CD_2Cl_2$ 75/25	CD_2Cl_2	Dioxane-d_8	Nitromethane-d_3	THF-d_8	Nitrobenzene-d_5	CD_3OD	Pyridine-d_5	Acetonitrile-d_3	DMSO-d_6
M7	**X**	20.0<br>**-1.74±0.10**	12.8<br>**-1.48±0.08**	6.4<br>**-1.08±0.05**	4.5<br>**-0.87±0.03**	3.4<br>**-0.71±0.03**	2.6<br>**-0.55±0.03**	**X**	3.3<br>**-0.69±0.03**	4.7<br>**-0.90±0.03**	1.4<br>**-0.20±0.03**
R8	**X**	0.48<br>**0.43±0.03**	0.59<br>**0.31±0.03**	0.72<br>**0.19±0.03**	1.5<br>**-0.24±0.03**	0.59<br>**0.31±0.03**	0.42<br>**0.50±0.03**	**X**	0.67<br>**0.23±0.03**	1.5<br>**-0.24±0.03**	1.2<br>**-0.11±0.03**
M8	**X**	21.0<br>**-1.77±0.10**	12.6<br>**-1.47±0.08**	4.2<br>**-0.83±0.03**	3.1<br>**-0.66±0.03**	2.2<br>**-0.46±0.03**	2.0<br>**-0.40±0.03**	**X**	2.5<br>**-0.53±0.03**	2.7<br>**-0.58±0.03**	0.67<br>**0.23±0.03**
R9	**X**	0.56<br>**0.34±0.03**	0.72<br>**0.19±0.03**	0.77<br>**0.15±0.03**	1.3<br>**-0.15±0.03**	0.59<br>**0.31±0.03**	0.44<br>**0.48±0.03**	**X**	0.63<br>**0.27±0.03**	1.9<br>**-0.37±0.03**	1.1<br>**-0.06±0.03**
M9	**X**	3.7<br>**-0.76±0.03**	2.9<br>**-0.62±0.03**	0.83<br>**0.11±0.03**	1.7<br>**-0.31±0.03**	0.53<br>**0.37±0.03**	0.77<br>**0.15±0.03**	**X**	0.40<br>**0.53±0.03**	1.8<br>**-0.34±0.03**	0.53<br>**0.37±0.03**
R10	**X**	1.6<br>**-0.27±0.03**	1.5<br>**-0.22±0.03**	1.4<br>**-0.20±0.03**	1.9<br>**-0.37±0.03**	1.2<br>**-0.11±0.03**	0.91<br>**0.05±0.03**	**X**	2.3<br>**-0.48±0.03**	2.6<br>**-0.55±0.03**	1.4<br>**-0.20±0.03**
M10	**X**	6.8<br>**-1.11±0.05**	5.9<br>**-1.03±0.05**	2.1<br>**-0.43±0.03**	2.7<br>**-0.58±0.03**	1.3<br>**-0.15±0.03**	1.4<br>**-0.20±0.03**	**X**	1.5<br>**-0.24±0.03**	3.4<br>**-0.71±0.03**	0.71<br>**0.20±0.03**
R11	**X**	**X**	1.4<br>**-0.20±0.03**	**X**	**X**	**X**	**X**	**X**	**X**	**X**	**X**
M11	**X**	**X**	0.56<br>**0.34±0.03**	**X**	**X**	**X**	**X**	**X**	**X**	**X**	**X**
R12	**X**	0.53<br>**0.37±0.03**	0.59<br>**0.31±0.03**	0.59<br>**0.31±0.03**	0.91<br>**0.05±0.03**	0.40<br>**0.53±0.03**	0.40<br>**0.53±0.03**	**X**	**X**	1.1<br>**-0.06±0.03**	**X**
M12	**X**	0.12<br>**1.23±0.05**	0.17<br>**1.03±0.05**	0.29<br>**0.72±0.03**	0.33<br>**0.64±0.03**	0.24<br>**0.83±0.03**	0.11<br>**1.28±0.05**	**X**	0.34<br>**0.63±0.03**	0.30<br>**0.70±0.03**	0.32<br>**0.66±0.03**
R13	**X**	**X**	0.77<br>**0.15±0.03**	**X**	**X**	**X**	**X**	**X**	**X**	**X**	**X**
M13	**X**	**X**	0.44<br>**0.48±0.03**	**X**	**X**	**X**	**X**	**X**	**X**	**X**	**X**

Table S2. Interaction free energies ΔG_{int} (kcal/mol) together with the corresponding polarization or charge contributions (ΔG_{pol} or ΔG_{charge}, in bold) measured with our 3-amine-3-deoxy-allose derivatives and aldehyde **a1** in various environments.

	**$CDCl_3/CD_2Cl_2$**	**$CDCl_3/CD_2Cl_2$**	**CD_2Cl_2**	**Dioxane-d_8**	**Nitromethane-d_3**	**THF-d_8**	**Nitrobenzene-d_5**	**CD_3OD**	**Pyridine-d_5**	**Acetonitrile-d_3**	**DMSO-d_6**
M1	X	-0.11±0.04	-0.12±0.04	-0.40±0.04	-0.37±0.04	-0.19±0.04	-0.15±0.04	X	-0.26±0.04	-0.35±0.04	-0.20±0.04
M2	X	-0.70±0.04	0.66±0.04	-0.75±0.04	-0.89±0.04	-0.64±0.04	-0.44±0.04	X	-0.64±0.04	-0.94±0.04	-0.53±0.04
R3	-0.59±0.04	-0.65±0.04	-0.61±0.04	-0.75±0.04	-0.89±0.04	-0.68±0.04	-0.500±0.04	-0.75±0.09	-0.68±0.04	-0.94±0.04	-0.51±0.04
M3	-1.19±0.04<br>***-0.60*±0.04**	-1.17±0.04<br>***-0.52*±0.04**	-1.09±0.04<br>***-0.48*±0.04**	-1.02±0.04<br>***-0.27*±0.04**	-1.22±0.04<br>***-0.33*±0.04**	-0.93±0.04<br>***-0.25*±0.04**	-0.86±0.04<br>***-0.36*±0.04**	-1.23±0.10<br>***-0.48*±0.13**	-0.99±0.04<br>***-0.31*±0.04**	-1.27±0.06<br>***-0.33*±0.06**	-0.74±0.04<br>***-0.23*±0.04**
R4	X	0.05±0.04	0.09±0.04	-0.31±0.04	-0.26±0.04	-0.22±0.04	0.09±0.04	X	-0.05±0.04	-0.15±0.042	0±0.04
M4	X	-1.86±0.06<br>***-1.91*±0.06**	-1.67±0.06<br>***-1.76*±0.06**	-1.42±0.04<br>***-1.11*±0.04**	-1.04±0.04<br>***-0.78*±0.04**	X	-0.89±0.04<br>***-0.98*±0.04**	X	-1.17±0.04<br>***-1.12*±0.04**	-0.88±0.04<br>***-0.73*±0.04**	-0.11±0.04<br>***-0.11*±0.04**
R5	X	-0.32±0.04	-0.31±0.04	-0.66±0.04	-0.62±0.04	-0.38±0.04	-0.18±0.04	X	-0.38±0.04	-0.63±0.04	-0.51±0.04
M5	X	-1.68±0.06<br>***-1.36*±0.06**	-1.46±0.06<br>***-1.15*±0.06**	-1.55±0.06<br>***-0.89*±0.06**	-1.10±0.04<br>***-0.48*±0.04**	X	-0.86±0.04<br>***-0.68*±0.04**	X	-1.32±0.04<br>***-0.94*±0.04**	-1.02±0.04<br>***-0.39*±0.04**	-0.53±0.04<br>***-0.02*±0.04**
R6	-0.54±0.04	-0.59±0.04	-0.60±0.04	-0.85±0.04	-0.95±0.042	-0.68±0.04	-0.50±0.04	-0.63±0.06	-0.68±0.04	-0.98±0.04	-0.53±0.04
M6	-1.91±0.06<br>***-1.37*±0.06**	-1.84±0.06<br>***-1.25*±0.06**	-1.67±0.06<br>***-1.07*±0.06**	-1.41±0.04<br>***-0.56*±0.04**	-1.13±0.04<br>***-0.18*±0.04**	-1.08±0.04<br>***-0.40*±0.04**	-0.89±0.04<br>***-0.39*±0.04**	-0.94±0.10<br>***-0.31*±0.11**	-0.87±0.04<br>***-0.19*±0.04**	-1.05±0.04<br>***-0.07*±0.04**	-0.32±0.04<br>***0.21*±0.04**
$M6_{Et}$	X	-1.46±0.04	-1.20±0.04	-1.02±0.04	-1.05±0.04	-0.80±0.04	-0.79±0.04	X	-0.77±0.04	-0.92±0.04	-0.20±0.04
R7	X	-0.36±0.04	-0.41±0.04	-0.62±0.04	-0.62±0.04	-0.48±0.04	-0.32±0.04	X	-0.59±0.04	-0.63±0.04	-0.39±0.04
M7	X	-2.33±0.10<br>***-1.97*±0.10**	-1.94±0.09<br>***-1.53*±0.09**	-1.59±0.06<br>***-0.97*±0.06**	-1.18±0.04<br>***-0.56*±0.04**	-1.24±0.04<br>***-0.76*±0.04**	-1.10±0.04<br>***-0.78*±0.04**	X	-1.22±0.04<br>***-0.63*±0.04**	-1.05±0.04<br>***-0.42*±0.04**	-0.25±0.04<br>***0.14*±0.04**

	**$CDCl_3/CD_2Cl_2$**	**$CDCl_3/CD_2Cl_2$**	**CD_2Cl_2**	**Dioxane-d_8**	**Nitromethane-d_3**	**THF-d_8**	**Nitrobenzene-d_5**	**CD_3OD**	**Pyridine-d_5**	**Acetonitrile-d_3**	**DMSO-d_6**
R8	**X**	-0.16±0.04	-0.15±0.04	-0.32±0.040	-0.55±0.04	-0.22±0.04	-0.05±0.04	**X**	-0.30±0.04	-0.39±0.04	-0.16±0.04
M8	**X**	-2.36±0.10<br>**_-2.20_±0.10**	-1.93±0.09<br>**-_1.78_±0.09**	-1.34±0.04<br>**_-1.02_±0.04**	-0.97±0.04<br>**_-0.42_±0.04**	-0.99±0.04<br>**_-0.77_±0.04**	-0.95±0.04<br>**_-0.90_±0.04**	**X**	-1.06±0.04<br>**_-0.76_±0.04**	-0.73±0.04<br>**_-0.34_±0.04**	0.18±0.04<br>**_0.34_±0.04**
R9	**X**	-0.25±0.04	-0.27±0.04	-0.36±0.04	-0.46±0.04	-0.22±0.04	-0.07±0.04	**X**	-0.26±0.04	-0.52±0.04	-0.11±0.04
M9	**X**	-1.35±0.04<br>**_-1.10_±0.04**	-1.08±0.04<br>**_-0.81_±0.04**	-0.40±0.04<br>**_-0.04_±0.04**	-0.62±0.04<br>**_-0.16_±0.04**	-0.16±0.04<br>**_0.06_±0.04**	-0.40±0.04<br>**_-0.33_±0.04**	**X**	0±0.04<br>**_0.26_±0.04**	-0.49±0.04<br>**_0.03_±0.04**	0.32±0.04<br>**_0.43_±0.04**
R10	**X**	-0.86±0.04	-0.68±0.04	-0.71±0.04	-0.68±0.04	-0.64±0.04	-0.50±0.04	**X**	-1.01±0.04	-0.70±0.04	-0.25±0.04
M10	**X**	-1.70±0.05<br>**_-0.84_±0.06**	-1.48±0.06<br>**_-0.80_±0.06**	-0.94±0.04<br>**_-0.23_±0.04**	-0.89±0.04<br>**_-0.21_±0.04**	-0.68±0.04<br>**_-0.04_±0.04**	-0.75±0.04<br>**_-0.25_±0.04**	**X**	-0.77±0.04<br>**_0.24_±0.04**	-0.86±0.04<br>**_-0.16_±0.04**	0.15±0.04<br>**_0.40_±0.04**
R11	**X**	**X**	-0.66±0.04	**X**	**X**	**X**	**X**	**X**	**X**	**X**	**X**
M11	**X**	**X**	-0.12±0.04<br>**_0.54_±0.04**	**X**	**X**	**X**	**X**	**X**	**X**	**X**	**X**
R12	**X**	-0.22±0.04	-0.15±0.04	-0.20±0.04	-0.26±0.04	0±0.04	-0.02±0.04	**X**	**X**	-0.21±0.04	**X**
M12	**X**	0.64±0.06<br>**_0.86_±0.06**	0.57±0.06<br>**_0.72_±0.06**	0.21±0.04<br>**_0.41_±0.04**	0.33±0.04<br>**_0.59_±0.04**	0.30±0.04<br>**_0.30_±0.04**	0.73±0.06<br>**_0.75_±0.06**	**X**	0.10±0.04<br>-	0.55±0.04<br>**_0.76_±0.04**	0.61±0.04<br>**_0.29_±0.04**
R13	**X**	**X**	-0.31±0.04	**X**	**X**	**X**	**X**	**X**	**X**	**X**	**X**
M13	**X**	**X**	0.02±0.04<br>**_0.33_±0.04**	**X**	**X**	**X**	**X**	**X**	**X**	**X**	**X**

Table S3. Equilibrium $K_{Z/E}$ (dichloromethane-d_2, 293 K) and $\Delta G_{Z/E}$ (kcal/mol, in bold) values measured with **M6** and **R6** employing aldehydes **a1-a3** and alternative counterions.

	**bistriflylimide**	**bromide**	**iodide**	**acetate**	**benzoate**
R6/a1	1.3<br>**-0.14±0.03**	=	=	=	=
M6/a1	8.0<br>**-1.21±0.05**	8.1<br>**-1.21±0.05**	7.5<br>**-1.17±0.05**	6.5<br>**-1.09±0.05**	6.0<br>**-1.04±0.05**
R6/a2	0.029<br>**2.05±0.10**	=	=	=	=
M6/a2	0.100<br>**1.34±0.08**	=	=	=	=
R6/a3	0.083<br>**1.44±0.08**	X	X	=	=
M6/a3	0.333<br>**0.64±0.03**	X	X	13.8<br>**-1.52±0.08**	7.0<br>**-1.13±0.05**

Table S4. Charge contributions $\Delta G_{charge,}$(kcal/mol) measured for **M6** in dichloromethane-d_2 at 293 K, employing aldehydes **a1-a3** and alternative counterions.

	**bistriflylimide**	**bromide**	**iodide**	**acetate**	**benzoate**
M6/a1	**-1.07±0.06**	**-1.07±0.06**	**-1.03±0.06**	**-0.95±0.06**	**-0.90±0.06**
M6/a2	**-0.71±0.13**	=	=	=	=
M6/a3	**-0.80±0.09**	X	X	**-2.96±0.11**	**-2.57±0.09**

Table S5. $[Z]_o/[E]_o$ ratios derived for selected model systems in both dichloromethane-d_2 and acetonitrile-d_3 at 293 K. Errors derived from the fitting procedure are shown.

	CD_2Cl_2	***Acetontitrile-d_3***
R0	1.1±0.08	1.9±0.14
M1	1.2±0.09	2.5±0.19
M2	3.2±0.24	n.d
R3	3.8±0.28	6.5
M3	**5.7**±0.57	8.7±0.87
R4	1.1±0.08	2,3±0.17
M4	n.d.	4.0±0.3
R5	1.6±0.12	3.4±0.26
M5	12.6±1.89	6.0±0.6
R6	4.0±0.3	7.4±0.74
M6	9.9±0.99	6.4±0.64
R7	1.9±0.14	n.d.
M7	3.0±0.23	2.5±0.19
R8	1.4±0.11	2.8±0.21
M8	6.9±0.52	1.6±0.12
R9	1.7±0.13	3.7±0.28
M9	2.3±0.17	2.6±0.20
R10	2.1±0.16	3.5±0.26
M10	3.5±0.26	3.0±0.23
M12	0.1±0.01	0.057±0.008

Table S6. α and β values employed in linear regressions (Figure 6 and S16)

	**αs**	**βs**
Chloroform	2.2	0.9
Dichloromethane	1.9	1.1
Nitrobenzene	1.35	4.12
Nitromethane	1.8	3.7
Dioxane	0.9	4.59
THF	0.9	5.9
Acetonitrile	1.7	5.1
Pyridine	1.4	7.0
Dmso	2.2	8.7

Table S7.- To provide an experimental validation for the confidence windows considered in Tables S1-S5, we completed measurements for a reduced set of representative models in triplicate. Individual $K_{Z/E}$ and $\Delta G_{Z/E}$ values (kcal/mol) are presented together with the corresponding mean values and standard deviations.

**Model/Solvent**	**Experiment 1**		**Experiment 2**		**Experiment 3**		**Mean±SD**
	$K_{Z/E}$	$\Delta G_{Z/E}$	$K_{Z/E}$	$\Delta G_{Z/E}$	$K_{Z/E}$	$\Delta G_{Z/E}$	$\Delta G_{Z/E}$
**M3/ CD_2Cl_2**	3.1	–0.66	2.9	–0.62	2.9	–0.62	–0.63±0.02
**R6/CD_2Cl_2**	1.2	–0.11	1.3	–0.15	1.3	–0.15	–0.14±0.02
**M6/CD_2Cl_2**	7.9	–1.20	8.0	–1.21	8.10	–1.21	–1.21±0.01
**M8/CD_2/Cl_2**	11.1	–1.40	12.6	–1.47	14.0	–1.53	–1.47±0.07
**R10/CD_2Cl_2**	1.5	–0.23	1.4	–0.19	1.5	–0.23	–0.22±0.02
**M10/CD_2Cl_2**	6.2	–1.06	5.9	–1.03	5.6	–1.00	–1.03±0.03
**M3/CD_3CN**	7.1	–1.14	6.5	–1.09	6.9	–1.12	–1.12±0.03
**M6/CD_3CN**	4.9	–0.92	4.7	–0.90	4.6	–0.89	–0.90±0.02
**M6/CD_3OD**	16.7	–1.63	14.0	–1.53	15.5	–1.59	–1.58±0.05

5. Figures S1-S21

Figure S1.- a) HSQC spectra acquired after addition of ^{13}C-labelled aldehyde **a0** to 3-amine-3-deoxy-allose **R0** derivatives with β (top) and α (bottom) anomeric configurations in dichloromethane at 293 K. Enamine species are dominant over imines only in the latter case. b) Enamine/imine equilibria in β (Left) and α (Right) 3-amine-3-deoxy-allose derivatives. An α-configuration of the anomeric position allows for the stabilization of the enamine species by hydrogen bonding interactions. On the contrary imine species are electrostatically repelled.

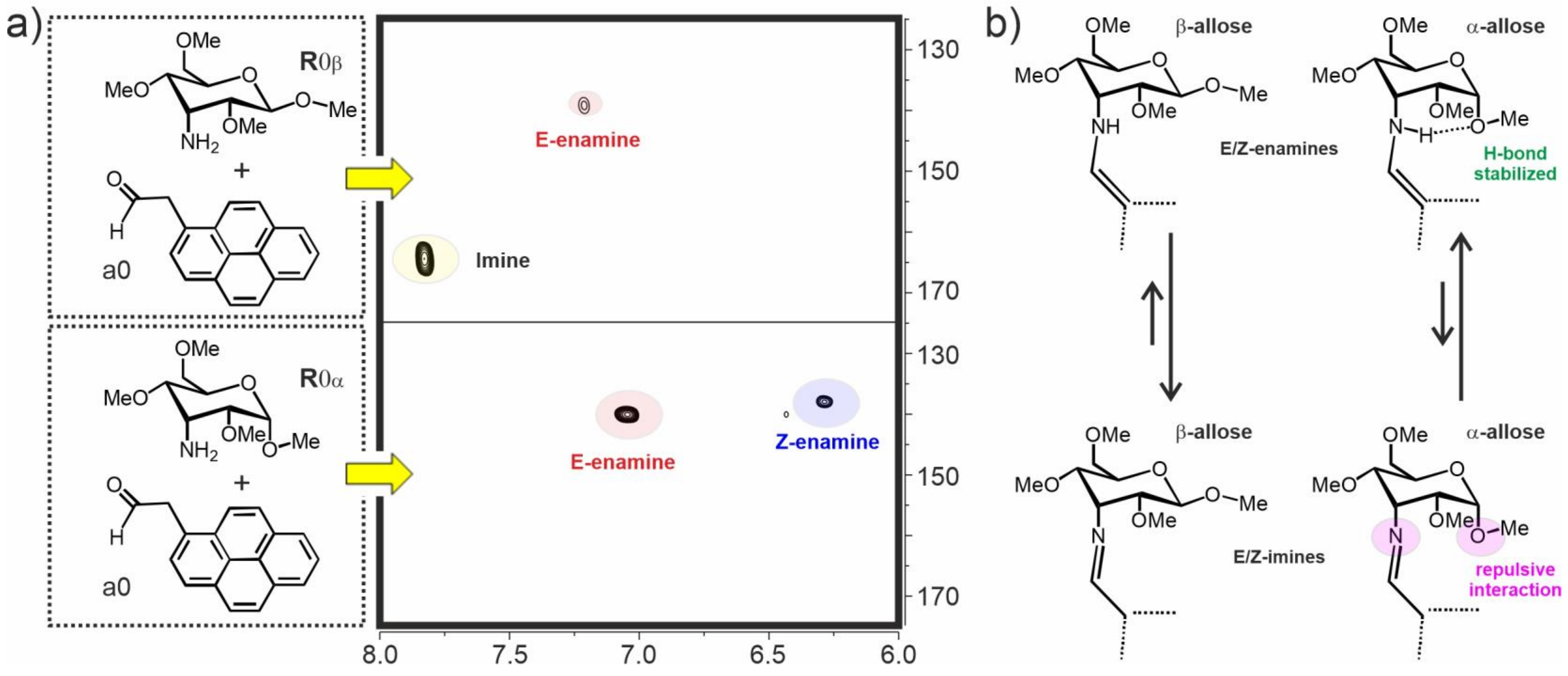

Figure S2.- Key regions of NOESY(left) and TOCSY(right) experiments acquired for the stereoselective assignment of **M5** in dichloromethane-d_2 at 273K. Key NOE contacts observed for **M5** and absent with **M4** are represented with red arrows above.

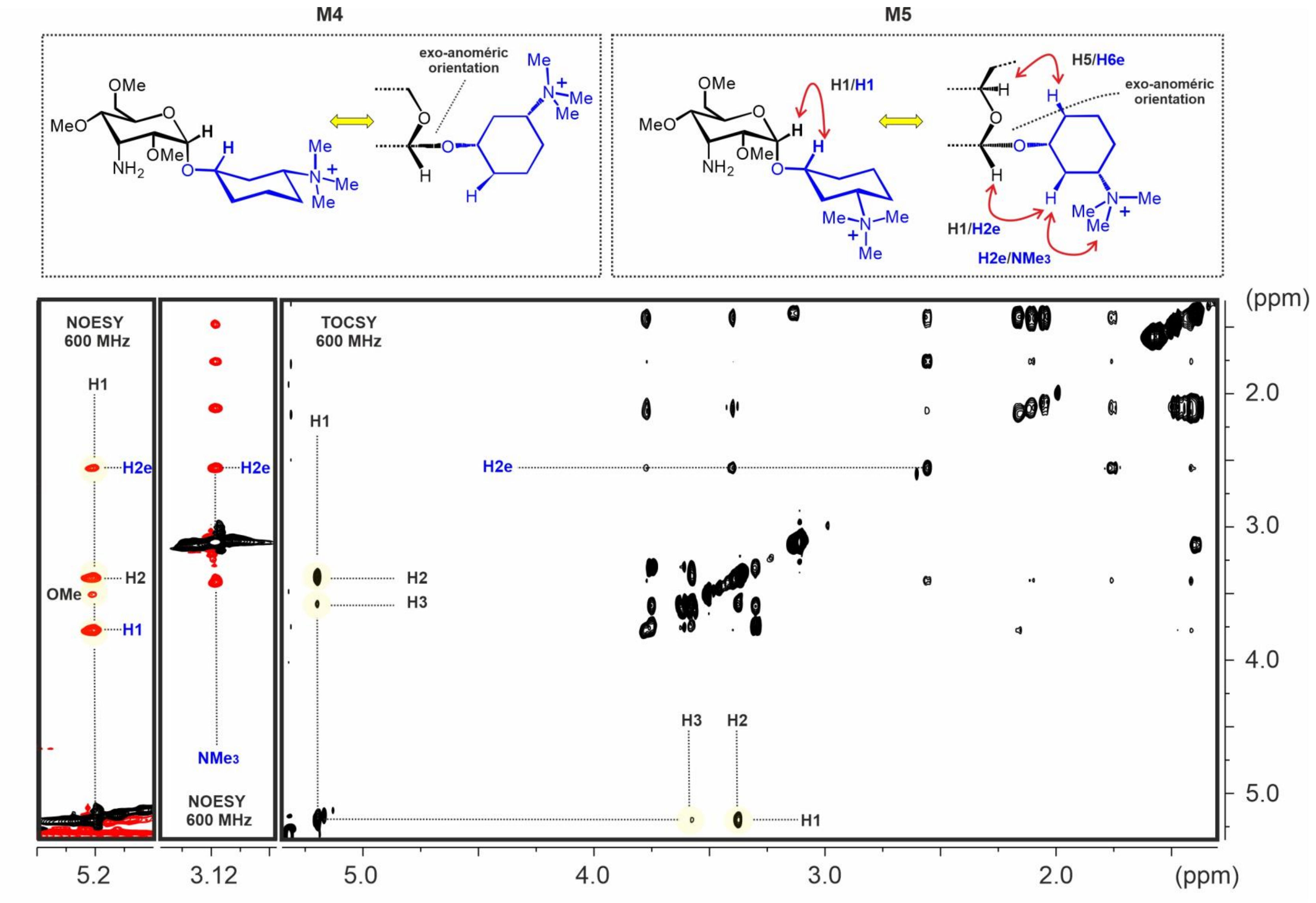

Figure S3.- Key regions of NOESY TOCSY and HSQC experiments acquired for the stereoselective assignment of **R12/M12** in dichloromethane-d2 at 273K. Key NOE contacts observed for **R12/M12** and absent with **R13/M13** are represented with red arrows.

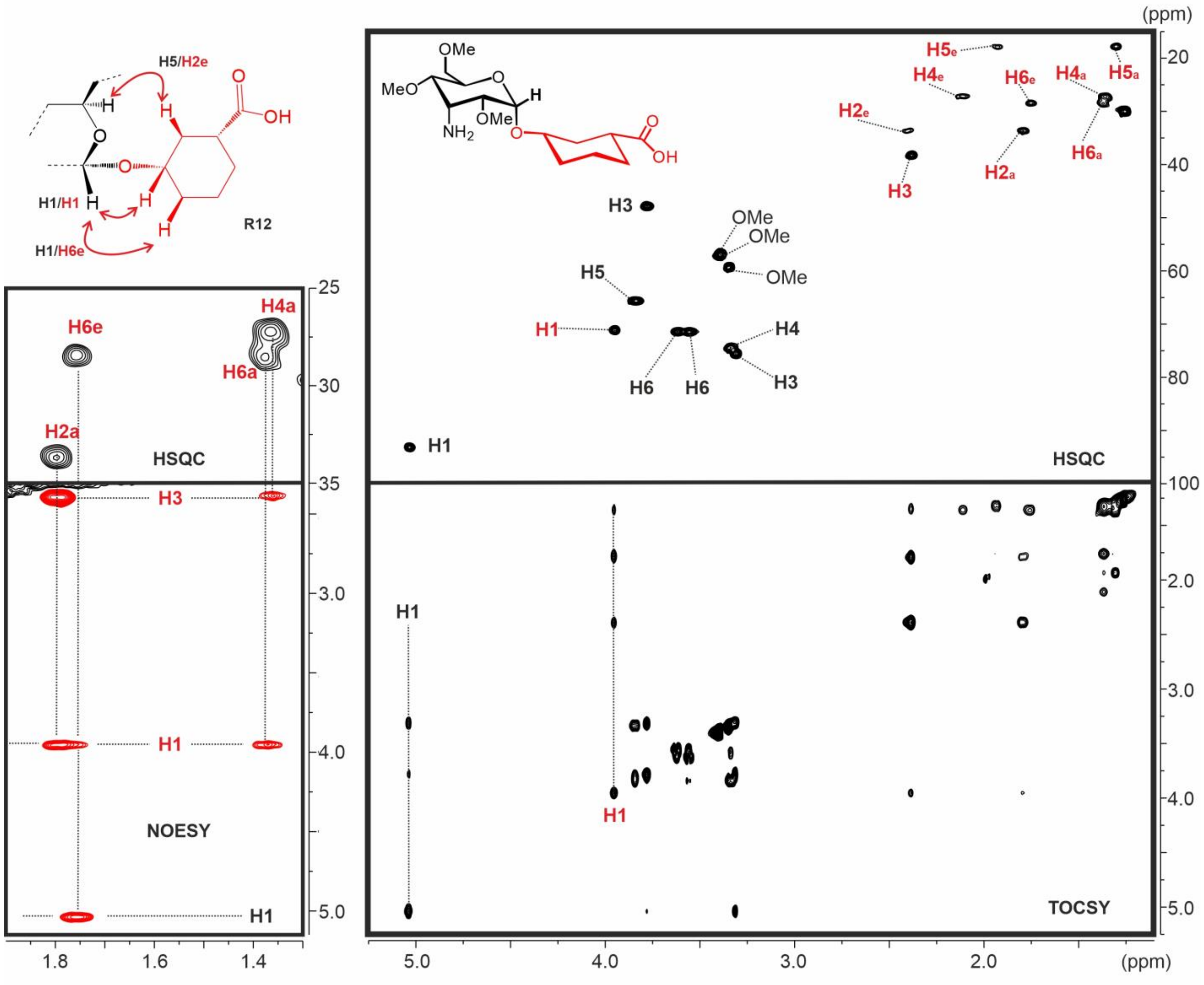

Figure S4.- Left.- HSQC spectrum acquired after addition of ^{13}C-labelled aldehyde **a1** to a solution of **R6** in dichloromethane-d$_2$ at 293 K. Enamine and aldehyde signals are labelled. Key NOE contacts employed for the assignment of the Z/E enamine species are represented above. Right.- Key Section of a NOESY experiment (800 ms mixing time) acquired after addition of unlabelled aldehyde **a1** to a solution of **R6** in dichloromethane-d$_2$ at 293 K

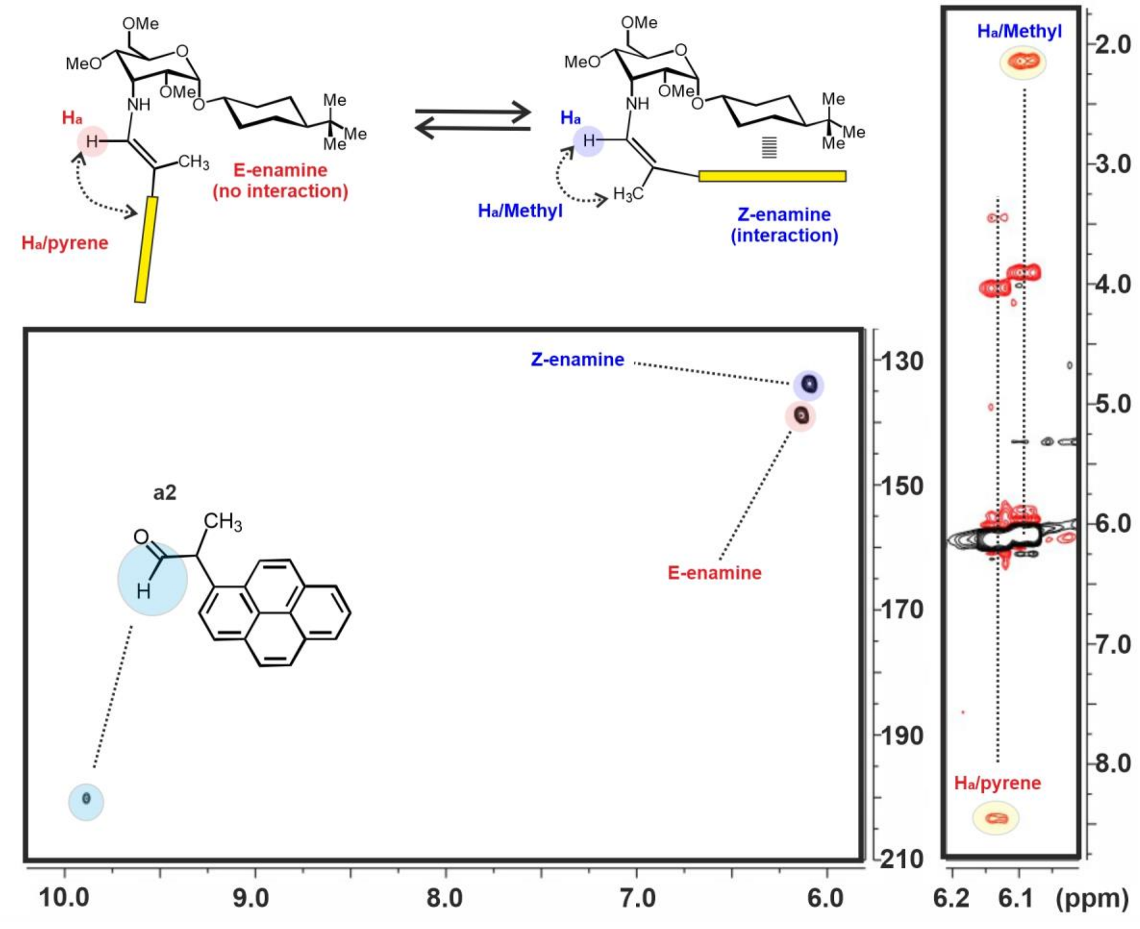

Figure S5.- *Z* enamine formation is in all cases accompanied by the appearance of strongly up-field shifted signals in the 1D spectra, indicative of shielding derived from aromatic complex formation. To exemplify this phenomenon, key regions of 1D NMR spectra, acquired after mixing models **R6** or **R9** with aldehyde **a1** in acetonitrile-d_3, dioxane-d_8 and THF-d_8 are shown on the left. A key region of a NOESY experiment acquired after mixing model **M5** with aldehyde **a1** in acetonitrile-d_3 is represented on the right.

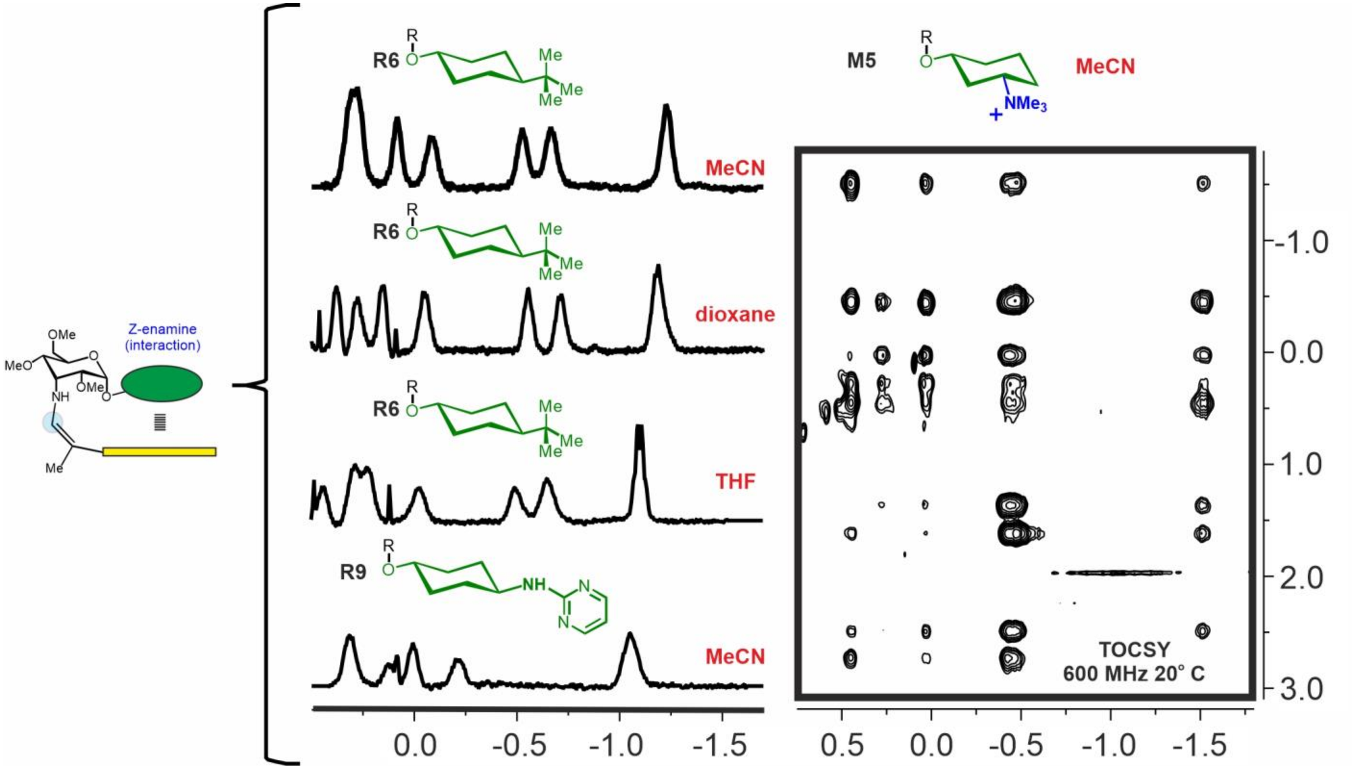

Figure S6.- Top.- Simple kinetic model for enamine formation considered in our Gepasi kinetic simulations. Bottom.- Theoretical curves calculated assuming a fast or slow bi-molecular adduct formation on the reaction time-scale. The employed kinetic constants (in M^{-1} s^{-1} or s^{-1} units for bimolecular and unimolecular processes, respectively) are indicated. For slow bimolecular associations, the enamine Z/E concentration ratio approaches equilibrium faster than the individual *Z* or *E* curves. In these circumstances, the equilibrium constant can be derived even from incomplete kinetic experiments (as illustrated in the bottom-right corner). *Z/E* ratios were approximated by a simple exponential function and the resulting values for the equilibrium constant and rate constant ratios are indicated. Curves marked with an asterix were best approximated by a sum of simple exponentials (see page S50).

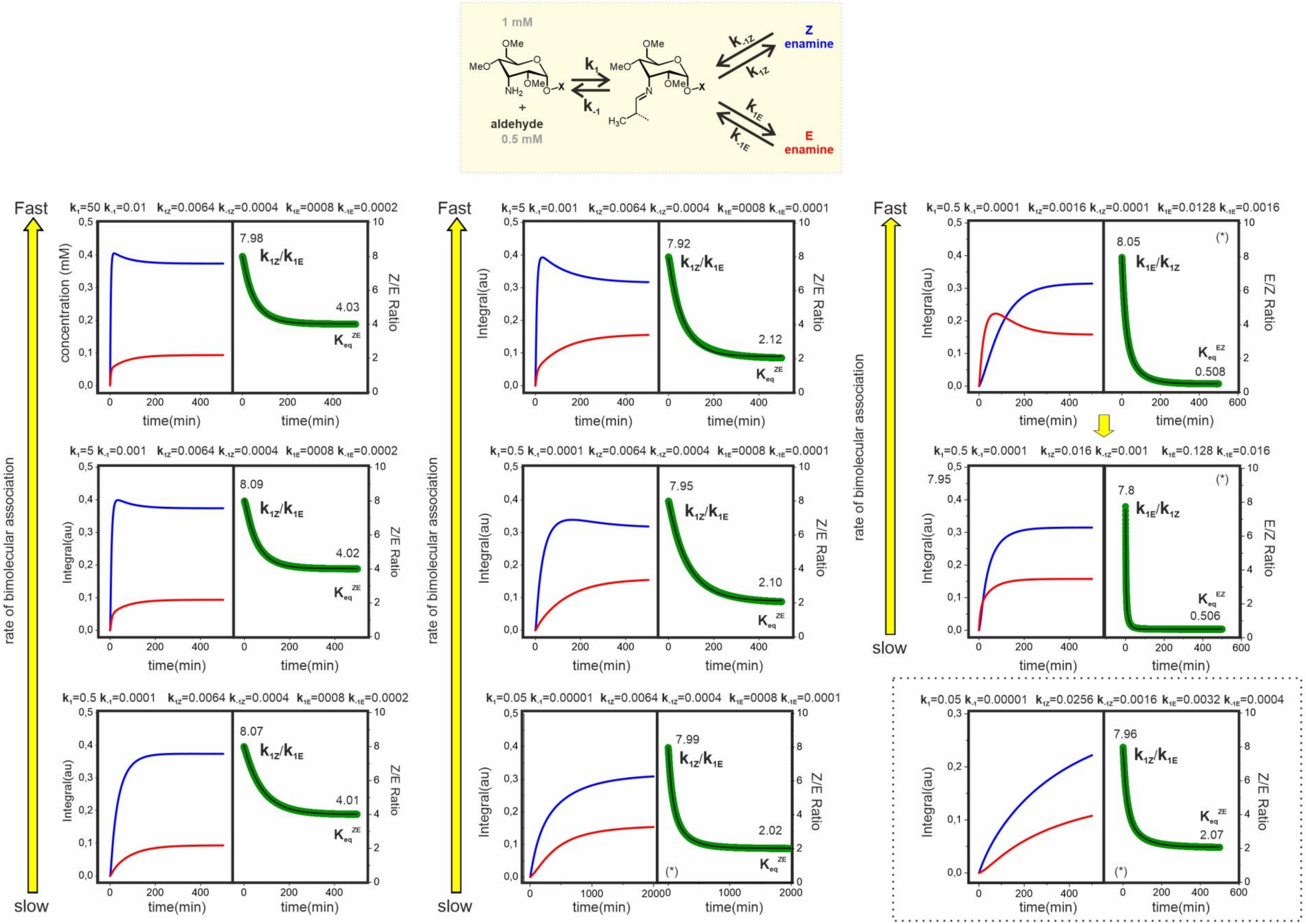

1 mM
aldehyde
0.5 mM
Z enamine
E enamine
Fast
slow
rate of bimolecular association
time(min)
Integral(au)
concentration (mM)
Z/E Ratio
E/Z Ratio

Figure S7.- Examples of reactions performed in dichloromethane-d_2 at 293 K, employing aldehyde **a1.** Time evolution of enamine Z/E ratios are represented on the right together with the fitted curves and the derived $K_{eq}^{Z/E}$ and k_Z/k_E values

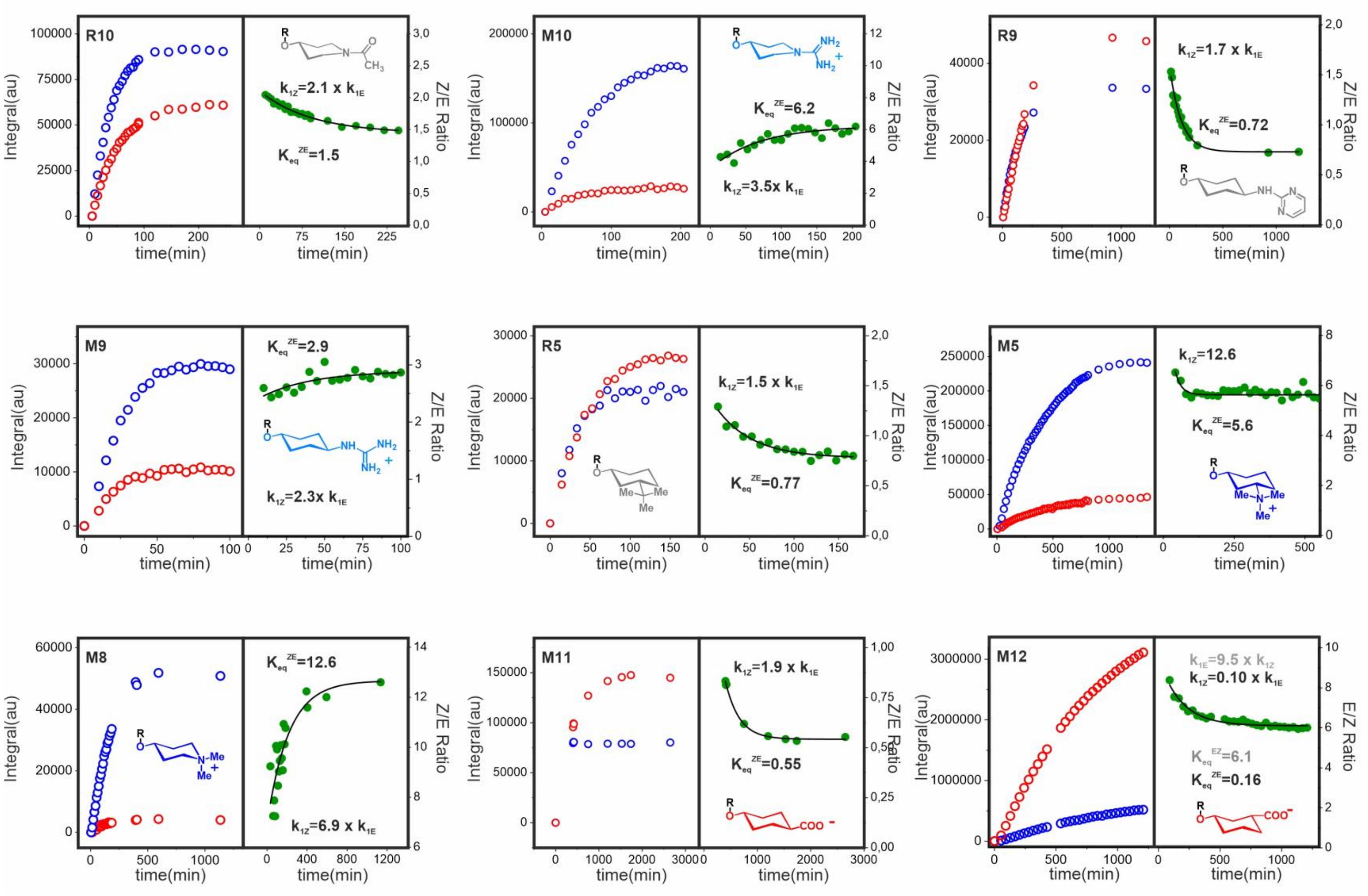

Figure S8.- Molecular dynamics ensembles obtained with models **M6** (top) and **M9** (bottom) in explicit dichloromethane. Simulation lengths were 1 μs. Pyrene unit is represented in yellow, **M6**/**M9** interacting units in blue or cyan, respectively and the bistriflylimide counterion in red. Closer ion/counterion contacts are observed for **M9** reflecting the excellent hydrogen bonding donor properties of the guanidinium function. Distances between the bistriflimide nitrogen and those of **M6** (top) and **M9** (bottom) charged moieties, calculated over all frames the simulations (totaling 25,000 data points), are represented as scatter plots on the right. For the guanidinium fragment different patterns of interaction are possible which determines a complex distance distribution for the three nitrogen atoms. Mean values calculated considering only the shortest distance for all frames are represented on the right.

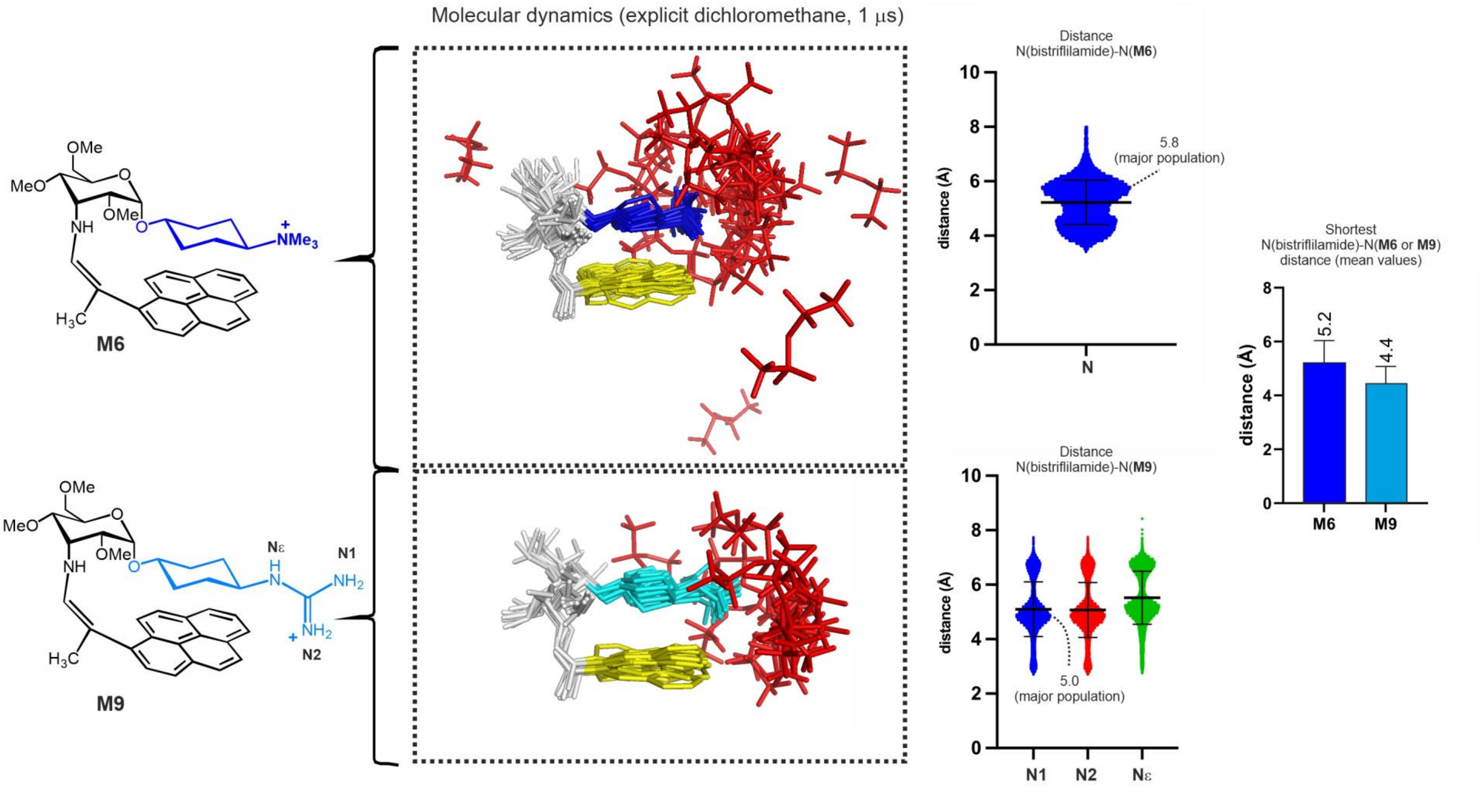

Figure S9.-a) Interaction free energy values measured for CH/π complexes established by cationic tetra-alkylammonium fragments and their neutral references, ΔG_{int} (in black, kcal/mol), together with the derived ΔG_{charge} or ΔG_{pol} (in magenta, kcal/mol). The corresponding free energies for pair **M3**/**R3** are also shown for comparison. Charged complexes display more reduced stability variations than the neutral ones (0.48 vs 0.75 kcal/mol). b) Reduced shape complementarity imposes a higher energy penalty on the neutral CH/π complexes, thus determining larger charge contributions to stability. Two examples of this phenomenon are represented.

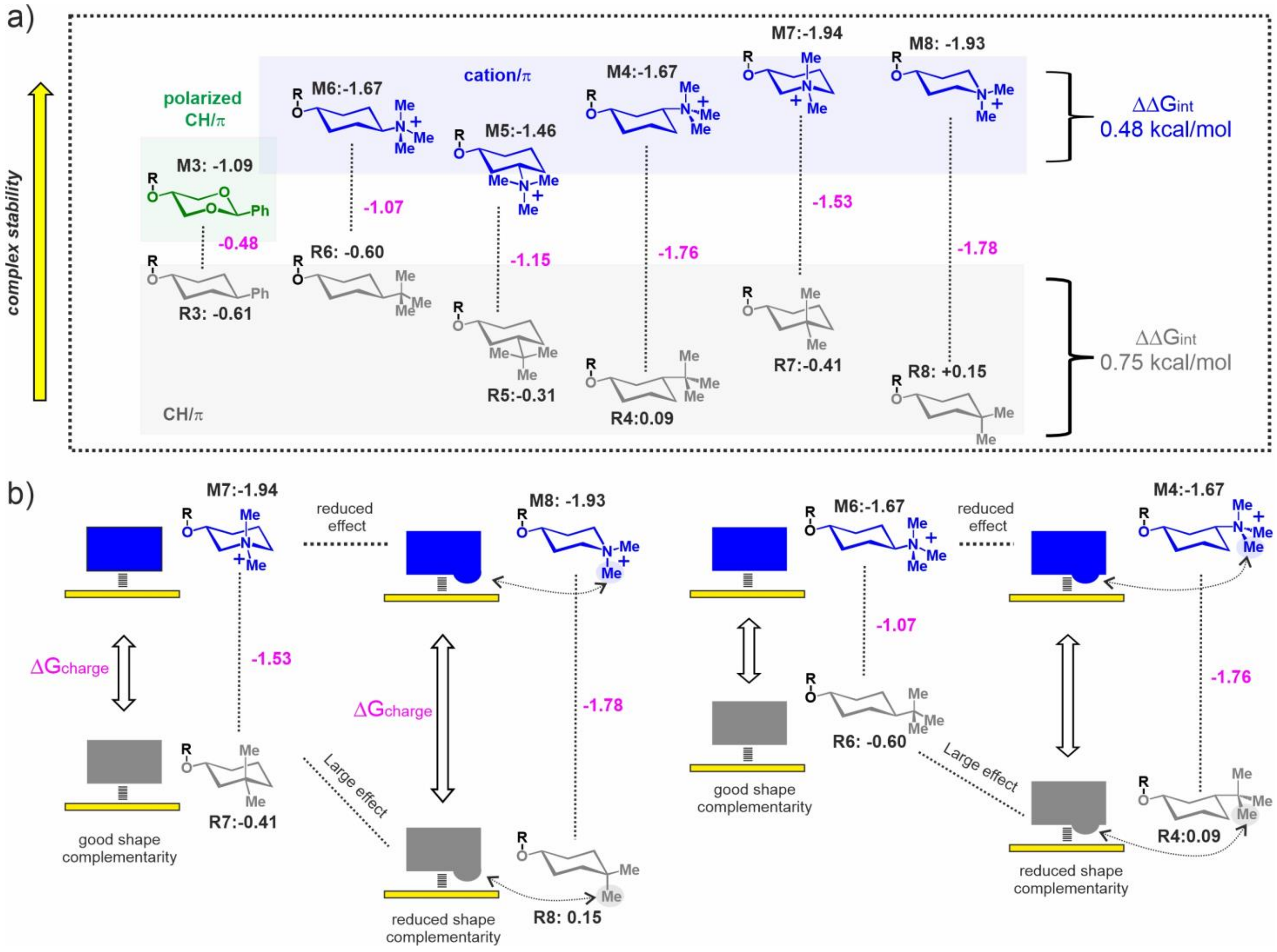

Figure S10.- Chemical reactions performed in NMR tubes with models **R6** (Left) or **M6** (Right) and aldehyde **a1**. Key regions of HSQC experiments acquired 24 h (top) and 500 h (bottom) after the aldehyde addition are displayed to illustrate the chemical stability of the enamine mixtures.

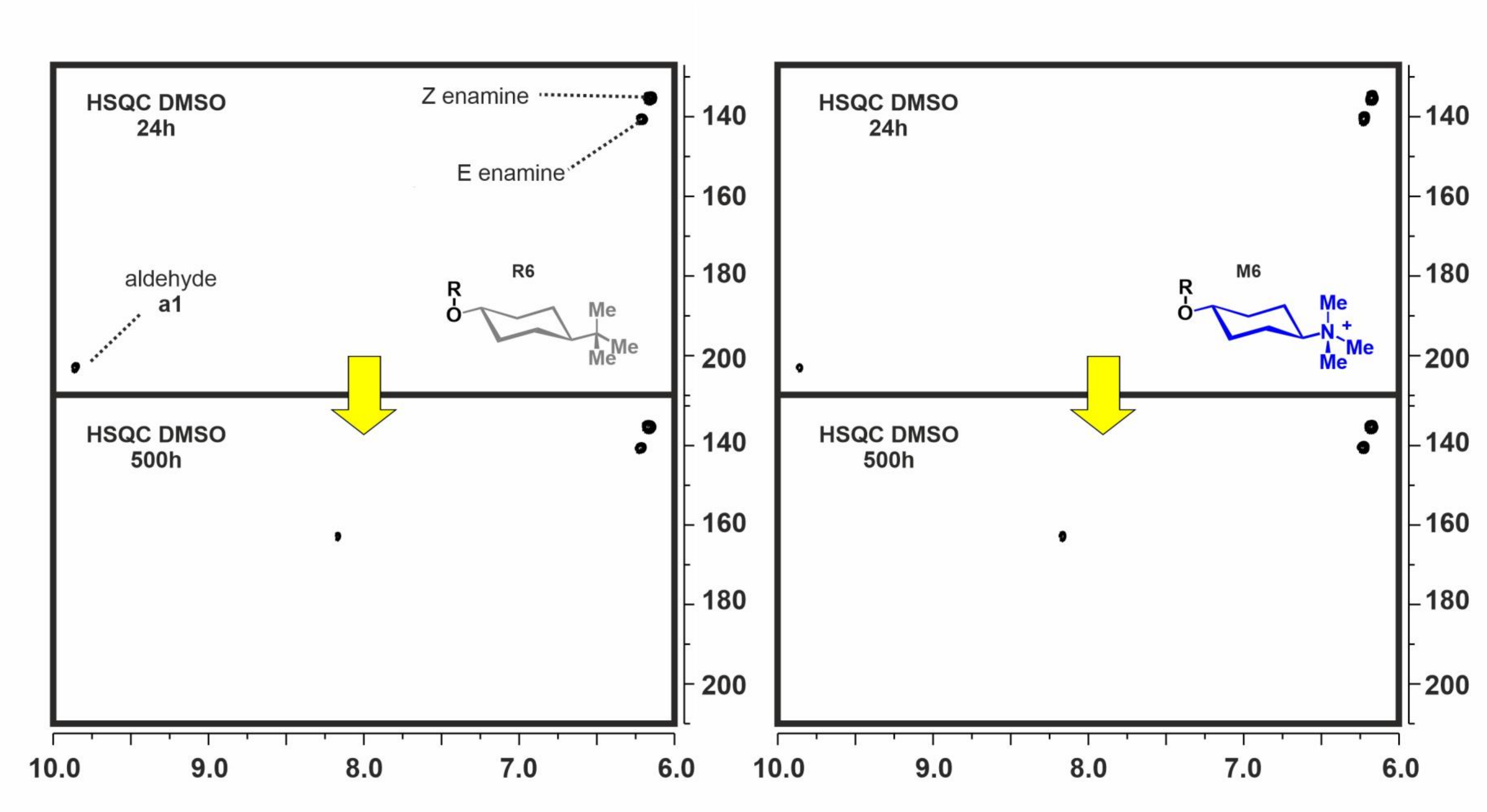

Figure S11.- Chemical reaction performed in an NMR tube with model **R0** (Left) and aldehyde **a1**. Key regions of HSQC experiments acquired 20 minutes (Left) and 14 h (Right) after the aldehyde addition are displayed at the bottom. Integration of Z/E enamine signals (top-left corner) throughout the reaction course allowed us to derive kinetic curves (top-right corner). Time-evolution of the Z/E population ratio is also represented (in green) together with the fitted curve (black) and the derived $K_{eq}^{Z/E}$ and k_Z/k_E values.

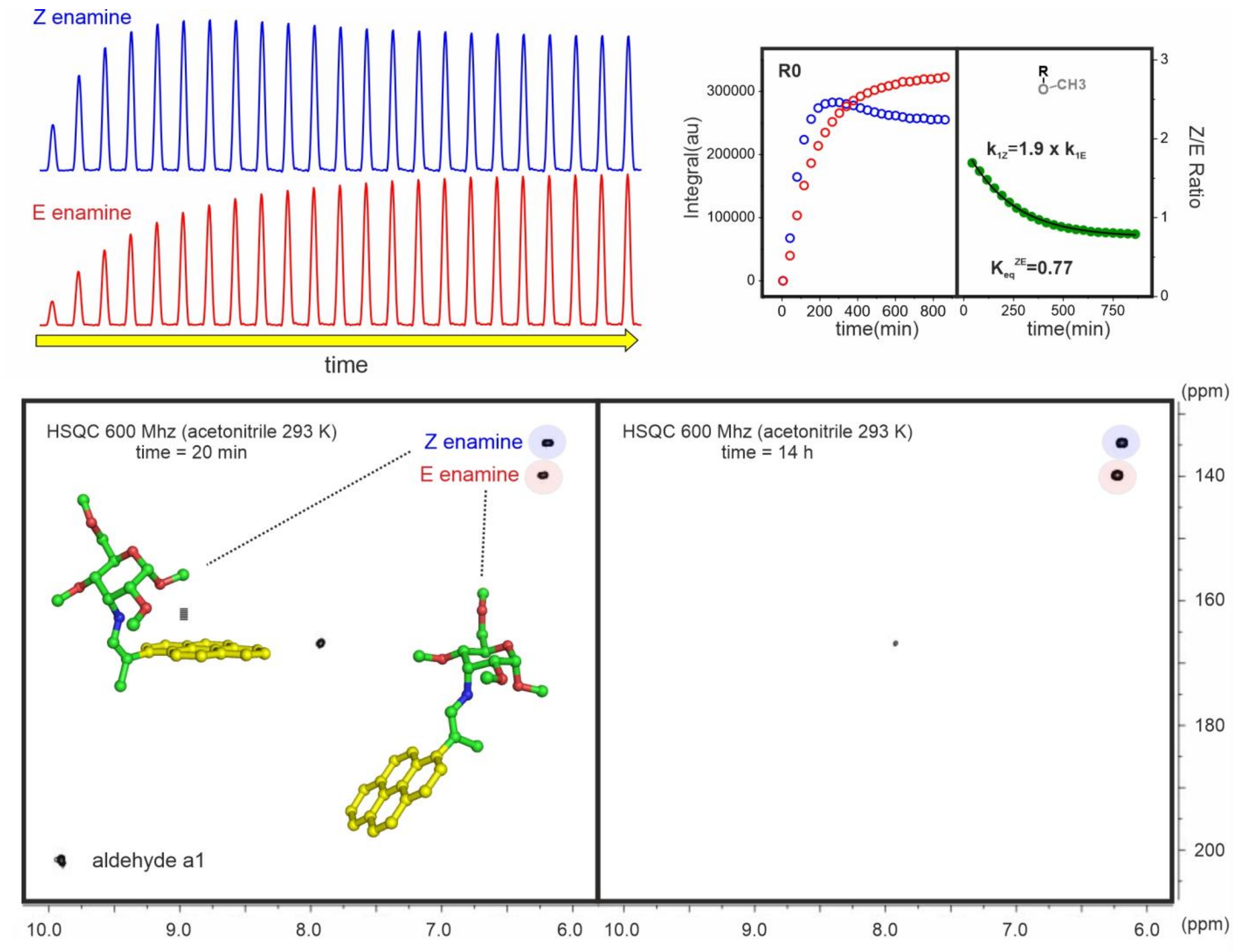

Figure S12.- Reactions performed with different concentrations of the represented models and catalytic TFA in dichloromethane-d_2, acetonitrile-d_3 and pyridine-d_5. Aldehyde **a1** concentration was, in all cases, 0.5 mM. Time evolution of enamine Z/E ratios are represented on the right together with the fitted curves and the derived $K_{eq}^{Z/E}$ and k_Z/k_E values.

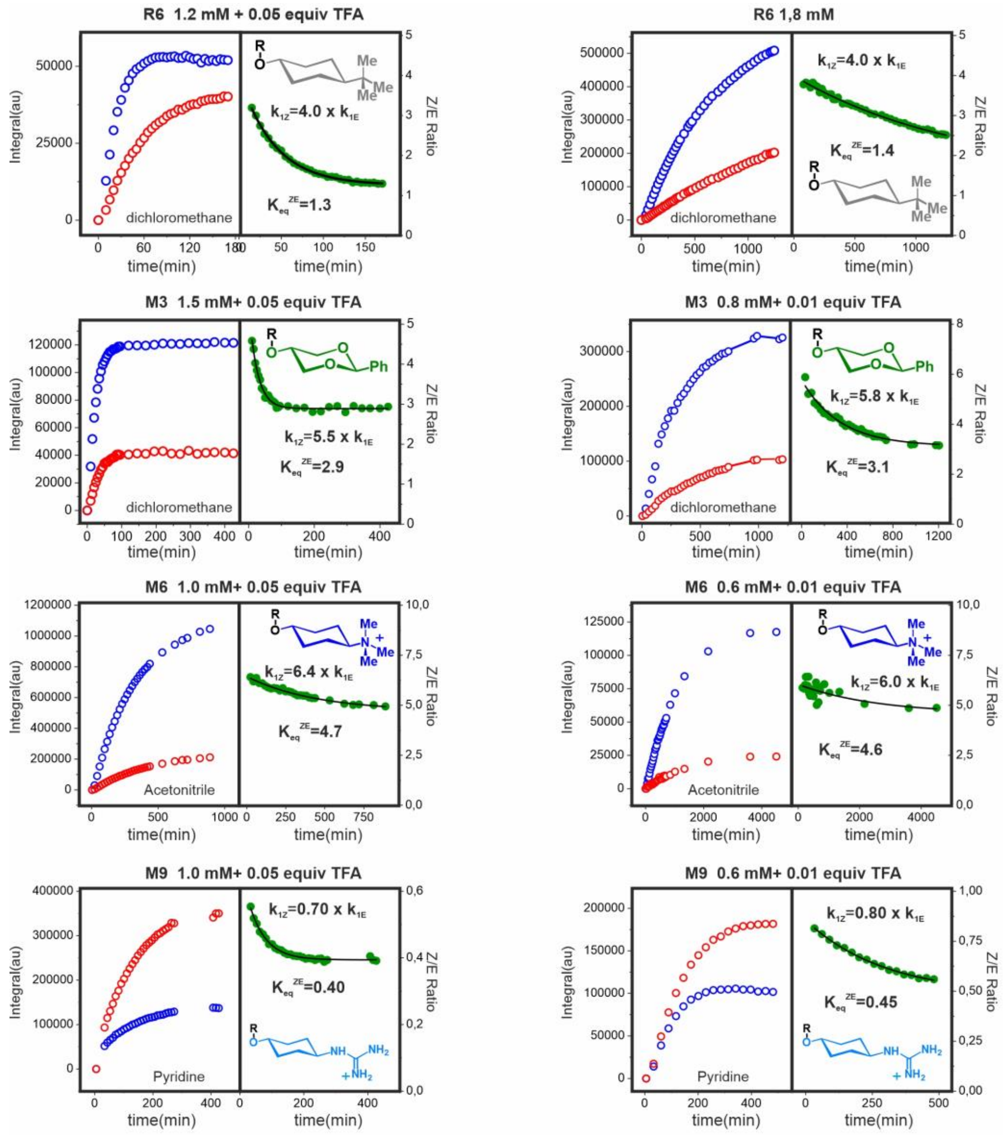

Figure S13.- Kinetic curves obtained at 293 K with aldehyde **a1** and selected models in alternative deuterated solvents**.** Time evolution of enamine Z/E ratios are also represented (green) together with the fitted curves and the derived $K_{eq}^{Z/E}$ and k_Z/k_E values

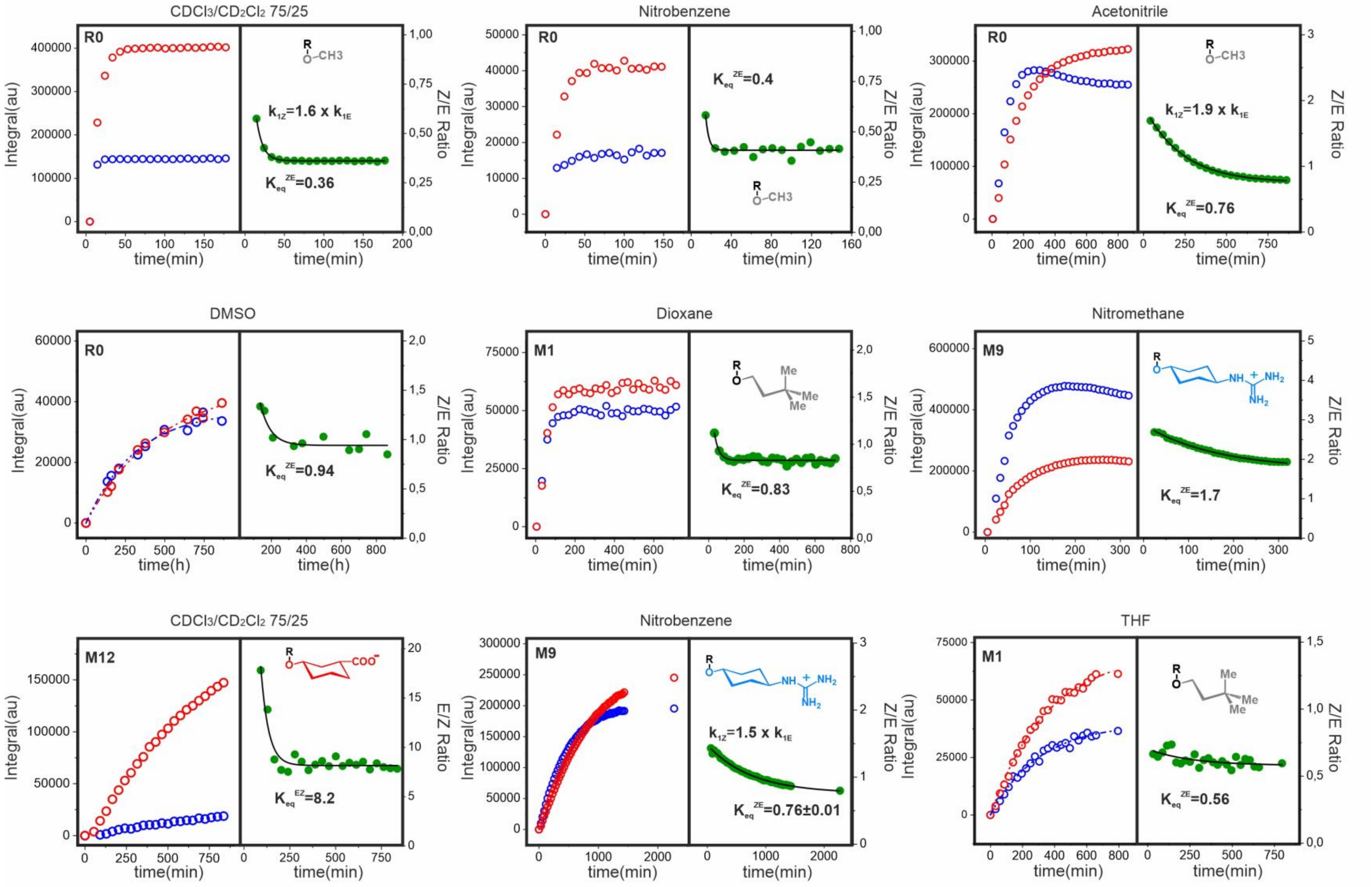

Figure S14.- Kinetic curves obtained at 293 K with aldehyde **a1** and selected models in alternative deuterated solvents**.** Time evolution of enamine Z/E ratios are also represented (green) together with the fitted curves and the derived $K_{eq}^{Z/E}$ and k_Z/k_E values

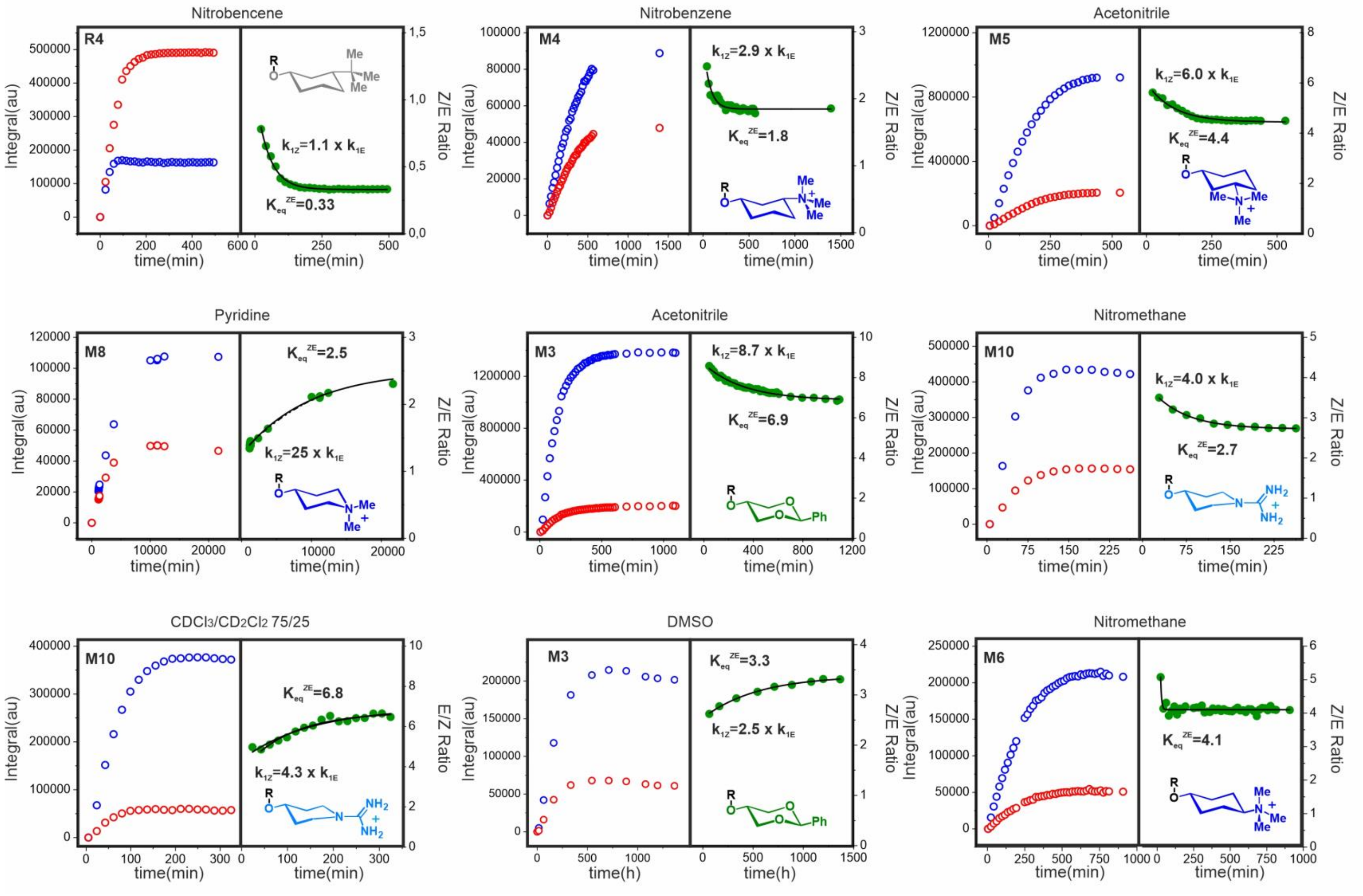

Figure S15.- Kinetic curves obtained at 293 K with aldehyde **a1** and selected models in alternative deuterated solvents**.** Time evolution of enamine Z/E ratios are also represented (green) together with the fitted curves and the derived $K_{eq}^{Z/E}$ and k_Z/k_E values

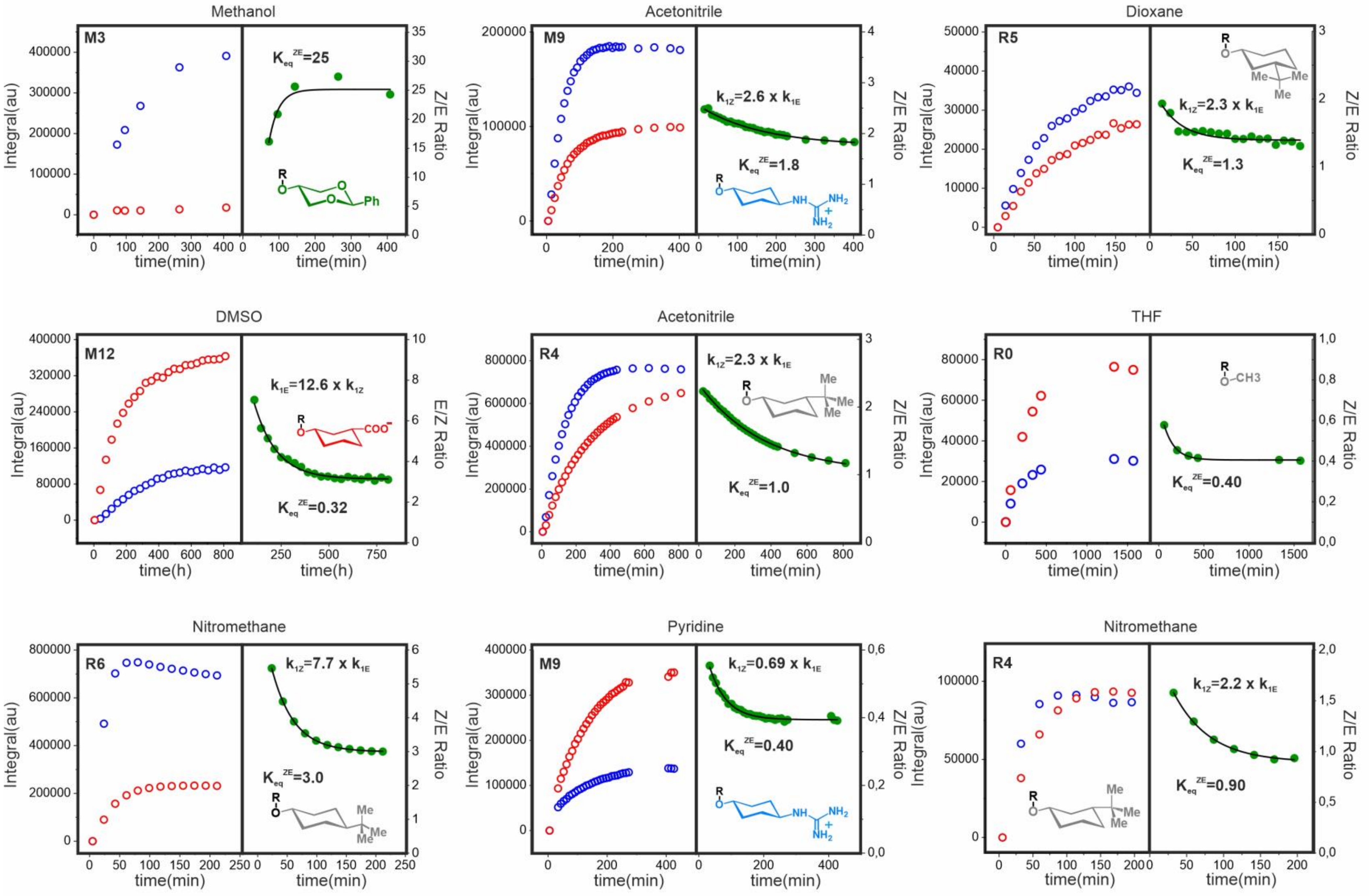

Figure S16.- *a*) ΔG_{charge} or ΔG_{pol} values (kcal/mol) measured for selected complexes in various organic media at 293 K. b) Correlations between ΔG_{charge} or ΔG_{pol} and the solvent H-bond acceptor (β) o donor (α) parameters observed for selected models. For chloroform solvent mixtures, these values were approximated by those of pure chloroform. Methanol free energies were excluded from these analyses due to their larger experimental uncertainties.

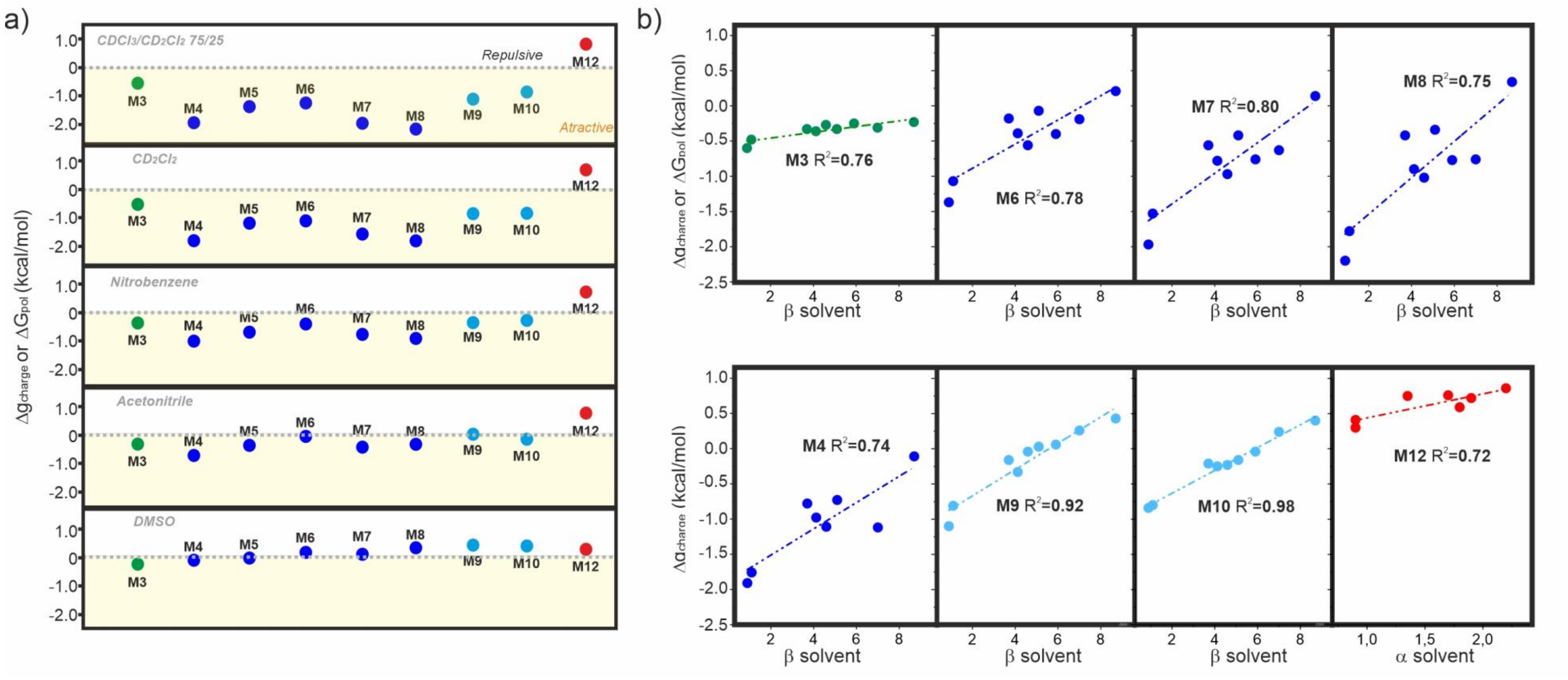

Figure S17.- According to our experiments, aromatic CH/π contacts kinetically favor Z- over E- enamine formation, as proved by the initial [Z]/[E] ratios measured with (bottom-left) and without (top-left) interactions. This behavior probably reflects the stabilizing influence exerted by the aromatic platforms on particular hemiaminal/imine conformations or transition states leading to the Z- stereoisomer (bold path on the Right-hand side of the figure). It should be noted that these putative contacts might exhibit related, but not identical, interaction geometries to those existing in the final Z-product, implying a multitude of potential stabilizing scenarios.

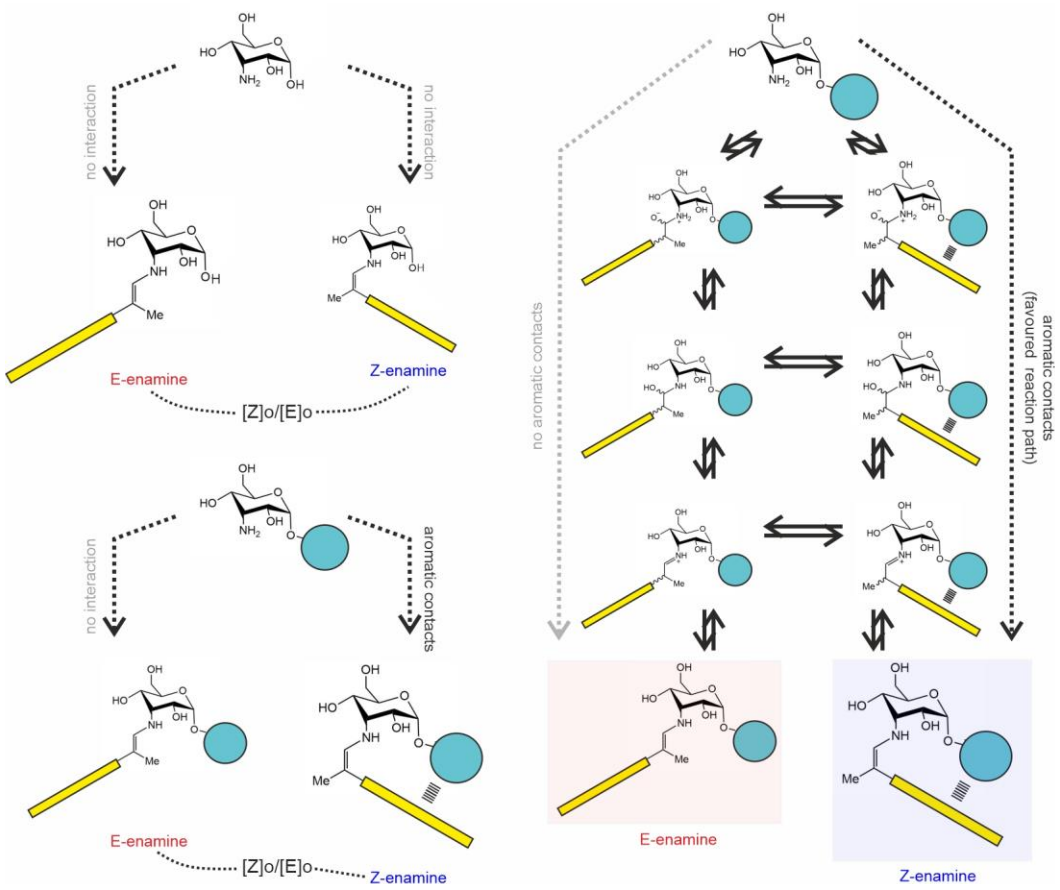

Figure S18.- Logarithmic representation of initial vs equilibrium *Z/E* ratios measured in dichloromethane-d_2 and acetonitrile-d_3 with selected derivatives. Left.-Dotted lines present identical $[Z]_0/[E]_0$ and $[Z]_\infty/[E]_\infty$ values. Right.- Dotted lines result from a linear fitting procedure. R^2 values are indicated.

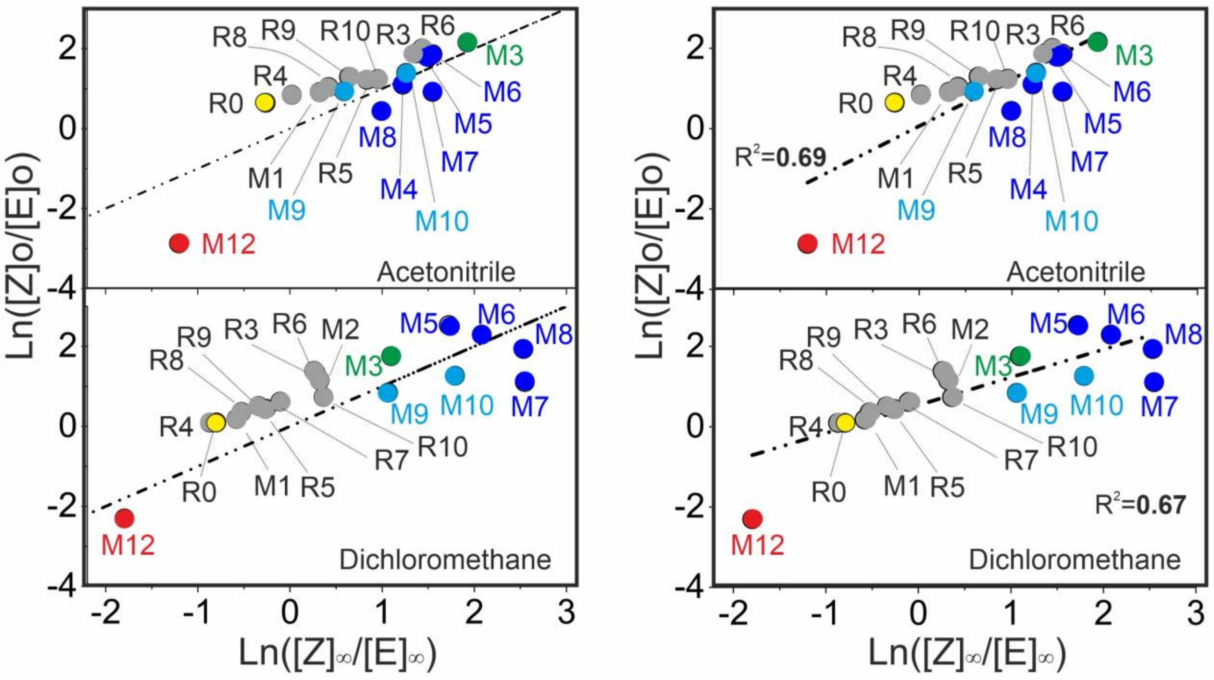

Figure S19.- Initial HSQC data sets from kinetic experiments performed with models **R0**, **R6** and **M6** in dichloromethane (top) and DMSO (bottom). Relative fractions for the Z and E enamines are indicated. In dichloromethane the stabilizing influence of **M6** positive charge is reflected in the relative formation rates of the enamines and determines a large Z/E initial ratio (9.0 for **M6** vs 3.8 for **R6**). The opposite effect is observed in DMSO (Z/E= 1.7 vs 4.6 for **M6** and **R6**, respectively) in agreement with preferred cation/solvent interactions (Table S2).

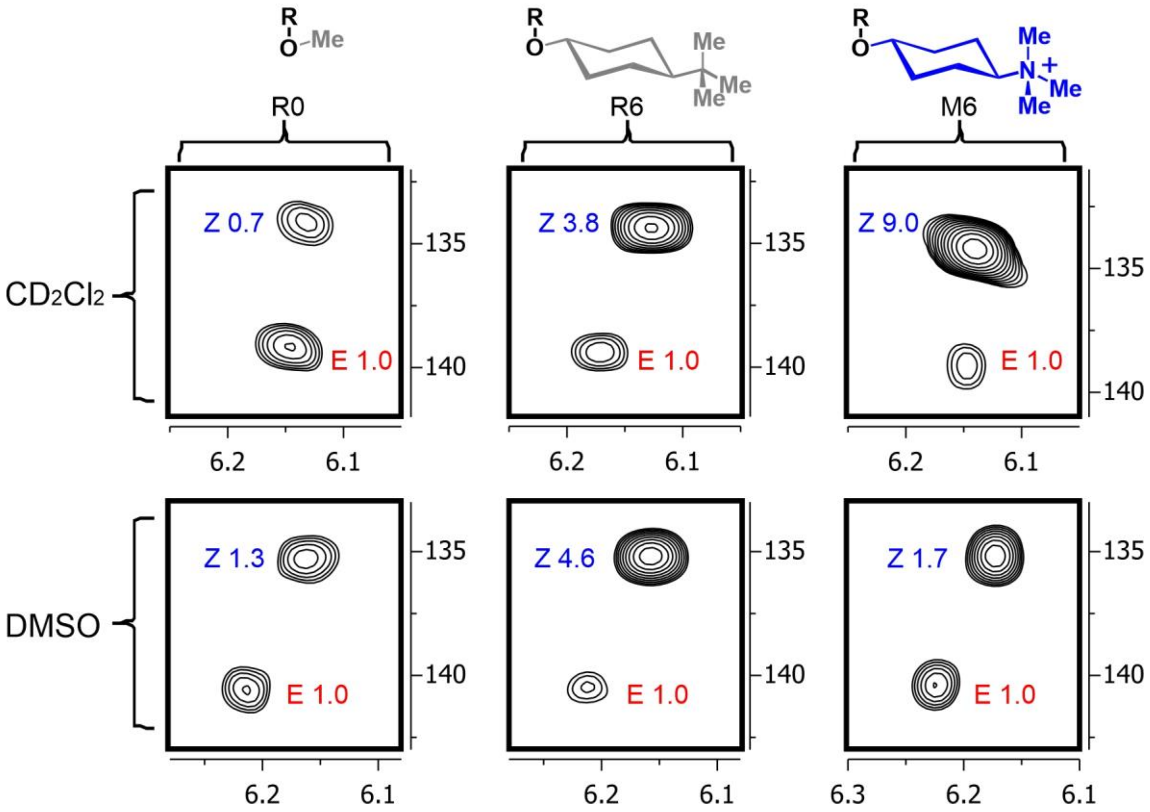

Figure S20.- 1D NMR (600 MHz, CD_2Cl_2 20 °C) experiments acquired with the **R6** reference system both, in the absence (bottom) and presence (top) of 15 mM TBA acetate. Added salt has no effect on this model chemical shifts. On the contrary, with cationic **M6** salt addition translates in significant chemical shift changes as shown by the superimposed HSQC data sets (in black and green). These are especially significant around trimethylammonium fragment and reflect the exchange in counterion (bistriflylimide by acetate)

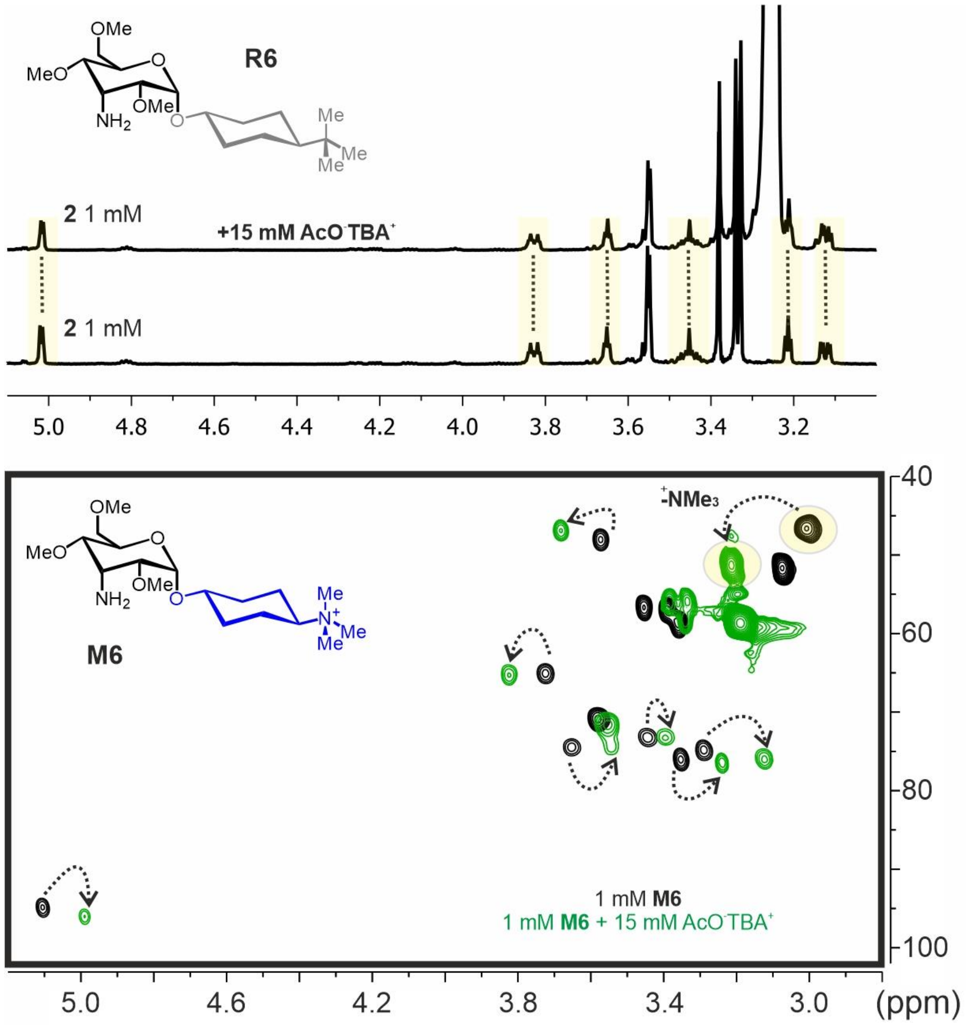

Figure S21.- Key section of HSQC spectra (CD_2Cl_2, 293 K) acquired with **M6** (top) or **R6** (bottom) in the presence of aldehydes **a1** (left), **a2** (middle panel), and **a3** (right). Relative *Z/E* populations at the equilibrium are indicated together with the corresponding $\Delta G_{Z/E}$ (kcal/mol) values. Charge contributions to the stability of the **M6** complexes (ΔG_{charge} (kcal/mol)) are shown in magenta.

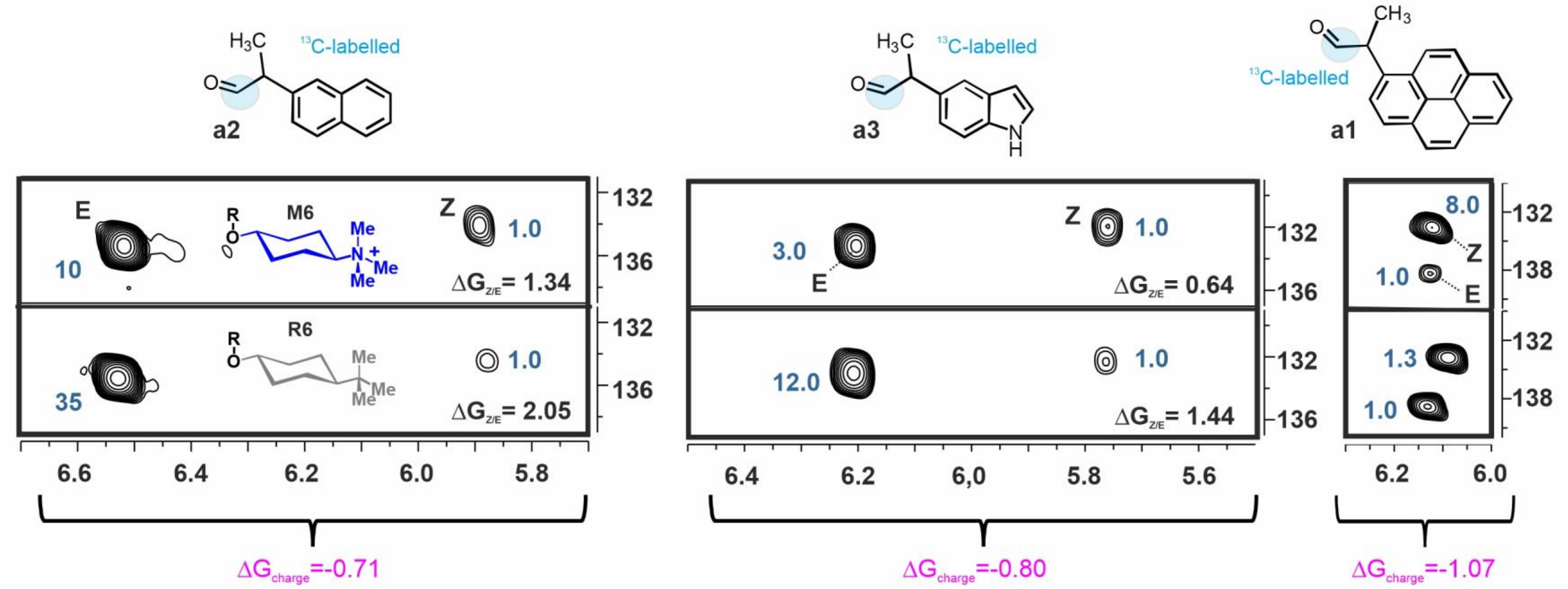

Figure S22.- Kinetic curves corresponding to the formation/equilibration of the molecular glyco-balance formed by treating **M6** (1 mM) with **a2** in dichloromethane, both in the absence and in the presence of alternative tetra-butylammonium (TBA) salts (15 mM). $K_{Z/E}$ equilibrium constants together with corresponding *Z/E* free energy differences (kcal/mol) are shown in black. Charge contributions to the **M6** complex stabilities (ΔG_{charge}) are represented in magenta. Most significant counterion-dependent variations in this parameter ($\Delta\Delta G_{charge}$) are represented in red.

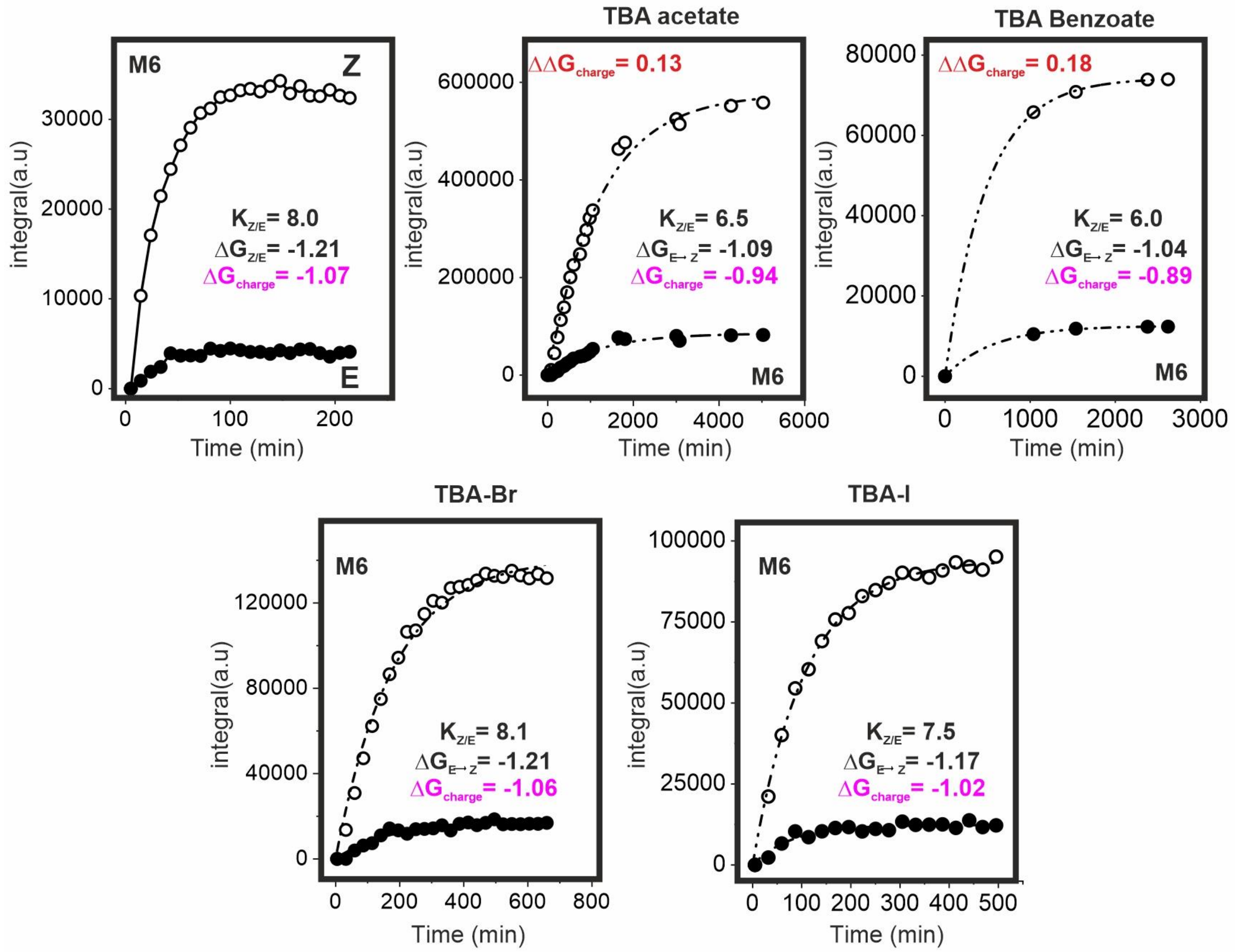

Supplement: Supplementary file 1 — au3c00592_si_001.pdf [file au3c00592_si_001.pdf]
